# Comprehensive primary health care and non-communicable diseases management: a case study of El Salvador

**DOI:** 10.1186/s12939-020-1140-x

**Published:** 2020-04-06

**Authors:** Marta Jimenez Carrillo, Montserrat León García, Nicole Vidal, Keven Bermúdez, Pol De Vos

**Affiliations:** 1 Institute for Global Health and Development, Queen Margaret University, Edinburgh, UK; 2 Biomedical Research Institute Sant Pau (IIBSant Pau), Iberoamerican Cochrane Centre, Universidad Autónoma de Barcelona, Barcelona, Spain

**Keywords:** Comprehensive primary health care, Non-communicable diseases, El Salvador, Community participation

## Abstract

**Background:**

One of today’s greatest challenges in public health worldwide - and especially its key management from Primary Health Care (PHC) - is the growing burden of non-communicable diseases (NCDs). In El Salvador, since 2009 the Minister of Health (MoH) has scaled up a national public health system based on a comprehensive PHC approach. A national multi-sectorial strategic plan for a comprehensive approach to NCDs has also been developed. This analysis explores stakeholders’ perceptions related to the management of NCDs in PHC and, in particular, the role of social participation.

**Methods:**

A case-study was developed consisting of semi structured interviews and official document reviews. Semi-structured interviews were developed with chronic patients (14) and PHC professionals working in different levels within PHC (12). Purposive sampling was used to recruit participants. A non-pure, deductive approach was implemented for coding. After grouping codes into potential themes, a thematic framework was elaborated through a reflexive approach and the triangulation of the data. The research was conducted between March and August of 2018 in three different departments of El Salvador.

**Results:**

The structure and the functioning of the Salvadoran PHC system and its intersectoral approach is firstly described. The interdisciplinary PHC-team brings holistic health care closer to the communities in which health promoters play a key role. The findings reflect the generally positive perception of the PHC system in terms of accessibility, quality and continuity of care by chronic patients. Community engagement and the National Health Forum are ensuring accountability through social controllership mechanisms. However, certain challenges were also noted during the interviews related to the shortage of medication and workforce; coordination between the levels of care and the importance of prevention and health promotion programmes for NCDs.

**Conclusions:**

The Salvadoran PHC and its comprehensive approach to NCDs with an emphasis on intersectoral participation has been positively perceived by the range of stakeholders interviewed. Social engagement and the NHF works as a driving force to ensure accountability as well as in the promotion of a preventive culture. The challenges identified provide keys to amplify knowledge for addressing inequalities in health by strengthening PHC and its NCDs management.

## Introduction

The growing burden of non-communicable diseases (NCDs) represents one of today’s greatest challenges in public health worldwide and it has been highlighted the key management from PHC [1]. NCDs are the leading cause of global mortality and disability worldwide (71% of all deaths globally) [2], with a rising burden in low- and middle-income countries [3] (LMIC). Each year, 15 million people die from an NCD between the ages of 30 and 69 years; over 85% of these “premature” deaths occur in LMICs [4]. The burden created by NCDs undermines individual and family well-being and hampers social and economic development [5]. The rapid rise in NCDs is predicted to impede poverty reduction initiatives in low-income countries, particularly by increasing household costs associated with health care. The multifactorial aetiology of NCDs [6] implies the need for the implementation of integral, intersectoral [7] and political strategies through accountability for health impact [8]. Health in all policies [9] involve an explicit concern for health in all areas of public policy. This seeks to ensure public policies across sectors systematically consider the health implications of decisions, seek synergies, and avoid harmful health impacts in order to improve population health and health equity. Moreover, NCD management requires an opportunistic case finding for assessment of risk factors, an early detection of disease, the identification of high risk status, a combination of pharmacological and psychosocial interventions and a long-term follow-up with regular monitoring and promotion of adherence to treatment that requires a comprehensive approach based in PHC [10].

El Salvador, located in Central America, has a population of around 6.3 million inhabitants (data for 2016) [11] of which 61.7% of the population live in urban areas, while 23,7%, almost one third of the Salvadoran population, live abroad. The median age is 26 years, and the life expectancy is 69 years for men and 78 for women. El Salvador is considered a country with a fragility context as it has one of the highest homicide rates in the world [12]. Young people are the most affected by violence, the war between gangs (“*maras”*) remains a serious social problem, leading to extensive displacement and thousands of deaths every year. The UN has classified El Salvador as one of the deadliest countries in the world outside of a war zone. NCDs are a leading cause of early death among the adult Salvadoran population [13]. During the 2011–2015 period, cardiovascular diseases accounted for 12.0% of the total deaths registered nationwide, followed by chronic kidney disease with 6.3%, cancer with 5.4%, and diabetes mellitus with 3,0% [14]. For 2015, the premature mortality rate due to cardiovascular diseases was 59.3 per 100.000 inhabitants.

In 2009 a Comprehensive Health System reform [15] was started in this Latin-American country based on the Comprehensive Primary Health Care (CPHC) principles and values: the right to health with equity and solidarity, through quality health care that is free at the point of delivery [16]. In over 10 years (2009–2019) the public health expenditure of El Salvador increased 33.7% [17], 67% of this amount being spent by the public health sector (compared to 59% in 2007) and 33% by the private sector (of which 27% was out of pocket expenditure, and 6% through private insurers). Between 2008 and 2013, the country’s income-poverty rate decreased from 46.4 to 34.8%, and extreme poverty dropped from 15.4 to 9.1%. Similarly, El Salvador’s Gini coefficient (0 less inequality-1 more inequality) improved from 0.47 in 2009 to 0.41 in 2013 [18] and the Human Development Index (0–1) moved from 0,529 in 1990 to 0,666 in 2013 [19].

The Comprehensive Primary Health Care strategy [20] (CPHC) implies that the ‘Primary Health Care’ concept is not limited to ‘well organized first line services’. It refers to an integrating approach for the whole national health system. First line health coverage has expanded by doubling the number of health units. A special focus was put on increasing coverage in the poorest municipalities. The community level of PHC networks are health units where community teams effectively bring healthcare closer to remote populations. The budget for PHC increased from 140,18 USD millions in 2008 to 256,73 USD millions in 2018.

Although a large amount of quantitative data has been recorded by the Salvadoran Ministry of Health (MoH) and others since the implementation of the Comprehensive Health Reform in El Salvador, to date there has been no qualitative study of these development providing fan in depth understanding of the reform process.

The study was focused on the NCDs management in PHC. Therefore, a case study method was applied to explore the functioning of the network model of the Salvadoran PHC, its comprehensive approach in the management of NCDs in PHC settings, as well as the role of social participation to strengthen NCDs services.

This work relates to other Global Health research from the NIHR Research Unit on Health in Fragility (RUHF), at the Institute for Global Health and Development of the Queen Margaret University in Edinburgh, emphasizing strengthening the provision of quality care for Non-Communicable Diseases and mental health in fragile settings: Syrian refugees and host communities in Lebanon, fragile communities in Sierra Leone, and El Salvador.

## Methods

### Data collection & sampling

The case study gathered information through a qualitative assessment consisting of semi structured interviews and official document reviews (mostly from the Salvadoran MoH as well as international health organizations as PAHO and WHO). Both interviews and document review explored three questions:1. How is the PHC organized in El Salvador? 2. What is the current NCDs approach in PHC? 3. How is community participation playing a role in PHC for NCDs prevention and care?

The objective of this analysis was to contrast and complete the information provided in the interviews and find new data that will contribute to the fulfilment of the objectives of the implementation of CPHC and its approach to the NCDs. A case study is an appropriate methodology when a holistic, in-depth investigation is needed and is designed to bring out the details from the viewpoint of the participants by using multiple sources of data [21]. By drawing upon both quantitative and qualitative data, case study methodology helps to explain both the process and outcome of a phenomenon within its real-life context [22]. In this empirical inquiry the contemporary phenomenon investigated was the development and organization of CPHC in El Salvador since 2009, particularly regarding NCD management, considering the voice of chronic patients and PHC providers’ experiences and assessing official documents such as the *“National multi-sectorial strategic plan for a comprehensive approach to NCDs*” [23] .

We conducted 26 interviews using a semi-structured interview guide to 14 chronic care patients and 12 PHC staff members as shown on Table 1*.* Purposive and snowball sampling methods were used to recruit participants. Staff members and patients were initially identified by the coordinators of the health units and interviewed at their place of choice, either in the workplace or domicile. Interviews took around an hour and participants were free to stop the interview at any time.

**Table 1:** Chronic patients interviewed and Staff member interviewed working at different levels of PHC

Chronic Patients	NCD suffered	Sex	Age	Marital status	Children	Level of education	Occupation
1.	Hypertension	F	44	Accompained	6	Primary education (until 8 years old)	Homemaker
2.	Hypertension	F	77	Married	6 (2 died)	Primary education	Seamstress
3.	DM type 2	F	57	Married	12 (5 died)	Illiterate	Street food vendor
4.	Hypertension	F	70	Married	6	–	Homemaker
5.	Hypertension	M	77	Widow	6	Primary education	Farmer
6.	DM type 2	F	62	Widow	–	Basic (1 year)	Domestic worker
7.	Hypertension + CKD	F	49	Separated	4	Primary education	Homemaker
8.	Hypertension	F	70	Accompained	8 (2 died)	Primary education	Homemaker
9.	DM type 2	F	61	Married	3	Illiterate	Homemaker
10.	CKD	M	58	Married	6	Primary education	Farmer
11.	Hypertension	M	80	Married	4	Illiterate	Farmer
12.	Hypertension + CKD	M	66	Accompained	6 (2 died)	Illiterate	Farmer
13.	Hypertension + CKD	F	61	Married	3	Illiterate	Homemaker
14.	Hypertension	F	64	Married	7	Primary education	Cook
PHC staff				Professional profile			Sex
Coordination level				1.Departamental Coordinator			Male (M)
				2.&3.Intermunicipal coordinators			M
				4.Regional coordinator			M
Interdisciplinary PHC team				5. Health educator			Female (F)
				6.Sanitary inspector			F
				7.Medical student completing year of social service			M
				8.General practitioner			M
				9.Family doctor			F
				10.Nurses			F
				11.Pharmacist			F
				12.Laboratory technician			M

Patient inclusion criteria was being over 35 years of age, living with an NCD, and receiving care from the PHC units. The age group was determined based on the WHO-defined ‘higher risk group’ for NCDs [24]. The 14 NCD patients interviewed suffered from Hypertension, Diabetes Mellitus type 2 (DM 2) and/or Chronic Kidney Disease (CKD).

Staff member interviews were included to incorporate a comprehensive understanding of the PHC system by presenting the providers’ perspective of NCD care. Health professionals working in different levels within PHC (inter-municipal, departmental and regional level) were selected to participate. The study included staff of basic, intermediate and specialized community health units, seeking to collect a variety of viewpoints and experiences. Inclusion criteria for staff members interviewed were to be actively working at some level of the PHC system at the time of this research and having worked at least for a year in PHC since the health reform started in 2009*.*

The interviews took place between March and August 2018 in three different departments of El Salvador: Chalatenango, Morazán and Bajo Lempa. Acknowledging that El Salvador has 14 departments, the aim of this case study was to see some representative pattern in the management of NCDs in PHC more than trying to generalize the data for the whole population or making and in-depth evaluation of the PHC system itself. Our interest was to closely investigate contemporary real life of people living with NCDs in El Salvador and how the implementation of PHC has had an impact on its management as well as on the care offered by staff members of PHC. The three departments were selected considering socio economic, historical and geographical disparities between them (with special focus in reaching remote areas), to represent variation in size of health infrastructure, population served and prevalence of chronic conditions. The selection of departments was also influenced by security factors during fieldwork and willingness of the PHC care team to participate in the research. Access to data was facilitated by members of the MoH, social movement members (NHF), and medical staff from the health facilities where data were collected.

### Data analysis

All interviews were conducted by the primary researchers in their mother tongue (Spanish) and were digitally recorded. For data analysis qualitative information collected was transcribed. After transcribing, audio recordings were permanently deleted. Then all qualitative data were coded, both manually and then using Digital coding with NVivo® version 10.

A thematic framework [25] method was applied for the analysis. A non-pure deductive approach was used to develop an open coding system around the topics based on the content and context of patient/provider interactions, work practices and patient/provider experiences of care. After grouping codes into potential themes, a purposive thematic framework was developed through the application of a reflexive approach and the triangulation of data [26] through different meetings (personal or virtual) of the research team to identify consistencies or variations across the dataset.

### Ethical considerations

The study was approved by the Salvadoran Ethical Committee (CNEIS/2018/005_A) and by the Queen Margaret University (QMU) Research Ethics Panel. Prior to each interview, an information sheet was given and explained to each participant, after which they were invited to sign an informed consent form. Interviews were recorded using a digital voice recorder. All data involving participants were made anonymous at the start of data collection. Participation in the research project was voluntary and no incentives were given.

## Results

Findings are presented through quotes from interviews with PHC workers and chronic patients. The thematic framework (see Table 2) was structured in 3 main themes: The Salvadoran PHC system is firstly described based on the perception of the stakeholders and documents reviewed emphasizing its main characteristics. Secondly, the specific management of NCDs in PHC was assessed in terms of policies implemented, prevention and health promotion strategies for NCDs and longitudinal care. Finally, the community perspectives and the potential of social participation to strengthen NCD care is presented. The perception of changes since the implementation of the health reform in 2009 is a crosscutting theme reflected in most of the codes.

**Table 2:** Coding and thematic framework

Themes	PHC Organisation in El Salvador	NCD Management in PHC	Social Participation in PHC
CODES	Integrated comprehensive health networks Community health networks and interdisciplinary PHC teams Coordination between levels of care Intersectoral participation and health in all policies Accessibility Quality of care	Health policies for the management of NCDs Prevention of NCDs and health promotion Longitudinal care of NCDs	National Health Forum Accountability Right to Health offices Community-based peer support groups for NCDs

Regarding the background of the chronic patients interviewed for this case study, their median age was 64 years old. Most of the participants had a low level of education achieving primary education until the age of 8 and were able to read and write. Regarding their marital status, most of the participants were married or accompanied and those who weren’t were mainly widow women whose husband was either killed during the war or through social violence. The participants had an average of 6 children, and some have lost children before the age of one (infant mortality has decreased from 21,52 in 2009 to 16,8 in 2017 (https://www.indexmundi.com/g/g.aspx?c=es&v=29&l=es)). Most of the participants had sons or daughters living abroad (mostly in the USA); family remittances benefit more than 20% of households in El Salvador. The chronic patients interviewed also mentioned their grandchildren with great concern as some of them have been killed or threatened by the social violence. In terms of occupation, men are more likely to work in agriculture, a fact that is related with the high prevalence of *chronic interstitial nephritis of agricultural communities* (CINAC) linked to pesticide intoxication among these workers [27]. Women interviewed worked in relation to care (inside and outside home). Chronic patient interviews covered aspects of their experience receiving care at the PHC facility and included questions on treatment initiation, care and support, communication with providers and quality of care as well as their perception of change in the management of their NCD after the implementation of the health reform and role of social participation. One of those patients was attending to community-based peer support groups for NCDs. In relation to the health workers interviewed, those working at the coordinator level were all men whereas in the interdisciplinary PHC team 5 out of 8 are women. The most recent MoH data shows that the staff members working in the NHS are 61.9% women and 38.1% men [17].

### Primary health care organization in El Salvador

#### Integrated and comprehensive health care networks

El Salvador’s PHC system is organized through ‘integrated and comprehensive health networks’. Their objective is to provide equitable, comprehensive and integrated health services to individuals and communities, for a well-defined population, within a specific geographic area. This organization has contributed to have a better follow-up of the epidemiological situation for each territory

*“Epidemiological surveillance, also of NCDs, is ensured, and every Community Healthcare Unit has identified its chronic patients for follow-up and medication. Data on drug supply are included in this surveillance. In micro-network meetings these health data are used to prioritize health services at municipal level”-* Intermunicipal coordinator.

*“We have the ‘Integrated Health Service Delivery Networks (IHSDNs) health indicators’ which include NCD data. The departmental level analyses the epidemiological surveillance data of all micro networks of the department. Following this, the coordinator shares them in the regional meeting which is helping to organize better health services in PHC”* - Regional Coordinator.

**Fig. 1:**
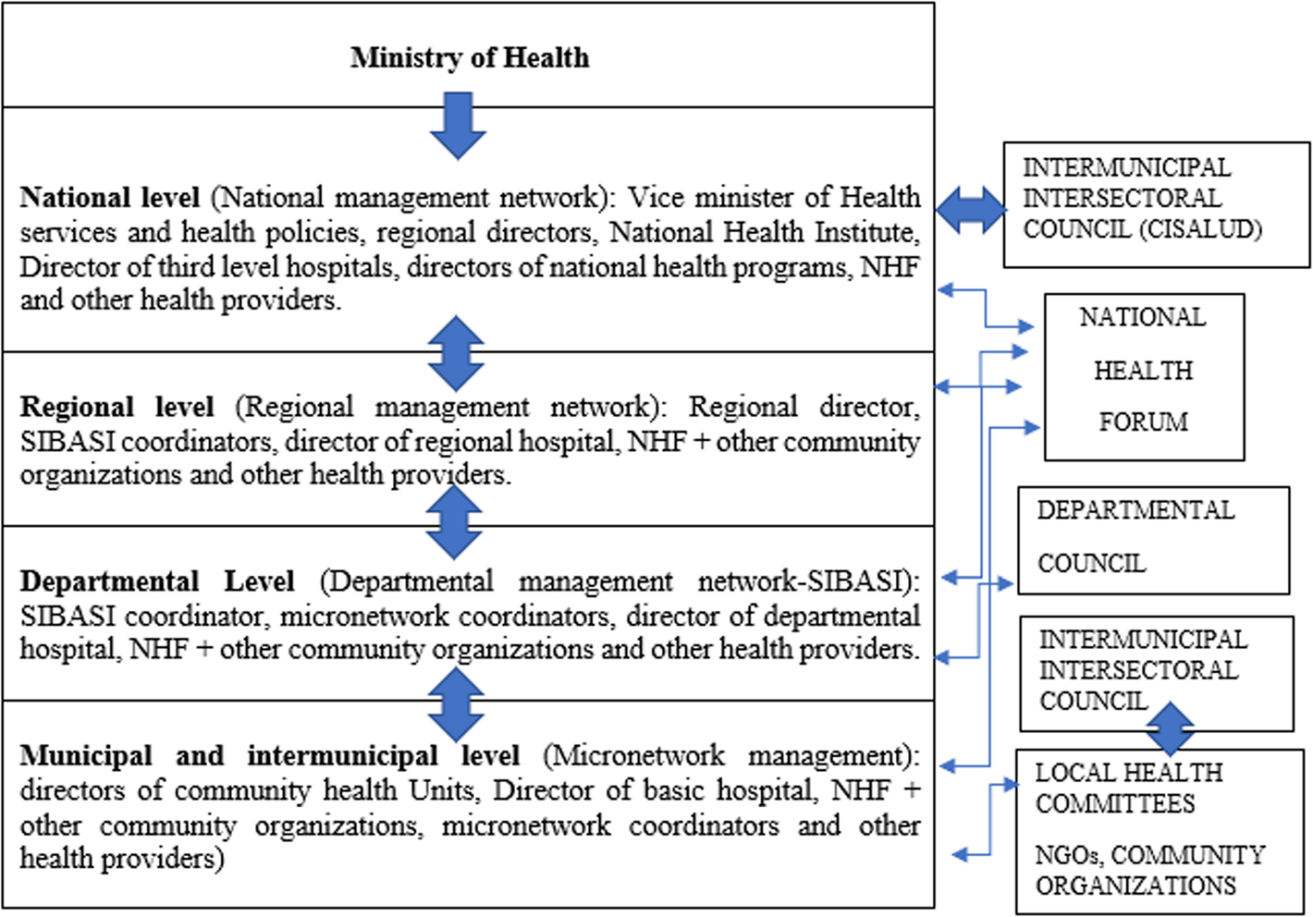
Integrated and comprehensive health networks and management councils (left section), social participation spaces and intersectoral participation (right section)

Different levels of decision-making exist at municipal, departmental, regional and national level (Fig. 1). The departmental level, called the Basic Integrated Health System (SIBASI by its Spanish acronym, *Sistema Básico de Salud Integral*), is the basic operational structure of the NHS where the Primary Health Care system is articulated with the other levels of care, and with community organizations. These meetings facilitate the sharing and dissemination of information and the coordination of activities to ensure a comprehensive care under the equity scope.

*“As a Basic Integrated Health System we hold meetings every 2 weeks in order to monitor acute and chronic diseases and we evaluate health indicators (mortality and morbidity rate within the region, etc.). Depending on the number of detected cases, we decide which activities should be prioritized*” - Departmental coordinator

One of the main purposes of these networks is the management and provision of health services to ensure a continuity of care: promotion, prevention, diagnosis and treatment, as well as in disease management, rehabilitation and palliative care. The coordination within the different levels of the integrated and comprehensive health networks helps to optimize resources, for example during medicine shortages.

*“We have good coordination between the micro-networks. If some medication is missing, we coordinate it in order to avoid drugs shortage.”-* Intermunicipal coordinator.

*“If for example there isn’t a specific medication in the region, we put them in contact with other places where there is enough supply and we try to avoid through our ‘integrated and comprehensive health* networks’ *the medicine shortage”-* Regional coordinator.

#### Community health networks & interdisciplinarity

The Comprehensive Primary Health care in El Salvador is organized in Family Health Community Units (UCSF by its Spanish acronym- *Unidades Comunitarias de Salud Familiar).* These are the facilities of the First Level of Care, where integral health services of different complexity are provided and they are classified as basic, intermediate or specialized Family Health Community Units. In El Salvador there are two types of PHC teams [28]: The aim of having two type of teams is on one hand to improve access in remote areas including specialized care, and in the other to provide care at the family level rather than individual level.

-Community Family Health Teams (Family ECOS, by its Spanish acronym *Equipos Comunitarios de Salud*): composed of a medical doctor, a nurse, a nursing assistant, three health promoters, and a polyvalent staff member. They work in basic and intermediate Family Health Community Units. In rural areas of ​​small municipalities with dispersed population, the family ECOS has an approximate population of 600 families under its scope of responsibility. In ​​large urban areas, Family ECOS have 1800 families within their scope of responsibility.

-Community health teams with specialities (Specialized ECOS): comprising a family doctor, a paediatrician, an obstetrician/gynaecologist, a psychologist, three dentists, a health educator, a sanitary inspector, a statistical assistant, a physiotherapist and two laboratory technicians. The specialized ECOS have approximate 6000 families, 30,000 people under its scope in rural areas and in average 9000 families, 42,000 people in urban area.

Each Family ECOS works in coordination with its corresponding Specialized ECO, integrating a networking, being responsible for the health of the ascribed population, incorporating the needs identified by the population itself in order to be responsive.

*“We work in a network, that means that each Community Family Health teams are connected with the Community health team with specialized within the area as well as with community organizations in order to properly approach the real needs of the population”-* General practitioner (GP).

The Family and specialized ECOS are intended to know the reality of their environment, identify with the community and, with the help of community leaders and where there is presence of the National Health Forum, consider the dynamics of the social determinants of the health of the population. This is done by analysing the health situation of the persons by traditional biological risk factors and identifying vulnerabilities of their families as well as considering social risks (violence, unemployment, etc.). Their purpose is to ensure the adequate follow-up of the health of their population of responsibility.

*“My work includes three types of populations: the primary population is the patient with a chronic disease. The secondary population is the family of the person, and the tertiary population is the community as a whole.”* - Health Educator.

Interdisciplinary PHC teams help to address health in a comprehensive way, addressing both preventive and curative health care. It also promotes health infrastructure development and community involvement, thereby promoting sustainable improvements of community health.

*“We work as a team, even if each of us have specific duties, we try to give a comprehensive care working together with the rest of the PHC team”-* Sanitary inspector.

Community health promoters play a key role in the coordination between patients and PHC staff. They are the actors in contact with patients in the community and who inform the rest of the PHC team about particular situations considering the fragile context of the country due to violence. They discuss an intervention plan or organize appointments to make sure that patients can be attended by a specialist and inform health staff when complications arise. Health promoters also identified patients that have never had contact with the health system before in remote areas and are in charge, together with the rest of the PHC team, of community health prevention and health promotion. In this sense, an essential strategy of the 2009 health reform has been fulfilled: the strengthening of the health promoter as an essential element at the first level of care.

*“Our ‘community eyes’ are the health promoters. They identify patients who have some risk factor”* - Regional Director.

*“Then the health promoters say, ‘Come doctor, I want you to accompany me to the house of a diabetic patient.’ And we go to the patient’s house, where they are given counselling, and where we also see what the patient’s living environment is like” -* GP.

Due to the high burden of Chronic Interstitial Nephritis of Agricultural Communities (CINAC) in El Salvador [29], in specific areas where this chronic disease is highly prevalent a nephrologist is part of the PHC team.

*“The nephrologist is oversaturated with renal patients, at the first level of care. Even if he tries to give high-quality attention, it is difficult to do so with such a heavy workload. He also works at the tertiary level of care in Jiquilisco’s hospital on Mondays and Fridays*”- Intermunicipal Coordinator.

#### Coordination between levels of care

Some of the participants interviewed pointed out that it can take months before a referred patient is seen at the hospital. Moreover, when patients had an appointment at the 2nd or 3rd level of care, they rarely return to the first line services for follow-up. Probably some hospital health staff still do not trust the strengthened primary care services in their follow-up of health problems. As a result, hospitals remain saturated with patients having to travel long distances for routine follow-up.

*“From the time I make the referral at the first level, it takes about 4-6 months for a patient to make an appointment with a nephrologist or another hospital specialists” -* Family doctor.

*“When hospitals diagnose a chronic patient, they sometimes do not inform the first level of care. Some hospitals have not yet understood the health reform in which PHC is the system’s frontline, where patient follow-ups can perfectly be conducted. Instead of removing the burden of so many patients being treated at the hospital, they do not make referrals because they think first line personnel has not enough training ... Finally, the most vulnerable people are the most affected as they cannot afford the transport to reach the hospital*” - Regional Coordinator.

#### Intersectoral participation and health in all policies

The strengthening of intersectoral approaches constitutes a strategic axis of the health reform, aimed at focussing on the social determination of health. At the national level, the Intersectoral Commission of Health (CISALUD, by its Spanish acronym Comisión Intersectorial de Salud) brings together more than 40 public, autonomous, private, and grassroots institutions to address health issues and considering the social determinants of health. This intersectoral approach responds to the Health in all policies premise, in which participation involves not just health but other sectors for local development. The strategic role acquired by CISALUD and the departmental councils, covers the identification, analysis and proposal of joint strategic lines and activities for occupational health and safety, health promotion and the prevention of diseases, integrated vector management and pregnancy in childhood and adolescence, besides promoting territorial management spaces. Departmental health councils are established as instruments for the participation of health professionals, trade unions, cooperatives and NGOs working together with an intersectoral approach.

“*This intersectoral approach is strategic, as it implies the coordination with other institutions. At local level this intersectoral committee is formed by: directors of community associations, NGOs, school directors, teachers, the police, etc… They meet periodically, and we inform them of difficulties we face, the epidemiological surveillance, etc… to work together and improved health outcomes trough coordination of the different actors*”. - Departmental coordinator.

#### Accessibility

Accessibility is defined as the possibility for individuals to receive the necessary medical assistance for their health problems, without geographical, economic or cultural barriers [30]. Accessibility can be understood as a condition of equity and as a component of the quality of care in general [31]. Since the health reform began, the dominant discourse of the patients interviewed refers to improvements related to free access to care - including the abolition of consultation fees and the overall reduction of out-of-pocket payments - better access to health care units, and to medicines and laboratory tests. In this sense, the health reform abolished consultation fees, known as ‘voluntary fees’, within the health system. This substantially reduced the out-of-pocket costs (while indirect costs, e.g. for transport, and some laboratory tests remain). Patients perceived this as facilitating access to care.

*“The abolition of voluntary consultation fee has helped us to have more access to the health system, because before the reform, we needed money to be able to pay the consultation”-* Man with hypertension.

*“I no longer have to pay the ‘voluntary fee’ every time I go to the doctor. That has been a great change for me and my family”-* Woman with Diabetes Mellitus type 2.

According to the last accountability report published by the MoH of El Salvador in 2018 [32] there are currently 753 Family Health Community Units, 577 Family ECOs and 39 Specialized ECOS all over the country focusing to increase access in the poorest municipalities.

*“When the health reform started, we started to focus on reaching population groups that never came to the health units. This is what I do, apart from working with the community” -* Sanitary inspector.

*“Before 2009 I had to take the bus to reach the health unit, but they opened a new one close to my house and l normally come walking now” -* Woman with hypertension.

*“I take a moto-taxi to reach the health unit that is close to where I live. Before the health reform every time I was sick due to my illness, I had to go to the hospital that it is far away from here and I couldn’t afford a moto-taxi until there” -* Man with Chronic Kidney Disease.

Nevertheless, some geographical limitations persist, due to the lack of public transport and the limited access to an ambulance (with gasoline) in case of emergency.

*“Transport is a weakness that we have as a specialized Community Health Unit, as some people come from remote areas and there is no adequate public transport for them”.* - Intermunicipal Coordinator.

The specialized PHC team travels twice per week to basic and intermediate Family Health Community Units in order to facilitate access to specialists for those living in remotes areas. With programmed community visits of the specialized CHT they ensure monthly visits to every basic and intermediate Family Health Community Units belonging to a specific geographic area. Nevertheless, the specialized team also express difficulties in reaching remote areas.

*“We conformed a specialized CHT, and we visit each basic and intermediate unit at least once per month. Chronic patients for the family doctor, children for the paediatrician...” -* Family doctor.

*“There are very remote areas that in the rainy season are flooded. Despite the geographic difficulties in accessing these sites, we also try to reach these places.”-* Health educator.

*“Even though the health unit is closer to my house, I had an emergency once and the nurse came to inject me at home”-* Woman with Diabetes mellitus type 2.

*“We organize home visits where the whole interdisciplinary team goes to see a patient with special needs. The doctor, nutritionist, psychologist, physiotherapist, health educator and nursing, we all go”-* Health educator.

Concerning the access to medication and laboratory tests, the main discourse of the chronic patients interviewed reflected the perception that access to free medicines is provided in PHC Units. But participants also commented on shortages in some Family Health Community Units.

*“Access to medicines is much better now than before because we used to pay for all of them and now the government pays all that...” -* Woman with hypertension*.*

*“They give us treatment in the health unit pharmacy where we get our prescriptions, but sometimes there is not enough medication” -* Woman with hypertension.

Although the health reform has facilitated the transport of laboratory tests from basic health units to health units with laboratories, some barriers still exist with transport due to long distances from and to remote areas. Inevitably, some laboratory tests cannot be realized at the first level, and the patients have to go to the hospital or to the private sector.

*“In basic health units, there is a specific day of the week when lab samples are taken by the nurse, and a polyvalent person from the staff brings the sample to the lab (...) There are specific laboratory tests that I have to send for analysis to private laboratories”. -*Laboratory Technician.

Finally, in terms of accessibility to the health professionals, even though the number of staff members working in PHC has increased in 6000 more workplaces since 2009 to 2015, the dominant perception of the stakeholders is that more work force is needed in PHC.

*“It would be nice to have more health personnel in the Family health Community Units, especially health promoters as they are the ones often coming for home visits”.* Woman with Diabetes Mellitus type 2.

*“Even if the number of workforce has increased a lot since the beginning of the health reform, it is important to keep investing in human resource in order to assure quality and access” -* Regional Coordinator.

#### Quality of care

Some interview items regarding the quality of care in PHC included in the interviews pointed to how patients felt they were treated by health workers or to the quality of the information received in PHC Units.

The results show what participants understand by quality of care [33] and were summarized along the following dimensions (see Table 3 below*):* quality of care by health workers, waiting time, attention time in consultation, health workers’ communication skills (including, the adaptation of language to the patients’ educational and socio-economic backgrounds), information and mechanisms to make suggestions and complaints as a PHC user. The dominant discourse of chronic patients interviewed was a positive perception regarding the quality of care in PHC.

**Table 3:** Perception of quality of care in PHC

Quality of care	Conclusions	Illustrative stakeholder’s quotes
Quality of care provided by health workers in PHC	Chronic patients mainly expressed having received adequate care.	*“In the health unit, I have always felt well treated. The nurses are very kind to me. To be honest I cannot complain; they have all been very nice every time”* Woman with diabetes. *“Here, in the new health unit, I feel well-treated (...) The nurses and the doctor are nice to me”* Man with CKD.
Waiting time	Waiting time is shorter in PHC than in hospitals. In PHC, the waiting time for specialist consultants who attend patients by appointment was shorter than the GP’s (waking patients)".	*“Before the health reform every time I had a problem because of my blood sugar was too high or down I had to go to the hospital and wait for hours, now I just walk to my nearest health Unit” Woman with Diabetes.* *“When you go to see the general practitioner, they give you a number at the entrance of the health unit and then attend in numerical order. When the specialist comes, I have my scheduled appointment from the previous time that I was seen”* Man with Diabetes.
Attention time in consultation	Health workers take the necessary time. Family doctors have 15 min per patient.	*“To be honest, in the health unit they have always given me the time I needed in the consultation”* Woman with hypertension. *“The standard is 15 min per patient, 4 patients per hour, but if for example a patient (...) needs more time, we make sure we give them the time they need”* Family Doctor.
Health workers’ communication skills	Communication is adequately adapted to the educational level of individual patients, while also engaging with the chronic patients’ families (especially cases where the patient may be illiterate or advanced in age).	*“I speak to them with words that are easily understandable, not with technical words. I adapt my language to the educational level of each patient”* Pharmacist. *“I explain to the patient and their family, especially when I am dealing with older patients, all the details about the treatment and. In cases when patients are illiterate, I draw signs to help them to know when to take the pills. I also ask them to repeat to me what I have just said in order to verify that they have understood”* Family doctor*.*
Health information quality	The majority of the discourse of the interviewed chronic patients responded positively when asked about the quality of the information given to them by the health staff in PHC.	*“The information I received was good because they explained what my disease was and how to take the medicine. Everything is fine. They always try to assure us that we understand everything correctly”* Man with Diabetes. *“The doctor gives us information about why we have to do lab tests, how to take the medication, information about diet and so on”* Woman with Diabetes.

### NCD management in primary health care

Secondly, interview questions were focused to explore the management of NCDs in PHC. The different policies for NCDs were analysed through the participants’ perspectives as well as the prevention and health promotion programmes and the longitudinal continuity of care of chronic patients in PHC.

#### Health policies for the management of NCDs

Interviews with staff members and the analysis of official documents from the MoH revealed that the following policies were of support in designing an approach to NCDs (Table 4):

**Table 4:** Health policies for the management of NCDs in PHC

Knowledge and capacity building in NCDs *-*National Training Plan	*“The ministry has been giving us training in noncommunicable diseases. In 2012, they gave us a complete course, which was specifically about diabetes, high blood pressure ...” *Health Educator.
Having specific protocols and intervention plans	*“The protocols are created by the National Institute of Health. As ECOS we have protocols for both the prevention and treatment of NCDs. They existed before the 2017 guidelines, but have been evaluate. They are already on the third validation”* Family doctor.
Coordination throughout the healthcare system	*“In the integrated and comprehensive health networks, networking has improved communication between the first level and the hospital”.* Departmental coordinator.
Intersectoral participation	*“In the intersectoral participate the mayor’s office, the church, some community leaders, the health unit, the police, civil protection services, the house of culture and, education”* Intermunicipal coordinator.
Interventions focused on prevention and health promotion	*“Informative workshops are given, community-based, peer support groups for NCDs are formed, and we also use the mass media to inform the population” GP.*
More medication and medical supplies	*“Medication supplies have been improved, glucometers too, now that the Ministry provides them. There are also more lab tests available”* Health educator.
Better prioritization of resources	*“We can only evaluate and prioritize certain health programs and resources”* Pharmacist.
Community engagement	*“The work of the NHF is complementary, because we do the healing part and they do the awareness part, with social participation, with promotion and accountability”* Health educator.
Medical specialists at the community level	*“Community approaches of the specialized team go once a month to each unit and have an annual scheduling”.* Intermunicipal coordinator.
Epidemiological surveillance of NCDs -Morbidity and mortality database (SIMMOW) -Map of health inequalities	*“The beginning of the epidemiological surveillance of NCDs has been carried out for a year now. Each Eco has identified its chronic patients for follow-up and medication. In addition, data on drug supply are also included”* Intermunicipal coordinator.

#### Prevention of NCDs and health promotion

In relation to health education and health promotion, the implementation of the CPHC has provoked a shift from an assistance care model to a preventive model that fosters a community perspective. However, some PHC staff mentioned that more prevention and promotion efforts must be done.

*“The NCDs strategy based in PHC is aimed at the prevention and health promotion approach that will avoid many deaths emphasizing they quality of life of chronic patients while also preventing others from getting sick”* – Intermunicipal coordinator.

*“Children and adolescents are not aware of the NCDs as they see it as something far away, in order to improve awareness, especially among young population, the use of mass media and health promotion activities in schools can be very useful”* – Health Educator

During the interviews, several preventive actions for NCDs that have been implemented in PHC were mentioned (see Table 5 below).

**Table 5:** Prevention of NCDs in PHC

Primary prevention	
Biological and environmental Risk Maps.	*“The health promoters make the identification of medical, environmental and social risk factors for every individual and family within same health unit, establishing a ranking of high, medium and low risk, drawing a risk map (…) home visits of the PHC team are scheduled according to risk”* Departmental Coordinator.
Comprehensive diet programs.	*“After detecting that a person has an unhealthy diet, not only the nutritional part is evaluated. We also visit their homes and detect the economic and geographic barriers that may limited them from accessing healthy food and from adapting our recommendations to each context”.* Nurse.
“Healthy passport, Exercise is medicine” program.	*“A strategy called ‘Healthy passport’ has been implemented in PHC leaded by the National institute of Sports (INDES), which is specifically for non-communicable diseases. It is a card that targets what we call ‘exercise is medicine’, where patients are given a prescription of exercises, not just medications. The specialist doctor gives them a prescription of exercises, and then the nurse explains how to do those exercises and fill out the healthy passport”* Health Educator.
Screening programs for Chronic Kidney Disease (CKD).	*“One of the proposals that was driven from this health unit was the screening of people with traditional and non-traditional risk factors for CKD (mostly farmers working with agrochemicals)”* Sanitary inspector.
Information talks of the Specialized ECOS with special focus in NCDs.	*“We organized informative talks in every health unit that we visited weekly before the consultation, which are already organized in a chronogram. We inform about chronic diseases, another day about vaccination, gender violence,* etc. *We are an interdisciplinary team, and we all attend to this information talks and it takes around 15–20 min. We always give those talks because it is part of the primary care that is provided, of the integral approach”* Family doctor
Secondary prevention	
Community-based peer groups for people living with an NCD	*“The self-help groups are conformed by chronic patients talking about their own experience with diseases, how they have overcome, or how they feel, and there is self-development. We firstly give a small information talk, and then they share about their chronic disease”* Health Educator. *“Regarding the educational part of NCD in PHC, the IHSDNs indicators include how many ECOs have self-help groups for NCDs and which ones still don’t have them in order to encourage them to start conforming them with chronic patients”* Regional Director.
Terciary prevention	
Prevention of complication of Diabetes	*“We also check diabetic patients’ feet and explain what the shoe should be like and the hygienic measures to prevent complications”* Nurse.
Prevention of complication of CKD.	*“There are people living with a kidney damage that is maintained, that does not reach dialysis, and that is because they understand the need to be treated by the psychologist and the nutritionist, because nutrition and good hydration is basic, and also the guidance of the psychologist”* Sanitary inspector.

#### Longitudinal care of NCDs in PHC

Longitudinal care allows the continuous evaluation of progress as well as the impact of the disease and disability on the chronic patient’s daily life. Attention is better adapted to specific needs and tends to show a greater capacity to respond to recommendations [34]. The Salvadorian PHC system seeks to improve the quality of care and to have better health outcomes, therefore improving longitudinal continuity of care. There is evidence of better preventive care, better use of health services, more appropriate and timely assistance, fewer preventable diseases, fewer hospitalizations, better compliance with prescribed pharmacological treatment and lower total costs among those patients treated by their family doctor/general practitioner over time [31].

In the table below (see Table 6), the main pathways of care for an NCD in PHC [diagnosis, follow up and complications] are shown, as well as the main conclusions after the analysis of the interviews and an illustrative quote of each of them.

**Table 6:**
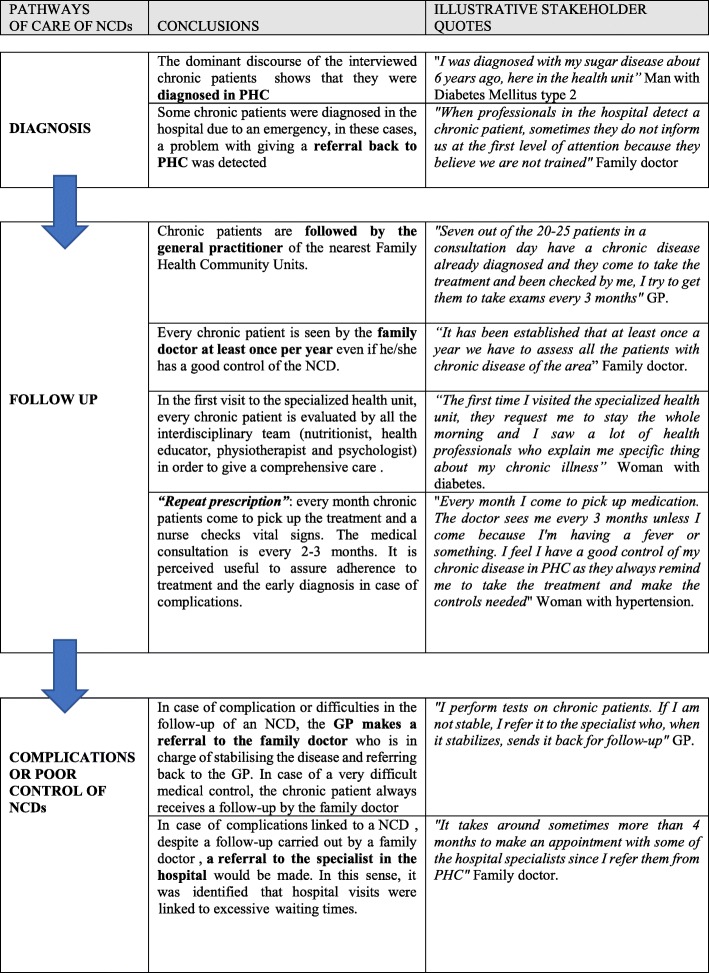
Pathways to care of NCDs in PHC

### Social participation in PHC

The Ministry of Health of El Salvador made a political and strategic commitment to create comprehensive PHC with a community orientation. For this reason, the fourth strategic axis of the Health Reform is social and community engagement through the NHF. This strategy aims to strengthen community organization and social participation, so that society is empowered and can fully exercise its right to health. The NHF is the autonomous social health movement with presence in most of the country and that coordinates with the MoH for achieving the right to health through community participation [35]. The NHF has established citizen participation mechanisms that guarantee social participation in the design, execution and evaluation of health policies that help to create policies based on the real needs of the population. The NHF opened spaces for making health decisions where people from the community can actively participate in addressing those obstacles that hinder better attention to health problems. These health committees of the NHF are composed of PHC staff members, coordinators of the Family Health Community Units and civil society actors organized through the NHF.

*“Community participation makes us see things that we believe we are doing well but that are not being perceived as such within the community. The participation of the NHF in the local committees, micro network and other spaces of decision-making, help us to improve particular situations or coordinate in order to provide additional support” -*Departmental Coordinator.

The NHF pursues civil society consensus in strategic decision-making, through a broad process of citizen participation. The integrated and comprehensive health networks also strengthen community capacity towards becoming active partners in the governance and performance evaluation of the network. Actions - not only related to the provision of care, but also towards preventive activities - are coordinated with community networks. Community organizations participate actively, mainly through the NHF, thereby serving as a bridge between communities and the health system. It fulfils the role of social auditing within the health system, for which it developed parallel structures with the health systems structures (see Fig. 1). The NHF is organized at municipal, departmental and national level, and has local committees in most of the country that work actively with the MoH. This strategy ensures a type of ‘co-responsibility’ between the Salvadoran civil society and the National Health System.

Through these spaces of community participation, PHC personnel can create mechanisms based on the real needs of the population, considering their socio-political and cultural context in order to develop a better approach by the co-dynamization of community processes.

*“In these participation spaces for health, the community can request specific initiatives for improving the management of chronic diseases that is finally considered in the departmental network”-* Regional coordinator.

The NHF also developed an accountability system. *“Offices for the right to health*” exist in the main hospitals across the country, where community members can claim their rights. With the support of the MoH, suggestion boxes are placed in every health unit and hospital. Complaints or suggestions are assessed by the facility management in presence of the NHF representatives.

In relation to the community-based peer support groups for NCDs, the main discourse of interviewed health staff members and chronic patients showed that these groups provide space for sharing and learning about the reality of living with a chronic disease. Even though the groups are currently coordinated from the primary health care units, the final objective is to have self-managed groups from the community. These community-based peer support groups for NCDs could improve chronic patients’ quality of life while also improving the prevention on NCDs through forms of social engagement that exist in coordination with PHC.

*“For the daily support for people with a chronic disease like me, it is nice that we all get together in these groups where we learn more about our disease. It allows us to give each other advice and to learn to survive in the best way with a specific chronic health problem...”-* Woman with hypertension attending a community-based peer support group for NCDs.

*“There are many factors / obstacles in our lives that interfere with leading a healthy life, but the objective of this group is that you know how to accept and recognize your own chronic diseases, how to control them and to know better what to do when there is some complication” -* Health Educator coordinating a community-based peer support group for NCDs.

## Discussion

This case study has enabled an in depth understanding of how CPHC is organized in El Salvador and how its implementation is facilitating NCD management with a strong social participation and accountability.

Regarding the organization of CPHC, the dominant discourse of the stakeholders interviewed was that the implementation of the Integrated Health Services Delivery Networks (IHSDNs) was an improvement in the coordination between different levels of decision making, as well as better follow-up of the epidemiological situation. The Pan American Health Organization considers the development of IHSDNs to be essential to provide equitable, comprehensive, integrated and continuous health services to the population [36]. The Salvadoran health care system networks follow this logic [37] but have gone beyond this point: the networks also include social and community organizations and working groups linked to the NHF. This strategy aims at strengthening the right to health, not only in terms of service provision, but also towards preventive community action. In addition, establishment of the Salvadoran Intersectoral Health Commission (CISALUD) is perceived as a key strategy for improving health outcomes as it improves the coordination of the different stakeholders and follows a Health in all policies [38] approach.

At the first level of care, a CPHC approach has shown to reduce accessibility problems for marginalized and impoverished populations [39]. Strong advances in terms of accessibility have been confirmed not just with the quantitative data reporting improvements in health coverage, but also as perceived by stakeholders.

El Salvador is now closer to achieving Universal Health Coverage (UHC) [40] due to the implementation of CPHC with free access to care (after the abolition of consultation fees and the overall reduction of out-of-pocket payments). As shown in other studies, countries can achieve greater health benefits when strengthening their first level of care [41]. Moreover, WHO’s recent universal health coverage (UHC) monitoring report from 2019 frames PHC as the “programmatic engine for UHC in most contexts” [42] in a variety of ways including community empowerment, social accountability and a multisectoral approach.

However, from this case study some difficulties regarding PHC organization have been identified. The challenges mentioned are similar to the ones assessed in previous studies [43] and some proposals for improvement were formulated by participants. Regarding accessibility, the main problems pertain to the supply system (access to medications and laboratory tests) and geographical access to health services. Participants proposed to simplify the supply system to avoid shortages and to increase public transport services. In relation to coordination between levels of care, stakeholders proposed to reinforce it by improving health system referrals and counter referrals. Finally, it was suggested to further increase the PHC workforce, with special focus on community health promoters. These were considered by interviewees to be a key element in effectively responding to people’s health needs.

In relation to NCD management in PHC, the different policies implemented follow the recommended interventions for the prevention and control of NCDs according to the WHO Global Action Plan of NCDs 2013–2020 [44] adapted to the Salvadorian context. Concerning the prevention measures implemented, the biological and environmental risk maps facilitate the identification and treatment of people at high risk of NCDs or with already established disease [45]. This approach should avert deaths in the short term as it has been identified by WHO as one of the best buys for NCDs [46]. Moreover, these maps also considered the social risk affecting health. The high levels of violence suffered by most of the Salvadoran population imply the need for adapting the counselling given by the PHC staff in relation to lifestyles behaviours in communities (e.g. modifying guidance about doing outdoor exercise).

The CPHC strategy in El Salvador is focused on reducing inequities in the management and provision of health services based on the citizenship taking control of “*the social determination of health*” [47]. This concept [48] is a broad theoretical analysis coming from Latin American critical epidemiology [49] that emphasizes the social structures and their impact on individual and collective health. It develops a critique of empirical-functionalist paradigm of epidemiology and serves as a tool to study the relationship between ways of living, getting sick and dying based on solidarity, sustainability, sovereignty and biosecurity [49]. The social determination of health could be applied to health promotion in NCDs; specific initiatives for managing the risk factors need to be supported by measures that tackle the root causes of inequities [50]. Health behaviours that increase the risk to develop NCDs are greatly influenced by peoples’ environmental, socioeconomic and cultural settings, and are more prevalent among the socially or economically disadvantaged worldwide [51]. Therefore, NCD management and prevention should consider the structural causes of poverty and inequity in health, to adopt effective means of promoting health equity.

Despite all the achievements, stakeholders also called for more prevention and health promotion programmes for NCDs. Some of their suggested recommendations included: to increase the use of mass media to provide information about chronic diseases; to reinforce lifestyle programmes adapted to the sociocultural and economic contexts, with special focus on children and young populations; to promote community-based peer support groups for NCDs; to implement screening programs towards those populations who cannot access health units. This last proposal is especially focused on the prevention and early diagnosis of Chronic Interstitial Nephritis of Agricultural Communities (CINAC). Regarding the continuity of care in chronic patients, the implementation of CPHC has facilitated NCDs diagnosis, follow up and management of any complications through different programs and strategies such as the repetitive prescription. The interdisciplinary PHC teams ensure a holistic approach for chronic patients. However, difficulties in coordination between levels of care were also commonly noted.

Furthermore, our study underlines the importance of a community perspective at its core, based on the principles of social participation, equity and participatory evaluation, which are all essential triggers for high-quality community action [52]. Social engagement has been one of the strengths of the Salvadoran CPHC in contrast to other PHC implementation experience which sometimes have been more focused on issues of efficiency and effectiveness in health care delivery [53] while showing less concern for equity and community participation in PHC [54, 55]. The National Health Forum has developed a strategy that combines empowering community participation with the pursuit of government accountability. The NHF is part of The People’s Health Movement [56], a global movement for the right to health, which argues that achieving health equity will require a redistribution of power and resources and the adoption of structural reforms. The collaboration of the Salvadoran MoH with the NHF ensured the strengthening of social participation and strong health system accountability towards the community (through the Offices of the right to health as an example). Spaces of community participation have been created in which PHC personnel can create mechanisms based on the real needs of the population in order to develop a more comprehensive approach of NCDs. Community-based peer support groups for NCDs provide spaces for sharing and learning about the reality of living with a chronic disease.

As the 2019 elections put an end to the decade long FMLN (Farabundo Marti National Liberation Front) government with the likelihood of important changes in the public health system analysed here; we consider it of importance to have documented and assessed the results and limitations of this experience of reform.

More than 40 years have passed since the Alma Ata declaration [57] and numerous experiences in implementing PHC have taken places all over the world, raising important achievements and challenges [58]. The implementation of the CPHC and its management of NCDs in Latin America and worldwide is still a work in progress [59]. Nevertheless, the analysis of the implementation of PHC, amplifies knowledge for addressing inequalities in health and reinforces the importance of an intersectoral approach with social participation and accountability within the health system.

## Limitations

Resource limitations meant that fieldwork was limited to a period of 3 months, and the full complexity of the sociocultural, political and economic context may not have been fully captured. Moreover, the high level of community violence in El Salvador at times limited the scope of the fieldwork carried out. These challenges were partially solved thanks to the support of key community members and health system personnel, who accompanied the field research group.

A more crosscutting general gender perspective is lacking from the research and it should be considered as a key aspect in studies in health [60]. Regarding the limitations of the methodology used, a common criticism of case study method is its dependency on a single case exploration making it difficult to reach a generalising conclusion [22]. This study complements two other qualitative methodologies (illness narratives and social connections mapping) that were applied in different departments of El Salvador and have been contrasted and enriched with objective information from official document review. In this sense, this case study demonstrates the value of context specific knowledge. On the other hand, even NCDs have specific characteristics in each context, although there are common patterns globally [61]. Lastly, in regard to the NCDs considered for this study, cancer and mental health patients were not represented and it would be appropriate to redress this. In this context, it is important to reinforce the research capacity of the Public Health Policies Observatory of the University of El Salvador – in which the NHF actively participates – as it could ensure the analysis of further developments of the national health system of El Salvador, and the impact of government change in 2019 on its structure and functioning.

## Conclusion

The Salvadoran Primary Health Care System, as developed between 2009 and 2019, shows strong advance in terms of accessibility, continuity of care of NCDs and quality of care and reinforces the importance of an intersectoral and comprehensive approach. Community engagement plays a key role in PHC and has ensured long-term commitment to remote communities and social accountability. However, challenges in PHC remain such as supplies shortages, the need to reinforce PHC workforce and the need for better coordination between levels of care in the approach to address NCDs. The process of identification of strengths and weaknesses in the management of NCDs in PHC provide keys to continue working and researching effective actions to reduce NCDs inequalities by further strengthening the right to health in PHC with UHC.

## Supplementary information

**Additional file 1.** Data collection: Semi-structured interviews.

## Data Availability

The datasets used and analysed during the current study are available from the corresponding author on reasonable request.
