# Supplementary material for: Comprehensive primary health care and non-communicable diseases management: a case study of El Salvador
Source: Int J Equity Health. 2020 Apr 6;19:50. doi: 10.1186/s12939-020-1140-x (PMC7132977; doi:10.1186/s12939-020-1140-x)
Supplement: Supplementary file 1 — Additional file 1. Data collection: Semi-structured interviews. [file 12939_2020_1140_MOESM1_ESM.docx]

**Data collection:**

**Semi-structured interviews**

# Patient interviews

## Patient interview 1: Woman with Hypertension

Entrevista con: Paciente HTA

Fecha: 13/06/18
Lugar de la entrevista: Unidad Comunitaria de Salud Familiar Jiquilisco
Código del informante: PFMJ001
Genero del informante: F
Nombre del entrevistador: MJ

1. **Antecedentes sobre el participante (datos sociodemográficos)**

I: ¿Me podría indicar su edad?

Mujer. Tengo 44 años.

**I: ¿Me podría usted hablar un poco acerca y usted y su familia?**

Nací en Jiquilisco, tengo 6 hijos, 5 hembras y un varón y tengo 6 hermanos: 4 hembras y dos varones, yo me acompañe y al repartir las tierras nos vinimos para acá, ya vine con dos hijos, mi vivienda es de ladrillo, tengo agua potable 24 horas, yo pago 23,50 dólares y voy a ver porque tan caro, de luz antes pagaba 8 y ahora pago 17 dólares, y pago cada dos meses entonces me salen como a 33 $ y es caro, y ya se me halla difícil porque necesito la luz para que mi hija estudie, solo mi compañero es el que trabaja, yo trabajo en los cuidados del hogar, porque antes trabajaba en la parcela pero como luego me cayó el potasio y el sodio y ya no voy al campo , ahora solo en mi hogar, yo recogía las semillas de milpa solo para el consumo del hogar.

**I: ¿Me puede explicar un poco más acerca del sustento familiar?**

Mi hijo varón trabaja, pero como él ya está acompañado, es poco lo que él me ayuda a mí. Mis hijas como están acompañadas, ya los esposos los mantienen a ellas. Ahora solo viven dos hijas conmigo de 19 y 7 años y una nieta de dos años, mis hijas me ayudan con alimentos, y gracias a Dios ellas no me dejan de la mano, aunque sea con alimentos ahí tengo yo el arroz y los frijoles. Mi compañero es el que trabaja y a veces le salen trabajos de 5$, cuando el sale a trabajar por ejemplo a picar leña y vende las carretadas a 20 dólares y así nosotros vamos saliendo.

Mis hijos estudiaron hasta octavo. Yo en casa tengo dos hijas una de 19 que estudio hasta bachillerato y otra de 8 que está en la escuela. Y mi hija tiene una niña pequeña de 2 años que también ya va a ir a la escuela; y su papa, bueno ya sabe usted que cuando una niña las deja embarazadas ya tiene que estar uno encima, porque ella abandono, pero ahí estamos nosotros criando a esa niña.

**2. Trayectorias de cuidado ante la enfermedad crónica**

**I: Ahora me gustaría que me hablara un poquito acerca de su enfermedad. ¿Cuándo la diagnosticaron?**

Me diagnosticaron de HTA, quiero ver, ya hace dos años, la fecha exacta no me acuerdo, pero desde entonces vengo luchando. A mí esto me comenzó por unos problemas que teníamos la familia con personas. El problema fue, que a mí me dijeron que iban a matar a un hijo mío, y no era así la cosa, era más bien por preocuparme, y como usted sabe no somo monedita de oro para caerle bien a todos, ya me comenzó todo eso, porque yo no padecía de nada más antes. Era una muchacha que llega de los cobradores, pero a mí me lo dijo solo para molestarme a mí, o por verme dañada, pero gracias a dios ya me siento bien. Mi hijo entonces lo tuvimos que llevar donde una familia, pero allí cuando ya llego un hermano de cristo y me dijo todo lo que paso, me lo fui a traer de vuelta otra vez. Pero aquello ya se me quedo esa angustia. Y de ahí me cogía mucha aflicción, tenía mucha tristeza con muchas ganas de llorar, no tenía ninguna alegría y ya al traerme a la clínica ya comenzaron los doctores a poner mano en mí, pero primeramente "el de arriba", ya después la mano del doctor. Ya de ahí para acá yo ya fui viendo que aquello ya había quedado, que padecía del corazón, entonces ya de ahí para acá fui teniendo el medicamento, de mes a mes, pero gracias al señor que los doctores de aquí son buenos , el señor les ha dado ese don para que ellos tengan esa manos poderosas de ellos de poder darnos medicamentos a nosotros, pero gracias a Dios ya voy va, ya me siento mejor ya no siento eso que sentía de unos años atrás, cuando me bajo el potasio y el sodio, pero era porque yo me iba a trabajar y casi no tomaba casi agua y el sol sofocante, cuando ya comencé con aquella angustia en la iglesia estaba, y ahí nació algo en mi cuerpo, aquel malestar que yo no aguantaba, pero ya de ahí me trajeron acá a la clínica en la noche, los doctores empezaron a diagnosticarme, a darme la medicina pues, gracias al señora ya hoy ya me siento mejor.

**I: ¿Hace cuánto le ocurrió todo esto?**

Esto fue hace unos 4 meses que estaba en la iglesia me empecé a encontrar mal tuve un calambre y me enrollaron los pies y la mano, sentí que me Moria y me trajeron a la unidad, volví a caer otra vez a la semana y me volvieron a traer aquí y ya de ahí el Dr. me mando en ambulancia para el hospital de Jiquilisco, ya ahí con el Dr. y los demás doctores me estuvieron viendo y gracias al señor me tuvieron 4 días, y cuando vine ya a la casa yo ya me veía mejor, solo débil del cuerpo.

**I: ¿Y en cuanto a la HTA, hace dos años como fue?**

Cuando empecé con la enfermedad fue en mi casa, y sentí depresión y le dije a mi esposo que que siento que me estoy muriendo y no sé qué es lo que me pasa, y ya es cuando el me trajo a la unidad acá esta especializada y ya me vio el doctor a evaluarme. En ese momento comenzaron a darme la medicación. Y gracias al señor yo ya me siento bien. Cuando me trajeron para acá el me trajo en carro de los hermanos de cristo, y se lo ha ido pagando después cuando ya tenía del trabajo, que me costó 5$.

**I: ¿Alguna vez ha ido usted a una clínica privada para asegurarse el diagnóstico?**

No he ido a ninguna clínica privada, por el dinero, aquí no más y cuando el doctor me deja para los exámenes, yo otro examen del corazón no me he hecho porque no tengo dinero.

**4. Calidad de la atención y percepciones de cuidado**

**I: ¿Y usted entonces aquí a la unidad se salud porque viene?**

Lo primero por lo que vengo la unidad de salud es para traer el medicamento, y segundo por anoche estuve con un gran frio y luego como que me entro fiebre, y un dolor en este pie que nunca se me quita, y ahora me dan calcio de nuevo. Cuando tengo una urgencia acudo a esta unidad andando o si ya estoy casi muriéndome entonces ya pagan transporte, pero mientras así a pie vengo.

**I: ¿Como encuentra el trato aquí en la unidad de salud?**

En la unidad de salud me tratan bien, todo ellos tienen mucha amabilidad, no tenemos por qué hablar mal de ellos. Todos me han tratado bien, aquí nunca me he quejado cuando he venido, todos trabajan igual. A mí me atiende el Dr. especialista siempre, el doctor es 10 porque siempre trata bien a uno, lo examina bien a uno; y los otros doctores también bien, porque si uno le dice esto, esto y esto, ellos lo examinan.

**I: El Dr. Especialista es del riñón, pero usted no tiene problema de riñón ¿no?**

Cuando se me bajo el sodio y el potasio salí con infección de riñón, pero ya gracias a dios eso me lo controlaron y ya estoy bien y en dos meses tengo que hacerme unos exámenes en el hospital de Jiquilisco.

**I: ¿Y cómo consigue usted ir hasta allá?**

Tengo que pagar el transporte, y gracias a dios menos mal que tengo un esposo lindo que el señor me ha dado y él se preocupa bastante por mí. El examen allí es de gratis, solo es el transporte que tengo que pagar. La ambulancia no nos lleva para exámenes, uno tiene que buscarse la vida. Y luego pues por ejemplo para citología me ve la Dra., las enfermeras también son muy amables y ellas lo que hacen es tomarnos la tensión, y a veces si trae calentura, van a ponernos el termómetro ahí. La promotora de salud va cuando nos ponen vacunas, y ahora me están poniendo una vacuna cada mes, contra la influenza, y como la tengo así a la par, vivo al lado de ella, cualquier cosa yo la digo lo que me pasa y ya me indican, y también me ha atendido la educadora porque yo tenía problemas y me ha dado terapia y me ha venido bien. También pase con la psicóloga que estaba antes y ya me ayudo bastante a solucionar mis angustias, y gracias a dios pudo contrallármelo todo.

**I: Y la educadora, ¿ha trabajado solo con usted o a nivel familiar también?**

La educadora solo ha trabajado conmigo. También me han indicado dieta, no grasas, solo verduras salcochadas, tomar fruta, poca sal y toda esta información me la ha dado el doctor especialista, es difícil cumplir con la dieta por la economía, no puedo pues, y no uno lo tiene todo, puede comprar poca cosa.

**I: ¿Que medicamentos toma usted?**

Tomo el suero, pastilla para la hipertensiva, calcio, acetaminofén para el dolor de cabeza y vitaminas.

Siempre me dan todos los medicamentos aquí, no tengo que comprarlos fuera, fíjese de que una vez la pastilla esta que me dan para el corazón me dijeron que tenía que comprarla, pero gracias a dios, a los tres días, me aviso el enfermero de que ya había, entonces ya vine yo a por ella.

El suero lo tomo ahora una vez al día, también bebo más agua, tomo el agua de mi casa que la hiervo y la dejo en el cántaro y vamos bebiendo, la cambio tres veces al día, he transmitido a mi familia que es saludable beber agua para no enfermar de los riñones.

**I: ¿Cuánto tiempo suele usted esperar cuando llega a la unidad?**

En la unidad se atiende en función de la hora de llegada, por ejemplo, si yo llegara a la mañana yo sería la primera va, pero si ya vengo tarde, entones tengo que esperar a que ya pase todo el mundo. Y entonces si me va a atender el Dr. Especialista llego yo a las 6,30h de la mañana para que me atienda de las primeras, pero ya me han tomado más y recoger medicamentos también llego a la mañana. Aquí hay un segundo turno a partir de las 10 de la mañana, y siempre me han atendido, siempre llevo la medicina.

**5. Apoyos y ayudas**

**I: En su casa entonces ¿cuáles son sus apoyos?**

Tengo apoyo de mi esposo y sobre todo de una hija que está siempre atenta y si me encuentro mal me trae en bicicleta a la unidad.

**I: ¿Que otros apoyos tiene usted para manejar la enfermedad?**

Lo primero los hermanos de Dios de la Iglesia, pero yo todo lo consulto con el de arriba, cuando tengo alguna duda el me da la solución. Y luego los problemas de salud solo con mi esposo. No lo comento con ninguna amiga, por el problema que usted le dice alguien los problemas y ya el vecino lo sabe. Entonces yo lo consulto con mi compañero de vida y ya luego me vengo para acá.

Aquí también venia de primero a las reuniones de los grupos de ayuda mutua, se hablaba de que uno debería de tener dieta, de no comer cosas saladas, grasas, dulces. Pero deje de ir, ya me aburría.

**I: ¿Y va alguna otra actividad de su comunidad?**

No yo no salgo solo a la iglesia. Me distraigo leyendo la biblia y orando, porque acuérdese de que cuando uno está triste lo que tiene que hacer es posarse de rodillas y pedir la solución al señor y que sea el que nos de la fuerza y la fortaleza, así que eso es lo que yo hago, después me viene después la alegría. Yo estudie hasta el octavo grado y se leer.

**I: ¿Y recibe algún sustento económico?**

No solo lo de mi marido, y para nosotros es bien difícil, solo lo que el consume de su trabajo. Cuando mi hijo lo llevamos donde el hermano de mi marido ellos también tenían problemas, porque como la mama de ellos esta invalida, ella se pasa el día acostada, entonces a él se le hacía difícil mandar ese dinero para andar dándomelo a mí. Nosotros a elle comprendemos bastante. Ellos me apoyaron con mi hijo un mes no más.

**6. Percepción de cambios en el tiempo**

**I: Usted, ¿qué cree que ha podido cambiar aquí en la unidad con respecto a 10 años atrás?**

Bueno yo antes no estaba viniendo, solo cuando estuve embarazada, venía a los controles, pero ya desde que nació mi niña ya no vengo, de allí a bueno que ya me causo la HTA, tuve que estar viniendo más. Desde entonces he visto un cambio maravilloso, porque ya lo que sentía ya no lo siento, ya me va mermando poco a poco, ya voy sintiendo más salud en mi cuerpo, ya me encuentro mucho mejor, por eso la atención es buena, ya no siento lo que sentía. Nunca he pagado por la consulta médica.

**I: ¿Que cree usted que se podría mejorar en la unidad de salud?**

La sugerencia que hago: que los medicamentos no falten nunca, que estuviera siempre. También una ayuda económica, para poderlo uno comprar lo que le mandan aquí, y tener una dieta eficaz, sana, y así iría a las despensas de Usuluán que es donde son más sanas. Aquí si pasan vendiendo porque pasan carros de verduras, y no es caro, pero se compran más baratas allá en Usuluán, porque acuérdese de que aquí ya es contra reventa. Las cosas de la dieta, a veces si voy a Usuluán y voy en en bus me cuesta 2 dólares ida y vuelta, compro frijoles, aceite…pero las verduras no las puedo comprar porque son muy caras.

**I: ¿Y a parte de la dieta hace usted alguna otra cosa para su enfermedad?**

Yo camino 4-5Km al día, al dejar y recoger a mi hija a la escuela. También salgo a veces a ver a mi mama en bus, porque ella es también hipertensa y va a la clínica de santa Elena, le acompaña una hermana mía.

## Paciente interview 2: Woman with Hypertension

Entrevista con: Paciente HTA

Fecha: 13/06/18
Lugar de la entrevista: Unidad Comunitaria de Salud Familiar Jiquilisco, Cantón San Ramón
Código del informante: PFML001
Genero del informante: F
Nombre del entrevistador: ML

**1. Antecedentes sobre el participante (datos sociodemográficos)**

Le agradecería si me pudiera contar un poco sobre usted, su edad, donde nació la gente que vivía en su hogar cuando era niño…un poco todo eso.

Tengo 77 años nací en el Cantón San Ramón acá mismo, siempre he vivido acá, acá conocí a mi marido era compañero mío de la escuela. Cuando era niña vivíamos 9 personas en total, tenía 4 hermanos, 2 primos, un hermano de crianza y de ahí mi papa y mi mamá.

- ¿Y fue a la escuela entonces?

- Sí, a esta de acá hasta tercero, hasta 8 años no había más acá en el cantón.

- ¿Cuánto tiempo llevan casados?

- 61 años casados, tenemos 4 hijos, dos muertos, tuvimos 6 hijos, 3 hembras y 3 varones. Uno se murió de tiernito y el otro por tema de violencia, el hermano de la novia es que le mató. Una vive acá al lado, pero tiene casa aparte. Una vive en la palma, otra en Chalatenango y el otro está en los Estados. ¿Y nietos tienen? 13 nietos y 11 bisnietos.

- ¿El agua en casa de donde la cogen?

- Bebemos directamente del chorro, acá está bien limpia viene directamente de la montaña y ha pasado exámenes.

- ¿Y han vivido siempre acá, la guerra y todo?

-Sí siempre acá…que guerra? ¡Que nos han tocado vivir dos! La de acá nos tuvimos que ir un mes con todos los niños a Citará, pero luego volvimos de nuevo al cantón, no nos pasó nada más gracias a Dios.

**2. Trayectorias de cuidado ante la enfermedad crónica**

- ¿Qué enfermedad crónica tiene usted? Soy hipertensa desde hace años ya.
- ¿Cuándo y dónde fue la primera vez que buscó ayuda para los síntomas que presentaba? En la cocina es que me dio me encontré bien mal como con nudo bien fuerte en la garganta y de ahí me mareé y me caí y me llevaron al hospital de Chalatenango. Me llevó don Julio al hospital porque mi marido andaba trabajando en la tierra. Ahí ya me vieron y me dijeron que tenía presión alta y me dieron medicamentos.
- ¿Le contaste a alguien sobre tu enfermedad? A la familia y así sí, pero al resto sí no me gusta contar. Fue en el 1989, a mi hermano le habían matado en 1988 y esto yo estaba preparando una comida para conmemorar la muerte de él.
- ¿Y luego que pasó? De Chalate me mandaron al hospital Rosales para hacerme la prueba de esfuerzo y todo eso. ¿Qué le dijeron que fue? Fíjese que eso no le cuentan a uno, solo me dijeron que era del corazón. Estuve 13 años lleno al Rosales década 3 meses me veía el médico y cada mes tenía que ir a recoger el medicamento y de ahí cuando me operaron de la rodilla en 2002 ya fue cuando me pasaron de nuevo al de Chalate. Cuando iba al Rosales iba en el bus yo solita como ahí tengo una hermana ahí me quedaba yo como dan las citas a las 6 de la mañana me quedaba allí a dormir, no recuerdo cuantos colones me costaba, pero era un gasto.
- ¿Ahora donde recoge los medicamentos?
- Ahora acá, desde que me empezaron a ver en Chalate luego me pasaron a la Unidad de salud de La palma para que me siguiera el internista y luego ya me lo pasaron a dar directamente acá en el cantón desde hace 2 años. Me lo han ido acercando todo. Desde que me pasaron a La Palma ya siempre me ha seguido el mismo internista iba cada 3 meses para allá y desde hace como dos años ya me ve acá y no tengo que bajar a la Palma.
- ¿Tiene que pagar para recoger la medicación? ¿Está siempre en la unidad?
- No nunca he tenido que pagar, normalmente sí que hay alguna vez que no ha habido he bajado a la especializada de La Palma y ahí la he conseguido, dos veces no más la he tenido que pagar. Cuando iba al hospital Rosales pagaba un dólar por cada medicamento
- ¿Qué dificultades encuentra para el manejo de su enfermedad? Cuando tenía que ir al hospital Rosales me costaba mucho lo del transporte y el tener que pagar por tantas medicinas que yo me tomo, entonces se pagaba…ya cuando llegó el gobierno esté ahí ya fue que no pagábamos los de la tercera edad con el Zaca que era de Arena y luego ya con el Frente (FMLN) quitaron de pagar todo. Yo antes cada examen antes de la cita me costaba un dólar luego ya no tuve que pagar, pero antes cuando me hicieron la prueba de esfuerzo y todo esto ahí sí que tuvimos que pagar 600 colones por la prueba, otros 275 colones, fíjese todo ese dinero costaba. En Chalate ya no tenía que pagar la medicación.

**3. Episodios de enfermedad crónica**

¿Ha tenido algún otro episodio grave? Sí una vez que estaba esperando en la consulta del corazón en Chalatenango y yo sentí como que iba subiéndome de la silla mareada y cuando ya la licenciada que me había tomado la tensión me mandó llamar y me pasó con el medico al que le enseño mi presión en un guante que se lo había escrito. ÉL me dijo que estaba medio muriéndome con esa presión y de ahí me llevaron a la emergencia, me pusieron una pastilla debajo de la lengua y no se me pasaba estuve ingresada 5 días, eso fue como en 2004, aunque no recuerdo muy bien el año exacto.

Y de ahí he estado bastante bien, solo una vez que me vino un dolor de estómago feo y me hicieron la prueba esa de la bacteria y me salió que la tenía, ya me la hizo el Dr. Burgo en La Palma, a mi hija también le pasó y como me conoce a mí y la conoce a ella ahí nos dio los medicamentos. De aquí del cantó me dio referencia el doctor para hacerme los exámenes, al final me fui a un sitio privado porque ahí salían de inmediato. Ahora sigo teniendo molestias a veces del estómago.

**4. Calidad de la atención y percepciones de cuidado**

Cuando acude a la **unidad de salud (tanto a la del cantón como a la especializada),**

- ¿Se siente **bien recibido**? Aquí me reciben muy bien, allá en la unidad especializada solo le ven a uno si lleva referencia, como ahora viene acá el doctor al cantón ya hago todas mis consultas acá con él, la última vez fue el 8 de marzo.
- ¿Cuánto **tiempo espera** normalmente para ser atendido? Enseguidita que llega uno le dan el número y es por orden de llegada. Y cuando viene el internista llevo mi cita de la vez anterior.
- ¿Desde cuándo le ve el internista en su cantón? Mas de un año, viene una vez por mes, aunque a mí me ve cada 3 meses él viene acá cada mes, antes me veía en Citará y previamente en La palma.
- ¿Cuánto suele esperar hasta que entra en consulta? Nunca he medido el tiempo fíjese, me quedo platicando con la gente allá (risas).
- ¿Cuánto tiempo suele quedarse en la unidad de salud cada vez que acude? Pues voy como a las 7 o 7 y media de la mañana, la gente va llegando, pero cuando me ve el doctor Burgos yo voy con mi cita y salgo antes del almuerzo.
- ¿Con cuántos sanitarios entra en contacto cada vez que acude y por cuánto tiempo? Cuando llego me prepara la enfermedad que me mide talla toma la presión y todo eso y de ahí ya me ve el doctor. Cuando voy solo a recoger la medicación está la auxiliar a ella solo veo y platicamos.
- ¿Considera que le brindan el tiempo necesario de atención?
- Ah no si me dan el tiempo que necesito. No conmigo nunca se han portado mal la verdad.
- ¿Considera que le brindan la **información** necesaria sobre su enfermedad?
- El doctor me da la información me lo explica todo y luego me hace la receta
- ¿Le apoya la unidad de salud con su **tratamiento**? ¿Cómo? ¿Sugiere alguna medida de mejora con respecto al acceso al tratamiento para su enfermedad?
- El año pasado si compre tres medicamos, pero fue una excepción cuando no ha habido mi hija me lo ha conseguido allá en la Palma con los de Fosales, su compañero trabaja de seguridad allá a las noches.
- ¿Ha tenido alguna situación que le ha dificultado **acceder** a la unidad de salud?
- Pues ahora me pilla bien cerquita la verdad, solo una vez que estaba bien mal y vino la licenciada a inyectarme a casa.
- ¿Cuándo va a la unidad de salud, que platica con la gente allá, usted cree que la gente se siente bien tratada o no? ¿Y con usted?
- Sí viera como son todos, todos los doctores que vienen hacer el año social son bien bonitos conmigo, aunque al año se van.
- ¿Se siente **apoyado** por el personal sanitario en el seguimiento de su enfermedad? ¿Habría algo que cambiaría para mejorarlo?
- Si me siento bien apoyada, no cambiaría nada, está bien así. En casa yo me organizo las medicinas que estoy tomando, mi hija también me ayuda cuando lo necesito en cualquier cosa. Y económicamente mi hijo el de los Estados nos manda una ayudita cuando se puede. Acá en la comunidad solo voy a la iglesia católica, allá nos juntamos todos y cada dos domingos vamos todos para Citará y se hace una fiesta con todas las comunidades, verá que lindo es allá con todas las familias y todo.
- ¿Y en concreto para su enfermedad crónica existe algún tipo de apoyo?
- No no hay nada…
- ¿le gustaría que hubiera algún grupo?
- La verdad es que sí me gustaría.
- ¿Quién ha trabajado en casa? ¿Reciben algún otro tipo de ayuda económica?
- Mi marido ha sido agricultor toda la vida parte lo dejamos para consumo nuestro que dejamos en el graneros y parte se les vende a los coyotes y yo he cosido mucho, para todos los hijos, pero también me hacían encargos hasta de trajes de novia, tengo dos máquinas de coser, una de ellas de 1966 y otra del 91 ahora le enseño si quiere… Mi marido sigue trabajando la tierra no se cansa nunca yo ya no puedo coser por la vista que me está fallando. De ahí no recibimos ninguna ayuda ni subsidio del gobierno, nada de eso.

**5. Percepción de cambios en el tiempo**

- ¿Ha notado cambios en el manejo de su enfermedad en los últimos diez años?
- ¡Como no! El que ya no tenga que pagar la cuota voluntaria cada vez que voy al doctor y el que cada vez lo haya tenido más cerquita de mi casa, eso ha sido un gran cambio, me ha facilitado mucho imagínese antes tener que ir cada mes al Rosales…
- ¿De qué manera las cosas mejoraron o empeoraron?
- No, han mejorado claramente…
- ¿Por qué cree que ha podido haber estos cambios?
- (la participante titubea e interviene su marido) Pues a través de las directivas cantonales las ADESCO se han conseguido muchas cosas, el dispensario, la escuela…ya no me acuerdo cuando se hizo el dispensario, pero lo luchamos, antes ahí venia una ONG que venían a dar unas consultas por mes y no nos hacían pagar, antes de que llegara el FMLN al poder.
- ¿La unidad de salud del cantón no recuerdo exactamente cuánto hace que empezó, la Beatriz empezó de promotora acá en 2009 no? (lo discuten entre ellos que año fue exactamente).
- El marido: yo llevo muchos años en **ADESCO**, ahora soy el presidente, pero he sido de todo secretario tesorero. No recuerdo cuántos años lleva, pero nos ha sido muy útil para reclamarle a la alcaldía, hace poco hemos conseguido que pusieran pavimento en las calles que hasta ahora eran de arena.
- La participante: yo nunca he participado en ADESCO ya estaba él ahí metido.
- ¿Tiene alguna sugerencia para poder mejorar el seguimiento de su enfermedad crónica desde el primer nivel de atención?
- No está bien como está.
- ¿Creen que esto se va a mantener o se puede perder?
- Bueno a ver qué pasa, pero ojalá y no se pierda, si entra nuevo gobierno quizás y cambien todo como hacen siempre…

## Paciente interview 3: Woman with diabetes

Entrevista con: Paciente Diabetes

Fecha: 13/06/18
Lugar de la entrevista: Unidad Comunitaria de Salud Familiar Perquin
Código del informante: PFML002
Genero del informante: F
Nombre del entrevistador: ML

**1. Antecedentes sobre el participante (datos sociodemográficos)**

**I: ¿Conoces tu edad? ¿Dónde naciste?**

Ttengo 57 años, nací en Perquín.

**I: ¿Cuántas personas había en su hogar cuando eras un/a niño/a? ¿Tiene hermanos y hermanas?**

Vivíamos 12 en la casa, con mis tíos y tres hermanos y mi mama nos dejó con mi abuela y mis tíos, yo tengo un hermano varón y una hermana.

**I: ¿Asististe a la escuela?**

Por la pobreza no nos enviaron a la escuela y mi abuela se iba a Perquin para recoger ropa y nosotras también lavábamos y por la noche íbamos a moler quintales de café en la piedra, las que éramos hembra trabajábamos mucho.

**I: ¿Tiene hijos/as? ¿Viven con usted en su hogar?**

Me case y ahí seguimos. He tenido 12 hijos, me quito el señor 5 y una hembra, entonces me quedo una hambre y 5 varones, se murieron de año y medio o dos, fue para la guerra, y no podía ir uno de aquí para abajo. Todos mis hijos son del mismo padre, es que yo los tuve todos de un solo; yo me case, como 13 años tengo de haberme casado, y me acompañe antes y mi primer hijo lo tuve a los 16 años.

Ahora ya como todos se casaron quedamos que actualmente vivimos en casa 5 con mi esposo, un hijo, nieta y un bisnieto de otra nieta que lo tuvo a los 13 años, y yo se lo quite porque ella es muy pequeña y no sabe lo que es tener un niño, y ahora tiene 5 años y lo llevo a la escuela.

Yo antes yo trabajaba en todo, yo hacía tamales, hacia pan, de todo pues, pero ahora con las enfermedades ya no puedo, tengo muchas veces un dolor de cabeza que me hace llorar, llevo con este dolor de cabeza hace 10 años.

**2. Trayectorias de cuidado ante la enfermedad crónica**

**I: ¿Tiene usted una enfermedad crónica? ¿Cuál es?**

Yo padezco de tensión y azúcar. Primero me comenzó con el azúcar, y yo creo que como vendía tortillas en un comedor no tenía tiempo para comer y me tomaba una cucharada de agua con azúcar, y de ahí cuando ya me fui a ver ya tenía. Yo ya tenía entonces en casa 5 hijos y mi marido que también trabajaba, a la par.

Al principio yo me sentía con una gran violencia, tenía que beber mucha agua de un litro y todo rato sintiendo ganas de orinar y de ahí me enviaron a la clínica de san Luis, y allí me hicieron pruebas y me diagnosticaron de diabetes y desde entonces no me pude componer, hace 10 años, y de ahí ya no me compuse. Lo que me dicen a mi es porque no pido una cita aquí para que me den una cita allí en el Hospital de San Salvador, porque allá si van a saber lo que es, eso me han contado varias de las personas que van allá, porque yo ando hasta llorando, pero como yo nunca les he dicho para que me vean en san salvador, porque allá es lo último, porque no me hallan nada. A este establecimiento solo me dan el acetaminofén, pero a mí eso no me calla nada, me tomo dos juntas y en la tarde me quito otras dos, y a mí el dolor de cabeza no me se me quita.

**I: Descríbame como maneja usted la diabetes**

De la diabetes estoy bien me pongo insulina, y una vez me chequee y la tenía bien bajita, y me agarro violencia, y lo que hice fue acostarme. Por eso yo me la chequeo y si está muy baja no me pongo la insulina. Yo me chequeo en mi casa el azúcar con una máquina que me tuve que comprar, para ver cuánto se me sube y baja y me costó como 100 dólares y no me la chequeo todos los días porque las tiras son las caras, fijes que me cuestan 47 dólares, es caro, y ya para todos los días no me aguanta, me las compro cada dos meses. Para la tensión tomo enalapril que me la dan en esta unidad especializada, pero las insulinas tengo que ir en san miguel y otras medicinas que yo compro es la jeringa de inyectar, que me cuesta 16 dólares.

**I: ¿Quién le ayuda entonces a pagar esos gastos?**

Mire, soy sincera, tengo un hijo en EEUU y ellos de poquito me mandan porque ellos tienen familia allá, y ellos de poquito me mandan para la medicina y como hay que pagar agua, hay que pagar luz, hay que pagar teléfono, me ayudan económicamente; del agua pago tres dólares, pero ya en el verano es más porque hay que regar los jardines, porque a mí me gusta regar jardines, y la luz 25-30, del aseo pago 5,50 y del alumbrado de la calle pago 7 dólares.

**I: ¿Como se maneja la enfermedad crónica en este establecimiento?**

Mire por ejemplo, mi esposo tiene alto el colesterol, pero el viene cuando ya está grave, y de ahí cuando ya se encuentra un poquito mejor el ya no se asoma, no acude a las citas y no tiene dieta y yo si llevo dieta yo no como pan, no tomo café, no como guineo, ni sal... aquí es donde me han recomendado la dieta; porque aquí quien me mira es la Doctora medica de familia, las enfermeras solo la de anotar, y también con la educadora, si me ayudan porque me dicen lo que tengo que comer.

**3. Episodios agudos de enfermedad crónica**

**I: ¿Ha tenido usted alguna emergencia?**

Por la tensión me enviaron al hospital porque no me bajaba, me llevaron de noche al hospital de gotera, entonces como ellas fueron a dejar a unas embarcadas en la ambulancia yo les pedí que me llevaran, y allí ¿qué hicieron?, ponerme una tensión de 150 sin verme en el expediente, allá en el hospital de gotera, y yo me volví a casa, porque que allí que hacía, era bien de noche, esto ha ocurrido hace un mes.

También hace 6 meses me ingresaron en Gotera porque no me bajaba el azúcar.

**I: ¿Como comenzó a hacer el seguimiento?**

La medica de familia de acá me mando para San Miguel, porque aquí solo me dan liquido de la insulina más lechosa y allí en san miguel de la más clarita, entonces me cayó bien a mí. Ahora voy a San Miguel una vez al mes, voy en bus y me cuesta 3,5$, y busco quien vaya conmigo, porque por la vista solo veo por un ojo entonces busco a una muchacha de aquí del pueblo para que me acompañe, y la pago el pasaje y comida. Nadie de la familia me puede acompañar porque ellos pasan trabajando.

**I: ¿Como ha cambiado entonces su situación laboral?**

Hace 5 años que ya no trabajo, yo solo la casa hago, deje por el dolor de cabeza y porque no veo bien. Una vez estaba yo en gotera y perdí el control, fíjese las vendedoras me levantaron y me fueron a sentar hasta que ya se me paso, por eso que no salgo sola.

Entonces a San Miguel voy para las insulinas y acá solo para ver como estoy para que me leven el control y las tiras para el azúcar las compro en la farmacia de Gotera.

**4. Calidad de la atención y percepciones de cuidado**

**I: ¿Como me usted que es el trato aquí en esta unidad de salud?**

En la unidad de salud me tratan bien, yo no me puedo quejar, y en el hospital me tratan bien.

**I: ¿Cuánto tiempo tiene que esperar para que la atiendan?**

Acá en la unidad de salud, depende de la gente que ya se tenga, ya si tiene dos pasos rápidos ni hay más tengo que esperar más.

**I: ¿Como ve el personal de acá?**

Son amables

**I: ¿Cree que hay algún aspecto que le falte?**

Yo necesitaría ayuda para mis enfermedades.

**I: ¿Como viene acá y cada cuánto tiempo?**

Yo pago una cosita para el pick-up, y tardo como 15 minutos y vengo al mes o cada dos meses.

**I: ¿Hay alguien que le van a visitar a casa?**

Nos visitan de la promotora en casa y hablamos.

**I: ¿Hay algún grupo de apoyo aquí?**

Si hay, y voy y ahí platicamos, y hay unos que tienen dolor en el corazón, y varias enfermedades, unos tienen una cosa y otras.

**I: En cuanto a la medicación ¿ha tenido algún problema de tomar?**

Antes me daban pastillas para el azúcar y hace tres años que me pongo insulina, la enalapril me tomo uno por la mañana y otro por la tarde.

**I: ¿Alguna vez ha utilizado medicina natural?**

Yo estuve tomando una valeriana para la tensión, pero ya la deje de tomar.

**I: ¿Alguna vez ha tenido que ir a clínica privada y como ha sido?**

Si he ido a clínica privada en Gotera varias veces por el dolor de cabeza, ahí cuando voy por gotera me fijo en los carteles de clínica y voy, o porque alguien me recomienda un doctor que sea bueno, y uno por curarse va.

También la gente me ha dicho que tome unas vitaminas para el dolor de cabeza, el nervobiol.

**5. Apoyos y ayudas**

**I: ¿Por quién se siente usted apoyada?**

Yo hablo de mis enfermedades con mi hijo y mi esposo.

**I: ¿Dentro de su comunidad hay algún sitio donde usted reciba apoyo?**

En la comunidad si hay apoyo en el caserito la Tejera y allí habla con las amigas y paseamos, y quitar el estrés, pero donde vivo no hay una organización y casi no voy a la iglesia, porque como me baja el azúcar.

**6. Percepción de cambios en el tiempo**

**I: ¿Me puede describir si existe algún cambio en los últimos 10 años en cuanto al manejo de su enfermedad?**

Ahora está todo mejor, hay más medicinas, más personal, nunca he tenido que pagar en la pública. Hace años fui a una clínica privada a San Francisco por el dolor de cabeza y me costó 200 dólares, y me dijeron que no tenía nada.

En gotera también estuve yendo a una clínica privada por este dolor de cabeza, pero los doctores, fíjese yo estuve lleno a una consulta privada porque tenía dolor por el colon, de ahí le hago la plática a el de que no aguanto el dolor de cabeza, no se me quita, me dijo que le llevara las radiografías y a los 8 días fui de vuelta a ponerme una inyección y ya lo vio y me dio unas medicinas para el dolor de cabeza y yo creo que eran las mismas que me dio para el colon y se lo comente a mi hijo y ya no volví, porque estos lo que quieren son billetes nada más.

Ahora lo que vengo a ver es que se me hinchaban las canillas y la doctora me envió a Gotera para hacer unos exámenes de orina, y ahora me van a enviar a San Miguel para otros exámenes.

**I: ¿Que recomendaciones daría?**

Yo recomendaría que hubiera más de todos los establecimientos cercanos para vernos, porque esta largo y hay que gastar. También ayuda para los diabéticos.

## Patient interview 4: Woman with Hypertension

Entrevista con: Paciente HTA

Fecha: 13/06/18
Lugar de la entrevista: Unidad Comunitaria de Salud Familiar Perquin
Código del informante: PFMJ002
Genero del informante: F
Nombre del entrevistador: MJ

**1. Antecedentes sobre el participante (datos sociodemográficos)**

**I: ¿Conoces tu edad? Si es así, ¿a qué edad tienes?**

Buenas tardes, tengo 70 años, nací en Perquín.

**I: ¿Dónde naciste? ¿Cuántas personas había en su hogar cuando eras un/a niño/a?**

Éramos 6 hermanos, dos varones y 4 hermanas, y mis hermanos se fueron y me quede con mi mama y mi papa. Luego yo ya me acompañé y nos fuimos a vivir cerca de ellos. Luego ellos fallecieron.

**I: ¿Tiene usted hijos?**

Yo tengo 6 hijos, como mi mama, 4 hembritas y dos varones, y ahora solo vivimos yo y mi esposo, porque todos mis hijos están acompañados. Mis hijos están al cuidado siempre mío, siempre vienen a ayudar con las cositas.

**I: ¿Y usted en que trabaja?**

Yo nunca he trabajado solo en el cuidado de mi hogar, mi marido tiene 73 años y trabaja ahora pero poco, porque ya está anciano, antes trabajaba de agricultor, en la milpa.

**I: ¿Me puede describir un poco su hogar?**

Yo vivo en la casa de mi papa, que es una casona que el hizo, es el recuerdo que me dejo. Cuando me case yo me fui a otra casa y cuando fallecieron, nuestro hermano mayor repartió todo y me quede con la casa. Las paredes son de adobe y el tejado de madera, tengo agua en casa y es potable, tengo chorro, se paga solo 1,5 porque es por la comunidad, la luz si está más cara, pago más de 10 dólares y antes que era barata no llegaba ni a 5 dólares, para la cocina compro el chinito de gas y no llega para 12 dólares cada tres meses. Mis hijos están pendientes de nosotros, son pobres, pero siempre nos dan algo para comer y ayuda, todos viven aquí cerquita, solo una tengo que vive más lejos pero siempre viene. También tengo bastantes nietos y bisnietos.

**2. Trayectorias de cuidado ante la enfermedad crónica**

**I: ¿Tiene usted una enfermedad crónica? ¿Cuál es?**

Solo soy hipertensa me lo diagnosticaron a los 45 años, siempre vengo a la unidad especializada. A mí me estuvieron mandando a Rosales por una operación, porque me encontraron los riñones inflamados, y de aquí la señorita C me mandaron para el hospital de gotera, y de ahí para allá, y mire cuanto me cuesta mucho ir para allá, el pasaje 25 dólares ida y venida y ya he ido tres veces y estoy en cita y exámenes me toca una operación porque tengo un quiste en el riñón no tengo miedo, ya me han operado de un cáncer de la matriz, y yo no tengo miedo, yo les digo ustedes no se asusten, mis hijos me preguntan si quiero y yo si quiero operarme, pero les digo que tienen que andar detrás de mí, porque ligerito a uno se le va de la vida, el cáncer me quitaron todo, en el 2005 y aquí estoy, estuve dos años con colostomía y me lo arreglaron en san miguel y de allí me enviaron a los Rosales para el cobalto y allí me quemaron la vejiga.

**I: ¿Qué paso cuando la diagnosticaron de HTA?**

Bueno yo sentía un hormigueo en los labios y me vio un doctor en una clínica publica y me dieron bastantes medicinas y me dieron cita para dos meses. Luego cuando me engrave por el cáncer que me lo detectaron porque me agrave bien y me llevaron a san miguel y allí me operaron, llamaron a una ambulancia desde aquí Perquín y tuve que ir a gotera y de ahí me llevaron a San Miguel, y me llevaron allá, me acompaño mi esposo y mis hijos, estuve 12 días, de ahí me despacharon y de ahí el doctor tenía miedo que yo me iba a morir, y una hija mía dijo “no doctor, no se va a morir, la queremos viva y sana, uno tiene que tener paciencia”, uno no tiene que hacer caso de los doctores. Y mis hijos como trabajan, me ayudan y cuando voy a rosales siempre me acompaña una hija. Yo a consulta acá vengo en un pick-up que pasa a las 6:30, a las 11:00, antes no había carro y siempre nos tocaba caminar.

**I: ¿Ha tenido alguna vez problema en conseguir la medicación?** 12:12

Siempre he tenido la medicina y si no tienen aquí en la unidad la medicina, me voy a Torula a la unidad de allí. No he tenido que comprar nunca la medicina.

**3. Episodios agudos de enfermedad crónica**

**I: ¿Ha tenido usted alguna otra vez alguna urgencia?**

No solo aquella vez que me tuvieron que llevar al hospital. Y al hospital Rosales también me mandaron hoy, para el 11 de julio tengo allá consulta y el 22 de agosto me operan. Esta Diosito conmigo, sí, yo desde que me tratan, en el nombre del señor voy a salir bien, es como que me da un ánimo aquello.

**I: ¿Como ocurrió todo aquello?**

Yo estaba en gotera, y allí me hicieron una ultra porque yo me sentía mal, yo sentía dolor fuerte y después de eso me hallaron ese quiste en Rosales. Pero yo digo gracias a Dios porque los doctores están siempre con uno, yo estoy contenta de todo eso.

**4. Calidad de la atención y percepciones de cuidado**

**I: ¿Cómo siente que es el trato aquí en la unidad?**

Yo estoy contenta en la unidad de acá, con los doctores, enfermeras ellas nos anotan nos pesan y de todo, en la Dra. Medica de familia que me mando para Gotera y de allí para el Rosales. En Gotera son muy amables y hay mucha gente, pero el Dr. que allí me trato es bien amable. También bien atentos en Rosales, pero allí hay gentío, difícil conseguí los cuartos, conseguir algunos papeles, pero allí hay carteles que le indican donde tiene que ir uno, es bien Galán aquello. Yo solo tomo medicina para la tensión, nunca he comprado el medicamento.

**I: ¿La acompañó alguien cuando tiene que ir a él Rosales?**

Cuando fui me acompaño la familia, pero ellos luego se vuelven, porque como tienen la familia, ellos tienen que regresar al hogar.

**I: ¿Tiene que esperar mucho tiempo cuando viene acá?**

Bueno yo si tengo que venir acá, vengo y sino no puedo y me tienen que hacer alguna cura, yo mando que vengan a mi casa la enfermera. La promotora también viene, no tarda ni un mes para venir a verme el agua, cuando necesita verle las cositas al agua viene a darme. Aquí me vengo a tomar la tensión

Los dos venimos aquí, mi marido padece de artritis, padece de las canillas, se hizo una ultra y pareció así el huesito nunca hemos ido a clínica privada.

**I: ¿La recomiendan algo para su enfermedad?**

De llevar una dieta, de no tomar sal, ni café, que los huevos no los tome seguidos, le dan bastante dieta a uno, y que salga a pasear, todo eso le dicen a uno. En las comidas lo cumplo, en no tomar mucha sal, no beber mucho café, no tomar chocolatinas, porque todo tiene grasas, si come arroz salcochadito, que no le echen mucho curtido. Per a caminar no puedo, porque ya mis canillas me dan mucho dolor, y ahora con este dolor no puede una ni dormir.

**I: ¿Y alguna vez ha tomado usted algún remedio natural?**

No he tomado nunca ningún remedio natural, solo las pastillas que me dan, no he tomado porque no me lo han recetado. Solo cuando me operaron la doctora me dijo que tomará un agua que se llama chichipince, y me lo tome un mes, pero era para sanar las heridas. Yo lo cogía del monte, y me lo preparaba y le tenía mucha fe. La promotora también me lo recomendó, y si sentía que me mejoro.

**5. Apoyos y ayudas**

**I: Me ha estado comentando acerca de su familia, ¿por quién más se siente apoyada en su enfermedad?**

Mire, por ejemplo, aquí la Doctora me dijo que me iba a conseguir donaciones de sangre, porque eso si es caro, yo estoy bien alegre, porque me van a dar la carta, para que ya la presente allí en el Rosales, ay fíjese que bendición de Dios, ellos apoyan a las personas de la tercera edad.

**I: Y dentro de su comunidad ¿siente usted apoyos?**

SI siento apoyo por los vecinos cercanos, porque si les digo que voy a salir hoy, me vigilan que no vaya a ver ninguna cosa. También tengo amigas y los domingos venimos a los cultos.

**I: ¿Y usted está en alguna organización dentro de su comunidad?**

No, No fíjese que no.

**I: ¿Y usted viene aquí a algún grupo de apoyo?**

Los vecinos míos vienen aquí, a pasar consulta y coger sus medicinas.

Mi principal apoyo son todos mis hijos y mi esposo también, porque como le cuento aquella hija que vive en sabaneta, ella en media hora está aquí para irnos, viera lo que les cuesta, si no vendría un día antes para dormir aquí conmigo, Y de ahí todos los muchachos me dicen tome mama para el pasaje, o para alimentos, siempre me apoyan.

**6. Percepción de cambios en el tiempo**

**I: ¿Y usted si viéramos 10 años atrás que cambios cree que ha habido en el sistema de salud?**

Ha habido bastantes cambios, porque hay medicinas nuevas, el agua lo están viendo de seguido, allá tenemos un gran tanque que vinieron a hacerle una visita, y todos tuvimos que pagar esa visita para que venga la gente de allá para que hicieran el examen en el tanque, mire que galán. Yo siento bastante ayuda, apoyo y mejoramiento.

**I: ¿Que otras mejoras o empeoras ha visto usted?**

Por ejemplo, ahora van a vacunar a uno allá, las enfermeras fueron con las muchachas a vacunarnos. Solo pasan ellas a vacunarnos, pero los doctores no vienen porque ya venimos nosotros antes aquí, antes de engravar.

**I: ¿Que recomendaciones tendría de mejora?**

Yo creo que como mejora que compren medicinas de las buenas, pero aquí son bien amables, prefiero como es todo hoy, antes tenía que comprarlas, y a veces no había para comprarlas. Ahora hay buenas medicinas.

## Patient interview 5: Man with Hypertension

Entrevista con: Paciente HTA

Fecha: 13/06/18
Lugar de la entrevista: Unidad Comunitaria de Salud Familiar La Palma
Código del informante: PMML004
Genero del informante: M
Nombre del entrevistador: ML

**1. Antecedentes sobre el participante (datos sociodemográficos)**

**I: Lo primero sería, ¿conoce usted su edad? ¿dónde nació?**

Hombre. Tengo 77 años, nací en cantón pazo de canoa, en el departamento de Sonsonate, tenía 2 hermanos y 2 hermanas, mi papa y mi mama. En mi casa ahora vivimos mi mujer y yo, la muchacha trabaja aquí, tengo 6 hijos, 4 varones y dos hembras, dos viven en honduras, una en Italia, una en salvador, uno en costa rica. Con la señora no estamos casados, amarrados solo. Tengo agua potable en casa, pago 18 dólares al año, luz paga 40 dólares porque tengo un negocio, está el molino, una cámara y un aparato, de gas gastamos 66 dólares al mes.

**I: ¿Usted en que ha trabajado?**

Aquí en la tienda solamente. Yo trabaje antes en la agricultura, hacia milpa, he trabajado bastante cuando vivía en Sonsonate. Luego ya mayor mis hijos no me dejaron trabajar en otras cosas, pero si trabaje en otros negocios, arreglando carros, en empaques poniendo adornos a los carros, trabajo suave. Ya no trabajo.

**2. Trayectorias de cuidado ante la enfermedad crónica**

**I: Ahora vamos a hablar de las enfermedades crónicas, ¿qué enfermedad tiene uste?**

Tengo hipertensión arterial y de la próstata, de la hipertensión me diagnosticaron aquí hace 9 años.

**I: ¿Como le diagnosticaron?**

Pues como uno cuando va a la clínica, me tomaron la tensión ya ya me echaron de ver y ya me mando el doctor medicina para eso, y estuve mejorcito durante un mes, y luego ya tuve que seguir tomándomela porque no se me mejoraba, me la bajaron, estaba normal, pero ya me la tenía. Me diagnosticaron aquí en la clínica de la Palma me hicieron exámenes, una ultra, y ya fui a San salvador para la prueba de la próstata, allí te hacen exámenes más especializados , fui a una clínica privada para la prueba de la próstata, vive allí mi hijo que es el que me ayuda económicamente, yo fui aquí a un doctor y a un laboratorio por Campero privado y yo le dije al doctor que me quería hacer un examen de orina en general y allí me hicieron un examen más especializado y no me salió ninguna infección y ya sabe que a partir de los 40 años hay que chequearse de la próstata, y me hicieron el examen porque estaba un poco molesto y ya antes hace muchos años tenía alterada un poco la próstata, luego me sane porque me dieron un medicamento que me costó 100 dólares todo el tratamiento y hace poco me hice un examen y la tenía también un poco alterada. El primer examen de la próstata la hice hace 13 años en la clínica privada y ya con el tiempo me ha vuelto lo de la próstata.

**I: ¿Pero para el control de la HTA donde se la hacen?**

El control de la hipertensión me lo hacen en la Palma y voy andando unos 20 minutos, como no tengo carro, y a veces me voy en moto-taxi que me cuesta un dólar.

**I: ¿Y ha ido usted alguna vez a otra clínica para el control de la HTA?**

No, solo aquí a la Palma, que es donde me llevan el control y me dan la medicación.

**I: ¿Cuándo acudes al establecimiento de la Palma va algún horario en especial?**

Voy a las 6 de la mañana a la consulta de La Palma, para control con el internista y para los medicamentos; porque ya a las 6:30 empiezan a apuntar. Mas temprano esta FOSALUD, a veces voy a Fosad, a veces cuando no hay un medicamento. Pero lo normal es que yo vaya a la Palma, a pasar control con el internista.

**I ¿Me puede explicar un poquito más como fue cuando fue la primera vez a la clínica de la Palma?**

A ver, no me acuerdo mucho, pero creo que fui porque me agarro una tos, como gripe, y fui a pedir el tratamiento y me tomaron la presión, y es cuando me detectaron.

**3. Episodios agudos de enfermedad crónica**

**I: ¿Alguna vez ha tenido usted alguna emergencia?**

Si, hace unos años iba caminando y me dio un pinchazo y me llevo mi hijo mando carro para que llevaran a San salvador a una clínica privada, y me dijeron que me tomara un examen y ya a los 15 días ya me alivie y ya volvi a la Palma y me hicieron análisis de creatinina, y me dijeron que estoy bien, luego me hicieron la del azuzar y también me dijeron que estoy bien, análisis de orina, me dijeron que estaba mal, pero ya estoy bien, me ha estado viendo el urólogo en el Hospital en la Nueva Concepción. Sigo un poco de dieta, es vedad que tomo gaseosa, no muy seguido. Tengo un poco de molestia por un dolor en zona de los riñones, siento una estacada, que aún me pasa, y de día en día me viene el dolor y me dijeron que era gases en clínica de san salvador en una clínica particular.

Yo cuando tengo una urgencia prefiero ir a clínica privada de san salvador porque en la palma no hay las medicinas que me pueden dar en la privada, solo hay para la tensión, o para el dolor, pero ya una medicina cara ya no la dan, no la hay no mandan medicación cara. Porque también tengo baja la hemoglobina y me dieron el tratamiento, que me cuesta 40 dólares cada caja.

**I: ¿Y cada cuanto va a pasar consulta allí a San Salvador?**

Cada mes voy a San Salvador y también a la Palma, a San Salvador solo cuando tengo alguna emergencia.

**4. Calidad de la atención y percepciones de cuidado**

**I: ¿Que medicinas está tomando usted?**

Para la hipertensión tomo irbesartan, vitamina para la hemoglobina, y para triglicéridos, en la palma me dan tratamiento para triglicéridos y también para el ácido úrico tome tres días, ahora ya no. Ahora solo tomo la de la tensión y la vitamina y la de los triglicéridos, que empiezo esta noche.

**I: ¿Y porque no la ha tomado hasta ahora?**

Porque como estaba tomando muchas medicinas, me daba miedo complicarla. Estaba tomando para eso del estómago, para los gases también.

**I: ¿Y alguna vez ha tenido alguna dificultada para comprar las medicinas?**

No, porque como aquí me les dan regaladas, las medicinas de la clínica privada las tengo que comprar y a veces he tenido problemas para comprarla, y mi hijo porque me hecha la manita.

**I: ¿Y cómo ve usted que es la atención aquí en la unidad de La Palma?**

Buena atención por los sanitarios cuando ya es uno un poco mayor lo tratan mejor. En la clínica privada te tratan mejor porque pago, más mejor que bien, en la Palma también me tratan bien, pero a veces no tiene la medicación.

**I: ¿Y en cuanto al tiempo de espera en La Palma?**

No, no tengo que esperar demasiado porque cuando vas con hora, a la hora que anotan, porque allí hay una hora especifica de que anotan a la gente. En la clínica privada no espero, le atienden a la hora que uno llega y me cuesta cada consulta 8 dólares.

**I: ¿Considera que le brindan la información necesaria sobre su enfermedad?**

Recibo buena información, porque la mejor información es que le caí bien a uno la medicina.

**5. Apoyos y ayudas**

**I: ¿Qué apoyo tiene usted personal, a la hora de manejar su enfermedad?**

Me ayuda mi hijo en la parte económica, tengo que estar alegre, me gusta poner música, ver películas bonitas, ver a las muchachas que pasan.

**I: ¿Con quien suele hablar usted sobre tu enfermedad?**

Y hablo con mi esposa y mis hijos.

**I: ¿Y habla usted con alguien de la comunidad?**

No me gusta lamentar con los demás, como le voy a comentar a alguien que no sea de mi familia, solo con mi familia.

**I: ¿Va algún grupo de la comunidad?**

Si, aquí cuando viene la gente a la tienda platicamos, bromeamos, y así uno disipa los problemas. Voy a veces a la canchita para distraerme, ver a los muchachos jugar. Yo a la iglesia no voy. Yo le sirvo a Dios de corazon. También voy a la Palma para comprar para mi tienda.

**I: ¿Y usted ha tomado alguna vez medicina natural?**

Si porque la gente mayor como yo, si, para los riñones tome medicamento natural que lo compre en la palma,y eso me cayo bien.

**6. Percepción de cambios en el tiempo**

**I: ¿Qué cambios ha notado usted en los últimos 10 años?**

He notado pocos cambios, he notado que las medicinas son más caras ahora que antes, no hay medicamentos de antes si había y eran buenísimos. Había más y había más posibilidad para comprarlas. También como diez años atrás había que pagar la consulta y cuando ibas al hospital, pagabas, y si no tenías todo el dinero, ahí podías ir al seguro social para que le bajara un poco, ahora ya no se paga, y esto es una mejoría.

Lo que ha empeorado es la pobreza, que uno quisiera tener para trabajar y trabajar y que le paguen cómodo, pero a uno más viejo de edad no le dan trabajo, en cambio antes si, en el campo uno trabajaba y le daban, y ahora en una empresa de 40 años para arriba no le pagan.

Antes no había tantas enfermedades terminales como ahora, porque las personas se cuidaban, ahora que, una joven podía tener relaciones, pero para no quedar embarazada toma medicamentos y eso le afecta. Antes venían aquí las parteras, y las mujeres antes no tenían tanto cáncer, ahora toman muchas medicinas que les queda en el útero y eso se queda en el cuerpo, esa masa pule. Ahora se toman más medicinas y eso hace que haya más enfermedades.

**I: ¿Y en relación al sistema de salud que cree que ha cambiado?**

El sistema de salud ha ayudado porque ahora no se paga, es una mejoría.

**I: ¿Estos cambios en que le han ayudado?**

Han ayudado en que yo tenga más acceso al sistema de salud, porque antes uno tenía que pagar la consulta, aunque fuera aquí en la clínica, tenía que tener dinero y también pagar la medicina.

**I: ¿Que recomendaría al Ministerio de Salud?**

Mire yo le voy a decir una cosa, que es lo que yo le diría al Ministerio, yo le podía decir, pero ellos lo que me contestarían es si el gobierno no le da lo que necesita al ministerio, ellos no pueden dar. Yo les diría que haya todos los instrumentos necesarios para una clínica, porque por ejemplo aquí en La Palma quiere dar alguien a luz y no puede, o imagínese que alguien se le muere en el camino y tiene que ser inmediato la emergencia. También que las ambulancias cuando las llames vengan, pero si no tienen para la gasolina no vienen, y pienso que son instituciones del gobierno, y que el gobierno tiene que ayudar a la gente. Pero yo no sé si son los coordinadores de todo eso quien tienen montado todo el invento, ahí no sabría decirle yo.

El gobierno debería de procurar que ninguna medicina no se pague y que este toda toda la medicina disponible para el paciente. Si el gobierno no aporta dinero como lo va a distribuir el ministerio.

## Patient interview 6: Woman with diabetes

Entrevista con: Paciente Diabetes

Fecha: 13/06/18
Lugar de la entrevista: Unidad Comunitaria de Salud Familiar La Palma
Código del informante: PFNV001
Genero del informante: F
Nombre del entrevistador: NV

Fue reclutada del grupo de apoyo de enfermedades no transmisibles

**1. Antecedentes sobre el participante (datos sociodemográficos)**

**I: ¿Conoces tu edad? Si es así, ¿a qué edad tienes?**

Mujer. Tengo 62 años, y nací en la zona alta, lugar las pilas, en el departamento de Chalatenango, yo ahora vivo en El túnel, que está lejos de aquí, y tengo que venir en bus.

En mi casa vivimos 4 personas: dos nietos y una hija; y allí vivimos los cuatro. Los nietos son una hembra y un varón. Una tiene 18 años, y el niño tiene 12, el está estudiando ahorita, está en la escuela. Mi nieta también está estudiando, ella quiere estudiar medicina, dice, tal vez, no está segura, pero le ha dicho un tío que la va a ayudar, porque ellas están jóvenes, con toda su juventud, ellas tienen que seguir adelante, y hay que hacer esfuerzos también.

Yo nunca tuve ni hermanos y hermanas, soy hija única. A la escuela fui un año, un año no más, y aprendí a escribir un poquito solo.

He estado casada pero ahora soy viuda, si ya tengo 32 años de vida; mi marido falleció por la delincuencia de la guerra.

Mi hogar es de adobe, los hombres construyen un cuadro de madera, y ahí construyen las estructuras y ponen prensado el adobe, y así queda todo el adobe, de ese material. Es una casa pequeña, tiene una habitación. Tengo algunos animales, poquitos, pero hay. Pero no cultivamos nada, es chiquito. Tengo agua y electricidad.

**2. Trayectorias de cuidado ante la enfermedad crónica**

**I: ¿Tiene usted una enfermedad crónica? ¿Cuál es?**

Yo tengo diabetes solamente. Bueno al principio fue, yo me sentí bien decaída, y este, a disgusto, que no le anhele nada, así me empezó a mí, entonces yo me preocupe, y vine al médico, y me hizo un examen, y me dijo, “usted tiene azúcar”. Yo vine al médico acá a la unidad de la palma. Esto fue cerca de 13 años que me paso. Ahora si hay una unidad en las pilas, pero antes no había, antes no había, el sitio más cerca era esta unidad.

**I: ¿Cómo y dónde fue diagnosticado/a?**

La razón por la que me disguste fue de repente, igual también por la muerte de mi esposo, que fue desaparecido, fue cosa que no se esperaba, entonces claro yo me sentí mal, me quedo una niña de brazo (año y medio tenía) en ese tiempo, y un niño de tres años. Me quede bastante triste. Mi marido era motorista de una casa grande. Yo he trabajado en el cuidado de mis niños. Yo cuando murió mi marido salí a trabajar, y bueno se arreglar ropa, planchar, lavar, así, se cose bien; y en ese tiempo viva en San Salvador, donde una tía, y allí vivimos. Y luego yo me volví a donde yo vivo ahora porque como allá esta una familia, y mi mama cuando falleció mi marido no quería que estuviera yo solita en la capital, entonces nos vinimos para allá. Mi mama ya está descansando, pero allá en aquella época vivía cerca de mí.

**I: ¿Cuándo empezó a acudir a este establecimiento para el tratamiento y seguimiento de su enfermedad?**

Como ya vivía acá cerca, venia aquí a recoger mis medicamentos, y el examen, aquí en las palmas. Allí en la unidad de las pilas no lo recojo, porque allí a veces tiene, a veces no, entonces de un solo vengo aquí. Ya una viene a hacerse los exámenes, y vengo al mes, vengo a por la medicación, a que me tomen las constantes vitales, y a por el tratamiento, porque uno si no tiene ya ligerito se la sube.

Fíjese que nadie de mi familia es diabético, no hay nadie de mi familia.

Para llegar aquí a las palmas tardo dos horas en llegar, y agarro el bus y me cuesta dos dólares ida y vuelta. Tengo que venir bien tempranito, porque hoy por ejemplo estaba ya a las 7 aquí, si con el primer bus. Vengo tan temprano, porque ahora tenía cita, tenía cita para consulta general, para lo de los huesos, porque tenía un dolor de huesos. Me pusieron unas inyecciones para el dolor y ya estoy mejor.

El tratamiento que tomo para la diabetes es la glibenclamida; la tomo antes de desayuno, después de almuerzo, después de cena, entonces tres veces al día. Y estoy pendiente para tomarla. Mi hija también está pendiente también, no quieren que me muera; porque hay ratitos que uno se siente bien agotado por la enfermedad, le da tristeza a uno, pero al mismo tiempo hay que pedirle a dios que le de fortaleza, y fuerza para vivir. Ya no tengo con tanta energía como cuando era joven, pero hace uno la inteligencia.

Mi hija ahorita no trabaja, está enferma, se descompuso la espalda y está mal, tiene dolor, porque se cayó.

También padezco de la tensión. La tensión empecé hace unos 9 meses, que me hallaron la tensión alta, y eso le pone violencia a uno. Y solamente me agarra violencia. Me la diagnosticaron aquí en la clínica. Me la diagnosticaron porque como te toman tensión a uno, ella en el aparatito como aquel reloj, ahí sale, ahí lo marcan a ella.

**3. Episodios agudos de enfermedad crónica**

**I: ¿Ha tenido alguna urgencia?**

Yo he venido varias veces muriéndome, porque se me sube el azúcar. Una vez se me subió demasiado, otras veces me bajo, no yo no sentí cuando me trajeron aquí. Me desmayé, dice mi hija, y me trajeron aquí (la unidad especializada de la palma), y ya cuando me desperté estaba ya en una camilla, después me tomaron tensión y todo, traía la boca bien reseca, y me pusieron suero, me inyectaron una insulina, y con el rato ya me fui a mejorando.

**I: ¿Y cómo vino hasta acá?**

Para venir aquí mi hija me trajo en un carro, ella no maneja, ella pidió a alguien, no más bien nosotras somos pobrecitas, no tenemos nada. La prestaron el dinero, y la regalaron una parte otra persona, el señor (mirando al cielo), siempre está por uno.

La otra vez que me pasó, aquí andaba yo en la palma, se me bajo otra vez el azúcar, me puse mal, pues ella me dijo ella “vámonos pues para la clínica”, mire es tremendo, es tremenda la vida, no uno cree que es fácil cuando esta joven, pero no, es serio. Yo a dios le pido que yo no me quiero morir, yo llevo una dieta en la comida, yo no me como un pan dulce, café aquí si se costumbre, y tomo un poquito no más, solo para despertar por la mañana que, si no, no puedo, ando bien cansada, dicen que es dañino, pero yo no lo creo, tomar moderadamente, no pasa nada.

Nunca he tenido que ir al hospital, gracias a dios, solamente acá que me han traído, ya para morir, digo.

**4. Calidad de la atención y percepciones de cuidado**

**I: ¿Como ve usted el trato aquí en la unidad?**

En la atención, mire las doctoras son algo pesaditas, pero tiene que hacer uno el que no siente, porque si no, uno no logra la medicina. Ellas tienen carácter, son enojadas, sí, pero como uno no tiene que hacer caso de eso porque si no, uno no lleva la medicina. A mí ya me han dicho, que si no me estoy muriendo que no me ven. Se enfadan porque vengo ya pasada de mi enfermedad, me dicen “usted no se espere hasta que ya parezca que se va a morir, que es eso de que cuando usted va morir viene”, yo solo les digo que “de repente me da el bajón de azúcar, y que le voy a hacer” y es verdad, no les puedo decir de otro modo. El bajón de azúcar no me da por no tomarme las pastillas, me da por que uno tiene problemas, no con uno, pero a veces los zipotes, mis nietos, entre ellos hay discusiones, y uno diabético si es delicado, yo ligerito me siento que me hormiguea hasta la cara y que me sube el azúcar. Mis nietos a veces me gritan, los niños de hoy no son como cuando antes se creía uno que era educado, ahora no, son malcriados y gritones, uno le dice así que se comporten. Se enfadan porque yo les digo así, que no sean malcriados, “¡ay usted cállese abuela!”, me dicen, así son, así son.

**I: ¿Cuánto tiempo espera normalmente para ser atendido?**

Cuando vengo aquí hay que esperar. Aquí anotan a las 8:00, y a veces tengo que esperar más de media hora hasta que me atienden.

**I: ¿Como ve usted la atención y la información que la dan?**

Aquí me atiende cualquiera, porque fíjese que aquí hay bastantes doctores. En cuanto a la información que me dan, aquí uno le dice solo al doctor las medicinas, y me hace el favor de dármelas, y si me duele algo, me recetan otras medicinas, o si me hallan la presión mala, me da medicinas también.

Allí a en mi unidad (unidad básica) no hay medicinas, y para hacer exámenes solo aquí me los hacen, allá no, no tienen los aparatos. Alguna vez si voy allí para poner inyecciones que allí uno, le dan a uno, o la lleva de aquí y allí se la ponen.

La promotora viene una vez por semana, para vacunarle a uno, unas veces contra la fiebre, solo para la vacuna, no para tomar la tensión, por ejemplo.

**I: ¿Le apoya la unidad de salud con su tratamiento?**

Aquí nunca he tenido problema de que no me dan la medicación, allá si (unidad básica cercana). Alguna vez si no hay aquí (en la especializada), vengo los sábados que como esta FOSALUD, y ellos tienen en la farmacia de ellos, ahí ya lo logro. A veces he tenido que comprar la medicación, y a veces si no tengo para comprarla, no la compro tampoco, y me estoy sin medicina y es ahí cuando me pongo mala, esto me ha ocurrido unas tres veces. Si porque como hay tanta gente que viene de cantones, la medicina se termina, porque mire aquí no hay un día que no se llene.

**I: ¿Y cómo ha hecho para comprar la medicina?**

Cuando he tenido que comprar la medicina, tengo que reunir el dinero por poquito, por un dólar, por dos cuotas, porque no tengo ayuda de nadie.

Yo lo que hago cuando no hay ni aquí ni halla, yo a alguien le pido, y luego ya se lo devuelvo, porque venderse no se puede, pero hay algunos que, si tiene el valor de vendérselo a uno, pero para bien de uno tiene que hacerlo. Es como que a uno le prestan algunas pastillas, una vecina, que es amiga y está igual a mí, y luego yo cuando ya tengo le devuelvo sus pastillas. Platicamos de la enfermedad de ya nunca nos vamos a arreglar (se ríe), y la dieta, y que tiene que salir uno a curarse, porque en la casa no puede estar uno sin medicinas.

De medicina natural tomo, tomo la hoja de almendro, que la cojo de por aquí, aquí me la da un familiar, también la raíz del arrayán, para bajar el azúcar, para nivelarlo. Yo sé que es bueno porque las personas que toman me han dicho, pero no se puede tomar cualquier cosa del monte, porque se puede envenenar uno. Me lo trae un tío que me lo regala, aquí él tiene amigos que tienen, y ya le cortan y me lo lleva, ayuda bastante fíjese. Yo se lo he contado al doctor y él me dice “ay por eso tiene usted bien el azúcar”.

**I: ¿Que otras dificultades tiene para su enfermedad?**

También tengo dificultades para venir aquí porque a veces no hay bus, o a veces se ha ido y hay que esperar a otro, y le comienza a agarrar la tarde, y a veces viene, y a veces ya no viene.

**5. Apoyos y ayudas**

**I: ¿Que le sirve de apoyo a usted para su enfermedad?**

Yo para entretenerme, yo salgo, y hasta en la tarde que yo ya estoy en la casa. Yo para entretenerme, en la casa bordo, hay un cuartillo ahí, y ahí me estoy, cierro la puerta, que no me molesten, solo una ventana abierta, ahí me pongo a costurar. También me voy para donde unas amigas, pero no todos los días, porque a veces me llaman para ver si voy a planchar o a lavar, y voy, y con eso gano un poquito de dinerito, pero no mucho.

También hablo con una amiga que es bien contenta, es bien contenta, hace chistes, y me dice “venga bailamos pues”, y yo la digo “no me gusta bailar, baile usted para verla”, y se pone a bailar y me hace sentir bien. Mi hija se enoja porque yo visito la casa ajena, “déjeme” le digo yo, si en la clínica hasta me dice la doctora que salga a platicar con amigas que tenga. Porque eso le da a uno stress, y eso le sirve. Así se le va el tiempo a una, también me pongo a ver a unos perritos, los animalitos también alegran, si se ponen a jugar, a varias formas de como alegrarse uno.

**I: ¿Hace alguna otra actividad en la comunidad?**

También voy a una iglesia, antes era católica y me pase a evangélica, y me gusta. Esta cerca la iglesia, se camina como a hasta ahí (señala como a 10 metros), esta cerquita. Pero ya otra cosa en la comunidad no hay. Ahí es bien sólido. Lo que si le digo es que hay bastantes árboles, pinos, hay cipreses, y es fresco. Yo salgo a caminar por ahí, y se suda, y como el doctor dice que hay que salir a caminar, porque eso le ayuda bastante a uno. Salgo yo sola, porque hay partes que son parejas y hay partes que son bien barrancosas, hay cascada, es bonito, hay pozas, pero retiradas.

Ya otro apoyo no tengo.Hay otras cosas que dependen ya solo de mí, de no comer cosas dañinas, por ejemplo, el pan dulce, el azúcar en el café, o cosas picantes, grasosas, todo eso. Mi principal apoyo es el estar tranquila, no oír cosas que le afecten, para tener mayor salud. Que colaboren con uno a no darle malos momentos, porque para uno, como dice el doctor, uno no tiene que tener ni alegrías, ni cóleras, porque eso le afecta, si, le afecta bastante.

I: ¿Recibe alguna ayuda o subsidio?

Recibo ayudas para el agua, pago tres dólares al mes, para la electricidad también recibo ayudas. Mi hija contribuye también en la economía familiar, un mes cada una.

**6. Percepción de cambios en el tiempo**

**I: Como usted tiene la enfermedad desde hace tiempo, me puede explicar cómo ve usted que era antes el cuidado de su enfermedad.**

Antes se pagaba la consulta, un dólar valía, y a parte del dólar que se pagaba daba colaboraciones uno, decían que era para comprar cosas de la ambulancia, para obtener la gasolina de la ambulancia, y para tener de eso que usan las torundas. Ahora no, ahora ya no se pagaba nada, ahora es gratis, ahora ya con ese dólar que no se da, ya le sirve a uno para una pupusita, verdad (se ríe), si porque como uno se viene de la casa sin tomar nada, antes no antes se pagaba. Eso ha sido una gran ayuda que nos da el gobierno.

Otra cosa es que antes había medicina, unas dos cositas le daban a uno, o ya no había, casi sin medicina se mantenía, ahora no, hoy hay medicina en abundancia, ahora mejor. Otra es que antes le sacaban a uno menos sangre, ahora si le sacan a uno más, un bototo a uno.

**I: ¿Que recomendarías usted para mejorar las unidades de salud?**

Para mejorar es que las enfermeras tienen que ser más agradables, tener amabilidad, no regañarlo a uno, si porque si no si no hace la enfermera y paciente no viene, no se hace nada, no tienen el trabajo ellas.

También cuando una persona es diabética no puede tener cólera, enojos, se enferma más.

## Patient interview 7: Woman with Hypertension + CKD

Entrevista con: Paciente HTA+ERC

Fecha: 13/06/18
Lugar de la entrevista: Unidad Comunitaria de Salud Familiar La Palma
Código del informante: PFNV002
Genero del informante: F
Nombre del entrevistador: NV

1. **Antecedentes sobre el participante (datos sociodemográficos)**

**I: ¿Me puede hablar un poco acerca de usted?**

Tengo 49 años, nací en El Salvador, vivimos tres ahorita mi hija, mi nieto y yo, los demás están fuera. Tengo 4 hijos. Mis hermanos viven fuera, uno en el Salvador y otro fuera. Fui a la escuela hasta sexto. Antes estuve casada, y ahora estoy separada. Yo me dedico a oficios domésticos en la casa. El agua que consumo es agua potable en casa las 24 horas.

1. **Trayectorias de cuidado ante la enfermedad crónica**

**I: ¿Qué enfermedad crónica tiene usted y cuándo se la diagnosticaron?**

Yo soy hipertensa y tengo también un problema renal. Estuve tomando medicamentos hace 17 años y de después me lo retiraron porque me dijo la doctora que no era hipertensa y estuve 5 años sin tomar medicamento, después otros doctores me dijeron que si soy hipertensa y llevo ya tomando los medicamentes para la hipertensión 7 años. La primera vez me diagnosticaron en el Hospital de la Palma, tuve un dolor muy grande de cabeza y me llevaron de urgencia, me llevó mi esposo a un doctor particular, y después el medicamento me lo empezaron a dar aquí, me lo diagnosticaron sobre los 20 y pocos años y era un médico conocido. Se lo conté también a mi familia. Mi mamá y mis dos hermanas tienen la tensión alta. La enfermedad renal me la diagnosticaron hace dos años, yo le pregunte al doctor por qué enferme, porque dicen que es por los químicos, venenos que hay y yo le dije que yo nunca he trabajado en el campo y me dijo que quizá porque estuve tomando medicamentos para la hipertensión y los deje de tomar y yo le dije que estuve 5 años sin tomar el medicamento, posiblemente es por eso. Mi marido tampoco trabajaba en el campo ni tenía contacto con los agrotóxicos.

**I: ¿A qué establecimiento va para el tratamiento y seguimiento de su enfermedad?**

Después del particular fui a Citará y después ya al médico de aquí me diagnosticó. Aquí también llevo el seguimiento en el seguro, mi esposo estuvo trabajando y yo tengo seguro, aunque me pilla bien lejos. A esta unidad intermedia acudo cuando tengo cualquier enfermedad que le da a uno, por un dolor, gripe y también para el medicamento de la presión, y para la enfermedad renal no llevo medicación solo dieta y el agua del grifo esta buena ya le hicieron estudios. No estoy preocupada por la enfermedad renal procuro estar tranquila, es la manera de estar controlada, continúo con mi vida ordinaria.

**3. Episodios agudos de enfermedad crónica**

**I: ¿Alguna vez tuvo un episodio agudo o alguna urgencia?**

Solo tuve urgencia la primera vez que me diagnosticaron eso de la hipertensión, otra vez fui a urgencias por un problema respiratorio y fui al hospital público de la Palma, a la unidad especializada de salud, me pusieron oxígeno, inyecciones, inhaladores, no me hospitalizaron. Solo he acudido a urgencias con el nieto porque se ha caído y se fracturo un bracito y fui al hospital público, en la Palma, fuimos en un carro particular. Cuando lo de la presión fui al médico particular porque fue por la noche, y entonces no había Consejo de Salud.

**4. Calidad de la atención y percepciones de cuidado**

**I: ¿Cuál es tu percepción de la unidad de salud?**

Siempre me ha recibido bien en la unidad de salud, la espera depende de la gente que vaya, al seguro voy con cita y aquí no, voy cuando me encuentro mal. Me atienden bien en el seguro, voy cada tres o cuatro meses y me ve también el ginecólogo, me controlan la tensión, y la enfermedad renal solo con dieta porque todavía no se me ha desarrollado la enfermedad grave, la dieta me la ha puesto aquí el doctor , está el seguro en Aguilar , voy en el bus, por la mañana pago una moto- taxi, el transporte de ida y vuelta me cuesta 30 dólares, lo que más me cuesta el la moto- taxi, el autobús es barato 1,60.

**I: ¿Cómo compara usted las unidades de salud pública frente al seguro?**

En el seguro hay más personal, hay más enfermería y médicos, la ventaja del seguro que para cualquier examen que te haga falta allí se lo hacen a uno, aquí si hacen exámenes, pero si hace falta una mamografía lo hacen en el seguro. En el seguro me atienden bien, y la información que me han dado sobre la enfermedad, sobre la toma de medicación está bien, uno tiene que poner de su parte, hacer caso de lo que te dicen, hay que tomarse la medicación y la dieta, siempre me han dado los medicamentos, si no hay, en otra unidad de salud me lo dan, no falto a mis citas voy sea como sea.

**5. Apoyos y ayudas**

**I: ¿Qué apoyo recibe usted del personal sanitario? ¿Cambiaría algo?**

Todo está bien, siempre me han atendido bien. Hablo de mi enfermedad con mi mamá y mi hija. No conozco ninguna asociación de apoyo de la enfermedad renal en la comunidad, de las dietas también lo hablo con mi mama, me apoya, y me ayuda con la dieta, aunque prácticamente solo la hago yo.

**I: ¿Qué apoyo económico existe en su hogar?**

Con la economía del hogar me ayuda mi hija y también mis otros hijos. Solo he trabajado en mi casa. No falto a mis citas. Recibo ayuda con el subsidio para el pago del gas y sale mas barato, el agua se paga solo dos dólares que es un pago de colaboración, la electricidad sí que es más cara

**6. Percepción de cambios en el tiempo**

**I: ¿Ha percibido algún cambio desde que la diagnosticaron por primera vez hasta ahora en el sistema de salud?**

Nunca he tenido que pagar por los medicamentos, solo he pagado de emergencia por el dolor de cabeza, porque el doctor era particular. Hace años había que pagar un dólar en las unidades de salud, pero eso hace años, a mi madre la diagnosticaron hace años y siempre ha acudido a consulta pública, si he notado mejoría con los ECOS porque así no hay que ir tan lejos, en el seguro la atención siempre ha sido igual, no tengo ninguna queja siempre me han atendido bien. Mi marido era promotor de salud, y es por eso que yo tengo seguro.

**I: ¿Hay algún aspecto a mejorar?**

El transporte sería el único, hoy fui al público a que me dieran el medicamento porque no podía ir al seguro, yo acudo más al seguro porque me han hecho estudios muy costosos, el seguimiento , medicación , análisis de sangre, peso, mamografías, la toma de tensión lo llevo acá, para la tensión tomo losartan y amlodipino para la tensión, en los estilos de vida hago dieta y salgo a caminar aunque no de seguido , al ginecólogo fui cuando estuve embarazada hace 17 años, también me hacen mamografías cada año, y la citología cada año o dos según se tenga.

**I: ¿Usted cree que a nivel de la comunidad la gente se preocupa por las enfermedades crónicas?**

Hay gente que le preocupa las enfermedades crónicas, aquí es una zona tranquila, en la casa he tenido mis 4 hijos. Tuve una intervención en el cuello por un hemangioma cavernoso. El apoyo para las enfermedades crónicas sería bonito que nos reunieran a todos y dieran charlas sobre la enfermedad, consejos y forma de manejar la enfermedad, como sobrevivir con un problema, hay gente que se estresa con un problema, yo intento no preocuparme mucho y mi mamá también mientras no falte la medicación, hay exámenes que se hacen en particulares en la Palma como creatinina, triglicéridos, colesterol, cuesta 5 dólares y proteínas en orina cuesta 15 dólares, yo me hice un examen para la enfermedad renal en clínica particular porque me sentí un poco mal, me estaba tomando un medicamento y se me hincharon las piernas y entonces me hice un examen que me costó 70 dólares, me hice potasio, sodio, el calcio y todo eso... y lo hice particular porque fui por emergencia, ahora el control me lo hago en la Palma en particular porque me lo hacen más rápido. El primer diagnóstico de la enfermedad renal fue en el ECO, me hicieron análisis de creatinina, pero me hice el análisis más rápido en el particular, nadie de mi familia tiene enfermedad renal, he tomado agüita de unas cáscaras del campo que me ha dicho una vecina que es bueno. No tomo ninguna otra medicación más por el problema renal.

## Patient interview 8: Woman with Hypertension

Entrevista con: Paciente HTA

Fecha: 13/06/18
Lugar de la entrevista: Unidad Comunitaria de Salud Familiar La Palma
Código del informante: PFMJ003
Genero del informante: F
Nombre del entrevistador: MJ

**1. Antecedentes sobre el participante (datos sociodemográficos)**

- Me gustaría preguntarla primero un poquito sobre su vida, que me informara sobre qué edad tiene y donde nació para empezar.
- Tengo 70 años nací en un cantón San José de Chalatenango.
- ¿Cuántas personas había en su hogar cuando eras un/a niño/a? ¿Tiene hermanos/as? Éramos 11 en la casa, éramos 9 hermanos.
- ¿Asististe a la escuela? En caso afirmativo, ¿por cuánto tiempo?
- Sí, pude ir solo hasta segundo grado, tenía 8 años.
- ¿Estás casada?
- Acompañada estoy, ahí vivo con mi compañero en la casa, llevamos 44 años juntos.
- ¿Tiene hijos/as? ¿Viven con usted en su hogar? Tuve 8 hijos, pero ahorita tengo 6. Uno se me murió tierno de 8 meses y el otro de 38 años (la participante había compartido en el grupo de autoayuda que a su hijo hace un año que le mataron por el tema de la violencia, decidimos entonces no ahondar más en ello ya que ya lo había compartido y supone algo emocionalmente duro para la participante.
- ¿Cuántas personas viven actualmente en su hogar?
- Ahorita solo mi compañero y yo vivimos en la casa, mis otros hijos...una vive en Tecoluca, otros 2por Chalate, otros dos en Usulután y otro le tengo en los Estados que hace un tiempo le pudimos visitar y nos llevó a un jardín lleno de rosas bien lindo, ¿pero no le pude ver mucho porque andaba trabajando mucho? Hace 25 años que vine acá cuando la Guerra me vine aquí al cantón porque era más fresco y más seguro.
- ¿Cómo es tu hogar?
- Mi casa está allá al lado del polideportivo, al lado de la carretera, no tenemos tierras ni nada, solo es la casa nomás.
- ¿De dónde consume el agua?
- El agua lo cogemos de agua de un manantial, del chorro utilizo para cocinar, pero para beber compramos de botella porque cuando llueve sale un poco sucia, solo la tomamos cuando está clarita.

**2. Trayectorias de cuidado ante la enfermedad crónica**

- Ahora me gustaría preguntarle un poquito más sobre su enfermedad, aunque ya nos ha compartido un poco en el grupo de autoayuda, pero por saber un poquito más a nivel individual.
- ¿Qué enfermedad tiene usted? Yo tengo estrés crónico y dolores musculares que me recorren todo el tiempo el cuerpo es bastante feo...también tengo lo de la presión alta y mala circulación de la sangre en las piernas.
- Si le parece vamos a hablar primero lo de la presión ¿Cuándo y dónde fue la primera vez que buscó ayuda para los síntomas que presentaba?
- Pues yo me sentía como embotada, ahogada, afligida, mareada...eso me pasó en el trabajo que teníamos un puestito que hacía papitas tostadas, eso es lo que yo hacía, he trabajado 27 años allá, pero lo tuve que dejar hace 2 años cuando me operaron de las piernas.
- ¿Cuándo fue esto? ¿Qué pasó después? ¿Cómo y dónde fue diagnosticado/a?

Mi compañero me llevó un día a la unidad de salud con el médico general, ahí me chequearon la presión y me dijeron que la tenía alta y de un solo me dio la enalapril, por entonces no estaba el médico internista aún.

- ¿Y con su trabajo tenía algún tipo de Seguro social? No, porque era trabajo propio, informal que le llamamos acá...trabajaba paradita todo el día por eso ahora que llevo la medias la gente se cree que es por lujo, pero no les explico que es porque las tengo que llevar. El doctor me dijo que las tenía que llevar porque si no se me pueden hacer coágulos y eso se me puede ir al corazón y morirme, así que es por eso que yo las llevo, aunque alguna vez en casa me las quito a ratitos porque me dan mucho calor...

- ¿Le contó a alguien acerca de tu enfermedad cuando le dijeron lo de la presión? No, Bueno a alguna de mis hijos que estaba enferma que tenía la presión alta, a mi mama y hermana, pero de ahí a nadie más, poco me ha gustado contar de mis cosas. Aunque después de la trombosis tuve que estar 4 meses con muletas y ahí sí que me preguntó la gente.
- ¿La unidad de salud le pilla muy lejos de su casa?
- No, a cuatro calles.
- ¿Y aquí mismo recoge la medicación? Sí, bueno, no, cuando me pasó lo de las piernas me empezaron a seguir en Chalate y ahí mismo me daban los medicamentos, pero hace ya un tiempito que me la dan acá.
- ¿Cuánto tiempo se demora en llegar a la unidad de salud? Como lo tengo bien cerquita no tardo mucho, suelo llegar a las 7:30 de la mañana para la consulta del Dr. Burgos que luego me da la receta cada 3 meses ahí suelo salir como a las 11 de la mañana, luego una vez al mes vengo a recoger los medicamentos y eso es rapidito como una media hora tardo. ¿Tiene que pagar a veces? No aquí no pago todo es gratis.
- ¿Qué considera que puede dificultar el manejo de su enfermedad? No no me cuesta controlar lo de la presión mientras tomo el medicamento, aunque a veces me canso de tomar y lo dejo 3 días y vuelvo, aunque el médico me ha explicado que tengo que tomarlo todos los días, a veces se me olvida también, ¿Y porque crees que es que se olvida? Pues mire que yo creo que estoy perdiendo la memoria que ahorita así de casa y ni se dónde dejé las llaves...a ver si voy a tener el Alzheimer ese que dicen...

No mujer no, está usted fenomenal (risas).

**3. Episodios de enfermedad crónica**

- Bueno, pues si le parece vamos ahora al tema de sus piernas, ¿eso cuando fue dónde?

- Pues la primera operación de las piernas fue hace 12 años, antes de lo de la presión, se me tapó una vena y tremendo dolor, vine a consulta acá pero no daban con lo que yo tenía porque por fuera no se veía nada la cosa era por dentro y al final me mandarín con referencia al hospital Nueva concepción Allí me hicieron exámenes y al final me tuvieron que operar, estuve en tratamiento más de un año con ellos. 5 años después se me tapó otra vena y me tuvieron que operar otra vez, siempre en el hospital Nueva Concepción. ¡Hace dos años me golpeé con un cubo acá atrás (se señala el gemelo) y se me hizo como una bola y que dolor qué dolor! Estuve viendo aquí a consulta, pero solo acitenomifen me daban una doctora alta que creo que ya no está...luego me pasaron con otra doctora pequeñita y cuando me vio me dijo que esto iba a necesitar algo más que el acitenomifen, le preguntó al Dr. Burgos y me mandaron para Chalate, ahí estuve 12 días ingresada. Bueno me estuvieron poniendo antibióticos porque pensaba que se iba a resolver, me dieron el alta y a los 8 días que volviera, a los 8 días volví con la misma pelotona y me dijo “uy no, le voy a tener que meter el cuchillo” y ahí me rajó en la propia consulta, me puso antes una inyección y me rajó, luego salió toda la sangre y no me suturó ni nada para que terminara de salir la sangre y ya luego me mandó para casa. Ahí tuve que pagar a una señora que me viniera a pagar porque no tenía cómo transportarme a la unidad de salud, me ayudaba una señora que sabía inyectar y todo, Bueno le iba a pagar pero era amiga y no me cobraba. Luego fui 8 días después al hospital para ver cómo estaba y me apretó fuerte la pierna, me salió sangre, yo ya le dije que no podía dar un paso, él me dijo que iba a estar en control por ellos hasta que me recuperara, siempre en el hospital de Chalate, luego me daban citas más alejadas que ya no sé si la perdí porque ya vengo a recoger los medicamentos aquí. También me vio allí el ortopeda que me dijo que esos dolores de mi cuerpo que ya no se me van a quitar, así que tampoco volví más a la consulta de él porque me queda retirado el hospital.

- ¿Entonces ahora quien la sigue?

-Solo el doctor internista siempre acá en la unidad de la Palma. Siempre me habían dado la referencia desde acá porque si uno va sin referencia no le atienden.

¿En el primer episodio hace 12 años, tuvo que pagar alguna cosa? No no le cobraban a una.

**4. Calidad de la atención y percepciones de cuidado**

Ahora nos vamos a centrar en cuando acude a la **unidad de salud, le recuerdo que la entrevista es anónima y la investigación es independiente,**

- ¿Se siente **bien recibido**? Si aquí atienden bien, solo le dicen a uno, son bastantes los pacientes y queremos atenderles a todos porque hagan un poquito, También explican que si llega emergencia van a tener que verle antes, y eso hay que entenderlo, hay gente que se desespera, pero yo le hago paciencia. Para mí son buenas las enfermeras de acá y el Dr. B.
- ¿Cuánto **tiempo espera** normalmente para ser atendido cuando viene por ejemplo a consulta con el Dr. B? Aquí nos tardamos hasta 3 horas y más porque como le digo vienen bastantes enfermos y a cada uno le pesan, le toman la temperatura, y como hay bastantes pacientes y ásale uno algo tarde, es que el Dr. B es el especialista y tiene muchos pacientes.
- ¿Con cuántos y con qué **personal sanitario** entra en contacto cada vez que acude y por cuánto tiempo? Primero la enfermera que le mide y pesa a uno, le mide la temperatura la presión y luego ya le pasan a la consulta del doctor. Luego da la receta para recogerlo a la farmacia y de ahí para la casa. Cuando vengo a retirar mi medicamento ya vengo solo a eso.
- ¿Considera que le brindan el tiempo necesario de atención todas esas personas que le ven? Si le dan el tiempo a uno- Ya cuando sale de la consulta del doctor la enfermera le explica como tomar y todo y se lo dan en la farmacia se lo explican todo si no lo entiendo, lo atienden bien.
- ¿Considera que le brindan la **información** necesaria sobre su enfermedad? Sí ha sido Buena porque le dicen a uno que es lo que tiene y le explican como tomar el medicamento y todo está bien. Siempre ellos le explican a uno para UE le entienda bien.
- ¿Cuántos medicamentos está tomando ahorita? La de la presión y la aspirinita, También me recetó el calcio y para los huesos y el omega 3 pero esos los compro yo en San Salvador o ahora que estuve en los Estados me los compró mi hijo allá.
- ¿Le apoya la unidad de salud con su **tratamiento**? ¿Cómo? ¿Sugiere alguna medida de mejora con respecto al acceso al tratamiento para su enfermedad?
- Mis hijas me ayudan un poquito cuando no hay medicamentos.
- ¿Porque a veces no hay medicamentos?
- La aspirinita siempre hay, pero la de la presión a veces no y mis hijas me ayudan con eso y con la del calcio y el omega 3. Peor muy poquitas veces ha faltado la de la presión. También el doctor me va a hacienda exámenes porque me ha dicho que tengo la prediabetes esa así que me los va haciendo él para controlarme.
- ¿Ha tenido alguna situación que le ha dificultado **acceder** a la unidad de salud? Cuando no podía andar
- ¿Se acercaron desde la unidad de salud?
- No como ya llevaba el control desde el hospital de Chalate ya preferí que me viera la compañera voluntaria que le decía. Yo no avisé a la unidad de salud, yo creo que quizás no pueden ir a hacer curaciones a la casa, no se no he preguntado, nunca pregunté porque yo cuando estaba así no tenía dinero para mototaxi, y no les gusta llegar hasta mi casa porque hay una cuestecita y no les gusta llegar hasta allá, así que se me hacía mejor estar con esta señora que me venía a curar.
- ¿Cree que hay muchas quejas, así que escuche a la gente en la sala de espera?
- Pues no muchas, pero como le digo, hay gente que no le pone paciencia y no quiere esperar y se va.
- ¿Y usted cómo se siente tratada?
- Bien, si bien, uno le hace paciencia y ya está.

**5. Apoyos y ayudas**

- Y con todo esto que le ha pasado de la presión, las piernas... ¿Se siente **apoyada** por el personal sanitario en el seguimiento de su enfermedad? ¿Hay algo que cambiaría para mejorarlo?
- Sí me dan pastillas para el dolor y para todo…pero ya no solo para medicamentos, en general, solo he tenido que venir una vez por la noche a fosalud y también bien.
- ¿Quién le apoya en el manejo de su enfermedad y cómo?
- Solo mi esposo porque es el único que está a la par mía, cuando tengo mucho dolor el me da masajes con cofal, dragon así distintas pomaditas, y si siento que me hace bien. También tengo mis hijos, pero pasan hasta 3 días y no saben si yo como si yo duermo...un poco simples jejé
- ¿Y este grupo que hemos estado hoy (autoayuda para enfermedades crónicas) siente que puede ser un apoyo para usted? Pienso yo que sí, porque uno dialoga de sus necesidades de sus enfermedades, cada quien lo que uno siente, lo que uno piensa....
- ¿Qué apoyos económicos existe en su hogar? Bueno yo he trabajado siempre para sobrevivir, mi esposo siempre ha estado ahí al lado mío en el trabajo hemos estado en eso 44 años, desde que nos acompañamos, mi hija de los Estados También nos manda una ayudita cuando puede. También tenemos gallinitas en casa y vendo los huevitos, eso lo hago desde que dejé el negocio.
- ¿Recibe algún tipo de ayuda? ¿subsidios? no no, de eso no me dan a mi nada, no tengo pensión ni nada.
- ¿Acá en la comunidad acude a alguna organización o a la iglesia? Sí acudo a la Iglesia todos los días domingo, está como a 5 cuadras, uno se siente bien lleno allá, aunque hay días que no puedo ir por los dolores y la cosa se me enrolla todo así y no puedo hacer nada me quedo ahí solita en la casa.
- ¿Y además de la iglesia a algún otro sitio en la comunidad?
- No no alcanzo, como le digo me gusta estar solita en la casa, así ando.

**6. Percepción de cambios en el tiempo**

· ¿Ha notado algún cambio en los últimos 10 años en cuanto al manejo de su enfermedad? ¿De qué manera las cosas mejoraron o empeoraron? Sí ha habido un cambio porque antes la unidad de salud era allá arriba donde el mercado, y ahorita es más personal tiene más gente, más médicos, Bueno, aunque cuando lo cambiaron hace ya 10 años, pero está mejor, hay más atención.

· ¿Por qué cree que ha podido haber estos cambios? Bueno el cambio ya vino desde que se terminó la Guerra y todo, aunque ahorita la situación está muy dura ahora de inseguridad y eso dicen que matan gente y eso... pero con lo de la salud está mejor porque el lugar antes era más reducido el sitio y ahora es más grande todo, aunque no sé porque es, yo pienso que es por la amplitud, hay más espacio ahorita.

· ¿Cómo han cambiado los costos de atención médica a lo largo del tiempo para usted? Esta mejor ahora que antes porque antes los medicamentos había que comprarlos y ahora es raro el medicamento que no hay y el Gobierno paga todo eso

·       Piensa que se va a mantener o se puede perder? Si yo creo que se va a mantener, siempre que entra uno nuevo promete cosas y dicen que va a mejorar, eso dicen, así que yo creo que se va a mantener.

· ¿Tiene alguna sugerencia para poder mejorar el seguimiento de su enfermedad crónica desde el primer nivel de atención?

- No.no se me ocurre nada.

¿Tiene alguna pregunta para nosotras? ¿Que si van a Volver? Me ha gustado la plática, se siente uno bien con otras personas que nunca ha tratado, me gustaría Volver a verla.

## Patient interview 9: Woman with diabetes

Entrevista con: Paciente Diabetes

Fecha: 13/06/18
Lugar de la entrevista: Unidad Comunitaria de Salud Familiar Perquín
Código del informante: PFML003
Genero del informante: F
Nombre del entrevistador: ML

**1. Antecedentes sobre el participante (datos sociodemográficos)**

- Bueno, muchas gracias por acceder a la entrevista, lo primero que me gustaría preguntarle es su edad, dónde nació y cuantas personas vivían en su hogar cuando era niña.
- Tengo 61 años, gracias a Jehová fui crecida con mis dos padres, pero desgraciadamente a ultimo tiempo tal vez cuando más lo necesitaba tuvimos que separarnos. Yo nací aquí en Perquin, en el cantón Casablanca y tuvimos que separarnos porque mi padre era hondureño y mi mamá salvadoreña y vivíamos aquí y allí, como estaba cerquita. Pero cuando se dio la guerra del 69 tuve que separarme de mi padre, por aquel entonces yo tenía 12 años. Nosotros todos éramos salvadoreños, porque mi mamá venía acá a parirnos, pero teníamos propiedades en los dos países. Nosotros éramos 5 hermanos y con mi padre éramos 7 en el hogar. De mis hermanos tengo una muerta que la mataron de 23 años, pero no cuando la guerra un travieso acá ella estaba lavando y la mataron. Los otros siguen vivos, tengo una hermana que vive en Honduras que ella está amputada de las dos piernas ya porque es diabética. Y los otros que estamos acá en el Salvador, éramos cuatro hembras y un varón.
- ¿Y ahora donde vive usted?
- Acá siempre en el cantón Casablanca, ya no me moví más, acá me casé y todo, ya tengo 44 años de estar casada y he procreado 3 hijos, vivos están los tres y tengo 6 nietos y de esos 6nietos ya tengo 5 bisnietos, esos salieron más inteligentes que yo (risas) porque yo la primera la tuve a los 19 años y ellos no, mi hija mayor la tuvo a mi nieta de 16 años y ella luego ya la tuvo de 26 años porque estaba estudiando. Yo quería que mis hijos se superaran, pero ellos no quisieron, una hace lo que puede.
- ¿Y allá en el cantón cómo es tu hogar, tiene algún terrenito o algo?
- Sí tenemos un terrenito hay finca de café y guineo que es nuestra, bueno de mi esposo y eso lo vendemos, aunque a veces también los regalamos, eso es lo que ocupamos para vivir. Yo he trabajado siempre en casa, que es mucho trabajo cuando vienen los hijos, pero ahorita que ya vivimos solo mi marido y yo vea que pena, yo pienso que cuando uno se queda solo es porque ya se está haciendo viejito, a mi marido por ejemplo hace poco le pusieron el marcapasos que hace poco le chequearon y está todo bien, y yo con el azúcar.
- Ahora vamos al tema del azúcar, solo preguntarle una cosita más sobre su hogar ¿De dónde consume el agua?
- Tenemos agua potable, potable es toda porque viene de cañerías, pero tengo también una privada que es solo para nosotros y la otra que nos da el municipio, tenemos dos aguas, pero para consumir utilizamos la privada. La que pagamos es la potable del municipio, pero la otra no porque compramos el nacimiento y ahí hicimos una cañería que nos llega directamente a casa y esa es la que tomamos y la otra solo para lavar la ocupo, la tenemos porque uno nunca sabe, decidimos comprar la privada porque antes era muy difícil el agua y preferimos estar pagando aunque sea, 3 dólares mensuales pagamos como casi no ocupamos la del municipio, sino sería más que hay contadores, la otra sí que sale directamente del chorro y ni la cierro, ahí pasa saliendo día y noche.

**2. Trayectorias de cuidado ante la enfermedad crónica**

- Bueno, pues ahora sí que vamos al tema de la enfermedad ¿Qué enfermedad es la que usted padece que me estaba empezando a contar?
- Fíjese que yo hace 2 años y medio que me han detectado el azúcar, pero al principio me decía la doctora que a lo mejor solo era nervioso porque fíjese, tanta cosa, que nosotros vivimos la guerra acá 12 años entonces por eso es que uno ha adquirido tanto mal, ahora tengo también olvidos. Entonces yo he ido a pasar consulta y me decía que más que todo son las secuelas de la guerra, aquí había mucho de la guerrilla y cuando venía el ejercito tremenda de montaba, aquí todos se mataban, yo decía que la guerra era más de lenguas, si le caías mal a uno le decían y te mataban, más que ellos decían que era una guerra justa, mentiras son, mírelos como están ahora, esas son las consecuencias. Gracias a Jehová una va pasando con sus dolores angustias y todo, pero está libre.
- ¿Cuándo y dónde fue la primera vez que buscó ayuda para los síntomas que presentaba?
- No fíjese que yo nunca siento nada, bueno ahora luego si es que estaba con unos mareos, pero era por los triglicéridos por lo visto.
- ¿Entonces, cuando es que le diagnosticaron del azúcar?
- Me diagnosticaron lo del azúcar hace dos años y medio porque la doctora me los mandó hacer, pero yo no sentía nada, lo único es que estaba bien gorda, entonces ya salí con eso del azúcar, pero no era tanto, eso me lo dijo la Dra. C., ella es la médica especializada en todo todo lo humano. Antes de eso me veía solo el médico general y él me refirió a la doctora porque de ahí ya le mandan a uno por la obesidad y esas cosas.
- ¿El médico general también la veía aquí?
- Sí sí, yo siempre aquí he estado, salvo cuando me operaron que fui al hospital de Gotera el resto yo siempre acá en la Unidad de salud he estado.
- ¿Entonces ya con los exámenes que le hizo la doctora la confirmaron lo del azúcar?
- Pues fíjese que no las primeras veces que me los hice todavía no lo tenía tan alto porque, ¡ah! otro problema es que a mi hace 5 años se me zafó esta rodilla derecha por eso es que vine y me veía el médico general y luego la doctora que me pidió los exámenes, entonces Sali con un poquito de azúcar, pero no era tanto, pero ahora ya sí que me sale con 400 está bien alto así que eso ya fue hace 2 años y medio. Para estos males solo acá he venido.
- ¿A quién le contaste acerca de tu enfermedad tras el diagnóstico?
- A mi esposo y a mis hijos sí, no me acuerdo si a alguien más.
- ¿Entonces usted desde el principio estuvo en seguimiento por la doctora especialista en la unidad de salud, y ya le puso el tratamiento y todo?
- Si siempre aquí empecé con pastillas, aunque ahora me asustó y me ha dicho que si sigo así me va a tener que poner insulina y eso no lo quiero yo.
- ¿Por qué el cantón donde usted vive le pilla cerca de la unidad de salud?
- Sí, de donde yo vivo está cerquita, ahora como ya me cuesta caminar me demoro 20 mis porque sobre todo al bajar la cuesta es que me molestan las canillas, pero antes tardaba 5-10 minutos, en general siempre vengo caminando salvo que me traiga algún amigo, pero yo caminando suelo venir siempre.
- ¿Acudes al establecimiento/unidad de salud en algún horario en especial?
- Sí vengo una vez al mes porque así es como está puesto dependiendo del control. Yo ya vengo con la cita me deja los exámenes que me voy a hacer para irme chequeando y así, antes que estaba más controlada venia cada dos meses, pero ahora ya vengo cada mes. Cada 2 meses me veía ella, pero cada mes venía a recoger los medicamentos.
- ¿Qué costes tiene para usted acudir a la unidad de salud?
- No no tengo que pagar nada, nada, nada.
- ¿Qué considera que puede dificultar el manejo del azúcar?
- No por ahora a mí no, si he comprado medicinas habrá sido 2 o 3 veces, pero siempre lo suele haber acá. El control directo aquí está bien.

**3. Episodios de enfermedad crónica**

- Y en estos dos años y medio desde que le diagnosticaron a usted lo del azúcar,

¿ha padecido algún episodio agudo/complicación de la misma?

-No, no, bueno este año me agarraron como taquicardias, pero no vine porque era de noche y como a los dos días me tocaba el control con la doctora pues ya me esperé y se lo dije a ella y ya me hizo el examen del corazón y todo.

- ¿Pero por las noches hay también atención acá por Fosalud no?

- Sí todo, pero a mí no me gusta venir a molestar, da miedo andar de noche, no es tan peligroso acá, pero usted sabe no sabe quién entra ni quien sale, ya de noche no es bonito andar, pero gracias a Jehová no hemos tenido grandes emergencias.

- Y hoy que la están poniendo el suerito?

- Lo mandó la doctora porque me ha visto la doctora en la consulta y ha visto que estaba muy alto y por eso es que me ha mandado acá, pero emergencias no he solido tener.

- ¿Y alguna vez ha acudido a algún centro privado?

- Memmi…pues solo para hacerme algún examen porque a veces no los hay acá, los reactivos y eso no siempre los hay. Hoy bastantes exámenes los mandan a San Miguel porque no hay reactivos suficientes. Y eso es un dineral la última vez los mande a hacer todos y me costó 27 dólares, cuando los hacen directamente en la unidad no hay que pagar nada, pero ahora no hay reactivos solo hay para orina, sangre, glucosa y eso general, pero nosotros que somos más delicados necesitamos los que se hacen en el hospital y esos se demoran hasta 10 días. Normalmente cuando me va a ver la doctora me tengo que hacer los exámenes tres días antes de la consulta, pero ahora como no hay ciertos reactivos pues hay que mandarlos a san miguel y eso se demora, en cambio en particular te los dan casi inmediatamente.

**5. Calidad de la atención y percepciones de cuidado**

- Ahora me gustaría preguntarle un poco sobre la unidad de salud ya que la conoce usted bien, recuerde que somos investigadoras completamente independientes y que todo es completamente anónimo.

- ¡De la unidad que barbaridad! Yo ya tengo más de 40 años de estar acá en esta zona, nosotros tenemos el hogar de espera materna que gracias a Jehová lo fundamos, nosotros lo fundamos al ser que había tanta necesidad y tantas muertes de madres. Nosotros somos una asociación de salud que nos llamamos ASPER (asociación para la Salud de Perquin o no sé qué no recuerdo bien las siglas exactas). En el servicio usted lo ve que viene gente de Honduras y usted sabe que la salud no tiene fronteras y entonces viene la gente que viene tranquila a esperar aquí 8 días antes del parto, se inauguró como en 2007-2008 no recuerdo exactamente, se compró un terreno y se utilizó un dinero que nos dieron una fundación de Estados Unidos que ayudó a construir el edificio, el resto lo rellenamos nosotros pidiendo dinero hasta en San Salvador y los clubs también nos ayudaron. Ahora la idea es hacerla más grande porque está pequeña, solo es para 10 y a veces tenemos hasta 25, la mayoría vienen de Honduras.

- ¿Y usted cuando viene acá se siente **bien tratada y eso**?
- Yo sí, la verdad es que sí, en general tienen buen trato, aunque usted sabe que no todas las personas somos iguales y que hay algunas que andan apurada. Alguna cosa que quiere la gente es pasar 2 o 3 consultas de un solo y deben entender que eso no se puede.
- ¿Cuánto **tiempo espera** normalmente para ser atendido?
- No en general no, todo depende de la hora de llegada, yo por ejemplo tengo el horario de las 7:30, luego hay otro turno de 10 en adelante.
- ¿Cuánto tiempo suele quedarse en la unidad de salud cada vez que acude?
- Normalmente cuando vengo a las 7:30 suelo salir como a las 10 porque ella está sola solo está la doctora y la enfermera. Yo normalmente siempre vengo con cita a ver a la doctora, si me atiende de las primeras a las 9 y media uno está listo con los medicamentos y todo.
- ¿Con cuántos y con qué **personal sanitario** entra en contacto cada vez que acude y por cuánto tiempo?
- Primero me ve la secretaria que saca el expediente que es a su vez la enfermera que luego nos talla, pesa y toma la presión y luego ya me ve directamente la doctora y de ahí ya a la farmacia a recoger los medicamentos. Que por cierto (susurrando) con las enfermeras pasa una cosa, que las que vienen de estudiantes son peores que las que están aquí por derecho. También tuvimos problema con un médico que trataba regular a la gente y yo lo fui a decir a la dirección.
- ¿Por qué como hacen para las quejas?
- Nos reunimos cara a cara con el coordinador del centro y algunas enfermeras que también son jefas y la directiva de la comunidad, de ASPER.
- ¿Y el foro nacional de salud también participa de esas reuniones?
- No, con ellos no nos juntamos mucho (risas). Es que a veces entorpecen más que todo, pero de esas cosas no le puedo hablar porque yo tampoco conozco mucho.
- Pero bueno, es para ver cómo responde la comunidad cuando hay esta seria de quejas a la unidad de salud.
- No claro claro, es directamente con uno porque nosotros hemos sido los que hemos luchado para que se reconstruyera la Unidad y que responda como es debido el personal de aquí, a veces os reunimos cada 2 meses a veces cada 3 dependiendo de la necesidad, a veces el coordinador de la unidad llama al director de ASPER y él nos llama para ver quienes podemos ir.
- ¿Y eso de los buzones de sugerencia que hemos visto en la entrada?
- Fíjese que yo a eso no le pongo mucho cuidado, me gusta ser realista, cuando tenemos alguna queja me gusta ser realista y decírselo cara a cara mire que yo esos nunca los he usado.
- ¿Y considera que podrían ser una buena herramienta para poner quejas y sugerencias?
- Bueno, algunas veces, pero como es un buzón vaya usted a saber qué tonteras echan, no es lo mismo que ver y escuchar porque a mí así me gusta, ver oír para poder decir las cosas, no me dijeron, ya eso no sirve.
- ¿Considera que le brindan el tiempo necesario de atención?
- Sí sí no van a prisas, y uno también tiene que entender que no le están atendiendo solo a uno, somos cientos de personas que tiene que ver.
- ¿Considera que le brindan la **información** necesaria sobre su enfermedad?
- Sí, está bien porque fíjese que la doctora cada 3 meses le saca exámenes a uno. Y de información de la información, pues bien, le explican a uno de la dieta y eso, pero algunas veces uno no pone de su parte como me está pasando (risas).
- ¿Por qué a parte de la doctora, le ve algún otro profesional de la unidad como la nutricionista, la educadora o la psicóloga?
- Sí, la nutricionista, no la psicóloga no, pero yo no les hago caso, soy necia (risas) pues yo sé que uno tiene que poner de su parte, pero no es fácil, una tiene muchas cosas que hacer en la casa, les doy la comida hasta a los trabajadores de la finca.
- ¿Y con el **tratamiento** me decía que se lo dan acá en la unidad de salud?
- Sí, la mayoría de veces me lo dan acá.
- ¿Y cuando no, las ha tenido usted que comprar fuera?
- Sí, pero son raras las veces que faltan los medicamentos.
- ¿Y le ayuda el personal sanitario de cómo tiene que tomar el medicamento y eso?
- Si ellos nos lo indican todo en la farmacia, como la doctora les pone cuando tomarlas y eso nos lo indican al darnos el medicamento.
- ¿Ha tenido alguna situación que le ha dificultado **acceder** a la unidad de salud?
- Miré que hace como 12 o 13 años se me fue una hija a estados unidos y a mí me pegó un mareo y luego me entraron ganas de vomitar y luego me dio diarrea y yo caí inconsciente, peor ya hace bastante tiempo, entonces el medico que estaba en aquel entonces vino a verme a la casa con la enfermera, me pusieron tratamiento y toda la cosa y me fueron a ver como 3 veces. Luego cuando me pasó lo de la rodilla sí que me tuvieron que traer mi hija en carro porque no podía caminar, eso fue hace 5 años, me llevaba a gotera porque me enyesaron y todo y me veían en el hospital porque acá no hay ortopedista, era un particular una clínica privada. Pero siempre desde que me dijeron lo del azúcar he podido venir yo o mando a alguien para que recoja los medicamentos, aunque ahora, con eso de que nos tienen que tomar la presión tengo que venir yo, ahora que la tengo mal…que también estoy teniendo que tomar pastillas para la presión.
- ¿Y cuánto hace que le dijeron lo de la presión?
- Uf eso hace ya mucho tiempo, hace como unos 48 años que me dijeron y desde entonces siempre he tomado medicamentos para ello, antes los compraba y ahora me lo ve también la doctora y me dan acá los medicamentos cuando hay,
- ¿Y en general cuando platica con la gente cómo considera que son **tratados** los pacientes en la unidad de salud? ¿Y usted?
- Como le repito somos nosotros los seres humanos que reaccionamos de diferente forma y hay gente que quiere pasar todas las consultas de un solo y yo les digo que no puede ser, que por eso nos llamamos pacientes, porque tenemos que armarnos de paciencia y saber esperar a que le atiendan a uno, entonces más paciencia, más nobleza, pero la gente no toda entiende. A mí la verdad es que nunca me han tratado mal, quizás porque saben que soy parte de ASPER, ese mismo caso del médico que le digo, él se ve que es así, pero es buen médico, se ve que hubo un caso de una señora que salió llorando de la consulta y que por lo visto le dijo que solo bebiera agua y a veces la gente no quiere entender, y a veces por eso se enojaban y se quejaron a la encargada, yo les dije enséñeme la amonestación, porque uno tiene que hacer un escrito que se lo dan a ASPER y se le llamó la atención y ha mejorado la verdad, aquí está todavía, aunque nosotros le dijimos al jefe.
- ¿Y en las quejas trabajan con el FNS?
- ¿EL FNS? Yo no había oído de ellos, es que nosotros aquí como salud y ASPER que es la que ha existido por años, antes era comités muchos nombres, pero siempre hemos estado ahí, por eso para eso sirve uno porque es la comunidad involucrada con la salud.

**6. Apoyos y ayudas**

- ¿Se siente **apoyado** por el personal sanitario en el seguimiento de su enfermedad? ¿Hay algo que cambiaría para mejorarlo?
- Sí, gracias a Dios sí. Ahorita si siento que solo tiene una enfermera y ella debe tener dos pero como la otra muchacha está con un tierno ya no está trabajando, y ahora lo estoy hablando con el director pero porque no quieren poner suplentes y aquí las jovencitas no no..bueno de todo hay pero para que vayan a hacer una cosa que no sirva…fíjese que una vez vine a recoger las pastillas de la presión y me las dio una practicanta y fíjese que me dio una para los que convulsiona y yo que no me fijé me sentí como que se me dormía la lengua pero yo me la tomé, y como tengo una hija que trabaja en salud, les enseñé la pastilla y me preguntó quién me la había dado que no son las correctas, yo le dije la practicanta y es por eso que ya no me fío de ellas porque me pasó. Yo se las fui a enseñar al director y me confirmó que estaban equivocadas y amonestó a la practicanta tenga más cuidado, sino entiende la letra pregúntale a una de las mayores, pero eso no se puede hacer, fíjese que susto.
- ¿Quién le apoya en el manejo de su enfermedad y cómo?
- Mis hijos y mi esposo. Mi hija que se fue a los Estados sigue allá, ahora tengo uno y los otros dos acá.
- ¿Participa en algún tipo de organización en su comunidad? ¿Qué tipo de apoyo le brinda?
- Ahorita solo en Asper, yo ya de más antes de que se hiciera la casa materna en 2007, yo empecé de la guerra para acá, desde el 93-94 tuvimos que pedir ayudas porque como la unidad se dañó por la guerra, se pidió pues al gobierno y a instituciones. Antes teníamos la unidad acá afuera y pagábamos el alquiler del terreno 150 colones al mes en una casa particular en el tiempo que se reconstruyó esta tras la guerra. Antes participé en otras cosas como en las directivas del agua de la comunidad, en una cooperativa de insumos orgánicos para las tierras para el café, pero ya no vamos, por los males, y que la gente es bien irresponsable y uno se desmotiva. Antes también estuve en la escuela y en el instituto cuando estaban mis hijos allá, acá así ha sido. En el verano hacíamos barbacoas para comprar la tierra y pagar el instituto, desde la directiva de padres primero por particular que pagábamos nosotros al maestro y todo. Ahora la gente todo lo quiere del gobierno y nosotros hemos luchado para tener lo que tenemos.
- ¿Y en estas organizaciones había mujeres y hombres?
- Sí, pero siempre más mujeres, porque como hay hombres que son a veces demasiado machistas. Y lo bonito es eso involucrándose, nosotros íbamos mi esposo y yo, a él le gustaba más hacerse de la directiva y yo más colaborar, bonito pero ya todos los hijos crecieron y los nietos también…Ahora ya solo nos quedan los bisnietos chiquitos y mire, con esto de la delincuencia hay que aceptarlo que somo los padres los responsables y culpables que esto esté así en primer lugar después de la guerra cuando se fue la gente dejaron sus hijos a la deriva y mandaron dinero y usted sabe que el dinero es tentativo, no había ninguna persona responsable y no les enseñaron a utilizarlo mal. Miren paz no va a ver porque no la habrá hasta que no venga el Señor, porque los militares que se fueron dejaron sus hijos, los guerrilleros igual, así que paz no va a haber. Porque Dios no se mueve, pero tiene a quien mandar, a su hijo, yo soy testigo de Jehová, estudiante por ahora porque aún no me he bautizado, pero confío, confío en que él me va a aceptar como su amiga.
- ¿Y entonces con eso tendría algún problema con la donación de sangre no?
- Ay sí, y es que uno según va leyendo la biblia se da cuenta de que es una realidad, eso no es bueno, máxime ahora como está la ciencia fíjese que hay operaciones que la sangre misma de uno la van limpiando, así tienen que operarnos a nosotros y sino pues hasta donde se llegue, porque solo él nos da la vida y sólo es nos la quita. Es bonita esa religión, pero estricta, yo tengo poco tiempo de estar en esta religión hace 7 años antes decía que era católica pero solo de palabra, pero ahora sí, será porque estoy mayor pero sí me gusta, salimos a predicar y eso es necesario, por eso vino su hijo también a predicar, porque hay miles de sectas, pero son pocas las que realmente quieren hacer la palabra de Dios en la tierra. (susurrando) Yo antes también intentaba a hacer lo mejor, por ejemplo, en la guerra si venían unos u otros tenía que hacer comida a quien fuera porque si no nos mataban, por eso, porque los que realmente vivimos la guerra ni hablamos de ello, porque estamos asqueados, porque los que hablan de ello, que la mayoría son jóvenes esos ni existían yo no quiero ni oír hablar de ello.
- ¿Existe algún grupo de apoyo específico para su enfermedad crónica?
- ¿Como que le den terapia a uno? ¿Un club? Eso no lo tenemos mire, eso sería bueno, hay intercambio de relación, estaría bueno. La vez pasada estábamos en eso, pero como a uno no le alcanza el tiempo…
- ¿Recibe algún tipo de ayuda? ¿subsidios?
- Solo el subsidio del gas recibimos.

**7. Percepción de cambios en el tiempo**

- ¿Nos puede describir si existe algún cambio en los últimos 10 años en cuanto al manejo de su enfermedad?
- Si, ya hace como 10 años que está esta doctora que ella es más que todo para los crónicos. Y antes la gente lo que podía, antes se pagaba, pero era una cosa mínima, la cuota voluntaria, un colón, algo simbólico y fíjese que era bonito. Ahora que todo es gratis, la gente es tan grosera que viene todita la familia a pasar la consulta y sacan un montón de medicamentos para botarlos que luego se ven los blísteres en la carretera. Era barato lo que se pagaba como unos 15 centavos de dólar.

- ¿De qué manera las cosas mejoraron o empeoraron?

- No ha mejorado nada nada, imagínese que con todo esto de la dolarización lo que valía n colón paso a ser un dólar y todo pasó a ser más caro, el negociante es el que ganó. En la unidad de salud ha costado verla cómo está. En algunos aspectos ha mejorado por ejemplo la asistencia con eso de que hay consultas de día y de noche, aunque con las medicinas como le digo a veces hay que comprarlas.

- Pero el hecho de que sea gratuita la asistencia entonces ¿usted cómo lo percibe?

- Pues es que ver el abuso de la gente pues duele, si algún día vuelve a ser pagado va a ser más fácil, dependerá del gobierno que venga, pero es que si las cosas no tienen un precio no se valoran entonces eso es lo que pasa, y más cuando no hay una buena administración, tal vez no acá porque acá es un sitio serio, pero hay lugares que hay muchos deterioros.

- ¿Por qué cree que ha podido haber estos cambios?

- Eso yo no lo entiendo porque es porque era bonito como le digo porque uno aprendía a valorar, y ahora como es gratis la gente no lo valora para nada, la gente es injusta para mí. Aunque las cosas no se tiene un amor por las cosas, aquí se pinta la unidad cada año y mire como está no se valora, y la educación como le digo, los padres son responsables de darles esa educación a sus hijos para que aprendan a valorar lo que hay y hoy en día no se hace.

- ¿Cómo han cambiado los costos de atención médica a lo largo del tiempo para usted?

- Particular es bien caro, pero acá no se paga nada nada, ni medicamentos ni nada.
- ¿Tiene alguna sugerencia para poder mejorar el seguimiento de su enfermedad crónica desde el primer nivel de atención?
- Tal vez pagando como antes se valoraría más. Como le digo lo que a uno le cuesta lo valora. Por ejemplo, este suero con todas las vitaminas y todo que tienen por particular te cuesta más de 5 dólares y aquí la gente no lo valora. Yo he vivido mucho con la gente y he aprendido muchas cosas y uno pudiendo colaborar en las cosas tiene que colaborar para poder dar un servicio para todos, pero no, hay alguno que no hacen caso y están con el teléfono, ay esos teléfonos, a la gente se le olvida el compromiso y eso es la mayoría. Para toda la atención deberíamos trabajar en gran parte con la educación del ser humano porque se ha perdido mucho valor. Nosotros quisiéramos seguir agrandando este centro porque es necesario, y los que vienen en la noche es otra cantidad porque aquí no se cierra, que siga creciendo de espacio y buscar personal adecuado porque a veces ese problema lo hemos tenido en todo el país, a cualquiera que se presenta le coge, a veces por colores políticos y yo no soy partidaria de eso. Ah bueno y mejorar lo del laboratorio para que tengan todos los reactivos para poder hacer todos los exámenes aquí. Ay hay muchas cosas que se necesitan y muchas cosas bonitas también así que debemos darles gracias a Jehová.
- ¿Tiene alguna pregunta para nosotras? Agradecerles la gentileza que tienen por venir a vernos. Muchas gracias.

## Patient interview 10: Man with Chronic Kidney disease

Entrevista con: Paciente ERC

Fecha: 13/06/18
Lugar de la entrevista: Unidad Comunitaria de Salud Familiar La Palma, Caserío Chalatenango
Código del informante: PMMJ004
Genero del informante: M
Nombre del entrevistador: MJ

**1. Antecedentes sobre el participante (datos sociodemográficos)**

- Buenas tardes, lo primero que nos gustaría preguntarle es sobre su edad, dónde nació, cuánta gente vivían en el hogar cuando era niño, un poquito todo eso.
- Buenas tardes, mi edad es de 58 años, nací en el puerto de la Libertad. Cuando era niño, éramos 6 hermanos, yo soy el cuarto, vivíamos con mi papá, mamá y mi abuelo, así que en total éramos 9 en la casa.
- ¿Y pudo asistir a la escuela?
- Sí, pero solo hasta tercer año, hasta los 8 años, eso en Honduras, porque me llevaron pequeño para allá no me acuerdo con qué edad porque estaba pequeño, pero regresé con 10 años. Nos fuimos a Honduras por el trabajo de mi papá en la tierra que fuera bueno para trabajar allá compro terrenos y cultivó caña, café piña y luego regresamos a Chalatenango cuando la guerra del fútbol, y volvimos a El Salvador a Dulce Nombre de María de dónde nos sacó la otra guerra, la guerra civil, y ya vinimos a caer acá al caserío.
- ¿Estás casado/a?
- Hace 39 años que estoy casado (mira a su esposa y se ríen).
- ¿Tiene hijos/as? ¿Viven con usted en su hogar?
- Tenemos 6 hijos y 6 nietos, somos bisabuelos ya tenemos dos bisnietos (una hembra y un varón). Todos mis hijos siguen vivos gracias a Dios.
- ¿Y Cuántas personas viven actualmente en su hogar? Ahorita en la casa vivimos mi mujer y yo, mi hija con su compañero, un nietecito y otra nietecita ésta del segundo hijo. Tenemos una hija mayor, después dos varones y después 3 hembras.
- ¿Y en la casa de dónde toman el agua?
- El agua la tomamos del chorro, de un proyecto de acá de la comunidad. Es de un nacimiento natural, de esa mima del chorro tomamos, está tratada.
- ¿Y tienen algún terrenito ahora? ¿Cultivan algunas tierras?
- No, alquilamos el terreno, pero cultivamos la milpa, una parte es de mi hijo que compró por acá. Ahorita cultivamos maíz en la milpa y frijol (que se le llama frijoleada) es para nosotros no lo vendemos.
- ¿Y echan pesticidas?
- Sí, porque aquí más que todo al frigo se le pone n bichito que le pone negro así que por eso le damos una bombeada cada 8 días en esa época.
- ¿Utilizan algún equipo especial? ¿Porque dicen que puede hacer daño no?
- Sí lo recomiendan porque dicen que puede producir la enfermedad renal, pero a veces uno no lo utiliza por el calor, pero si tengo compañeros que han padecido la enfermedad y algunos han muerto. Aquí lo dejamos en una bolsita en casa (me enseña la bolsa y la máquina para bombeo que tienen en la entrada de la casa).

**2. Trayectorias de cuidado ante la enfermedad crónica**

- Si le parece ya pasamos a preguntarle un poquito sobre su enfermedad crónica, gracias por contarme un poquito sobre su vida. ¿Qué enfermedad es la que tiene usted?
- Yo lo que tengo es diabetes e hipertensión.
- ¿Y desde hace cuando cada una?
- No tengo una fecha exacta, pero más o menos hace 8 años.
- ¿Las dos a la vez le diagnosticaron? ¿Cómo fue eso?
- Sí las dos a la vez, yo fui porque sentía más que todo con la orinadera y como cansancio y por eso fui a la unidad de salud y el doctor me pidió los exámenes y al recibir los resultados me dijo que era diabético, que no me afligiera pero que tenía que tomar los medicamentos y cuidar la dieta, pero gracias a Dios por ahora estoy bien.
- ¿Y lo de la presión como se lo diagnosticaron?
- Bueno, me tomaba la presión la enfermera en la unidad de salud y también me dijeron que era hipertenso.
- ¿Fue a recibir los exámenes solo o con alguien?
- Yo solito fui a recogerlos.
- ¿Y a quien se lo contó?
- A mi mujer y a mis hijos. A la familia más que todo a nadie más.
- ¿Así que desde el diagnostico ya le vieron en la Unidad de salud especializada de La palma? ¿Y los medicamentos y todo se lo dan allá?
- Sí, desde que me dijeron lo del azúcar y la presión siempre voy a la Unidad cada vez que el doctor me deja la cita, según esté me deja para cada mes si estoy mal y si estoy bien hasta cada 3 meses me ve dependiendo de cómo tenga el nivel del azúcar. Él me dice cuando me quiere ver y ahí pido la cita a la hora que más me convenga, normalmente la pido para las 12:30 porque es cuando yo ya he terminado de trabajar.
- ¿Y cuando le dan la cita para esa hora, cuanto tiempo tarda desde que llega a la unidad de salud hasta que sale?
- Salgo como a las 3 de la tarde, pero depende a veces salgo hasta la hora de haber llegado, pero a veces el doctor B., el especialista que es el que me ve tiene más pacientes y se demora algo más.
- ¿Y le cuesta algo?
- ¿La consulta y los medicamentos?
- No nada.
- ¿Y cómo va a la unidad de salud?
- En mototaxi desde acá que está cerquita, me cuesta 1,5 doloras ir y regresar.
- ¿Y qué es lo que cree que más le dificulta un buen manejo de su enfermedad? Pues fíjese que lo que más se me dificulta es el control en la comida, porque es una enfermedad que a uno le da mucha hambre y eso es lo que más me cuesta controlar. Porque llegar es fácil, esta cerquita y siempre hay mototaxis que le llevan a uno porque yo no tengo carro propio, pero no tengo problema para llegar.

**3. Episodios de enfermedad crónica**

- ¿Y en estos 8 años que lleva usted con la enfermedad del azúcar y la presión, ha tenido algún susto relacionado con ellas?

- Sí varios, porque se me ha elevado mucho el nivel del azúcar y la presión y me ponen suero, bueno yo así le llamo, el doctor le llama solución salina, me lo ponen en la vena y oxígeno también me ponen.
- ¿Y siempre que ha tenido esos episodios donde ha ido?
- Siempre a la unidad de salud, bueno una vez una amiga que se afligió mucho me llevó a una clínica privada, pero por fin terminamos en la unidad de salud porque es donde está el medico que me llevaba. Allí sí que tuve que pagar, porque me hicieron un electrocardiograma y todo, me pusieron una inyección que también tuvimos que pagar, como 25-30 dólares en total, no recuerdo exactamente, pero al final nos fuimos a la unidad de salud, esto fue como hace 3-4 años, pero en general siempre en la unidad de salud. Siempre me ha pasado porque me sube mucho el azúcar.

**4. Calidad de la atención y percepciones de cuidado**

Ahora me gustaría preguntarle sobre la **unidad de salud,** le recuerdo que somos completamente independientes de la unidad de salud así que no se preocupe de contarnos también las cosas que no le gusten tanto para que así, gracias a sus opiniones se pueda mejorar, además usted la conoce bien que lleva 8 años lleno allá así que es especialmente importante que nos cuente cómo lo ve.

- ¿Cuándo llega a la US **como se siente recibido**?
- A veces cuesta porque hay pocas enfermeras o doctores y a veces le toca esperar a uno porque llegan emergencias y demás y hay que hacerle paciencia. Mis hijas por ejemplo han venido alguna vez los fines de semana con salud y se han demorado mucho en atenderle. Pero para mí en la consulta con el Dr. Burgos no es muy difícil porque ya tengo la hora y aunque a veces se demore un poco siempre me ve, es más difícil cuando uno va sin cita para consulta general.
- ¿Cuánto **tiempo espera** normalmente para ser atendido por su doctor?
- A veces como máximo tres horas, pero muchas veces menos, depende de los pacientes que tenga delante.
- ¿Cuánto tiempo suele quedarse en la unidad de salud cada vez que acude?
- Pues después de que me vea el doctor hay que sacar nueva cita, pedir el laboratorio, así en total 4 h oras, si pilla la hora del almuerzo y no está por ejemplo la que da la medicina hay que esperar más.
- ¿Con cuántos y con qué **personal sanitario** entra en contacto cada vez que acude?
- Pues normalmente como voy con mi cita, me recibe la enfermera que me toma la tensión y todo eso, luego me ve el Doctor especialista y luego a la farmacia y pedir la siguiente cita con las secretarias.
- ¿Considera que le brindan el tiempo necesario de atención?
- Sí, el doctor siempre me atiende el tiempo que necesito la verdad, a veces necesito más y a veces menos pero siempre me atiende lo que necesito.
- ¿Considera que le brindan la **información** necesaria sobre su enfermedad?
- Pues yo creo que a veces quizás no por no afligirlo a uno, no le dicen realmente las consecuencias de la enfermedad, como cuanto puede durar uno con la enfermedad y eso. Bueno sinceramente nunca le he preguntado porque no quisiera saberlo.
- ¿Y por ejemplo en estas veces que se ha puesto más malito con las subidas de azúcar le han explicado porque se produce y como evitarlo?
- Sí me explicaron que tengo que hacer dieta y salir a andar. Y la verdad es que sí que lo estoy haciendo porque me amenazó con la insulina y ahora estoy saliendo a andar y controlando más la dieta. Te doy la última dosis de tabletas me dijo, ya lo siguiente sería la insulina sino conseguimos controlar el azúcar. Así que ya llevo 4 meses de caminar y comer sano, no estoy comiendo tantas grasas, pan, azúcar, sal tampoco. Y yo he sentido una gran mejoría la verdad. De hecho, la última vez que me he hecho el control de la glucosa he tenido 92 y la presión 120/100 así que mucho mejor que otras veces, me ha dado la enhorabuena la próxima cita la tengo el 22 espero seguir igual de bien.
- ¿Y cómo hace con el **tratamiento le apoya alguien**?
- Yo solito me lo preparo. (Interrumpe su mujer: Yo le explico que uno tiene que estar pendiente de ver cuantas llevas y recordar que se toma cada día las que debe, ya le he dicho a mi nieta que le haga un cartelito que indique cuando tiene que tomar cada una, así como ella tiene el horario de clase porque a veces los sustos de subida de azúcar han sido porque no lo ha tomado…). Ahora tomo dos metforminas al día y sí tengo que mejorar que no se me olvide. ¿Sugiere alguna medida de mejora con respecto al acceso al tratamiento para su enfermedad? No se me ocurre cómo puedo mejorar, quizás sí lo del cartelito de la nieta estaría bien, ya luego le voy a decir.
- ¿Ha tenido alguna situación que le ha dificultado **acceder** a la unidad de salud?
- No en general no he tenido problema, si no veo mototaxi voy a pie.
- ¿Y cuándo se ha puesto más malito, le han llevado o cómo?
- Siempre me han llevado mis hijas si estaban acá o mi mujer en el mototaxi.
- Y cuando está en la sala de espera de la unidad de salud, platican con la gente imagino… ¿Cómo considera que son **tratados** los pacientes en la unidad de salud? ¿Y usted?
- Pues a veces se oye que la gente se queja, más que todo por el tiempo de espera. Yo en mi caso cuando no voy a hacerme los exámenes del azúcar desayuno bien antes por si acaso me toca esperar y le hago paciencia. Yo no me siento mal por la espera, como el día que saco la cita es para eso nada más pues ya estoy hecho a la idea, como puedo ir para allá entiendo que no va a venir el medico a verme, solo cuando uno está mal por ejemplo como le pasó a mi nietecito el otro día que estaba con calentura y vino el medico a verle a casa peor yo siempre voy y entiendo que a veces hay que esperar y le hago paciencia.

**5. Apoyos y ayudas**

- ¿Se siente **apoyado** por el personal sanitario en el seguimiento de su enfermedad?
- Pues yo sí me siento bien apoyado por la unidad de salud, se ve que están pendientes de uno. Por ejemplo, a mí se me olvidan a veces las citas y le vienen a decir a uno con alguien que pase cerca de la casa, la promotora o quien sea. Siempre están ellos pendientes de uno.
- ¿Hay algo que cambiaría para mejorarlo?
- Yo creo que para mí está bien, no cambiaría nada.
- ¿Quién más le apoya en el manejo de su enfermedad y cómo?
- Mi familia me apoya siempre y el personal de salud. Mi mujer por ejemplo tiene la misma enfermedad que yo así que nos intentamos ayudar con la dieta. A mí me da hambre la enfermedad, pero a ella no…y me dice que no me coma más tortillas y eso. Quizás es otra clase de enfermedad del azúcar que tenemos y por eso a mí me entra hambre y a ella no, y eso que lleva 10 años con la enfermedad.
- ¿Participa en algún tipo de organización en su comunidad?
- Pues antes participaba en la Adeco, asociación de apoyo comunal, cada 2 años cambia la directiva y yo ya cambié, porque hay más gente que lo puede hacer mejor que uno, o porque hubo problemas en el periodo que estuvo uno y no quiere volver a meterse en problemas, pero sigo apoyando en los proyectos que organizan y participo peor ya en directiva ya no. Ya en otra cosa no participo, bueno voy a la iglesia, pero no participo activamente, vamos a la iglesia evangélica los fines de semana.
- ¿Y de apoyo económicos que hay en su hogar?
- Pues nuestros hijos nos apoyan, sobre todo los que viven en Estados Unidos nos mandan una ayudita. Pero yo sigo trabajando de carpintero cuando puedo, siempre he trabajado en eso, trabajo en una empresa que me hace pedidos y yo los hago y también así a gente particular. Mi mujer trabaja en casa no más.
- ¿Recibe algún tipo de ayuda? ¿subsidios?
- Nos ayudan para el gas, pero de ahí nada más.

**6. Percepción de cambios en el tiempo**

- ¿Nos puede describir si existe algún cambio en los últimos 10 años en cuanto al manejo de su enfermedad?

- Sí antes se pagaba, pero ya hace tiempo, un colón se pagaba, pero ya luego no se ha pagado eso que llamaban la cuota voluntaria, pero de ahí la eliminaron como hace 10-12 años, como en 2009.

· ¿De qué manera las cosas mejoraron o empeoraron?

Para uno han mejorado, porque por ejemplo nosotras tuvimos que llevar a una hija al hospital porque tuvo un accidente y le tuvieron que hacer una operación bien grande de los brazos, el estómago, etc. Y era cuando todavía se pagaba y nos cobraron como 800 que al final nos hicieron una rebaja a 500 pero fue mucho pisto para nosotros, fue como hace 16 años ello.

· ¿Por qué cree que ha podido haber estos cambios?

- Quizás por la presión del pueblo. No sé bien eso quien lo eliminó, el gobierno de Funes creo que fue.

· ¿Cómo han cambiado los costos de atención médica a lo largo del tiempo para usted?

- Ahorita ya no pagamos nada para la consulta ni por los medicamentos en la unidad de salud, desde hace 10 años más o menos.

· - ¿Tiene alguna sugerencia para poder mejorar el seguimiento de su enfermedad crónica desde el primer nivel de atención?

- Que el doctor viera menos pacientes para que no tuviera que demorarse, que hubiera más personal de salud, especialistas más que todo, internistas y por ejemplo ginecólogo que solo viene dos veces por semana y solo ve a embarazadas, eso fuera bueno que hubiera uno permanente que viera toda la semana como antes. Y medicinas siempre suele haber, es muy raro que no halla, son pocas las medicinas que no da el ministerio, la mayoría de medicamentos los tienes, yo de los que tomo nunca he tenido problemas para tener mis medicamentos.

¿Tiene alguna pregunta para nosotras? Que ya no se vayan y que vengan más seguido a vernos, luego ya a uno le hace falta estarlas viendo.

## Patient interview 11 : Man with Hypertension

Entrevista con: Paciente HTA

Fecha: 13/06/18
Lugar de la entrevista: Unidad Comunitaria de Salud Familiar Perquin
Código del informante: PMML004
Genero del informante: M
Nombre del entrevistador: ML

**1. Antecedentes sobre el participante (datos sociodemográficos)**

E: Bueno, pues, buenos días, vamos a comenzar con la entrevista y quería preguntarle si conoce usted su edad, ¿qué edad tiene?

P: 80.

E: 80 años. Muy bien. ¿Y dónde nació usted?

P: Yo nací en *Curino*

E: Ajá, ¿y dónde está eso?

P: Aquí para al lado.

E: Ajá. ¿Aquí en el departamento de Morazán?

P: Sí, pero ahora estoy aquí en *Curino.*

E: Ah, vale. ¿Pero usted nació acá en este departamento de Morazán?

P: Sí.

E: Ah, muy bien... ¿Y cuántas personas había en su hogar cuando usted era niño? ¿Cuántos hermanos y hermanas tenía?

P: Mmm, yo tenía…éramos seis…tres varones y tres hembras.

E: Ah, qué igualados estaban. Muy bien. ¿Y vivía usted solo con sus hermanos o vivían también con más gente en casa? ¿Con algunos tíos, sobrinos?

P: No.

E: Sólo los seis.

P: Sí.

E: Ajá. ¿Y usted fue a la escuela cuando era pequeño?

P: No…

E: ¿No fue?

P: No.

E: Ajá. ¿Y ahora…ha estado usted casado?

Sí.

¿Y ha tenido usted hijos?

Sí.

¿Cuantos hijos ha tenido?

Tres.

¿Cuantos niños y cuantas niñas?

Mire, nosotros teníamos siete, pero murieron…

¿Cuatro?

Cuatro. Quedaron tres.

¿Cuándo eran chiquititos murieron?

Sí…

¿Sí? ¿Qué edad tenían cuando fallecieron?

Uno tenía diecinueve meses.

Uf…

Estaba malito ya.

¿Estaba tiernito?

Sí, era hembrita.

Ajá. ¿Y los otros también cuando eran chiquititos? ¿Y los otros también fallecieron cuando eran tiernitos?

Sí, tiernitos.

Tiernitos todos.

Todos.

Y los tres qué son. ¿Cuántas hembras y cuantos varones?

Una.

Una hembra y dos varones.

Sí.

Ajá. ¿Y viven ellos ahora ellos con ustedes?

No, están aparte.

Están aparte. ¿Y entonces ahora quién vive en su casa?

Mire, estoy ahora allí ahorita, estoy con mi hija, la esposa mía y una nuera.

Ah. ¿O sea que son cuatro?

Sí, y un cuñado.

Un cuñado también.

Somos cinco.

Ah, cinco. Muy bien. ¿Y tienen niños pequeños también en la casa?

Sí… tengo un nieto chiquitillo.

Ah, ¿sí?

Sí.

Qué bonito.

Tengo un nieto.

¿Y qué edad tiene el nieto?

No tiene un año todavía.

Ah, o sea que es bien pequeñito.

Sí, bien pequeñito.

(Risas)

Y me puede describir un poquito como es su hogar. ¿Cómo es su casa? ¿De qué está construida…?

De adobe.

De adobe, ajá. ¿Y tiene usted agua en casa?

Sí.

¿Sí? ¿Tiene chorro entonces?

Sí, como de manguera, en pozo.

Ah. ¿Y ustedes van a recoger el agua al pozo y luego se la traen para casa?

No, ahí mismo… con manguera.

Ah, muy bien. ¿Y esa es el agua que beben y cocinan, o solamente para cocinar?

Par’ tomar.

¿Para tomar y para cocinar también?

Sí.

Muy bien.

¿Y paga usted algo por el agua?

No.

¿No paga nada?

No.

Ajá. ¿Y por la electricidad tiene que usted pagar algo?

Sí.

¿Cuánto paga más o menos al mes?

A quince dólares el mes.

Bastante entonces.

Y es poca la...

¿La luz?

Sí.

Memmi, eso sale.

¿Y el gas? tiene usted que pagar gas?

Mmm… Sí.

Ajá. ¿Y cuánto le sale el gas más o menos?

Como nos dieron….

¿Un subsidio?

Ajá.

Ajá.

Sí.

Muy bien. ¿Entonces no tiene que pagar nada?

Como no, como según. Son cinco dólares.

¿Ah, para cada mes?

Sí.

¿Y usted en qué ha trabajado, Miguel?

Yo… jornalero de Milpa

Ah, jornalero de Milpa*.* ¿Muchos años?

Sí, muchos años.

¿Cuándo comenzó entonces a trabajar más o menos?

Mire, yo tenía…unos…doce años […]

¿Y cuándo pasó la guerra, usted tuvo que irse a otro lugar o se quedó acá?

Sí, nos fuimos a otro lugar.

¿A Honduras o a…?

A Usulután.

A Usulután. Ah, se fueron para Usulután entonces. ¿Y cuándo volvieron para acá ustedes?

Fue pasado la…

¿La guerra?

Sí.

¿Ah, ya volvieron ustedes para sus tierras?

Sí.

Y las tierras eran suyas o eran para alguna… Ya les dieron aquí tierras o tuvieron que…

Alquilado.

¿Alquilado?

Sí.

**2. Trayectorias de cuidado ante la enfermedad crónica**

Ajá. Muy bien. Bueno, pues, ahora vamos a hablar un poquito de su enfermedad, ¿vale? Que usted padece del corazón. ¿Cuándo comenzó usted a padecer esta enfermedad?

Mmm, yo tengo varios años…

Ajá…

Y dolor de cabeza que no se me quita. […]

¿Hace cuántos años le comenzó más o menos su enfermedad del corazón?

Hace como unos… como unos tres años.

¿Tres años?

Sí.

Ah, entonces no hace mucho, ¿no? O sea…ya estaba usted…ya estaba bien.

Sí.

Ajá.

Y cuando me duele el corazón, camino un poquito, una cuestecita ahí, y ya me canso.

Ajá.

¿Y es la única enfermedad usted que padece? ¿Padece alguna otra enfermedad?

No, nada...

¿Solamente el corazón?

Sí.

¡Está usted muy fuerte! Eso no es nada.

Sí.

¿Y por qué le comenzó el dolor de cabeza? ¿Sabe usted por qué?

No, no sé por qué, pero me pegó así.

¿De golpe?

Sí.

Ajá. ¿Y qué pasó? ¿Qué estaba usted haciendo cuando le paso eso?

Miré, yo andaba por allá y allí empezó… y allí me vine para acá. Me vine a pie. No miraba nada, oscuro.

¿Oscuro todo?

Sí.

¿No veía nada usted?

No.

Me acosté un rato a una sombra ahí… Dos horas… Me fue pasando de a poquito. Y vine para la casa. Pero estaba siempre…

¿Estaba mal?

Sí, siempre mal.

¿Y usted se vino directamente para acá?

Mmm?

¿Se vino directamente a esta unidad?

Sí.

- ¿Y estaba usted muy lejos cuando tuvo que venir a la unidad de salud?

-Cuando…

-Cuando vino para acá. ¿Tuvo que andar mucho?

-No.

-Ah… Cuánto tiempo más o menos tuvo que andar?

-Poquito…

-Ah, o sea que está bien cerquita.

-Sí.

¿Y entonces usted espero ahí el pick up y lo trajo para acá?

Ajá.

¿Y en el pick up tiene usted que pagar algo?

Sí.

¿Y cuánto paga más o menos?

Mire, una cuota…

¿Una cuota?

Sí. Cuera ida y vuelta.

Ajá. Y aquella vez que estaba usted que se encontraba tan mal, la primera vez, ¿también cogió el pick up para venir acá?

Mmm, no. Me pasó así un poquito. Como a los dos días vine aquí.

Ah. O sea, usted cuando venía se sentó a la sombra un poquito, que pasara como dos horas, se acostó, ¿y ya se fue para la casa?

Sí, me fui para la casa.

Y ahí ya no se encontraba mejor, y a los dos días ya vino para acá.

Sí.

¿Y usted se lo contó a alguien que se encontraba mal?

Sí.

¿A quién se lo contó?

[…] Ya como a los tres pick up me pasaron…

Se vino usted…

Sí.

Ajá. Y se lo comentó a su mujer o a….

Sí.

¿Sí?

Sí.

¿Y lo acompañó su mujer o vino usted solo?

Yo solo […] Tiene mal las canillas.

¿Ah, tiene mal las canillas…las tiene mal?

Sí.

Ajá. ¿Y lo acompañó algún familiar suyo?

Mi hija.

¿Su hija?

Sí.

¿Su hija la acompañó? ¿La primera vez?

Sí.

Muy bien. ¿Y ahí ya qué le hicieron? ¿Cuándo llegó acá entonces usted contó…pasó con el médico general?

Sí.

¿Y entonces le tomaron los signos vitales?

Ajá.

¿Y le dijeron que tenía hipertensión?

Sí.

Ajá. ¿Y entonces qué le dijeron? ¿Le pusieron medicación o …?

Sí, me dejaron… me dejaron en control en el hospital.

¿Ah, se lo llevaron para el hospital?

Sí.

¿En ambulancia?

Sí.

¿Y se fue su hija con usted?

Sí.

¿Y qué hospital fue?

Costera.

¿El de Costera?

Sí.

Ajá. ¿Y allí cuánto tiempo estuvo ingresado?

Estuve como tres días.

¿Tres días?

Sí.

Ajá. ¿Y su hija se quedó allí con usted o iba y venía?

Iba siempre […]

Ajá. ¿Y allí tuvo que pagar algo? ¿Tuvo que pagar algo cuando estuvo allí en el hospital?

No.

¿Nada?

No.

Ajá. ¿Y luego ya le trajeron la ambulancia de vuelta o cómo se volvió?

No, ya cuando venía el pick up…

Ajá. ¿Y cuánto cuesta ir al pick up hasta el hospital?

Setenta y cinco centavos.

¿Setenta y cinco? O sea, tres cuotas.

Sí.

Ajá. Bueno. Y entonces, luego ya después de aquella vez, ¿qué pasó? ¿Ya continuó aquí en seguimiento?

Sí.

**3. Episodios de enfermedad crónica**

Ajá. ¿Y alguna otra vez ha tenido usted alguna otra emergencia también?

Mmm…

¿Que haya tenido que ir al hospital en alguna otra ocasión?

No.

¿No?

No. Sólo eso.

Sólo eso.

¿Y aquí a qué viene usted, aquí a la Unidad de Salud?

¿Aquí?

Sí.

Para que me miren. Para que me hagan examen al corazón.

Ajá. ¿Y qué le hacen de exámenes?

Toman…

¿La tensión?

Eso.

Ajá. ¿Y le ponen las banderitas también?

Ajá.

Ajá. ¿Y viene usted a recoger aquí la medicación?

Sí.

¿Qué se toma usted? ¿Sabe lo que se toma de medicación?

Mire, unas pastillas [menudas]. Una al día.

Ajá. ¿Chiquititas?

Sí.

¿Solamente una?

Sí.

Ajá. Una al día y ya está.

Sí.

¿Y cómo está ahora? ¿Cómo se encuentra usted ahora?

[…]

¿Mejor?

No, mejor no.

¿No se encuentra mejor?

No, no mucho.

¿Se encuentra todavía peor?

Sí.

¿Y usted continuó trabajando o ya no trabaja?

No, ya no trabajo.

Ah, qué bien.

Sólo descansar.

Ya, nada… Ajá. ¿Pero dejó usted de trabajar por aquella enfermedad o…?

Sí.

¿Porque antes sí trabajaba?

Sí, trabajaba.

¿De jornalero?

Sí.

Y ya le dijeron que no pudo continuar trabajando, ¿no?

Ajá.

Ajá.

Que descanse.

Que descanse (risas). Muy bien. ¿Y entonces ahora quién se encarga de la economía familiar?

Mis hijos…

¿Los hijos?

Sí.

Ajá.

Los dos varones.

Los dos varones. ¿Pero no viven con usted, o sí?

No. Están aparte.

Están aparte.

Sí.

¿Pero viven cerquita?

Sí, cerca.

Ah.

Sí, ellos me van a ayudar para la comida.

Ah, muy bien, muy bien.

Sí.

¿A partir de su enfermedad que le están ayudando?

Sí.

Ajá, muy bien.

Sí.

¿Y en casa alguna vez han tenido alguna emergencia? ¿En casa, de alguna cosa de salud que hayan tenido que ir al hospital?

Mmm…no.

¿No?

No.

**4. Calidad de la atención y percepciones de cuidado**

Y aquí en esta Unidad, cuando viene usted, ¿qué, aparte de hacerle los exámenes, ve a alguna otra persona, aparte del médico? ¿A quién ve usted? ¿Ve a la enfermera?

Sí.

¿Ve a la educadora?

Ajá.

¿A la educadora también la ve?

Sí.

¿Sí? ¿Y qué le dice la educadora?

Estoy así…

¿Que está así?

Sí.

¿Y le han dicho de tomar alguna dieta?

No.

¿No?

No me han dicho.

¿Y qué come usted más o menos al día?

¿Ah?

¿Qué es lo que suele usted comer?

Arrocito, ahí… con verduras, ahí…

Ajá. ¿Y los frijolitos?

Y los frijolitos.

(Risas)

Sí…

¿Y alguna tortilla también toma?

Sí.

Ajá. ¿Y cafecito toma también?

Sí, pero ahora poquito.

Poquito, ¿no? Porque por la tensión no puede tomar mucho café.

Sí…

Y eso quién se lo dijo, ¿que no podía tomar café?

[…] Iba mermando, mermando…

Ajá.

Porque a mí me gustaba.

Bien de café, ¿no?

Sí.

Y ahora no, poquito.

Bueno. ¿Y la sal, toma la comida con sal?

Un poquito sí.

¿Un poquito?

Sí…

Ajá, muy bien. Muy bien. ¿Y usted cómo se siente aquí? ¿Por ejemplo, tienen también psicólogas?

¿Eh?

¿Tienen psicólogos también acá?

Sí.

¿Y también le atienden?

Sí.

Ah, ¿sí? ¿Y qué tal? ¿Qué le dice?

Bien, así…

¿Que así…?

Ajá.

Ajá.

¿Y por qué es que se encuentra usted así? ¿Cómo es que se siente?

[…] No me siento bien, bien.

¿Se siente débil?

Sí…débil…

¿Y de ánimos como se encuentra?

No… sólo quiero estar acostadito en la hamaca.

Ajá.

Primero […]

¿Ya nada?

Mmm…

¿Se siente usted triste?

Sí…

¿Por causa de la enfermedad?

Ajá…

¿Está preocupado? ¿Se siente preocupado?

Sí…

Pero si no pasa nada, si está hecho usted un toro, no pasa nada. Está muy fuerte.

(Risas)

Y… Allí cerca de su casa, ¿cuánto tarda más o menos usted en llegar acá? ¿Siempre coge el pick up o tiene allí cerca de su casa algún otro establecimiento?

No… allí espero el pick up.

Allí espera el pick up. Ajá.

Sí…

¿Y cada cuánto viene usted para acá?

¿Ah?

¿Cada cuánto tiempo viene usted para acá?

¿De… tiempo?

Sí.

[…] Cinco minutos.

Ah, muy rápido.

Cerquita.

Está cerquita. ¿Y viene usted cada cuánto tiempo? ¿Una vez al mes…?

Una vez al mes.

Una vez al mes solamente. Ajá… Y allí ya le dan la medicación…

Sí.

¿Y… más o menos, usted cuando llega a qué sitio va? ¿A quién va a ver de acá, dentro de acá? ¿Va usted a ver al médico?

Mmm…

¿Y… a la enfermera?

Sí.

Ajá. Y quién más, ¿qué otras personas ven usted también?

Las que haya.

¿Las que haya acá? A usted le ve todo el mundo (risas). Está bien atendido.

Sí…

¿Y cómo considera que lo atienden aquí? ¿Cree que lo atienden bien o cómo considera usted que lo atienden?

No… son bien buenos…

¿Son buenos?

Sí.

¿Son amables con usted?

Son amables. Todos me conocen.

Claro.

La doctora y la enfermera… Todos me conocen.

Todos le conocen.

Sí.

¿Y tiene usted que esperar mucho tiempo hasta que le atienden?

Mmm… Sí, como hay veces que vengo ya algo tardo.

Ajá.

Los primeros los van pasando.

Ah…

Y tengo que pasar yo.

Y tiene que pasar usted.

Sí…

¿A qué hora suele llegar usted acá?

¿Eh?

¿Más o menos cuándo llega usted aquí? ¿A qué hora más o menos?

Como a las… vine como a las nueve.

A las nueve.

Sí.

Ajá. ¿Y tiene usted cita? ¿A veces?

Sí.

Ah, tiene cita.

A la hora.

Pero eso es cita con el especialista, ¿no?

Ajá.

¿Y qué especialista le ve?

Aquí la señora…

¿La internista?

Sí, la…C.

¿La doctora C.?

Sí…

Ah…muy bien. ¿Pero hoy no va usted a ver a la señora Caña, o sí?

Sí.

Ah, también. Muy bien. ¿Y a la educadora cada cuánto tiempo la ve?

Mmm…. Debe ser como al mes, debe ser…

Ah, muy bien. ¿Y con la psicóloga cuando la ve usted?

Mmmm…es que con ella no…

¿No ha ido a la psicóloga?

No.

Al médico de las emociones.

Sí.

¿A ese no va usted?

No.

¿No? Ajá, bueno. ¿Y… Cuánto tiempo más o menos suele esperar usted hasta que lo atienden acá? ¿Desde que llega a las nueve?

A las nueve, a las diez, a las once, así.

A las once, así. Ajá, muy bien. ¿Y por ejemplo usted se acuerda siempre de tomarse la pastilla por las mañanas, o a veces se le olvida?

Sí, siempre.

¿Se acuerda?

Sí, me acuerdo. Está la esposa… me dice que tome la pastilla.

¿Su esposa le ayuda a recordarle?

Ajá.

Muy bien. ¿Y alguna vez ha tenido algún problema en llegar aquí a la Unidad de Salud? Por tema de transporte, o por tema de que no haya podido pagar el pick up, ¿o alguna vez ha tenido algún problema para llegar hasta acá?

No...

¿No?

No.

Muy bien.

Sí…

**5. Apoyos y ayudas**

Y quién más, en su comunidad, ¿le… Va a algún grupo de apoyo o alguna cosa?

Mmm…no...

¿No? Ah… ¿Tiene amigos dentro de la comunidad?

Sí.

Ajá.

¿Y qué hacen juntos?

Mmm?

¿Qué hacen?

Amistades, ahí...

(Risas)

¿Que se conocen hace mucho tiempo?

Sí.

¿Y qué hacen ustedes?

¿Ajá?

¿Qué hacen para divertirse?

Mmm… nada, solo…

¿Saludar?

Sí.

¿Platican algún poquito?

Sí.

Ah, muy bien. ¿Y va usted a la iglesia por ejemplo?

Sí.

Ah, ¿cuándo va usted?

Los sábados.

¿Los sábados? ¿Y va usted con su mujer?

Sí, y con mi hija.

¿Con su hija?

Sí.

Muy bien. ¿Y a qué otro sitio va usted normalmente dentro de su comunidad?

Sólo a buscar palitos de leña sequita, ahí…

Muy bien, muy bien… ¿Y qué otras cosas más hacen usted para divertirse?

Ir a ver al Milpa, […]

¿A la feria de la Milpa?

Sí…

¿Que eso cuándo eso?

¿Eh?

¿Eso cuándo es? ¿La feria de la Milpa?

En agosto.

¿En agosto? ¡Qué bien! ¡Qué divertido!

Sí…

¿Y allí qué se hace? ¿Qué hay? ¿En la feria?

Mmm… como nosotros llevamos…ya en agosto… ya se hacen tamalitos ahí en la casa.

Claro. ¿Y hay alguna vez que no haya tenido medicación acá? ¿Que no le hayan podido dar medicación?

Sí.

¿Y qué ha hecho usted en esos casos?

He ido al hospital.

¿Ah, ha ido hasta el hospital?

Sí.

¿Ah, y ahí se lo han dado?

Sí.

¿Y alguna vez lo ha tenido que comprar usted?

Mmm… No.

¿No?

No.

Ah, muy bien.

Sí…

Muy bien.

Sí, siempre.

¿Siempre al hospital de Costera?

Mmm…

6**. Percepción de cambios en el tiempo**

Muy bien. ¿Eh… me podría decir usted por ejemplo si ha notado que ha habido algún cambio en la salud, de aquí a diez años atrás? ¿Usted cree que ha habido cambios en el sistema de salud?

Memmi…sí...ha habido.

¿Cuáles, por ejemplo?

Mmm?

Qué cambios ha habido, ¿que usted haya visto?

Y como que antes no tenía… digamos… ahora hay más…

¿Que hay más medicamentos?

Sí.

Ajá. ¿Y están más cerquita los médicos?

Sí, más cerquita.

Y ahí, a su casa, ¿le va a visitar algún promotor de salud?

Sí.

Ah, ¿sí? ¡Qué bien!

Sí, siempre llegan.

Ah… ¿Siempre llegan?

Sí.

¿Y qué le hacen cuando llegan?

¿Eh?

¿Qué hacen cuando llegan?

Que aseemos bien toda la casa… limpiar toda la casa…

¿Vienen a limpiar toda la casa? ¿Para los bichos?

Ajá.

Ajá. ¿Y le toman la tensión, la presión le toman?

Mmm… sí.

¿Sí? Ah, muy bien. ¿Y a su esposa también le toman la presión?

Sí.

Ah, muy bien.

Es que lo mismo… lo mismo que tengo yo lo tiene ella.

¿También tiene hipertensión?

Y más peor, del corazón, del hígado.

Ah…

Y el vaso. Le han dicho que tiene inflamado el hígado.

¿El hígado?

Y el vaso.

¿Y ha tenido alguna urgencia su esposa alguna vez?

Mmm?

¿Ha tenido alguna emergencia? ¿Ha tenido que ir al hospital?

Sí…

¿Sí?

Hace poco la tuvimos una semana en San Miguel.

¿Ah…en San Miguel? ¿Tan lejos?

Sí.

Mmm…

Porque no le hallaron en Costera y se fue para allá.

¿Y se la llevaron para allá? ¿Y cómo se la llevaron para allá? ¿La llevaron en ambulancia?

Sí.

¿Y usted pudo ir a allá a visitarla?

Sí, todos los días.

¿Todos los días? ¿Y cuánto le costaba el transporte?

Allá vale… como… cuatro dólares.

¿Cuatro dólares?

Mmm…

¿Y lo podía usted pagar?

Sí…

¿Podía usted pagarlo? ¿Que lo ayudaban sus hijos a pagarlo?

Sí, me ayudaban.

Ajá…

Ellos me mandaban pues parque fuera.

¿Para que fuera? Ah…

Sí…

Muy bien. ¿Y ahora cómo se encuentra su esposa?

Siempre…[fregado]… un ratito y se entra a la hamaca porque no aguanta.

No aguanta.

Mmm…

¿Y viene ella a recoger aquí su medicación?

Sí.

¿Ella viene también?

Ella viene también.

¿Y la acompaña usted?

Sí.

¿Siempre viene usted con ella?

Siempre venimos juntos.

Muy bien. ¿Y hoy no está ella con usted?

No…

Hoy no. Porque solamente es para usted que viene.

Sí.

Ah…muy bien. ¿Y alguna vez a ella le ha faltado la medicación?

Sí.

¿Sí?

Sí…

¿Y qué han hecho entonces?

Siempre cuando no hay aquí tengo que ir a Cotera.

A Cotera, siempre.

Siempre.

¿Lo ha tenido que comprar alguna vez en la farmacia?

Mmm... sí.

¿Sí?

Sí…

¿Para usted o para ella?

Para ella.

Para ella.

Ajá.

¿Y le ha costado muy caro?

Como veinte.

Uf…

Mmm…

¿Y lo han podido ustedes pagar? Alguna vez…

Siempre… siempre los hijos me mandan…

Siempre los hijos… ¿Pero sus hijos viven todos en El Salvador? ¿O viven fuera?

Sí.

¿Todos viven acá?

Sí.

Ah… ¿Y todos trabajan entonces?

Todos trabajan.

Bueno… Y les ayudan a ustedes.

[………]

Ah, claro. Qué bien. ¿Bueno y entonces qué cosas usted cree que podrían mejorarse acá en esta Unidad? ¿Qué cosas considera que podrían ser mejores?

Mmm… no…

Y, por ejemplo, usted conoce a otras personas, de sus amigos, ¿que también padezcan de la misma enfermedad? ¿O no sabe?

Mmm… sí, hay varios.

Hay varios.

Sí…

Ajá. ¿Y ustedes platican?

Mmm?

¿Platican de ello?

Sí…

Mmm… ¿Y ellos toman también la misma medicación que usted?

También.

Ajá…

Sí…

¿Y hablan con sus amigos de la enfermedad? hace usted algún deporte con ellos? ¿Salen a caminar un poquito… algo?

Sí.

¿Usted camina?

Sí.

¿Cuánto tiempo más o menos camina?

Como unos… Unos diez minutos.

Unos diez minutos, porque ya se cansa, ¿no?

Ajá…

¿Y luego vuelve para casa?

Sí…

¿Y camina usted solo o camina con alguien?

Yo solo.

Usted solo. Muy bien.

[…]

Ajá. Muy bien… ¿Y alguna ha tenido que venir usted andando hasta acá, a la Unidad de Salud?

No.

No. ¿Siempre con el pick up?

Sí.

Muy bien. ¿Y si alguna vez no tiene para pagar le dejan pasar?

Sí.

Ajá.

Siempre me traen, aunque sea…

Aunque sea sin nada.

Sí…

Qué bien.

Sí…

Muy bien. Muy bien. Y, por ejemplo, con sus amigos que tienen la misma enfermedad, se reúnen para hablar de como qué hacer, ¿o alguna cosa?

Sí. Como que le preguntan y usted cómo está… así fregado del corazón…pues nosotros también…

Y ya está. (Risas).

Sí.

[…]

Será a lo mejor ya de la edad…simplemente…

Sí…

Porque yo primero trabajaba de seis a cuatro.

¿Seis a cuatro? ¿De seis de la mañana a cuatro?

Ajá…

[…] Entra a la seis, a las doce ya está fuera…

¿Ah… ahora?

Sí…

¿Ahora solamente de las seis a las doce?

Sí.

¿No dejan a la gente trabajar más?

Nada, no, no trabajar más.

Bueno, porque son muchas horas, ¿no? Hace aquí… Bueno, aquí se está bien fresquito, ¿no?

Ajá…

Aquí no se pasa mucho calor.

Sí…

¿Y usted bebe bastante agua?

Sí.

¿Sí? Muy bien. Muy bien, pues Miguel. No sé si me quiere contar alguna otra cosa de su enfermedad… que sienta, o alguna cosita más que quiera…

Sí, que yo tengo un dolor aquí también.

Ajá.

Me dijeron que en el colón... Y me duele bastante.

¿Y de hace cuánto tiempo tiene ese dolor?

Y hace como unos cuatro años.

¿Cuatro años?

Sí…

Ah… ¿Y ya le han visto qué puede ser?

Mmm… no... Me han llevado a la doctora, pero no me acuerdo…

Ajá… ¿Le hicieron algún examen?

Sí…

¿Y qué le dijeron?

No me dijeron nada.

¿No le dijeron nada?

No.

¿Y dónde le hicieron estos exámenes?

En el hospital.

¿En Otra?

Mmm…

Ajá. ¿Y no le dijeron qué podía ser ni nada?

No…

¿Y no lo ha vuelto usted a consultar otra vez con la doctora XX?

Mmm… no.

Ya ahora hace poco que paso con ella.

¿Ah… antes pasaba con otro médico?

Sí.

Ah… ¿Que ya no está acá?

Ya viene solo por la tardecita, por la noche.

Ah… ya viene solo por la noche.

Sí…

Mmm… ¿Y alguna vez ha tenido que ir el médico a visitarle a su casa?

Mmm…no.

¿No?

No.

Bueno… Y el dolor por qué no se lo consulta a la doctora ahorita cuando pase con ella, ¿que tiene dolor?

Sí, yo le voy a decir.

Claro. Dígaselo, a ver si le puede mandar algún examen.

Si, yo le voy a decir.

¿Porque todos los exámenes siempre los hace en el hospital Gotera?

Sí.

¿O alguna vez le han hecho aquí algún examen?

No, allá…

Allá todas.

Sí…

Ajá. ¿Todos se los hacen allá?

Sí, todos.

¿Y examen de la sangre, por ejemplo?

Ese lo hacen aquí.

Ese lo hacen aquí.

Sí…

Ah, muy bien… Y, por ejemplo, examen de la orina, ¿del pis?

También.

¿También se lo hacen acá?

Sí.

Ajá. ¿Sólo el del corazón, se lo hacen allá?

Sí.

Ajá. Muy bien. Bueno, Miguel, pues ya está. Ya hemos terminado. Muchísimas gracias.

Bueno, por nada.

Muchas gracias.

## Patient interview 12: Man, with Chronic Kideney disease+ Hypertension

Entrevista con: Paciente ERC+HTA

Fecha: 13/06/18
Lugar de la entrevista: Unidad Comunitaria de Salud Familiar Jiquilisco
Código del informante: PMNV003
Genero del informante: M
Nombre del entrevistador: NV

**1. Antecedentes sobre el participante (datos sociodemográficos)**

- Muchas gracias por acceder a la entrevista, lo primero que me gustaría preguntarle es su edad y dónde nació.
- Tengo 66 años, nací en la ciudad de Usulután y allá me crie de niño.
- ¿Cuántas personas había en su hogar cuando eras un/a niño/a? ¿Tiene hermanos y hermanas? Éramos 6 hermanos en total, 2 hembras y 4 varones yo soy el tercero, vivíamos juntos allá en Usulután con mi mamá y papá.
- ¿Pudo ir a la escuela?
- No no, muy pobres éramos.
- ¿Estás casado/a?
- No acompañado, 46 años llevo con ella.
- ¿Tiene hijos/as?
- Tuvimos seis hijos, dos murieron tiernitos.
- ¿Viven sus hijos/as con usted en su hogar?
- Una vive acá en el cantón Nuevo Amanecer y tres en Usulután, ahora nosotros vivimos también en Nuevo Amanecer, desde hace 25 años, aun había guerrilla acá cuando vinimos, pero yo ya era hombre ya.
- ¿Cuántas personas viven actualmente en su hogar?
- Solo mi mujer y yo vivimos, pero mi hija vive muy cerquita, que tiene dos nietos, los otros hijos que viven en Usuluán también tienen hijos, 8 nietos tenemos en total. Pero es que a mi compañera la tengo enferma ahora.
- ¿Cómo es tu hogar, tienen animalitos o algo? Ya solo gallinitas tenemos, antes teníamos más pero ya solo gallinas.
- ¿Y el agua de donde la consumen?
- Del chorro, es la que utilizaos para cocinar, beber y todo.

**2. Trayectorias de cuidado ante la enfermedad crónica**

- ¿Tiene usted una enfermedad crónica? ¿Cuál es?
- Padezco de los riñones y de la presión.
- ¿Cuándo y dónde fue la primera vez que buscó ayuda para los síntomas que presentaba?
- Hace como unos 6 años que yo caí grave y me llevaron a Usulután, me vieron las dos a la vez, que no me la controlaban hasta que vino uno de San Miguel y ya me vieron todo.
- ¿Y cómo fue y dónde que le terminaron diagnostando de su presión alta y los riñones?
- Pues me empezó a venir como un ardor de cuerpo cuando estaba yo ya en casa por la noche que había venido de trabajar y no me llevaron hasta el siguiente día mi sobrino, me llevó a una clínica privada en Usulután.
- ¿Y le dejaron ingresado entonces allá? Si me dejaron allá varios días, pero es que cada dos días había que pagar 100 dólares, era bien caro, pero allá sí que podía entrar la familia y todo y en el hospital público no, solo en horario de visita.
- ¿Y por qué es que decidieron ir a la clínica privada?
- Yo les dije, que según como iba de enfermo, si me llevaban al hospital público me iba a morir.
- ¿Usted con el trabajo tiene algún seguro social o algo?
- No, yo tenía unos animalitos y los vendí todos para pagarlo, pero después de unos días sentí que me tenían ahí para cobrar más y entonces nos fuimos y hasta que no fui al cardiólogo de san miguel no fue que me diagnosticaron la enfermedad. Ellos solo me tenían entretenido y pagarles.
- ¿Qué pasó después? ¿Cómo y dónde fue diagnosticado/a?
- Entonces el cardiólogo me vio y me dio otra medicina, porque en la clínica no me dijeron nada, mi hija me sacó porque me dijo que allá me estaban sacando el pisto y no me iban a curar, ella tenía una amiga que conocía a un cardiólogo del hospital de San Miguel que tenía acá una privada y me cobró 275$ por la consulta pero ya no me iba a quedar ingresado, solo me cobró eso porque vino a verme a Usulután y porque me sacó todos los exámenes y me vieron lo del riñón que me costaron otros 20$ pero ya me lo vieron y lo de la presión también.
- ¿Y allá mismo le dieron medicamentos?
- Sí, yo los compré porque era particular y no te los daban, uno los tiene que comprar en la farmacia y costaban 60$ y no los pude comprar al principio porque ya había vendido todos mis animalitos.
- ¿A quién le contaste acerca de tu enfermedad tras el diagnóstico?
- A mi familia, y a bueno a personas de acá del cantón que me preguntaron que me había salido en los exámenes y yo les conté. De ahí me estuvo viendo el doctor privado dos veces más y cada vez que iba a la consulta me costaba 120$, como él venía de San Miguel en transporte es por eso que me cobraba tanto, me volvía a chequear, me dejaba la receta y ahí ya las compré una vez, y me dijeron que tenía que tomarlas de por vida, el potasio, para los riñones y más cosas.
- ¿Cuándo empezó a acudir a este establecimiento para el tratamiento y seguimiento de su enfermedad?
- Después de que me terminé las medicinas y ya no podía pagarlas fue que empecé en la unidad de salud, pero aquí solo me dan las de la presión. El cardiólogo me dijo que me fuera para la unidad de salud al ver que yo no podía pagarlas, eso fue como al mes de empezar con él. Pero la del riñón no me la dan acá, no hay.
- ¿Y cuando vino a la unidad de salud ya le empezaron a ver acá?
- Si un médico, yo traje el expediente con todo lo que me habían hecho, me ve el médico, pero algunos exámenes me los tengo que hacer fuera y yo le digo que no tengo dinero.
- ¿A usted le está viendo el doctor especialista (nefrólogo)?
- Sí como no, él me ve, pero como le digo no siempre puedo comprar los medicamentos que me manda porque hay que comprarlos por aparte.
- ¿Y cuando viene usted a la unidad de salud, viene en alguna hora especial?
- Ayer mismo vine a hacerme unos exámenes de la sangre y del orín, pero cuando voy a ver al doctor C. ya traigo mi consulta y vengo bien prontito por las mañanas, como a las 8.
- ¿Y tiene que pagar algo en la US?
- No aquí no pagamos nada.
- ¿Qué considera que puede dificultar el manejo de su enfermedad, porque le quede lejos, o no lo pueda pagar...?
- No aquí me queda bien cerquita la unidad y no tengo que pagar nada.

**3. Episodios de enfermedad crónica**

- Desde que te diagnosticaron la enfermedad, ha padecido algún episodio agudo/complicación de la misma?

- Sí a mí me molesta el ácido, me da un dolor así en las canillas.

- ¿Tuvo que ir a algún lugar por ello?

- No me dan los medicamentos para el dolor acá.

- ¿Y alguna vez que se halla puesto más malito de la presión o de sus riñones?

- No, alguna vez he tenido que ir a hacerme exámenes a Jiquilisco porque acá no los había, pero no he tenido que pagar por ellos, era siempre en el hospital de Jiquilisco y ahí no paga uno, solo en el particular y a ese ya sí que no he vuelto.

**5. Calidad de la atención y percepciones de cuidado**

Como nos interesa que la atención en la **unidad de salud** es por eso que le voy a preguntar ahora más sobre cómo se siente atendido, recuerde que nosotras somos investigadoras independientes y todo va a ser anónimo así que dígame como lo percibe usted que es lo que nos importa,

- ¿Se siente **bien recibido cuando llega a la US o no**?
- Sí porque nos dan las medicinas, no andan enojadas, le reciben bien a uno.
- ¿Cuánto **tiempo espera** normalmente para ser atendido cuando viene para que el vea el doctor, la enfermera? Pues la enfermera nos pregunta cómo estamos y todo y luego ya me ve el doctor y me da la medicina en la sala de espera espero poco porque es por orden de llegada. De ahí las saco en la farmacia. ¿Cuánto tiempo suele quedarse en la unidad de salud cada vez que acude? Lo más que nos tardamos es como una hora, un poco más de una hora cuando hay bastantes pacientes, pero si no no me demoro mucho.
- ¿Con cuántos y con qué **personal sanitario** entra en contacto cada vez que acude y por cuánto tiempo?
- La primera que me recibe es la enfermera, que me toma la presión y me pesa, de ahí me da un numero de mi expediente y ya me pasa con el doctor, ya luego con el doctor platicamos con él nos hace las preguntas y de ahí me hace la receta para la farmacia.
- ¿Y cada cuando le ven?
- Pues yo cada mes vengo a recoger los medicamentos, el doctor me ve como cada 3 meses a menos que venga porque esté con calentura o algo.
- ¿Y cuando viene por que tiene calentura o algo, tardan mucho en atenderle?
- De ahí tardo más que cuando vengo con mi cita, pero tampoco se demoran mucho, sobre todo si uno viene malito no tardan mucho en atenderle, siempre depende de cuanta gente halla que por veces hay bastante.
- Además del Dr. C., ¿le ve alguna otra persona de acá de la US, la psicóloga, la nutricionista, la educadora?
- No no, solo el doctor. Nunca me ha visto nadie más que el doctor. Lo único que nos han dicho es que comamos verduras que no tomemos café ni chile…
- ¿Y eso se lo ha dicho algún otro personal sanitario?
- No siempre el doctor me lo dijo, ellos pueden decir, pero poder pagarlo es el problema.
- ¿Considera que le brindan el tiempo necesario de atención?
- Si lo tratan bien, le hacen las preguntas, le digo lo que tengo que decir y lueguito ya me voy para no entretenerle, pero él está todo lo que yo necesite, no es que él me diga que me vaya ni nada. Me da las medicinas y ya luego me voy.
- ¿Considera que le brindan la **información** necesaria sobre su enfermedad?
- Sí si ya sabemos cómo estamos, yo lo que sé es que padezco esta enfermedad y lo que me dijeron es que estaba mal de los riñones y que no podía comer cosas malas, pero uno para cubrir todo eso no alcanza.
- ¿Le apoya la unidad de salud con su **tratamiento**? ¿Cómo?
- Solo me dan la de la presión, la de los huesos, acá traigo el botecito (me lo enseña), pero la de los riñones no me lo dan solo me dieron suero porque las otras todavía tenían unas tabletitas en casa, no he tenido que pagar nada acá, las he pagado afuera.
- ¿Y le dan siempre las medicinas que necesita?
- No, como le digo a veces que no hay y las tiene que comprar una en Usuluán, pero a veces no le alcanza a uno para comprarla entonces en vez las compro a veces no, las de los riñones, las del potasio y ahí es donde hay que comprarlas afuera.
- ¿Y qué le dice el doctor de eso?
- Pues que estoy mal de los riñones, le traje los exámenes de Jiquilisco y me dijo que tengo que controlarme la presión y los riñones, pero ya se sabe que uno de pobre a veces no puede.
- ¿Sugiere alguna medida de mejora con respecto al acceso al tratamiento para su enfermedad?
- Pues que den esos medicamentos que no hay en la unidad de salud y no halla que comprarlos fuera.
- ¿Desde que viene a la US que fue como en 2012 no? ¿Ha tenido alguna situación que le ha dificultado **acceder** a la unidad de salud? ¿Cómo lo ha resuelto?
- Yo siempre he podido venir, en bicicleta me vengo. Cuando vengo con mi mujer ella no puede y me la traigo en mototaxi por un dólar. Mi señora a veces la trae un sobrino en carro porque ella está bien malita, el derrame la dejó bien sequita, se trancó y no comía, le pasó como a las 10 de la noche y allí me la llevé a un doctor privado y de ahí me la pidieron un Tac que me costó 120$, eso fue como hace 8 meses, fíjese que ahora no puede ni hablar.
- ¿Y a ella la ven acá en la US?
- Si cuando se encuentra mal se viene acá y le dan las medicinas y eso, pero el privado la sigue viendo con un Tac cada 6 meses por si sangra o algo, pero gracias a Dios no sangró la consulta nos cuesta 20$ y la prueba 120$ en septiembre va a ir otra vez al examen, pero no habla a penitas dice alguna palabra.

**6. Apoyos y ayudas**

- ¿Se siente **apoyado** por el personal sanitario en el seguimiento de su enfermedad?
- Sí porque me dan las medicinas.
- Pero no solo con las medicinas, a nivel emocional me refiero, le apoyan con lo de su mujer por ejemplo que pueda usted platicar con alguien (una psicóloga, por ejemplo).
- Sí si le apoyan a uno, pero yo acá no he contado nada, lo que sabe el doctor es la enfermedad mía no lo de mi mujer.
- ¿Hay algo que cambiaría para mejorarlo?
- No bien, no nos vienen viendo mal, intentamos hacer las recomendaciones que nos han dicho, en veces cuando nos dicen que hay que comprar algo afuera es lo que no podemos porque somos pobres y no podemos comprar una medicina cara a fuera.
- ¿Quién le apoya en el manejo de su enfermedad y cómo?
- Mis hijos están en Usulután, pero yo solo yo con ella paso. Mi hija la que vive acá lo que hace es que la muele las tortillitas y eso porque no le dejamos que esté en la cocina porque nos han dicho que el humo la sienta mal así que a veces me toca a mí prender el fuego para que no esté ella fogueándose.
- ¿Participa en algún tipo de organización en su comunidad? ¿Qué tipo de apoyo le brinda?
- No en nada estoy participando, yo trabajo todo el día en el campo, nunca he participado en la Adeco ni nada, fíjese que yo ni a misa voy porque me queda muy retirado.
- ¿Sabe si hay algún grupo de apoyo específico para su enfermedad crónica?
- Sí hay gente bastante que viene a buscar medicinas para lo mismo, yo digo que está bien que haya una reunión para poder hacer preguntas apoyar, pero yo cuando las han hecho nunca he podido venir por lo de mi mujer.
- ¿Y me decía que trabajaba en el campo, siempre ha trabajado en eso?
- Sí desde los 12 años es que trabajo allá, tengo una tierrita, pero lo que cultivo es para la casa. Antes de más chiquito trabajaba en una hacienda en las algodoneras y bombeaba, pasaba el avión y ese nos bañaba, era veneno que n os caía, nosotros nos agachábamos, pero siempre nos caía.
- ¿Y cuánto tiempo trabajo en las algodoneras?
- Pues más de media vida, con eso crie a mis hijos cobrábamos me acuerdo 124,50 colones cada 15 días y todo y luego ya pasé a cultivar mi tierra cuando nos dieron el terreno allá por los 80, en la guerra vinimos a agarrarlo, pero no podíamos cultivarlo porque no nos dejaban, pero después ya sí que tras los acuerdos de paz nos lo dieron.
- ¿Recibe algún tipo de ayuda para pagar todo lo de las consultas privadas como lo hicieron? ¿recibe algún tipo de subsidios?
- Pues fíjese que vendí todos mis animalitos para pagarlo. De ahí nos dan subsidio para el gas desde que como comenzaron a darlo hace unos cuatro años, nos dan una tarjeta y con eso pagamos el bolado del gas.

**7. Percepción de cambios en el tiempo**

- ¿Podría describir si ha notado algún cambio en los últimos 10 años en cuanto al manejo de su enfermedad?

- El cambio es que ahora nos queda cerca y no tenemos que pagar las medicinas, ese es el mayor cambio. Antes no estaba la unidad de salud acá, no recuerdo cuando empezó.

· ¿De qué manera las cosas mejoraron o empeoraron?

- Por lo menos está mejor porque nos dan las medicinas que acá lo tenemos cerca.

· ¿Por qué cree que ha podido haber estos cambios? ·

- Esto salió como a modo de proyecto y dijeron que iban a hacer la unidad de salud, supuestamente fue la directiva de la comunidad la que hizo esas gestiones, la adeca.

· ¿Han cambiado los costos de los medicamentos? ¿Cómo?

- Sí ha cambiado, acá no se paga nada de las medicinas ni nada, nos las regalan.

· ¿Tiene alguna sugerencia para poder mejorar el seguimiento de su enfermedad crónica desde el primer nivel de atención?

- Bueno yo creo que el objetivo es que al menos se quede como está.

¿Tiene alguna pregunta para nosotras? ¿Mire y esto para que es, esta encuesta, esto para qué es? Pues estamos colaborando desde una universidad de Inglaterra con el ministerio de salud y con el FNS y con todo lo que ustedes nos digan haremos propuestas para que mejore la unidad de salud.

Patient interview 13: Woman with Hypertension

Entrevista con: Paciente con HTA

Fecha: 14/06/18

Lugar de la entrevista: Cantón La Palma

Código del informante: PFNV004
Genero del informante: F

Nombre del entrevistador: NV

NV: Muchas gracias por acceder a la entrevista, lo primero que me gustaría preguntarle es sobre usted, su edad y dónde nació.

P: Tengo 61 años, nací acá en la Palma.

NV: ¿Cuántas personas había en su hogar cuando eras un/a niño/a? ¿Tiene hermanos y hermanas?

P: Éramos 4 hermanos en total, 2 hembras y 2 varones .

NV: ¿Pudo ir a la escuela?

P: No, nunca pude acudir a la aescuala, eramos muy pobres y desde chiquita tuve que ayuda r en el hogar.

NV: ¿Estás casada?

P: Sí, llevo mucho años casada con mi mardio, bien jovencita me casé.

NV: ¿Tiene hijos/as?

P: Sí, tengo 3 hijos, uno de ellos vive en EEUU y otros dos acá cerquita.

NV: ¿Cómo es tu hogar, tienen animalitos o algo?

P: Ya solo gallinitas tenemos, antes teníamos más pero ya solo gallinas.

NV: ¿Y el agua de donde la consumen?

P: Del chorro, es la que utilizaos para cocinar, beber y todo.

NV: Ahora me guataría pasar a pregutnarle sobre su enfermedad, ¿Tiene usted una enfermedad crónica? ¿Cuál es?

P: Padezco de la presión y de los riñones.

NV: ¿Cuándo y dónde fue la primera vez que buscó ayuda para los síntomas que presentaba?

P: Hace como unos 4 años que yo caí grave y me llevaronl hopaital de La palma,

NV: ¿Y cómo fue y dónde que le terminaron diagnostando de su presión alta y los riñones?

P: Pues después del ingreso en el hospital me dijeron que debía hacer seguimetno en la Unidad de salud de La Plama y es allí donde finalmetne me confirmaron que tenía lapresion alta.

NV: ¿Y desde entonces le siguen acá en al Unidad de salud?

P: Sí la me siguen acá en unidad, aunque a veces es difícil encontrar los medicamentos..

NV: Pero no rmalemtne le dispensan acá los medicamentos para su presión?

P: Como le decía, bien difícil encontrarla en la unidad de salud. Ya me tocaría comprarla. Porque estoy tomando de dos. Una para no juntar agua. Me dijeron que tenía que tomar las dos.

NV: ¿Dos por día? ¿A qué hora se los toma?

P: Si, a mí me dijeron a las 5 de la tarde. Con comida.

NV: ¿Alguna vez ha pasado que vas a la unidad y no hay medicamento?

P: Gracias a dios hasta al momento no, porque me dijeron que fuera los 3 días antes. Y siempre… cuando estuve internada me dieron para un mes. Y como yo siempre tengo de reserva.

NV: Ojalá que no pase, pero en donde comprarías si tendrías que hacerlo?

P: Las farmacias quedan en la palma.

NV: ¿Y cuánto cuesta?

P: La del corazón, me costó 17 dólares cuando lo compre por el mes. Hay bus que cuesta 90 centavos para ir. Si vas hasta La Palma vale otro 90 para ir y para llegar. Es como 45 minutes para llegar.

NV: ¿Desde que le diagnosticaron la enfermedad, usted a aparecido algún episodio o complicación?

P: No, casi no.

NV: ¿Cuándo fue que le diagnosticaron?

P: Fue en agosto del año pasado. No fue en marzo, porque ya hizo año.

NV: ¿Y ya se controló?

P: Si, o sea al tener preocupaciones, como siento que la presión del pecho siempre… a veces el brazo…

NV: Ahora le voy a preguntar sobre la calidad de atención de salud que usted ha recibido. ¿Me podría decir un poco sobre su experiencia?

P: En algunas personas, fue buena. Pero con otros, algo como aburrido, como que están tomado a cargo, pero…no me refiero aquí pero allá en el hospital hay de todo. Veo uno que les da malos tratos a los pacientes. A mi gracias a dios, un poquito aburriditos por ratito, pero uno como de necesidad tiene que tener paciencia.

NV: ¿Me puede dar un ejemplo?

P: Yo allí a donde estaba avía una muchacha que le daban dolores de parto y la trataban como que…bien raro. Porque ella se quejaba, le maltrataba una señorita como a la media noche. Ella quizás se había quedado sola de turno y quizás ya estaba aburrida. Ahora cuando estas con los grandes dolores…yo siento que es un maltrato porque en esos momentos necesitan que alguien le hable. Para me era duro. Ella estaba solita. Bien jovencita como de 17 años y asustada.

NV: ¿Alguien se acercó a ella para dar le algún apoyo?

P: Todos estábamos bien mal. Nadie le podíamos dar apoyo. A mí ya me avían operado, estaba allí que recién que me han llevado. Mis otras compañeras con bebitos que también. De paso hacíamos comentario que a veces no se merece que le traten de esa manera. Porque a veces la exigencia que uno no se queda en casa, no permiten la partera en las casas. Aquí hay insistencia que vayan al hospital.

NV: ¿Pero usted se sintió bien recibida?

P: Si, por mí no hubo ningún problema.

NV: ¿Y como son los controles?

P: Una vez por mes. Siempre casi voy a la misma hora. En la tarde.

NV: ¿Cuantas personas trabajan en la unidad?

P: A veces esta la enfermera, y la licenciada. Pero a veces como ellas entran así siempre con reuniones, ella siempre esta.

NV: O sea usted visita una vez por mes con la enfermera. ¿Cuanto tiempo es la visita?

P: Si, a veces voy con mis hijas cuando están enfermas. Nunca he medido el tiempo, pero menos de media hora. Uno tiene que ser consciente, si hay pacientes, tiene que esperar un ratito.

NV: ¿A las otras unidades, no tiene necesidad de ir?

P: Cuando no hay aquí hemos ido a La Palma. Pero allí es bien duro. Un día llegamos con mi esposo porque él iba bien mal, y llegamos a las 9:30 de la mañana y nos venimos como a las 3:30pm sin atención, porque no nos atendieron en La Palma.

NV: ¿Porque fue que no atendían? ¿Por mucha gente u otra razón?

P: No había tanta. Porque ya hemos ido cuando esta así y nos atienden. Pero ese día, no sé por qué estaba tan lenta la consulta. Dijeron que solo había un médico, pero yo vi como tres que estaban dando consulta de primero. Pero no sé qué paso.

NV: ¿Que hicieron entonces?

P: Nos venimos sin pasar la consulta. Nos regresamos para la casa, sin medicinas, sin nada porque no nos atendieron.

NV: ¿Regresaron otro día?

P: No, mi esposo compro medicina para calmar el dolor.

NV: ¿Eso ha sido en La Palma?

P: Si en La Palma. Ese día no compramos nada por que dijimos vamos a regresar a la unidad de salud. Pero no, así compro la medicina para el dolor.

NV: ¿Considera usted que le dan el tiempo necesario en las citas médicas?

P: Si. Bueno depende del tiempo que tiene el médico. Porque a veces tiene muchos pacientes. Pero si, cada paciente lleva su tiempo. Porque se tiene que escribir.

NV: ¿Cuanto tiempo les dan el medico?

P: Unos 15 minutos. Me pregunta si me he sentido mal. Siempre me pregunta cómo voy, de la gastritis, del colon.

NV: ¿Y le da usted consejos?

P: Si, sobre la comida, que tengo que tener cuidado con la comida porque es parte de la medicina. Porque si uno no tiene cuidado con la comida entonces hay problemas.

NV: ¿Que tipo de comidas le conseja?

P: Comidas suaves, verduras, cosas verdes

NV: ¿Le hace falta otros tipos de comida?

P: Si a veces uno no la loga consumir la porque económicamente no tiene para comprar, como la fruta. Si hay algunas frutas, pero no de todas. Y en algunas yo tengo problema por lo cítrico.

NV: ¿Sobre la información, usted siente que le brindan la información suficiente sobre su enfermedad y cómo manejarla?

P: Si, siempre con los cuidados especiales, de no comer sal, no tomar café, soda…consejos.

NV: ¿Usted siente que la apoya la unidad de salud con su medicamento?

P: Si

NV: ¿Sugiere alguna medida al respeto del acceso al tratamiento?

P: Por el momento yo he sentido que no he necesitado ningún otro tratamiento por qué no he empeorado.

NV: ¿Usted ha tenido alguna situación que le ha dificultado acceder a la unidad de salud?

P: A esta unidad no, pero cuando vamos al hospital sí. Porque ya ha habido veces que hay paro, y que no estado corriendo los buses. Este, le cuesta uno para llegar pues. También por las calles que está tapada, llueve mucho con derrumbe y no se puede pasar.

NV: En esos casos, ¿que hace?

P: Sacar nueva cita. Por ver allí, sacarme la cita, y allí el proceso es más largo, 4, 5 meses o 3 meses. Por eso se lucha por siempre estar pendiente.

NV: ¿Porque se demora tanto?

P: A mí me han dicho porque está llena el libro. No hay espacio. Solo dejan un espacio para las emergencias.

NV: ¿Que pasa si se pierde la cita?

P: Para ver si le solucionan un poco la medicina para mientras.

NV: ¿Como consideras que se tratan los otros pacientes en la unidad de salud?

P: Aquí las veces que he ido, normal. Yo preferencias no he visto.

NV: ¿Se siente usted apoyado por el personal sanitario en el seguimiento de la enfermedad?

P: Si. Si porque si voy allá me preguntan cómo voy. Y siempre me dicen, no dejen de tomar su medicamento. Si usted lo deja es problema, porque en tres días de no tomar, empiezas sentir mal.

NV: ¿Usted tiene algún otro apoyo?

P: No de otra gente no, pero mis hijas, mi esposo, mis yernos, el apoyo de familia.

NV: ¿Ellos te apoyan en el manejo?

P: Mi esposo, mis hijas. ¿Me dicen ‘¿Allí mami, tienes la alarma para la medicina?’ Mi esposo también.

NV: ¿Tu esposo también los toma?

P: No.

NV: ¿Se les olvida de vez en cuando?

P: Si, ellas tienen allí la alarma para que me las tome a la misma hora. Solo cuando salgo, a veces o tengo algún apuro, me la tomo a distinta hora.

NV: ¿Que apoyos económicos existen en el hogar?

P: Solamente mi esposo. Solamente a veces se prestan el dinero, entre familia.

NV: ¿También hay apoyo de la comunidad?

P: O sea yo siempre he sido así tímida de no querer molestar a alguien. Gracias a dios no, porque yo sé si molesto… prefiero mejor entre familia.

NV: ¿Usted participa en alguna organización comunitaria?

P: No ahorita. Estuve en la directiva del dispensario de la unidad de salud, pero ya no. Allí estuve en la directiva viendo en que se apoyaba porque allí avían muchas necesidades.

NV: ¿Cual era su función allí?

P: Este como de las ultimas, como de la directiva, pero la cambiaron. Intentaron hacer ventas cuando se necesitaba, para pagar la luz…no tiene el apoyo…o sea no le paga la luz el gobierno, y si no le pagan se quedan sin luz.

NV: ¿Eso pasa frecuentemente?

P: Si, hoy no le preguntado como están, pero si el año pasado, allí tenían que embolsarse entre ellos para no se les corten la luz. Hasina vents, tacos, pastels, purposes.

- Interrupción –

NV: ¿Usted ha notado algún cambio en los últimos 10 años en el contexto de visitar a las unidades de salud?

P: Si, por el medicamento. No lo digo por lo del corazón. Pero si voy por la gastritis, no hay, si voy por el corazón no hay. Ya eso le perjudica alguno. Porque uno lo necesita y de allí hay que comprar.

NV: ¿Esto lo ha notado en los últimos 10 años?

P: Si en estos últimos años. Lo que ha mejorado es que hoy tenemos los médicos más cerca. No antes no, antes nos tocado ir a La Palma.

NV: ¿Este que está aquí a lado, cuanto tiempo está allí?

P: Yo no recuerdo, 7 o 8 años.

NV: ¿Por que han decidido ponerlo allí?

P: Porque muy lejos, y mucha gente, creo que no le daban cumplimiento atenderla todo allí.

NV: ¿Algún otro cambio que se le ocurre?

P: [No response}

NV: ¿Estos cambios del medicamento, porque cree usted que esto ha pasado?

P: Es la pobreza quizás no van [¿], porque más años más enfermos quizás porque la población ya no vivimos alentados. Talvez antes la gente se enfermaba menos. A veces el médico me dice lo siento, pero no hay medicina, solo la receta, ‘tu si la compras’.

NV: ¿Como han cambiado los costos de los medicamentos?

P: Si yo digo que si hay cambios. Pues si yo siento que hoy ya el dinero no alcanza. Para los hombres no crea usted que hay un gran trabajo que uno va a ganar dinero. Uno va peor. Le ayudamos trabajar allí en campo cuando hay necesidad, pero dinero no sé. A veces si sembramos, no hay cosecha, porque viene el huracán, y en el tiempo se pierde.

NV: ¿Y algún costo de atención medica se ha cambiado?

P: No siempre ha sido así, sin pagar.

NV: Algunos de estos cambios, o sea de los costos, ¿han cambiado su uso de la atención medica?

P: Si, a veces tengo que dejar algunos tratamientos porque no alcanzo comprarlos. Por ejemplo, tuve la bacteria también…el nombre no sé, pero la tuve. Me dejaron el examen a los tres meses para que siguieran viendo si tengo. Pero no me lo he hecho porque esa cuesta como 28 30 dólares el examen.

NV: ¿El examen no es gratis?

P: No, porque hay que sérselo particular uno por que lo hacen en laboratorio. Aquí no llevan todo los examines. Si a uno le, tiene emergencia, tiene que hacérselo en el particular.

NV: ¿Usted tiene alguna sugerencia sobre de mejorar la atención de salud?

P: Pues sí, lo importante y lo bonito fuero que si tendrían el medicamento. Si de verdad le preocuparía si eso no había. Uno se preocupa mucho si no hay. Pues sí, transporte sería necesario. Porque hasta aquí no llega ambulancia. Bueno a veces los jueves traen carros para traer a las embarazadas. Hicieron una reunión como para los dueños de carros si ellos estaban de acuerdo a colaborar. En una emergencia, la verdad que la gente no tiene facilidades llevarlos a la unidad más cercana.

-Interruption-

End of Interview

## Patient interview 14: Woman with HTA

Entrevista con: Paciente

Fecha: 15/06/18

Lugar de la entrevista: Unidad de Salud La Palma

Código del informante: PFNV005
Genero del informante: F

Nombre del entrevistador: NV

Sección 1: Perfil del participante y datos demográficos

NV: ¿Me podría decir su edad por favor?

I: Mi edad, tengo 64 años.

NV: ¿De donde viene usted?

I: Nací en San Ignacio.

NV: ¿Usted vive en San Ignacio?

I: No, yo vivo aquí.

NV: ¿Cuándo se mudó aquí de San Ignacio?

I: Cuando estaba (¿tierna?) [joven], nos mudamos aquí.

NV: ¿Con quien vive ahorita?

I: Vivo con mi mama y mis hijos. En mi hogar viven 9.

NV: ¿Quien son los que viven allí?

I: Mis hijos, o sea en mi hogar solo son… mis hijos son 7, ya de allí son nietos los que tengo…

NV: ¿Hay algunos que viven en la casa?

I: Si, viven 4.

NV: ¿Tiene hermanos que también viven aquí?

I: Tengo un hermano en La Palma. Antes vivía en El Refugio. Yo vivo en El Refugio, y mi hermano vive aquí en La Palma.

NV: ¿Usted asistió a la escuela?

I: Si, aquí en La Palma.

NV: ¿Hasta que año asistió la escuela?

I: 6to grado

NV: ¿Usted está casada o a estado casada?

I: No

NV: ¿Me podrías contar de sus hijos?

I: De mujeres son 4 y de barrones 3

NV: ¿Y sus nietos?

I: Todos barrones

NV: ¿Como se hace dinero en su casa?

I: Mis hijas trabajan

NV: ¿En que trabajan?

I: En comedor. Y mis hijos trabajan en tierra

NV: ¿Usted antes trabajó?

I: Yo trabajé antes, así como mis hijas, en comedor

NV: ¿Y está cerca adonde trabajan?

I: Aquí en La Palma

Sección 2: Trayectoria de la enfermedad

NV: ¿Me podría explicar un poco sobre la enfermedad que usted tiene?

I: En realidad solamente de la presión. Y la última semana que tenía un poquito alta es de los triglicéridos…pero de colesterol no. Y diabetes.

NV: ¿Me puede contar sobre la primera vez que usted descubrió esto, o sea como fue la diagnosis?

I: Hace unos 6 años. Yo llegué a la clínica comunitaria en Cítala, me dijeron que tenía la presión alta y sobrepeso y me mandaron para acá [La Palma].

NV: ¿Como era esa clínica?

I: Es así como…que hay así son las unidades.

NV: Eso fue en…

I: En El Gramal

NV: ¿Después que fuiste a la unidad que pasó?

I: Me mandó porque tenía sobrepeso y entonces el doctor ya me puso el control de la presión

NV: Te tomaron la presión allí mismo. ¿Le dieron algún medicamento?

I: No, ya me dio el medico

NV: ¿De allí en la comunidad le mandaron aquí a La Palma?

I: Si

NV: ¿Como fue cuando usted llego aquí a esta clínica? ¿Me podría contar como fue esa primera visita?

I: Es que como ya me dio el doctor allá, ya me dio un papelito como una referencia. Entonces me atendieron aquí.

NV: Entonces usted llego a la ventanilla aquí y le mandaron a ver el doctor. ¿Cuanto tiempo demoró la cita con el doctor?

I: Yo llegué día miércoles a la clínica comunitaria como le digo. Y después de un día me vine aquí.

NV: Cuando usted llego aquí, cuanto tiempo paso durante toda la visita

I: No recuerdo mucho

NV: Esta bien. ¿Ellos le dieron el medicamento o tuvo que ser con receta?

I: Si medicamento

NV: ¿Por cuanto tiempo le dieron?

I: Primeramente, un mes. Y de allí cada 3 meses. Solo vengo a retirar medicina

NV: Y le hacen los controles cuando vienes

I: También

NV: ¿Eso es con cita o vienes nomas?

I: Con cita.

NV: ¿Como se saca la cita?

I: Aquí se la dan en la consulta

NV: ¿Ha habido una vez que no pudiste estar en la cita?

I: No, no he faltado a la cita

NV: ¿Cuándo le diagnosticaron, le conto a alguien?

I: Solo la familia

NV: Sus hijos

I: Si

NV: Siempre haces las consultas en esta unidad. ¿Ya no necesitas ir a la de la comunidad?

I: No ya no

NV: ¿Cuándo llego aquí a la unidad, tuvo que esperar?

I: Había que esperar, como llega bastante gente. Cuando uno llega así lo van atendiendo

NV: ¿Generalmente a qué hora viene usted aquí?

I: Depende. Como hay unos que le pueden atender en la mañana, otros de medio. Si no desde las 12…

NV: ¿Usted tiene preferencia?

I: Han

NV: ¿Y como llega usted a esta unidad?

I: En bus.

NV: ¿Cuanto tiempo demora en bus?

I: 10 minutos

NV: ¿Es fácil tomarlo, vienen frecuentemente?

I: Si, cada media hora

NV: ¿Desde su casa para llegar aquí, cuanto tiempo demora para llegar aquí?

I: Depende, porque a veces uno sale temprano de la casa y alguno…acaba de pasar uno y tiene que esperar el otro. Una media hora hay que estar allí esperando

NV: ¿Hay algo sobre su enfermedad que usted ha encontrado difícil en el manejo?

I: Todo bien. La medicina la voy manejando bien.

NV: ¿Es medicina que se toma con horario?

I: Si, en la mañana en ayunas. Una pastilla todo el día.

NV: ¿Hay veces que se le han olvidado? ¿Como hace para poder acordarse?

I: A veces me olvido. Como voy a la iglesia como allí hacemos desayuno entonces allí si no los tomo. Me las tomo hasta que venga de regreso

NV: ¿Hay algún otro ejemplo de algo que lo encuentra difícil sobre el manejo?

I: No

NV: ¿Desde que le diagnosticaron, usted ha padecido algún episodio o complicación de la enfermedad?

I: No, bien controlado

NV: ¿Cuándo usted entro la primera vez con la presión, allí nomas todo se controló con el medicamento?

I: Si

NV: ¿Y cuando le diagnosticaron por primera vez, como se sintió emocionalmente sobre el diagnostico?

I: No…yo solo vine y me dio la medicina y yo no me sentía ni muy mal ni bien. Me sentí tranquila

NV: ¿No se sorprendió?

I: No, como yo no soy tan de… a mis nervios se me sentían bien

NV: ¿Entonces eso fue hace 6 años, en esos 6 años todo controlado?

I: Todo controlado, si

NV: ¿Ha tenido alguna otra enfermedad en esos 6 años?

I: Lo más ha sido mi rodilla porque eso es lo que me molesta a mí.

NV: ¿Que tiene, siente dolor?

I: Es que tengo como artritis, o no sé qué tengo en la rodilla. Caminando así rápido no puedo. Este hay días que me amanece hinchada la rodilla. Es que a mí me dio una enfermedad que se llama ciática. Pero a mi dio como ya hace 38 años que estoy padeciendo. Y esta rodilla me la inyectaban.

NV: ¿Alguna otra complicación en los últimos años?

I: No.

NV: ¿Las citas que haces siempre son para recoger los medicamentos o también por otras razones?

I: A veces una que otra ves que como que me quiere dar infección a los riñones. Que me agarra el dolor aquí así. Entonces vengo así, pero a veces de bajo salud o consulta general.

NV: ¿La consulta general lo hace aquí?

I: Aquí mismo

NV: ¿Y que le han dicho sobre dolor de riñón, le dan medicamento?

I: A veces me dan para infección…como de…como te iba decir, examen de orina. Y a veces cuando me sale con infección, me da medicina para la infección. Antibiótico.

NV: ¿El antibiótico se lo entregan aquí?

I: Si, si

**Sección 3: Calidad de atención**

NV: ¿Cuándo usted acude a la unidad de salud, se siente bien recibida?

I: Si, si

NV: ¿Me puede dar un ejemplo de donde usted sintió algo superior, o al contrario?

I: No, todo el tiempo a mí me han atendido igual. Ni más arriba, ni más abajo.

NV: ¿Usted a notado que es igual para todos?

I: Si, sí.

NV: ¿Se acuerda cuanto tiempo se queda en a la unidad de salud generalmente?

I: Yo vengo a las 9, 10:30 ya voy de regreso. Hora y medio

NV: ¿Y en ese tiempo usted tiene algo que hacer o quizás siente que necesita el tiempo?

I: Es que uno tiene que sacar el tiempo para venir. Porque si no saca tiempo para la medicina de uno, entonces…si, se tiene que sacar el tiempo.

NV: ¿Y que haría si no estuviera con la cita del médico?

I: Pues si, tal vez trabajando.

NV: ¿Cuándo usted llega aquí con cuanto personal sanitario se encuentra?

I: Bueno, solamente con la enfermera y el doctor. Porque ella reciba tarjeta, y de allí ya está el cuadro preparado y ya de allí para sacar [¿].

NV: ¿Siente que el tiempo con el doctor es suficiente?

I: Si

NV: ¿Nunca siente que quisiera más tiempo para averiguar información, o más tiempo en general con el doctor?

I: No, como uno va allí, ya sabe, no le pregunto

NV: Y usted cuando le han diagnosticado, le dio alguna información sobre la enfermedad, ¿o como le explicó?

I: Me explicó que la medicina la tenía que tomar todo el día conforme la receta que le da a uno, la hora de, cuando tiene que tomarla…

NV: ¿Sobre las enfermedades, le dio alguna explicación sobre las causas o síntomas?

I: Si

NV: ¿Que le explico?

I: Ya no me acuerdo

NV: Esta bien. ¿Usted siente que usted tiene entendimiento de todos los síntomas, causas…de donde se consigue esa información? ¿Eso lo tuvo antes o según el diagnóstico?

I: Según el diagnostico

NV: ¿Y antes de la enfermedad, usted tenía esta información?

I: Yo no sabía. Yo no sabía que tenía todo eso.

NV: ¿Pero en general, quizás conoces otra persona con la misma enfermedad?

I: No en la familia no. No. En mi hogar, solo mi mamá

NV: ¿Y ella le contaba sobre su experiencia?

I: No…como ya también…no…no me acuerdo de cuánto tiempo se sabía identificar…

NV: Ya, está bien. Bueno, sobre este grupo de apoyo, ¿como es que se enteró de este grupo?

I: El doctor me dio la orden.

NV: ¿Fue un orden?

I: Pues sí, él me dijo que era una estrategia este para una…para salir mejor

NV: ¿Cuándo fue eso?

I: viernes. Ya hace pocito.

NV: O sea usted vino a la consulta

I: Cita, tenía cita

NV: Y le dijo sobre el grupo…

I: Que forma la parte de este grupo

NV: Y como decidió de ir allí

I: Yo dije, “voy a ir allí para ver de qué se trata” Es que quiero ir a ver de qué se trata

NV: ¿Y le dio alguna otra información sobre el grupo o solo la orden?

I: La orden, y me dijo con la enfermera ya me dio la cita para hoy

NV: ¿Y la unidad de salud la apoya con su tratamiento?

I: Si

NV: ¿Sugiere alguna medida de mejora con respeto al tratamiento?

I: Yo creo que todo está bien. Si.

NV: Usted ha tenido alguna situación que le ha dificultado acceder la unidad de salud. ¿Por ejemplo, con el transporte u otra situación?

I: La cosa es como a veces uno…es como está la situación…se preocupa por venirse, aunque sea en carro

NV: ¿Se preocupa por venirse en carro?

I: Si por estar a tiempo a la cita. Cuando a veces no hay autobús y veo que ya es tarde, yo veo como hago para venir.

NV: ¿Conoces a alguien que te puede llevar?

I: Si, mi sobrino tiene carro. Si necesito lo llamo

NV: ¿Alguna otra situación de dificultad?

I: No

[Interrupción]

NV: ¿Usted ha visto como están tratados los otros pacientes?

I: ¿Como…que le tratan bien?

NV: ¿O sea un ejemplo si usted ha visto que le tratan muy bien o al contrario?

I: Todo bien, todo bien.

NV: ¿Y para usted?

I: Igual

NV: Para seguir con preguntas de apoyo, bueno el doctor le dio esta información, ¿no? ¿El doctor le dio alguna otra información sobre grupos de apoyo?

I: No porque…solo este.

NV: Y antes, en los últimos 6 años, ¿le dio algún otro dato sobre grupos de apoyo?

I: No, no habido necesidad quizás. Yo manejo bien la enfermedad.

NV: ¿Tiene alguna otra persona que le apoya, quizás en la familia?

I: En la familia…si como todos ellos ya saben que yo tomo la medicina, allí está el apoyo hasta que me la tome. Si no se me olvida a mí pues.

NV: ¿Y en su comunidad hay algún tipo de grupos en que usted participa?

I: No. En realidad, yo tengo tiempo de no ir a la comunidad. Solo aquí.

NV: ¿Ah ya, y aquí hay grupos?

I: Como le digo, no sé.

NV: ¿Seria algo que usted tendría interés de participar?

I: Yo pienso que como nos vamos reuniendo allí vamos a saber.

NV: Oka está bien. Ahora quisiera preguntar un poco sobre su experiencia visitando las unidades de salud, pero por otras razones no relacionados con la enfermedad de ahora. ¿Usted ha visitado la unidad de salud por otras razones?

I: No…Si, por gripe sí.

[Interrupción]

NV: En esas ocasiones de visitar la unidad de salud, o sea por gripe u otra razón, que tipos de cambio ha notado en los servicios de salud que ofrecen, o sea en calidad, acceso…

I: Igual, igual, todo el tiempo ha sido igual.

NV: ¿Y quizás en los últimos 10 años, tendrá un ejemplo en algo que ha mejorado?

I: Si, está la casa materna.

NV: ¿Ah sí? ¿Es nuevo?

I: No sé desde cuando

NV: Sera una gran necesidad. ¿Que función tiene la casa?

I: Porque aquí las tienen a ellas esperando el bebé, y de allí los llevan al hospital

NV: ¿Y no había enantes? ¿Como hacían antes?

I: En la casa estaban, y ahora no porque están más cerca.

NV: ¿Mas peligroso en la casa?

I: Si. Mis hijos nacieron en la casa.

NV: ¿Si pasara en esta época, usted prefería estar en la casa materna o en casa?

I: No pudiera decir

NV: ¿Bueno eso es un cambio que ha mejorado, algún otro?

I: Hay un montón de cambios, pero no me recuerdo muy bien.

NV: ¿Que otros tipos de cambios ha habido, para entender un poco mejor?

I: Valla como esta muchacha licenciada que está enseñándonos esto, educarnos así en la alimentación, para educarnos más en la, con las enfermedades de uno. Eso es mejora para uno.

NV: ¿Y ese tipo de trabajo no existía antes?

I: No

NV: Que había antes:

I: Memmi nada.

NV: ¿Solo médicos?

I: Memmi sí.

NV: ¿Y por qué cree usted que han hecho estos cambios?

I: No podría decir

NV: Esta bien. Y en la atención medico por el largo tiempo, o sea quizás cuando era niña, ¿se nota algunos cambios?

I: ¿No, cambios? No le podría decir en qué forma. Solo cuando… hoy está más avanzadas las cosas, verdad hoy está mejor.

NV: ¿Como esta más avanzada?

I: Porque tenemos clínica nueva

NV: ¿Esta clínica?

I: Ya no esta nueva, ¿no? Pero esta mejor. Ya como era antes, cuando estaba en el pueblo.

NV: ¿Que había antes?

I: [silencio]

NV: Esta bien. ¿Usted noto algún cambio en costos de medicina o de atención medica?

I: No. De costos no. Bueno en aquellos tiempos cuando estaba más joven sí. Se pagaba antes. Antes antes, pero ahora ya no. Hoy es bonito.

NV: ¿Tiene algunas sugerencias para poder mejorar el seguimiento de la enfermedad?

I: Bueno yo digo que las cosas, que uno mismo tiene que ver cómo puede seguir adelante, que de uno mismo depende como mejorar la salud.

NV: ¿Y como hace usted para poder mejorar la salud?

I: Tomando la medicina a tiempo, cuidándome y alimentándome como dice la licenciada que comemos una dieta saludable. Uno mismo lo tiene que hacer.

NV: Estaba escuchando que ella habla de hacer cambios en el estilo de vida. ¿Que cambios son los que se tiene que hacer?

I: Pues, yo no sé qué forma será

NV: ¿Usted ha hecho algún cambio significado en estos años?

I: Es que la cosa con la dieta que uno tiene que hacer es este cuidarse como le digo yo. No comer sal, cosas que le caigan mal a uno para la presión, corazón, porque si uno se pone a comer de todo, de todo lo que haiga, y si no hay una, como le quiero decir, que haiga un cambio, tiene que cambiar, comer lo que no le haga daño para que nuestra salud este bien.

NV: ¿Y usted como sintió que ha sido ese cambio? ¿Lo sintió fácil o difícil?

I: Yo no.

NV: ¿O sea sintió algún cambio con diferente dieta?

I: No.

NV: ¿Que tipo de comida es la que se tiene que comer?

I: Eso nomas, verduras, fruta. Porque a veces cuando tenía sobre peso, este tenía bastante, entonces yo con lo que me había dejado la licenciada, comer fruta, verduras, para que pudiera ir bajando de peso. Porque así es lo que uno tiene que hacer, educarse en la comida, como le digo, ¿sí?

NV: Y eso, sobre eso, le han dado algún tipo de información sobre que otros tipos de cambios, ¿o así nomás con lo que dijo la licenciada?

I: Como, me lo dejaron a mí que lo hiciera. Si, entonces yo tenía que hacer de mi parte también.

NV: Muy bien. ¿Y en su hogar con sus hijos y familia, ellos han cambiado algún tipo de dieta o fue usted nomas?

I: [se ríe] Ay el que me lo que quede [¿?]

NV: ¿Y cómo es eso, es un poco difícil? ¿Porque Usted cocina para todos ellos también cocinan?

I: Este, uno como lo que, más bien lo que hay en la cocina, la papita, una sopita. Y de allí, tienen que ver que van a comer.

NV: Pero allí en la casa, cuando usted está comiendo la fruta, la verdura, quizás siente que bueno, si sus hijos están comiendo comida chatarra, ¿de repente le da algún antojo?

I: No, a ellas le gustan huevo, no les gusta comida chatarra

NV: ¡Ah bueno, está bien! ¿Y hay otros tipos de comida o bebidas que le han dicho que no debe de comer o tomar?

I: Yo tomo uno que otra vez. No me gusta lo que es el churro, la gaseosa.

NV: ¿Y su familia?

I: Ellos sí.

NV: ¿Ellos tienen alguna predisposición a las enfermedades o están bien?

I: [Se ríe] están bien.

NV: Que bien. Entonces para seguir, hay algo que usted piensa que quizás quisiera que ha sido diferente, ¿o algo que usted piensa que pudiera mejorado?

I: Bueno no le voy a decir. Si mejorar [se ríe]

NV: ¿Tiene alguna recomendación, no solo para la unidad de salud, pero para cualquier servicio médico?

I: Todo está bien.

NV: Esta bien. Muchas gracias, allí terminamos la grabación.

End of interview.

# Staff interviews

## Staff interview 1: Departmental coordinator

Entrevista con: Coordinador Departamental

Fecha: 13/06/18
Lugar de la entrevista: Unidad Comunitaria de Salud Familiar Jiquilisco
Código del informante: SMML005
Genero del informante: M
Nombre del entrevistador: ML

**I: ¿Como es el funcionamiento del SIBASI?**

El funcionamiento de Sibasi (sistema básico de información en salud), somos como una raíz de la región oriental con sede en san miguel, la región de salud está constituida por sibasi de Morazán, la unión, san miguel y Usulután. Tenemos una coordinadora de sibasi, que es la doctora, nosotros estamos encargados de todas las unidades del departamento, tenemos 23 municipios, que son 90 establecimientos de salud, entre especializada, unidades intermedias y básicas.

**I: ¿Nos puede indicar el número de cada una de ellas y las microrredes que componen el sibasi?**

Son 25 unidades intermedias, 57 unidades básicas, 4 unidades especializadas y también el lugar de espera materna en San Miguel. Las dividimos en 5 microrredes, está la Microred de Jiquilisco que lo componen los municipios del propio Jiquilisco (municipios de Jiquilisco san francisco Javier, san Agustín y puerto del triunfo); también la Microred de usulutan ( y la componen los municipios de usulutan, san Dionisio, Sta. Elena, oxactlan) también la Microred de concepción Batres, que la componen 3 municipios, la Microred de Santiago de maría que la componen los municipios de Santiago de maría, decapan, california, u cuaca, san buena ventura y el triunfo, la Microred de Berlín que están los municipios del propio Berlín, nueva granada, alegría y otros dos municipios más.

**I: ¿Cómo funciona sibasi con las microrredes?**

En el sibasi también tenemos equipos territoriales compuesto por médico, enfermera, inspector de saneamiento, una estadística , promotor de salud comunitaria, la conexión territorial del sibasi con las microrredes está a cargo de este equipo de territorio que son los encargados de monitorear y ver funcionamiento de toda la Microred, en el caso del equipo territorial 1 son los encargados de la Microred de Santiago de María y Berlín, el equipo 2 encargados de la Microred de Jiquilisco, el equipo 3 encargados de las microrredes de usulutan y concepción Batres, la comunicación esta entre el equipo de territorio y la Microred, de ahí la comunicación se hace a través de las reuniones mensuales, las unidades municipales ellos se reúnen con sus ecos generalmente la primera semana del mes, suele ser el miércoles, la segunda semana se reúnen todas las microrredes, por lo que todos los temas que se tratan en la Microred municipal, luego se discuten en Microred intermunicipal, donde están reunidos todos los municipios y de ahí hay una tercera reunión donde solo van la cabeza de cada Microred, es importante aclarar que en cada Microred hay un responsable del nivel local que se le llama cabeza de Microred, que suelen ser médicos , ( se va de las reuniones de las eco, municipales , intermunicipales, Microred) y la cuarta reunión que es la red departamental, el tercer miércoles de cada mes y la coordinadora de sibasi ve todos los problemas expuesto en las reuniones anteriores y en la cuarta reunión que es la región oriental donde cada coordinador del sibasi expone todos los problemas, y de esa manera fluya toda la comunicación por ultimo otra reunión de los directores regionales que van a reunirse a nivel central con el ministerio de salud.

Por ejemplo Puerto del Triunfo es una Microred de Jiquilisco, entonces esa es la reunión municipal, de ahí el coordinador de la unidad de Puerto del triunfo se reúne en la intermunicipal, y el coordinador de la intermunicipal o del sibasi se reúnen aquí en la red micro departamental y ahí solo vienen los cabezas de Microred y los hospitales, los directores de hospitales, cabezas de enfermería, los epidemiólogos del hospital; por ejemplo aquí seria el hospital de Jiquilisco, Santiago de María y Usulutan, los tres hospitales que también componen el SIBASI.

**I: Relacion microredes y hospitales**

Se tratan puntos especificos: las referencias y retornos, los pacientes que se derivan al hospital y luego retorno para seguimiento local y comunitario, unas de las cosas importantes que quiere el ministerio que se trate, es la morbimortalidad materno-infantil, indicadores de infecciones vias urinarias y vaginosis en las embarazadas y periodontales también, situaciones que no se solventan en las reuniones de microredes.

**I:Recorrido del paciente cronico**

Nos enfocamos con los enfermos renales, nuestros ojos comunitarios son los promotores de salud, ellos identifican a los pacientes con algún factor de riesgo, la obligación de ellos es referirlo al eco para que el medico haga su evaluación médica, abastecimiento de medicamentos, programar visitas a nivel domiciliar, si necesita evaluación extra como psicólogo, nutricionista, nefrólogo, médico internista...ellos lo puedan referir, si el médico internista lo ve y decide en ese momento quedarse con el paciente o el paciente está controlada y lo retorna a nivel local para su seguimiento y abastecimiento, a nivel comunitario. Los análisis se hace en el eco y se responsabiliza el eco en llevar la muestra y recoger los resultados, hoy es la muestra la que viaja, antes eso es lo que ha cambiado, viajaba el paciente y no la muestra, hoy viaja la muestra con el ECO, ellos son los responsables del embalaje de la muestra, y lo llevan a la unidad donde existe el laboratorio, el ECO también es el resultado de traerlos y visitar a su paciente cuando ya este con la muestra.

**I: ¿Las unidades intermedias todas tienen laboratorio?**

Algunas, no todas, nosotros tenemos 16 laboratorios clínicos en las unidades intermedias.

Los pacientes también pueden acudir en la modalidad espontánea al eco y una vez en la consulta de preselección la enfermera toma los signos vitales y anota alguna anormalidad y lo pasa al médico, o lo contrario cuando no es identificado por la enfermera, el medico lo evalúa ahí y ya sea a través del examen médico o examen de laboratorio requiera una mayor evaluación, le manda lo que el necesite para que le sigan.

RECORRIDO EN EPISODIO AGUDO

A veces se detecta por el promotor si está el paciente esta en su casa y lo refiere al establecimiento, y también puede acudir el paciente de forma espontánea al eco y el medico primero lo tiene que estabilizar y luego referir.

ACERCAMIENTOS DE LOS MEDICOS A LAS COMUNIDADES

Los médicos tienen programados los acercamientos que hacen a las comunidades evalúan a los pacientes e identifican a los pacientes y ya lo dejan en el control, en el acercamiento, visita domiciliaria y también en la visita de atención integral familiar en el domicilio, porque la dispensarización es individualizada, pero la integral es ver a la familia, es ver todo el entorno se ve la infraestructura de la casa, la situación sanitaria, ver problemas de higiene, problema de vectores ..... y evaluación del paciente, hay lineamientos para abordar cada visita. El promotor previamente tiene identificada la dispensarización.

Cada promotor de salud tiene identificada cada familia por alta, medio y bajo riesgo ya sea por un factor médico o ambiental, hay un mapa identificado, cada eco tiene aproximadamente unas 200 familias, y en función del riesgo se programan las visitas, que se puede realizar por el promotor de salud , se programa el día de visita y que profesionales le van acompañar, puede ser el médico, el inspector de saneamiento, la enfermera, psicólogo, promotor, como es una programación yo puedo ver quien me acompaña en mi visita.

**I: ¿Se considera tambien como riesgo la violencia?**

Si se considera el riesgo social.

**I: Control de los regresos de los pacientes ya diagnosticados**

Tenemos un Sistema de información y cuando al paciente se le da el alta aparece en el sistema y nosotros aquí en el sibasi estamos encargados de bajar diariamente los regresos y nosotros los enviamos a las unidades por vía correo para que estén enterados para poder visitar al paciente en su casa, hacer la visita domiciliaria, ver indicaciones que le dio el medico, y dejarlo en control del establecimiento, visitas domiciliares o controles en la sede del eco.

**I: Priorizacion de las actividades en el abordaje de las enfermedades cronicas**

Tenemos en primer lugar la sala situacional, nosotros como sibasi la realizamos cada 15 días y se hace vigilancia de las enfermedades prevalentes y no prevalentes, diarreas, neumonías... y enf. crónicas y dependiendo del numero de casos nosotros decidimos que actividades se deben realizar, por otro lado, tenemos los indicadores la evaluación es cada tres meses, trimestral, se evalúa de enero a marzo se crea plan de mejora, de enero a junio se crea otro plan de mejora, de enero a septiembre y a final de año de enero a diciembre se hace la evaluación comparativa con el año anterior.

Los indicadores ya traen sus componentes. El primer componente a la atención a la persona a la familia y a la comunidad, el segundo componente promoción a la salud, el tercer componente gestión de medicamentos, el cuarto gestión del servicio de salud, el quinto financiamiento (hospitalario), el sexto mecanismo de coordinación en los servicios de salud, el siete de la niñez, el octavo adolescente, el nueve de mujer en edad fértil, el 10 de mujer embarazada, el 11 adulto mayor, el 12 de enfermedades no transmisibles priorizadas. En el 12 se ve el porcentaje de pacientes hospitalizados por diabetes mellitus no controlada por complicaciones, de trastornos mentales comunes, trastornos mentales graves, intentos suicidas, letalidad de diabetes mellitus, mortalidad por HTA, mortalidad por cáncer de mama, mortalidad por cáncer de cérvix, mortalidad por cáncer de próstata, mortalidad por insuficiencia renal y mortalidad por diabetes mellitus. Esto se realiza trimestralmente a nivel de SIBASI, consolidamos por municipio y luego a nivel regional consolidan por departamento; a nivel de microred se socializan los resultados de los indicadores; las microredes socializan con municipios y los municipios socializan con ECOS; tanto los planes de mejora, como los resultados de los indicadores.

**I: Abordaje desde la intersectorialidad**

La intersectorialidad es parte de la reforma de salud, que lo que se quiere con este objetivo es que para que los compromisos que nosotros asumimos como salud debemos apoyarnos en las otras instituciones que también trabajan en pro de salud en su área de responsabilidad, nosotros somos los responsables de que funcione en un comité intersectorial donde ubicarnos a nivel local puede estar conformado por los directivos de los diferentes ADESCOS, alguna ONG, que este en la zona trabajando con la misma población, también pueden ser directivos de escuelas, maestros, institutos de educación, la policía, otras instituciones de la zonas pedimos que se reúnan periódicamente para darles a conocer las dificultades, la vigilancia epidemiológica de las enfermedades, y otros acontecimientos para trabajar en conjunto, para así algún problema que tenga alguna institución lo pueda solventar con otro.

**I: ¿En este comité intersectorial también participa la comunidad?**

Si claro, están los directivos de los ADESCOS, cada comunidad constituye un ADESCO y también el foro nacional de salud, dependiendo donde tenga la sede el foro, por ejemplo, aquí en la zona Norte de Usulután, que ahí está el foro, para el tema de la contraloría.

**I: ¿Quien convoca las reuniones?**

El ministerio de salud. También dependiendo del empoderamiento que se tenga en el comité, porque hay unidades de salud que han empoderado mucho a las instituciones y son ellas las que han tomado el mando del comité y son ellas las que están convocando las reuniones, tienen su agenda, los compromisos, los acuerdos, las actas, todos ellos la llevan.

**I: ¿Qué función tienen los comités de salud?**

Son parte de la reforma con mucha participación ahora, y ya se han constituido formalmente, son personas altruistas de la comunidad que trabajan solo por la satisfacción de poder ayudar y apoyar en la salud de su comunidad, también se reúnen en la intersectorial y con los establecimientos, apoyan sobre todo a los promotores de salud de la zona.

**I: ¿Como cree que la participación comunitaria fortalece el funcionamiento de la riis?**

La misma participación comunitaria nos hace ver cosas que nosotros creemos que las estamos haciendo bien, ellos tienen esos espacios en los establecimientos de salud o en una asamblea comunitaria a exponer esas dudas o dificultades que ellos han tenido al visitar esas unidades de salud, nos hacen ver en las cosas que nosotros no hacemos bien para poner cartas en el asunto y como tratar de mejorar o coordinar con ellos alguna situación particular y poderles apoyar.

**I: Barreras y facilitadores del sibasi para realizar su función**

Somos el sibasi más grande del Salvador, hay otros que tienen 23-25 municipios, y nosotros contamos con 90, solo contamos con tres equipos de territorio y además que somos también 5 microrredes y solo dos equipos de territorio, nos faltarían otros dos equipos de territorio, es difícil realizar todas las supervisiones, seguimientos y llevar todos los programas que en MINSAL pide, al píe de la letra. La dificultad más grande es el acceso porque las unidades están muy retiradas, hay muchas zonas rurales, con riesgo y sobre todo en épocas de lluvias, también falta personal. Ese es el problema más grande en función del número de personas que trabajamos aquí, y la distancia a la unidad de salud.

**I: ¿Que facilitadores ve usted en el manejo del paciente crónico?**

Los facilitadores: es el trabajo en red, no es tan distanciado el seguimiento, ha mejorado la comunicación entre el primer nivel y el hospital, en ambos sentidos la comunicación es más fluida, estamos más pendientes; los hospitales nos comunican de estos pacientes y nosotros desde las unidades también a los establecimientos y, por el contrario, nosotros hablamos con el hospital y de acercarle el paciente, entonces lo ingresan y lo tienen ya controlado. Entonces antes no se tenía esa comunicación, a veces mandaban al paciente de alguna unidad de salud, entonces no teníamos esa reciprocidad, y también los médicos tienen más conciencia a veces que se refiere a pacientes de muy lejos, de muy rurales, o zonas de difícil acceso, entonces ya el medico cuando llega de lejos al hospital, ya lo piensa un poquito en retornarlo, en eso pues hemos estado mejorando, no solo funcionan las microrredes sino también tenemos los comités de referencia de retorno, donde nos sentamos un grupito de personas y vemos todo el proceso de la referencia y tenemos reunión cada mes. También hay comités de materno infantil, abastecimiento de medicamentos, enfermos crónicos…, aquí está implicado personal del primer nivel y el hospital.

**I: Retos en el primer nivel para mejorar el manejo del paciente cronico**

Quizá el principal reto que el ministerio tiene es el recurso de especialistas, porque nosotros a veces nos enfrentamos a que referimos a nuestros pacientes a nivel hospitalario y lo que nos espera es que a los pacientes nos los citan entre 3- 9 meses porque hay pocos especialistas a nivel de hospital; por lo que ese es el reto tener especialistas a nivel del primer nivel y en hospital, este sería uno de los retos aumentar el número de especialistas, para que no nos los citen con visitas bien prolongadas.

**I: Retos en el primer nivel en relacion a la prevencion y promocion de la salud**

Hay un buen plan de abordaje en cuanto a la prevención y la promoción, el problema es quizás cultural en la población, hay buen número de charlas en los establecimientos, participación en las asambleas comunitarias, ferias de la salud.... a veces la parte cultural de la población, falta de concienciación y rechazan o buscan otras alternativas de tratamiento y esto al final nos perjudica porque nosotros intentamos dar atención integral al paciente, y a veces falta a su cita, buscan otro médico o médico naturista...

**I: ¿Hay abastecimiento de medicamentos?**

Hay comité de fármaco-terapéutico ,y en cada establecimiento de salud hay un responsable o referente de consolidar la información, del abastecimiento de medicamentos, cual es el medicamento no disponible, cual medicamento le va a vencer, cual medicamento puede transferir, cual medicamento puede aceptar una transferencia , esa persona encargada del eco se reúne con su unidad intermedia y ellos exponen esa situación, si ellos no logran resolver esa situación esa información pasa al comité fármaco terapéutico de la Microred y si lo tiene hacen la transferencia, como Microred también consolidan esa información y también va al comité fármaco terapéutico departamental y si la Microred no tenía ese medicamento desde todo el departamento lo tienen que tener y ellos mismos lo transfieren, los referentes que vienen ven la necesidad y ellos mismos hacen la transferencia antes de llegar a la región, puede existir algún tipo de falta de información entre los establecimiento que se logra de solventar de esta forma.

## Staff interview 2: Intermunicipal coordinator

Entrevista con: Coordinador Intermunicipal

Fecha: 13/06/18
Lugar de la entrevista: Unidad Comunitaria de Salud Familiar Perquin
Código del informante: SMMJ005
Genero del informante: M
Nombre del entrevistador: MJ

**Sección 1**: Perfil del participante y función en la prestación del servicio del primer nivel de atención

**I: Tiempo que lleva como coordinador y perfil profesional.**

Entrevista con XXX, coordinador intermunicipal (de Microred) y director del establecimiento hace unos 7 años, he conocido bastante el equipo especializado, lineamientos y nivel de la base de la reforma de salud, también coordino la casa de espera materna, y también he estado anteriormente en FOSALUD que es parte del ministerio, también en la unidad de Torola que es una unidad intermedia.

**I: Desde cuando funciona esta unidad, y cuando se hizo especializada**

Esta unidad es del 2004-2005, se hizo especializada en el 2010, que empezó el equipo especializado al principio no tenía un cubículo para consulta y en el 2013 ya se hizo el módulo del equipo especializado y ya cada quien tenía su área de trabajo con las herramientas necesarias para poder funcionar.

**I: ¿Cuál es su función actual en el primer nivel de atención?**

Como coordinador de la Microred realizo mi actividad en la unidad especializada, también coordinación de atenciones con el ministerio de salud así en el día, nosotros trabajamos en horario de 6 de la mañana a 6 de la tarde y también en la unidad de FOSALUD que es de 6 de la mañana a 6 de la tarde, todos los días de la semana y los fines de semana trabajamos 24 horas con FOSALUD, también se coordinan actividades con los hogares de espera materna que también funciona 24 horas al día durante los 365 días del año, esto es en general en el establecimiento. Acá está el equipo especializado donde se lleva con ellos la coordinación en la Microred, también esta FOSALUD que también se coordinan todas las actividades porque en verdad también están en el establecimiento que esta igualmente como nosotros trabajando, por lo que tenemos reuniones periódicas con FOSALUD para coordinador actividades con el equipo de ministerio de salud de acá, lo que me corresponde parte de consulta verificar que todo esté funcionando bien en cada área y coordinar los médicos , referencias también, con la Microred se ve algunos lineamientos que son del ministerio en la reunión con Microred. La reunión de Microred se hace una vez al mes, y con SIBASI y se coordina todas las actividades y lineamientos que indica el ministerio, en esas reuniones tenemos intervenciones de veteranos de guerra, porque en la zona tenemos bastantes veteranos de guerra, también vemos con el foro nacional de salud que participa con nosotros en las reuniones, también se hace programación anual que se ve en cada establecimiento , las referencias, y los retornos que se mandan al hospital, también está la parte materno infantil donde se ven algunos planes como la reducción de la mortalidad, el de vaginosis, y otras cosas que hayan ocurrido como la morbilidad infantil extrema que se pueda abordar, es decir hacer una auditoria, también se parte de morbilidad obstétrica en embarazadas que también si es necesario se audita en esa reunión, también se ve la parte de los medicamentos como están abastecidos los establecimientos de la Microred y si algo se necesita se hace transferencia de los medicamentos a otras unidades dependiendo de la cantidad que ellos tengan y ahí se coordina y se apoya, también se hace control de las referencias que se envían para tratamiento para el cáncer el hospital de referencia se ve el seguimiento que da a los pacientes ya en los municipios, también hay un punto de control del hogar espera materno y se ve las pacientes que se han enviado al hogar materno de cada establecimiento de la Microred y hay un seguimiento de cómo fue el parto, si fue cesaría , vía vaginal, si se quedó ingresada dependiendo porque previo a eso, ellos me envían la lista de las embarazadas que van a verificar el parto; por ejemplo de la unidad de Arámbala, me enviaron tres y si en el hogar de espera materna solo tengo 2, ¿qué paso con la otra?, ¿dónde está?, ¿dónde tuvo al niño?, entonces cosas así, también coordinamos si un establecimiento se queda sin ambulancia y el que este más cerca tiene ambulancia ese apoya a ese establecimiento en emergencia para poder solventar más rápido, entonces hay un flujograma para saber a qué establecimiento hay que llamar en el momento de la emergencia, estas actividades se ven en las reuniones de Microred.

También está la red departamental también se ven puntos parecidos a los de la Microred y si algún punto no se pudo resolver en la Microred se presenta en estas reuniones, y se ve con el SIBASI, y ya ellos nos dicen que solución o si se lleva a la red regional también hay comités maternos, de farmacovigilancia, y de referencia-retorno, vemos casos y su necesidad.

**Sección 2**: Comprender las trayectorias de los pacientes en el primer nivel de atención

**I: ¿Con que hospital estáis en coordinación?**

Con el hospital de Gotera es nuestro hospital de referencia que es el segundo nivel, y ellos valoran si hay que enviarlo al hospital de San Miguel que es el tercer nivel, pero en algunas ocasiones si por ejemplo hay algún problema oftalmológico que nosotros sabemos que en el hospital de la gotera no hay oftalmólogo nosotros le derivamos al hospital de San Miguel, para no demorar la atención del paciente o algún paciente psiquiátrico que nosotros sabemos que ellos no cuentan con psiquiatra lo enviamos al tercer nivel, dependiendo.

**I: ¿como es la coordinación de las microrredes?**

Nuestro departamento cuenta con 5 microrredes y cada Microred cuenta con un coordinador, cada Microred hace su reunión y al final el último viernes de cada mes vamos a la reunión de la red departamental y aquí solo vamos los coordinadores de cada Microred, se hace en SIBASI que está en Gotera, que ya han transcurrido todas las actividades de ese mes y se ve si ha habido alguna dificultad, aquí si está presente el hospital , la región, SIBASI, cada quien de cada programa y en base en eso se coordina.

**I: ¿Cuál es el recorrido de un paciente crónico dentro de la Microred? donde se le hace el primer diagnostico?**

Por ejemplo un paciente crónico que tengamos, viene a la consulta general del establecimiento y le ve el médico general y se le explora, verifica los signos vitales y ve por ejemplo que la presión y si la tiene alta, se mide la frecuencia cardiaca y varias tomas de presión arterial en días alternados, se les deja un hojita para las distintas tomas de presión y si vemos que la mantiene alta ya le podemos catalogar como una persona hipertensa, entonces ya el medico inicia el tratamiento con la dosis necesaria; y en algunas ocasiones se manda exámenes complementarios, de función renal, porque algunos pacientes que ya son diabéticos y tal vez no se han acercado a ningún establecimiento y no sabían que eran además hipertensos y habían estado un montón de tiempo sin tratamiento, entonces se le mandan los exámenes y el médico general valora en las consultas interconsulta con el especialista o el médico internista o de familia, y ya valoran las dosis necesarias y ven los exámenes, si esta descompensado el paciente, no se controla la presión, el médico especialista se queda con el paciente y lo mantiene en control. Estos procesos también son vistos por la educadora y la nutricionista, que ellas les dan su manejo según lo que ven, por ejemplo, si algún paciente que tiene triglicéridos altos, colesterol, la nutricionista les hace una dieta, la educadora les explica que tienen que hacer y que no, entonces los manejamos de esa forma. Si el paciente viene muy descompensado y no se controla si se le envía al hospital de Gotera que es nuestro hospital de referencia. Si el médico de familia ve que el filtrado glomerular, la creatinina está demasiado alta hacemos referencia a consulta externa al hospital de segundo o tercer nivel, nosotros sacamos cita para el paciente, para que el paciente de acá pueda ir para allá, sin que el paciente tenga que ir hasta allá solo para sacar la cita, entonces tratamos de reducir eso, porque a veces tardan todo un día, o no lo conocen, o no tienen los recursos necesarios, entonces nosotros valoramos eso y hacemos comunicación con la consulta del hospital

**I: ¿Como de haría la búsqueda de casos?**

Todos los pacientes que vienen acá es tomado los signos vitales, en algunas ocasiones hacemos actividades por ejemplo la del adulto mayor, que es más frecuente en algunos casos, entonces hacemos un tamizaje en ellos para identificar algún riesgo que puedan tener, esta actividad la hacemos 1 ó2 veces al año con adultos mayores, para identificar alguna situación que no se haya podido, porque en algunas ocasiones el paciente se siente mal y no acude al establecimiento y en estas actividades se aprovecha para detectar.

El promotor de salud también nos ayuda bastante, porque ellos en la comunidad son los que más conocen a las personas, entonces el promotor de salud lo que hace es mandar una hojita de referencia, entonces toma los signos vitales y la presión si tiene alguna alteración se envía al médico y en ocasiones se retornan con un manejo el paciente al promotor para que le haga el seguimiento.

Los pacientes que están controlados y todos los meses vienen a por el medicamento, hay un programa de receta repetitiva que se da por tres meses, entonces ya en ese programa se le toman los datos al paciente en farmacia y el día que viene a por el medicamento se le da la receta del día y tres más y se le indica que está controlado, cuando viene a retirar el medicamento siempre se le toma la tensión. El programa de receta repetitiva lleva unos dos años, entonces ya tenemos a varios pacientes, no a todos, porque no todos cumplen, por ejemplo, hay pacientes diabéticos que se les deja la glicemia cada dos meses, entonces un mes la tienen bien, entonces eso no lo podemos dejar, entonces dependiendo de lo que valore el médico y lo responsable que sea el paciente.

**I: Cuando el paciente no acude a consulta ¿cómo se gestionan esos casos?**

A través de los promotores de salud, nosotros tenemos un listado de los pacientes diabéticos y de hipertensos en el municipio, entonces la médica de familia tiene también un listado de los que ella ve; entonces si no acude el paciente hace mucho tiempo, el promotor le hace una visita a ver que paso y en base a ello valora. Si el paciente es de otro municipio, también se reporta a su unidad para que lo tengan en cuenta. También, el equipo especializado visita a todos los establecimientos de la Microred prácticamente se conoce a todos los pacientes.

**I: ¿Cuáles son los desafíos que se encuentra para cumplir su función en el día a día?**

Mayormente nosotros en esta unidad es la demanda que es muy alta, si fuera solo los del municipio sería otra cosa, se recibe también paciente de Honduras y de otros municipios. En general estamos bien de recursos, tenemos incluso medicamentos del hospital, de última generación por ejemplo de hipertensivos tenemos el irbersartán, enalapril, amlodipino… En verdad, nuestro establecimiento solo debería atender a la gente de este municipio, lo que pasa que hay gente de otros municipios que les gusta venir acá, porque el establecimiento trabaja 24 horas y siempre hay un médico para atenderlos, porque FOSALUD también trabaja dentro del establecimiento, entonces prácticamente no cerramos la puerta, entonces las personas vienen y siempre se les atiende, en otros establecimientos (intermedios o básicos) se trabaja de 6:30- 15:30 entonces ellos dicen “ ¿y si el doctor ya no está?”, entonces tienen unos horarios más reducidos y también como el establecimiento es más grande los pacientes se creen que hay otros medicamentos....La idea es que cuando lleguen los especialistas a las unidades básicas o intermedias tengan la mayor parte de pacientes que estén descompensados, además de aquellos que se les haya ingresado en otra ocasión. También nosotros tenemos laboratorio, odontología, médico internista.

**I: ¿Qué estrategias que se podrían diseñar o aplicar en los establecientes intermedios o básicos para que no haya tantos pacientes acá**

Mas que todo es por el horario extendido que nosotros tenemos, entonces los pacientes vienen de un solo vienen aquí. Creo que solo es eso, por yo creo que los establecimientos tienen bastante control de sus pacientes, incluso cuando viene aquí el paciente ellos ya saben lo que tiene ese paciente. Pero algunas veces los pacientes deciden a donde ir.

Yo pienso que la estrategia que nosotros estamos llevando a cabo con la receta repetitiva, con las fichas familiares, transferencias de medicamentos que tienen los demás establecimientos y el control de los pacientes es bastante bueno; una estrategia mejor es bien difícil, porque tenemos a los especialistas que se acercan al establecimiento, tenemos los medicamentos y por ejemplo un paciente que llega a un establecimiento básico o intermedio, y allí no tienen un medicamento, entonces ellos me llaman a mí y me dicen “aquí tengo un paciente que viene a retirar un medicamento pero no tengo”, entonces yo les hago la transferencia del medicamento, y si solo es un medicamento de un paciente que tiene ese medicamento, ellos llaman acá y yo autorizo la receta y se dispensa, y así se resuelve.

**Sección 3:** Trayectorias de cuidado de personas con enfermedad crónica

**I: para usted en la consulta a pacientes crónicos, ¿qué cree que pude ayudar al paciente crónico?**

En caso de nosotros que somos especializadas además de la consulta que nosotros le brindamos y consejería, no tenemos tanto tiempo para estar explicando al paciente; contamos con la educadora y nutricionista y también tenemos un club y aquí tocamos bastantes temas con ellos, porque ya no es adulto mayor, sino integrado, y damos un pequeño espacio y la mayoría de los pacientes son bien abierto a hablar, entonces yo pienso que las experiencias de uno pueden ayudar a los demás, son grupos de autoayuda, esto siempre se ha hecho, además también participan otras instituciones como los que llevan la cultura, también la fisioterapeuta, para el ejercicio físico, les da algunas técnicas, y a ellos les gusta mucho. Estas reuniones las programa la enfermera, y según el tema que se vaya a desarrollar ese día la apoya la nutricionista, fisioterapeuta…, y suelen acudir bastante, en el último han acudido 30 o 40 pacientes. Antes teníamos un grupo más grande porque teníamos un poco de financiamiento por la alcaldía, y llegamos hasta más de 40, pero ahora sin el financiamiento es menos. Siempre intentamos de busca algo para que coman, pero ya no es tanto que tuvieran algo.

I**: Barreras con las que el paciente se suele encontrar frecuentemente a la hora de seguir su seguimiento**

Si una cosa que yo me he dado cuenta es que muchos de los pacientes, los recursos de ellos no son tan buenos, entonces este, no podemos recomendar cosas que no puedan, entonces cosas que puedan llegar fácilmente para su dieta, como fruta, y luego también puede ir a la nutricionista que tampoco le va a pedir cosas extraordinarias, que vaya a un super a por esta y esta otra cosa, pero si la parte económica es a veces complicada, pues sí, porque hemos tenido a veces adultos mayores, con problemas, entonces nosotros tenemos un pequeño grupo que coordina, ese grupo veíamos las necesidades por ejemplo tal paciente ya no se levanta, ya no nada, entonces le hacíamos una visita el grupo y tratábamos de coordinar algunos alimentos o cosas así, en ese grupo hay personas que no tienen a nadie, por ejemplo teníamos un señor que no tenía casa donde vivir, porque vivía arrimado a otro y no lo cuidaba, entonces en esta ocasión coordinamos, conseguimos un espacio y entre todos le construimos una casa, y fuimos y le hicimos una habitación y se la dejamos al señor, hicimos dos nada más e hicimos eso una reunión, y veíamos algunas situaciones, donde hacía falta hacerle una visita a las personas, llevarle algo, alegrarle, y eso es porque la situación económica afecta, también la situación familiar, nosotros intentamos trabajar con la familia, porque a veces por ser señores, y estar en el trabajo, nadie le pone mucha atención, entonces a veces se trabaja con quienes cuidan, de llevar un adecuada alimentación saber cuándo tomar la medicación, por cuanto tiempo y tal. Mire, por ejemplo, el hombre que le decía de la casa parecía que donde vivía, no vivía muy bien, con muchos problemas con el que vivía.

**I: ¿El tema del deporte como esta organizado?**

Si, pero el deporte, es algo que lo ocupan como los mas jóvenes, entonces generalmente para el adulto mayor, la fisioterapista lo que hace es como ejercicios, porque los deportes están como.

**I: ¿Y la violencia social afecta mucho en esta zona?**

Fíjese que menos, es una zona un poco más tranquila, y eso favorece digo yo, un ambiente más tranquilo. Yo creo que lo más importante que ha sido es la organización de las personas, en esta zona a mí me gusta trabajar acá, porque son muy organizados y todos participan, entonces cuando ellos ven alguna situación todos están pendientes, hay muchos líderes que siempre están para colaborar, entonces a veces también tenemos reuniones intersectorial, donde ahí se plantean algunas de las situaciones, y la policía está realmente bien atenta a esas situaciones y hacen un llamado o cualquier cosa, y eso prácticamente ayuda a la zona. Yo el tiempo que he estado acá es una zona tranquila.

**I: ¿Que apoyos tiene usted personalmente, profesionalmente y también en su comunidad para abordar más la enfermedad crónica?**

Esa parte que yo le comentaba de estar organizada es la intersectorial, porque participa la alcaldía, participa la iglesia, participa algunos líderes, el establecimiento de salud, entonces en algunas ocasiones educación, pero en la parte intersectorial, si esta la policía, protección civil, casa de la cultura, la alcaldía que le corresponde, salud, educación, la iglesia, entonces así eso nos ayuda para resolver alguna situación, por ejemplo, si tenemos alguien en la escuela que sea por ejemplo una adolescente embarazada que iba a la escuela, entonces hablamos con el director, y le decimos “mire que aquí tenemos que hacer más intervenciones en pro de evitar los embarazos en adolescentes, y entonces organizamos una actividad con la escuela, sin hacer daños a terceros, entonces es una intervención que nosotros planteamos en base a eso, y vamos trabajando, por ejemplo si es de seguridad entonces con la policía. Entonces por ejemplo nosotros, gracias a dios por las reuniones y la coordinación la policía siempre está atenta a la llamada de nosotros, y la alcaldía.

Si se tiene un grupo de reunión con la comunidad, por ejemplo en la casa materna, hay que hacer muchas actividades de reposición, y mire que falta esto, falta lo otro, entonces en relación a gente que no quiere venir acá, hay que darle ayuda y decirle, mire esto es así, esto es asa, pero no es lo mismo que yo lo quera llevar o si alguien de la comunidad dice yo lo traigo, ahí es más fácil entonces ellos nos apoyan bastante la comunidad. Y ahora que se ha incluido el foro nacional si nos está ayudando mucho, al principio fue un poco complicado, yo me acuerdo cuando empecé, le voy a contar un poco la historia.

Fíjese que yo al principio que vine no había reunión de micro red, entonces yo empecé a ver que por ejemplo tenía un compañero que me pedía ayuda, entonces yo le ayudaba, entonces entre todo vimos que era necesario que nos reuniéramos, entonces este, yo solicite permiso una vez para reunirnos, incluso yo hasta ni sabia hasta donde podía llegar la Microred entonces llame a otro, entonces vienen y nos dijeron “ ¿y qué es lo que quieren hablar?”, no queremos ver que podemos llegar, entonces no venían de sibasi y solo nos dejaban reunirnos 3-4 horas, al principio, los médicos, 3-4, nosotros éramos bien críticos y discutíamos varias cosas, y así empezamos la microrred, algunos a veces peleaban, luego contentos, luego peleaban otra vez, y tratábamos de hablar, porque lo que tratábamos era de mejorar, entonces después, este, nosotros veíamos como necesario la reunión, entonces nosotros dijimos que era muy poquito tiempo para discutir algunos puntos que teníamos que discutir y tres horas no eran suficiente, porque teníamos que ver muchas cosas, me acuerdo cuando empezaos con un sistema inventado de referencia y retorno, y de ahí también veíamos la parte epidemiológica, entonces este y otras cositas más. Antes las especializadas eran diferentes, no tenían espacios y se iba a muy poco establecimiento, antes no había casi interés de coordinación, esto era cuando iba comenzando la reforma, antes era un quebradero de cabeza, porque si se tenía que ir a un sitio no había transporte, era un dolor de cabeza, y como yo sabía que había gente esperando, todos los días era un dolor de cabeza. entonces cuando empezamos con la reunión de microrred dije yo, no, lo primero que vamos a hacer vamos a programar los carros, porque a mí me duele la cabeza, todos los días por la mañana como voy a mandar al equipo especializado y ellos perdían mucho el tiempo acá y cuando iba allá habían perdido un montón de pacientes. Entonces con la reunión de microrred empezamos con la coordinación y programación, primero hicimos una reunión mensual, pero igual que el siguiente mes me costaba, entonces dije yo, no, eso no puede ser así, vamos a hacer una programación anual, y tomando en cuenta las fechas patronales, entonces hicieron una programación anual del equipo especializado, porque les dije yo, imagínense que llega un paciente hoy, y le dice la doctora para el seguimiento que no sabe cuándo le a ver en dos meses, entonces es una dificultad, entonces dije de hacer la programación anual, para que puedan llegar el grupo especializado una vez al mes a las comunidades teniendo en cuenta las fechas patronales, y los vehículos, programar los vehículos. También, antes se daba la dificultad que le decían al paciente “mire yo no le voy a citar a tal especialidad, porque no tengo pacientes para él”, pero como no va a tener pacientes para especialidad si hay un montón de gente, entonces después creamos que tenían un agendamiento de cada quien a quien podía ver, entonces eso los discutíamos también en las reuniones de Microred, veíamos esto, que era necesario ver, no porque hacíamos esto, y porque, “es que no tengo a ninguno”, no tiene ningún niño por ejemplo para que vaya el pediatra, no creo, entones yo empecé a exigir un poco a esa parte, de que tenían que tener a pacientes citados, entonces al principio quedaba allí toda esa información, y luego creamos un hoja en donde se dejaba una copia acá y otra allá, entonces cada quien me ponía a cuantos citaron, cuantos él había dejado citados y cuando vinieron, y cuantos dejaron citados para el próximo mes, y se les había dado interconsulta, entonces todo eso no teníamos, si ocurría algún inconveniente, si se reunieron al final, si dieron charlas. Por ejemplo, otra cosa es que aquí el traslado se demora 30- 45 minutos, de distancia, entonces allá le tienen que tener al menos 3 o 4 pacientes preparados, y eso es lo que viene reflejado en el informe.

**I: ¿ Esto fue algo innovador por su parte?**

Pues fíjese, al principio vino aquí la Dra. XXX. Pero bueno luego era un problema, porque el equipo especializado iba, pero a veces el carro como tenia que venir desde por ejemplo Torola, tenia que venir a traerlos, dejarlos, y llevarlos de nuevo a Torola, y yo sentía que era como doble gasto, a veces el carro se tardaba como demasiado tiempo, y yo no podía decirles venga apúrense, porque es una ambulancia, entonces yo lo que hice, fue conseguirles un carro especializado para que ellos se puedan mover, entonces yo les dije, mire este carro, ustedes lo llevan y lo traen, entonces se van temprano, empiezan a ver a los pacientes temprano, y cuando ustedes terminen de hacer la reunión y todo se pueden venir, y ser mas independientes.

**I: ¿me puede ahora describir cómo es la relación con otras microrredes?**

En mi caso, que siento que las personas acá como más de zona rural, del campo, en otras microrredes son como más urbanizadas, verdad, entonces, es un poco más diferente, pero si tenemos buena coordinación con las demás Microred, porque nos reunimos varias veces, con los otros coordinadores, e igual si falta algún medicamento lo coordinamos, y ahí se plantean nuevas ideas, nuevas cosas, para mejorar, en el caso de nosotros que estábamos coordinados con los del SIBASI, como sabia como trabajábamos, porque yo, a otras microrredes tampoco iban todos los especializados, entonces sabían que nosotros en las reuniones de microrred repetíamos todo eso, entonces también se les programo de la misma manera, con un carro, entonces cada quien tenía como 14 establecimientos, a cargo de los equipos especializados, y eso 14 prácticamente cubrimos todo el territorio. Yo siento que a medida que ha ido pasando todo, ha ido todo mejorando, se han buscado nuevas estrategias, nuevas formas y aquí también se han buscado el SIAP, que es una forma de llevar un control más específico de los pacientes, por ejemplo ahorita estamos con las fichas nada más, pero luego vamos a tener los otros módulos de laboratorio y de farmacia, donde los pacientes van a ir a buscar sus medicamentos, y entonces cuando se integre todo va a ser mucho mejor porque ahora pacientes que están pasando consulta en Sánala por la mañana y luego por la tarde y vienen acá y si es posible por la noche van a FOSALUD, para más medicamento, entonces eso va a ayudar para controlar un poco, también tenemos una televisión local, para la prevención y generalmente siempre tocamos distintos temas, y en algunos casos temas de hipertensión, diabetes, insuficiencia renal; mire los días martes la radio y canal v, que es de la zona, entonces ellos nos dan el espacio de unos 5 minutos, para una noticia así rápida, programamos el tema, por ejemplo si está muy frio y viento o algo empiezan las enfermedades respiratorias, entonces dependiendo de la situación son diferentes temas.

Otra cosa que quería comentarle sobre la reunión de Microred que no le había dicho antes es que solicitamos que nos dieran un día entero para reunirnos, y nos lo aprobaron, luego yo estaba acá y yo no sabía mucho de contraloría al principio, entonces vinieron un grupo de señores, porque antes no se tenía el espacio, y no se podía porque se veía como que ellos iban en contra, y yo no sabía nada, entonces un día vienen y me dicen “mire doctor, fíjese que nosotros queremos hablar con usted”, y yo “ si como no” y empezaron a contarme que ellos querían ver cómo nos comunicamos, y yo “si”, “si porque no, además fíjese que hacemos un día al mes una reunión, y ¿Por qué no vienen?”, y ellos dijeron que “nosotros de verdad podemos estar ahí, que nosotros nunca nos dejan escuchar platicas” y “por qué” les digo yo, “por mí no hay ningún problema”, al principio algunos no querían que estuvieran, me recuerdo, porque me preguntaban qué porque los señores estaban ahí y yo les decía que los había invitado yo, entonces este, porque no van a venir si no tenemos ningún secreto, pero y después ya les fue gustando, al principio eran un poco fuertes con el personal, con los médicos, eran un poco fuerte, pero a medida que fue pasando el tiempo, ellos fueron conociendo el trabajo que salía de médico y ellos lo que había en la comunidad, y el personal de salud se quedaban asombrados porque no sabían todo el trabajo que hacían los señores en la comunidad, y les contaban todos los programas que hacían en la comunidad entonces ellos ya fueron conociendo el trabajo de los medicamentos. Estos señores eran de contraloría social, pero no estaban tan integrados como el Foro Nacional de Salud, eran de contraloría que ellos no habían aprendido mucho, y eran muy fuertes porque ellos no sabían. Y bueno al final acabamos llevándonos todos muy bien, y a mi considero que ellos me ayudaron bastante porque me decían “mire no tienen el medicamento tal” y yo les explicaba el porqué, y entonces ellos ya lo entendían; y bueno después yo siempre llevo a la reunión departamental, a miembros del foro, y de la comunidad, y que casi nadie lleva, y a mí me gusta, porque a veces cuando les voy a hablar, no es como que la comunidad diga oiga quiero esto y esto, sino que ellos ayudan a fortalecer; por ejemplo cuando lo del módulo del especializado, vinieron del FNS, y se solicitó que viniera también la ministra, y vino la Dr. XXX( del departamento de en crónicas), y fue muy efectiva esa reunión, muy muy buenas ideas que salieron, y se hizo el especializado, fue muy bueno juntar a todos; algunas del personal acá se preocuparon porque puede faltar tal y tal fármaco e igual me van a puntear, y yo dije no, déjenlos, y lo contario hicieron un programa para la gestión del fármaco, y después todos fuimos beneficiados con el proyecto del fármaco, un sistema mejor del medicamento, incluso me hicieron una farmacia nueva, y tengo la farmacia nueva aquí en la unidad, tiene 4 meses, en base de la contraloría social que nunca nadie pensó que eso iba a ayudar así, y beneficio al final a todo el departamento, no solo a nosotros, entonces cosas asi que la comunidad puede ayudar, asi que yo considero que si son muy buenos para eso, y poder mejorar, hay personas que tienen muy buenas ideas.

**I: ¿Como ves que tu equipo lo percibe, sigue habiendo resistencias?**

Al principio era difícil, muy difícil, yo no decía nada, pero sabia que ellos pensaban “ahí vienen otra vez” cuando venían los de contraloría, entonces hicimos una reunion, hicimos unas reuniones, porque el problema era que pensaban que era como ponerles el dedo, pero no era asi, entonces había algunas reuniones que eran muy fuertes, nos van a regañar pensaban, pero en estas reuniones que se hacían con el personal y los de contraloría social se les explicaba que era contraloría social, y como funcionaba, y no era poner el dedo, y ya algunos fueron entendiendo, ahora ya todos lo ven normal.

**I: ¿Y el buzón de sugerencias como lo ve?**

El buzón de nosotros si funciona, nosotros y los del FNS, y gente de la comunidad que son los que dan las charlas y ya tienen programado, cuando se va a abrir, entonces vienen y dan una charla y abren el buzón, luego nos venimos para acá, se traen todas las sugerencias que tienen allí en el buzón y se leen, se hace un acta, y se ve, entonces en la próxima vez que nos reunamos se ve si se ha resuelto el problema, que se hizo, y dependiendo de eso, pero si vienen bastantes de la comunidad.

**I: ¿Y usted cree que la gente tiene miedo de meter algo en el buzón?**

Al principio fíjese que sí, yo tenía un buzón que no era tan bonito como el de ahora, no estaba tan organizado, yo lo abría, y a veces un papelito ahí que no decía nada, a veces le echaban monedas, de esos centavos y yo veía unos centavos, pero hoy que ya pienso que, con las charlas, con la educación fíjese que la gente ha perdido el miedo. Ha mejorado mucho, el funcionamiento, porque hay veces que uno no puede estar todo el tiempo encima del personal, o a veces ellos hacen recomendaciones, por ejemplo, el odontólogo tiene mucho trabajo y sería necesario contratar a un ayudante, o alguna sugerencia de miren necesiten más personal ahí, no solo se fijan en cosas como no miren, me maltrato, yo estaba fijándome que ellos tienen buenas ideas.

**I: ¿Que consideras que se podría llegar a mejorar de la reforma?**

Bueno en el caso de nosotros yo pienso que lo de la reforma va como en evolución, yo pienso que como estaba a como esta hoy, ha mejorado enormemente, lo que pasa es que como va evolucionando paso a paso en base a las necesidades que se van presentando, por ejemplo, los sistemas en línea, el trabajo de red, es bastante fuerte, entonces en el caso de nosotros yo pienso que tal vez un poco de fortalecimiento en cuanto a cómo es la reforma, porque algunas veces si se está trabajando pero no se está conociendo mucho, porque incluso hay personas de acá que dicen que aquí en la unidad no hay de nada, pero nunca han venido, o vinieron hace 15 años entonces no se está dando a conocer, que tenemos especialistas, que tenemos laboratorio, que tenemos más medicinas en la farmacia, tenemos más cosas, están los sistemas en línea que cada día van evolucionando más, los datos, entonces todo eso no lo saben, incluso me encontré con un líder de la comunidad que fue alcalde hace muchos años, y le enseñe todos los datos que tenemos, y le dije yo puedo ver como está la gente, su salud, y él se quedó asombrado de que estuviera todo tan avanzado y ya que los médicos van a tener una computadora y todo eso, entonces, yo siento que la reforma está bien pero algunas veces lo que falta es promoción, pienso yo, que lo que más veces falta es promoción. Yo pienso también que, con todos los papeles, un montón de informes, un montón de cosas, pero como le digo hay que tener ilusión y ahora es necesario, pues con los sistemas de línea que se están creando ya no va a ser necesario. Lo de los papeles son lo que más se quejan, pero como va evolucionando pienso que con los sistemas en línea va a mejorar prácticamente todo, bueno yo lo que he conocido y lo que he visto como funcionamos es de la aplicación, porque muchas veces, por ejemplo, a veces están las cosas, pero no se conocer, entonces a veces la implicación es complicada.

**I: ¿De otras zonas del departamento que recomendaciones darías?**

Mas que todo la queja general, si usted va a cualquiera es la papelería, que se rellenan muchos documentos o muchos informes, pero como por ejemplo yo llevo un análisis de mortalidad así bien especifico, y otra gente no se lo toma enserio y solo contabilizaban lo que más le convenía, lo que no, no. Entonces es importante porque si no puedes ir a auditoria, como fue el caso, y se verifica el porque, entonces se hace un plan de mejora, para que no vuelva a ocurrir lo que ya paso, entonces se ve que va mejorando, yo siento que es de evolución y a veces es de aplicación, eso el lo que yo veo.

**I: ¿Otros problemas como falta de personal, falta de abastecimiento…?**

Vaya en el caso mío, no tengo ginecólogo, me viene a cubrir un ginecólogo de gotera, del establecimiento intermedio, que es de otra Microred, pero como trabajamos en red él puede venir porque a mí me falta ginecólogo, entonces nosotros lo que hacemos, es que el viene un día a tomar ultrasonidos de la Microred, y el siguiente día viene a ver pacientes en riesgo de la Microred, entonces eso nos ayuda un poco. El problema de nosotros es por la distancia que los especialistas no quieren venir tan lejos, entonces se intenta contratar a otros y no vienen, porque está muy lejos, porque ellos quieren estar más que todo en la ciudad, y en algunas ocasiones quizás el personal si puede ser un problema, por ejemplo también en la farmacia a mí me gustaría tener un farmacéutico, para que orientara a los pacientes, para que llevara a cabo cuantos medicamentos hay, y que pueden decir que medicamentos faltan, yo tengo una encargada de farmacia que es una enfermera, que no es lo mismo que si fuera un farmacéutico. Son detalles que van quedando, pero como ya le decía son procesos de evolución, incluso ahorita los centros están bien equipados.

**I: ¿Y el tema del desabastecimiento?**

Fíjese que gracias a Dios no, el trabajo en red ayuda bastante, incluso ahora aparecíamos como la más abastecida del departamento, entonces se hace algunas gestiones y coordinaciones, y en base a eso incluso en algunas ocasiones coordinamos con el hospital, para que ellos nos puedan transferir algunos medicamentos.

**I: ¿Y el laboratorio?**

El laboratorio lo estamos coordinando de esta manera, por ejemplo, el laboratorio de nosotros, gracias a dios, tenemos un licenciado de laboratorio, y ellos tienen alguna cantidad de exámenes básicos que hacemos aquí, pero los días lunes hacemos unos especiales para pacientes que están citados y tomamos los exámenes y lo llevamos a un laboratorio más grande, entonces ya después tenemos los resultados, y si son de emergencia aquí también se le pueden tomar muestra. En tema de insumos pues como todo hay veces que hay más cosas y otras menos, a mí me gustaría tener de todo, pero no se puede, peor igualmente cuando falta de algo aquí se toma la muestra y lo llevamos al regional. Por ejemplo, una vez paso que teníamos mucho Chagas, entonces a los pacientes que me mandaban IgG, y no teníamos Ag especifico, entonces detectábamos muchos casos, o con el hellicobacter pylori, yo siento que muchos pacientes pueden tener eso y no saben cómo es el tratamiento entonces la posibilidad de las muestras nos ha permitido llevar algo más.

## Staff interview 3: Intermunicipal coordinador

Entrevista con: Coordinadora intermunicipal

Fecha: 13/06/18

Lugar de la entrevista: Unidad Comunitaria de Salud Familiar La Palma

Código del informante: SFNV006
Genero del informante: F

Nombre del entrevistador: NV

Sección 1: Perfil del participante y función en la prestación del servicio del primer nivel de atención

NV: ¿Información sobre su perfil, que es el rol que usted tiene?

I: Mi nombre es xxxx, soy enfermera coordinadora de la micro red. Nuestro trabajo es la red. Eso quiere decir que tenemos ecos adjuntos a este establecimiento, en total son 9. Entonces yo soy enfermera coordinadora de todos esos ecos. Entonces llevamos la función administrativa y parte técnica.

NV: ¿Cuanto tiempo ha trabajado en esta unidad?

Informante: En esta unidad llevo en total desde que yo empecé, 26 años. En enero voy a cumplir 27 años. Y mi trabajo ha sido siempre en esta misma red.

NV: ¿Entro directamente a esta red?

I: Si, yo soy de acá de La Palma. Entonces mi servicio social lo hice en Los Planes. Cantón Los Planes que es parte de la zona alta. Luego, después que, hecho mi servicio social en la zona alta, me pasaron para La Palma. Luego de la Palma, en el 2000 me fui para San Ignacio porque allí se necesitaba un recurso que fuera a cubrir, parte de la micro red. Pero ya a partir del 2010 he estado permanente acá.

NV: ¿Me puede contar sobre su función actual en el primer nivel de atención?

I: Si, va en caminado a la atención preventiva y curativa. Y este ver el funcionamiento que el profesional de enfermería brinde las atenciones con calidad y calidez a los usuarios. Ese es mi rol. Velar por qué los procesos de enfermería se cumplan según lineamentos establecidos. Y de igual manera se hacen monitoreos a las diferentes áreas para ver que todo esté en regla como decimos. Que el paciente se atendido como se pide, como debe de ser.

NV: ¿Y esos monitoreos, de que se tratan?

I: Son monitoreos de como unas listas de chequeo. Como en donde yo voy al área de vacuna, un ejemplo, voy al área de vacuna, verifico que la cadena del frio, de la refrigeradora este bien. Para las vacunas. Y verdad que se cuente con todas las vacunas que están ofertas, que este completa. Porque si no hay, que pasa, porque estamos desabastecidos. Vemos si algún otro lugar tiene existencia y que nos transfiera.

NV: Lo que me cuenta del monitoreo, eso es en asegurarse en cosas como de vacuna, de medicamento, ¿funcionamiento de la unidad? ¿Y no tanto para el monitoreo del paciente todavía?

I: También. Va aparte. Para ver lo, técnicamente, verdad, igual. Sigo con el ejemplo de paso de vacuna. Que el recurso que está aplicando la vacuna sea con la técnica correcta, las medidas de asepsia correctas, la vacuna correcta.

Sección 2: Comprender las trayectorias de los pacientes en el primer nivel de atención

NV: Esa parte es para saber un poco sobre su role, su perfil. Esta investigación se trata de enfermedades crónicas, ¿no? Transmisibles ¿no? Entonces, lo que queremos hacer es entender un poco sobre los pacientes, bueno. También sobre su role con los pacientes. Pero para tener una introducción. Para entender un poco, de cómo se presentan aquí los pacientes para entender las trayectorias. Les pregunto algunas preguntas en ese tema, ¿no?

Para comenzar, me puede describir su participación con pacientes, con enfermedad crónica aquí en esta unidad, o si no, ¿en su trabajo?

I: De entrar, verdad cuando brindamos de charlas educativas sobre enfermedades no transmisibles: hipertensión arterial, diabetes, estilos de vida saludable, como promocionar los estilos de vida saludable. Eso es, por una parte. Charlas. Consejerías. Estar aquí sentado con el paciente. Tenemos unos consultorios adonde podemos brindar unas consultorías más personalizadas. Que sea que el paciente tenga la enfermedad transmisible o que no tenga la enfermedad transmisible. Que sea para prevención o para estabilizar. Eso es una. Como preventivo. Y también, nosotros, como enfermería, hacemos procesos sobre atención de enfermería. P.A.D.E.S.- Procesos de Atención de Enfermería. Y también va en el ámbito educativo. Y en el caso de las auxiliares, las compañeras de blanco, también so auxiliares. Entonces ellas también tienen su parte brindando consejerías. Las llamamos protocolos de atención de enfermería. Ellas y en caso de nosotros, procesos de atención que llevan más con un plan de cuidados.

NV: El Proceso es el plan de cuidados y el protocolo. Es más bien como consejería educativa.

¿Y en el primer nivel de atención en términos de actividades que cumplen en las diferentes etapas de su proceso de buscada de atención? Por ejemplo, en la recepción o registro de pacientes.

I: A la entrada, todo paciente que solicita atención en esta unidad comunitaria de salud familiar, se toma sus signos vitales. Sea hace de entrada, un traje. El Medico es el encargado del traje. La señorita enfermera les dan también, y en ocasiones también el médico, brindan la orientación a los situaros sobre cuál es el proceso para la atención. Que va a pasar primero por un traje, por qué va hacer el médico, quien va valorar, la urgencia con el que va pasar usando los colores: el rojo, el verde, y el amarillo. El rojo va a la emergencia que es en otro lugar. Si es el amarillo, puede esperar aquí dentro del establecimiento entre una y dos horas. Y el verde puede esperar otro tiempo prudencial o lo ofertamos una cita, en el agendamiento. Para que el usuario que viene el siguiente día, archivo ya le tiene sus expedientes. Entonces, el Medico valora en ese momento si es rojo, amarillo o verde. Y dependiendo de eso, pasa a la consulta. Luego, la enfermera empieza a tomar señales vitales, a tomar temperatura, a pesar, pallar, y algo bien importante, toma de presión arterial. Allí, empezamos muchas veces a detectar pacientes con hipertensiones altas.

NV: ¿Y no se cambia el color en ese momento?

I: Como no. Pero como también el Medico lo hace en el traje, también él. Pero como de un rato a otro, puede variar, lo hace el medico en el traje, y luego ya la enfermera, otra vez, en lo que archivo, está sacando los expedientes.

NV: Porque, ¿de repente de vez en cuando se pone más alto cuando esta con el medico?

I: Así es. Y como trabajamos en rede, entonces vienen de otros ecos, de otras unidades comunitarias de salud familiar, de la red, los pacientes vienen con referencia (le llamamos una hoja de consulta) para que sean evaluados por un médico familiar.

Las unidades comunitarias de salud familiares a inscrita a esta UCSF, consulto con su equipo, le dan seguimiento a la despenalización. La despenalización es que todo paciente necesita ser evaluado integralmente. Y, si tiene una enfermedad crónica, nosotros [¿?] Esta enfermedad transmisible, y depende el médico, lo puede estar evaluando, o cada 4 meses, o si está estable, cada 6 meses. Esas son las enfermedades no-transmisibles. A el seguimiento de la despenalización, igual el médico de familia si ¿? Tiene que evaluarlos por lo menos una vez al año si está estable. Y si esta descompensado, el doctor del médico de familia, lo dejan, y él sabe hasta cuándo le van a dar el mal. Hasta que lo tiene estable.

NV: Cuales son sus principales desafíos que enfrenta a cumplir su función

I: Disminuir las enfermedades no-transmisibles en alto porcentaje, y las que ya están, que se mantengan estables con su medicamento, con estilo de vida saludable, el hecho de brindarle la consejería: no alimentación con grasa, condimentada con sal, no bebidas carbonizadas, no todo lo que le va hacer daño a su organismo.

NV: ¿Hay una etapa que piensa usted que sea más importante enfocarse, la prevención, la diagnostica, la detención, seguimiento, ósea para desafíos?

I: Si. Quizás la prevención. Porque el que ya está, el que ya tiene su enfermedad no-transmisible es mantenerlo con sus medicamentos, su consejería. Pero el que no, uno no sabe cuál enfermedad le puede llegar. Entonces, el principal desafío es la prevención. La alimentación saludable, ejercicio 30 minutos todos los días, el consumo de agua segura, 8 vasos de agua diarios. Dormir lo suficiente. Recrearse, todo eso es importante. Uno con las consejerías, el proceso de atención de enfermería, los protocolos, las charlas. Todo eso va encaminar. Y tenemos aún a vuestra estrategia, ejercicio. Ejercicio es medicina. Entonces allí también, hay gente que se les están llenando aun como su pasaporte, le decimos. En donde él se compromete, o por lo menos, ¿si come comida chaparra, cual es el compromiso del acuerdo? Disminuir un poquito. Entonces, también importante que, a esta gente, es incorporándolos a grupos de autoayuda. Entonces eso hace que el usuario del paciente se llene de información, y si ve que el otro lo hace, el otro lo hace, yo también lo puedo hacer. Entonces en eso están los clubs de adultos mayores, y los grupos de auto ayuda. Ahora estamos trabajo nada más con uno, en el Canto [¿Mao de Huacatay?] que queda en la zona rural.

NV: ¿Como respondan los pacientes con los mensajes? ¿Los siguen?

I: Si. Cuesta un poco. Pero si hay gente que dicen sí, yo necesito y lo puedo hacer. Lo voy a hacer. Entonces todo se lo va monitoreando. En el caso de la obesidad, digamos, se le va monitoreando el peso, y van viendo que hay resultados, contentos, y siguen.

NV: ¿Con la obesidad, so casos que los pacientes piensan que ya necesitan ahora mismo? O de repente porque en el futuro se van a poner un poco mal y lo tengo que hacer ahora. ¿Hay urgencia en esto con los pacientes?

I: Es de una forma gradual. Pero sim si uno les dice, hay que hacer lo ya, comenzar, ya, ¿? que es paso a paso. Es un proceso. Igual, yo mamo, les enseño a mis hijos, a mi esposo, a la comadre, a la vecina, para ir en esas secuencias.

NV: ¿En caso de desafíos de pacientes que no quieren responder, tienen alguna estrategia para poderlo manejar?

I: Justo allí, encaminamos a los procesos de atención de enfermería. Nosotros hacemos un plan de cuidado. Por ejemplo, un hipotenso. Yo les siento, hacemos un plan de salud juntos. A que se compromete. Tiene su salero en la mesa. Sabe que la sal es buena, pero sabe que la sal le va hacer mal. Porque yo le he explicado todo lo daño que le va hacer el comer sal. Entonces le dice que sabe que no, no tiene que comer sal. ¿Usted cree que podemos retirar el salero de la mesa? ¿Sí? Entonces ese es un acuerdo, ¿verdad? Y lo hace. Entonces yo le doy el seguimiento. La idea es que no se me descompense lo que tiene la enfermedad, pero lo va a mantener estable. Esos son los procesos de atención de enfermería adonde la atención es más personalizada. Con esos pacientes descompensados. No esos pacientes que vienes con el azúcar a 600 a 700 y que toca bajarlos al hospital de segundo nivel. A ellos no podemos hacer nada. Ellos ya se van. Cuando regresan, atravesó de una referencia, se le notifica al eco para darles su seguimiento.

Sección 3: Trayectorias de cuidado de personas con enfermedad crónica

NV: Podría describir los pasos de enfermería que usted toma con un paciente de enfermedad crónica que entra a la unidad de salud.

I: Hay dos días. Uno, o es de emergencia y esta descompensado, o el compensado. El compensado tiene una cita de seguimiento. ¿Por qué? Por que como es unidad comunitaria de salud familiar especializada, quiere decir que tenemos aquí el médico de familia, tenemos la nutricionista, tenemos el psicólogo, tenemos laboratorio, tenemos ontología, entonces ellos ya vienes con su cita previa. El médico de familia lo evalúa o cada mes o cada dos meses, cada tres meses, o el medico lo ve en los acercamientos, porque el médico de familia también va, a san Ramon, a la zona alta, hacer la buscada de esos pacientes en eso. Entonces si el paciente viene compensado, el médico, igual. Pasa por la enfermera, lo prepara

NV: ¿Usted o cual quiera otra enfermera?

I: Yo. Las auxiliantes son mis compañeras. Yo veo que ellas, esa forma de atención sea constante.

NV: ¿Cuándo los pacientes vienen con sus compañeras, usted tiene un rol allí?

I: Muchas veces sí. Porque a veces vienen referidas De hablar de pacientes descompensados que vía teléfono informa que va a venir un paciente, muchas veces acompañado por el personal de salud. Entonces ya vienen con su referencia entonces esa atención, si es directa tienen el área de emergencia allí, alii si me desplace para ver lo que se necesita en el momento. Para apoyar al médico y si es necesario, ser trasladado al hospital del segundo o tercer nivel, se llega.

NV: ¿Hay algunos casos de pacientes que tienen su cita, pero no llegan? ¿Usted tiene un rol en esos casos?

I: Por lo general, en la expedienta, se inscribe de que el paciente no ha ¿? y se informa al eco correspondiente. Lo retoma, saca cita nuevamente y se le vuelve a dar la cita.

NV: ¿Cuéntame por favor a cerca de su experiencia brindando apoyo a pacientes crónicos, de repente con un ejemplo reciente como usted realizo en el seguimiento del paciente de las enfermedades no transmisibles? Me dio el ejemplo de la educación sobre la sal. ¿Tiene usted otro ejemplo?

I: Si, por lo general, siempre cuando vienen descompensadas con la azúcar alta. Los pacientes andan ansiosos, que quieren pasar ya, y andan preocupados por su misma enfermedad. Entonces, el medico la manda para emergencia, para colocarles su catéter, hidratarlos, ponerles su dosis de insulina, para estabilizarlos. Entonces hablamos con el paciente sobre el estilo de vida saludable, sus medicamentos, si se los toman a la hora. Eso más que todo. Y no se le delata hasta que ya este compensado. Eso es constante. Es un ejemplo general.

NV: ¿Hay un ejemplo que le ha pasado a usted recientemente con un paciente que necesita apoyo? ¿Con manejar su enfermedad crónica o si viene a pedir asistencia?

I: Si, tuve la experiencia con un caso con una persona adulta mayor, femenina. Ella siempre visita esta unidad comunitaria de un correspondiente a otro eco. Pero ella le gusta venir acá a la palma por que le gusta la atención que se le brinda. Prefiere La Palma. Entonces viene con la azúcar alta. Venía con la azúcar a 700. Entonces entro, estaba desesperada, entonces la llame, me senté con ella en un cubículo. Empecé a hablar y la pase de inmediato con el doctor. Y el doctor le dejo su insulina. Nada más necesitaba matricular y para mantenerla tranquila, la llevé, la acosté, estuvimos hablando, y se tranquilizó.

NV: ¿Aquí?

I: Todo aquí. Ya con referencias a el hospital por el momento no. Aquí se las estabilizan bastantes pacientes. Se fue tranquila, quisiera tomar su medicamento todos los días a la hora, su dieta.

NV: ¿Entiendo lo de la diabetes, pero por que se le subió la azúcar?

I: No había tomado su medicamento. Entonces, allí la orientaba a la consejería.

NV: ¿Ella le dijo alguna razón por que no lo tomo?

I: Se le olvido. Y ella vive sola. La mayoría de casos de adultos mayores, porque no tienen a alguien cercano a ellos. No pueden leer, se los olvida. No están al cuidado de otra persona. Eso es el mayor problema, por olvido. O si vienen, y vienen con otra patología, les dan varios medicamentos, entonces ellos llevan toda la medicina y no saben cuál.

Mi mama es diabética e hipertensa. Se cuida muy bien. Ella sabe lo que tiene que hacer, su medicamento, en sus cajitas: ese a la hora, ese a otra hora, ese a otra hora. Así separados. Y en el hospital de segundo nivel, la ve el cardiólogo, la ve el endocrinólogo porque tiene antecedente de tiroides. Y la ve el neurólogo. Ella no pierde sus citas.

NV: ¿Es San Miguel?

I: No, Santa Tecla.

NV: ¿Es parte de la microrred?

I: No. De nuestra microrred no. Pero es parte de la red de los servicios de salud.

NV: Ahora, Que tan potable es brindan un mejor soporte a los pacientes que lo necesitan, de repente sobre aspectos prácticos de habilidad o de repente sobre sustentabilidad. Podría ser relacionados al ejemplo que me dio de la señora con la azúcar alta.

I: Si hay muchos aspectos en que se les ayuda. Siempre garantizarles sus medicamentos. Siempre. Si es cual quiera patología, garantizarles los medicamentos cuando el venga se va a tener el estabiliza miento. Si viene descompensado y de allí es necesario trasladarlo a el hospital, se lo lleva la ambulancia con personal médico o de enfermería si es necesario y la atención especializada. Eso es importante porque, si ando ansioso y tengo el psicólogo, el psicólogo me puede dar una atención.

NV: ¿Y aquí hay psicólogo en esta unidad también?

I: Si.

NV: ¿Puede pensar en un problema que presenta frecuentemente los pacientes y que realizan su seguimiento con usted?

I: ¿Las patologías?

NV:

I: La obesidad. Si entran por los estilos de vida saludables, la mal alimentación, no consumo de agua, no hacer ejercicio, se palea frecuente.

NV: ¿no consumo de agua? ¿Y eso es por qué?

I: No les gusta. El habito no está. Los pacientes no tienen la costumbre

NV: ¿Y la obesidad es por…?

I: La dieta. No tener dolor de cabeza por la misma deshidratación.

NV: ¿Noto alguna diferencia entre estos y sus otros pacientes que tienen diferentes conocimientos de sus enfermedades? ¿Hay diferencias entre pacientes que quizás sean más conscientes que los otros?

I: Si. El nivel cultural. No vamos a comparar una población que vive en un área rural a una población que vive a un área urbana. Igual, no vamos a comparar una población que vive en un área urbana que es profesional y otra que no es profesional. uno que es profesional le va a entender. Lo va a comprender. Le va a decir la información a alguien que no puede leer. Es diferente. Entonces el factor cultural es una de las barreras más grandes para que al paciente le pueda dar seguimiento a sus tratamientos. Para que le pueda comprender una dieta, que tienes que tomar un medicamento todos los días, a la misma hora, para que no se le descompense. Y muchas veces el factor económico porque necesita una dieta saludable y balanceada.

NV: Eso cuesta más, ¿no?

I: Cuesta, tal vez por el hecho de desconocimiento. Hay va enfocar nuestras prioridades de atención, ¿verdad? Nuestras prioridades de consejería. Que tiene hierbas, que consume con las hierbas. Que tiene huevos. ¿Que consume con los huevos?

NV: Entonces ustedes les dan la consulta cuando ven que no…

I: La nutricionista. Tenemos nutricionista

NV: Así que, si hay nutricionista, psicólogo…

I: Es integral

NV: Para entender la trayectoria, el paciente viene, no tiene mucho conocimiento sobre la obesidad o la nutrición. ¿Este paciente les manda a diferentes consultas en la misma unidad?

I: Si. Por eso, el ECO le manda acá con su referencia. Entonces el médico de familia que tiene el ¿? De treinta, verdad, le manda con un nutricionista. Le ve carias con el odontólogo. Le de trastornos de comportamiento con el psicólogo a manera que sea integral, y prevenirlo todo

NV: Si este paciente viene y necesita odontólogo, psicólogo, o uno que viene con todo, ¿tiene diferentes citas en diferentes días o lo hace todo el mismo día?

I: Se trata de pasare el mismo día, pero se hace difícil. Por lo general son citas en diferentes días

NV: Y en los pacientes que se nota que hay diferencias culturales en el entendimiento, ¿que estrategias hay? ¿Por ejemplo, hay campañas?

I: Si, visita de domicilio por parte del promotor de salud o el equipo

Sección 4: Estrategias en el manejo de pacientes crónico

NV: Que tipos de desafíos surgen en el manejo del paciente crónico, enfocándonos en problemas sobre desafíos de comunicación

I: Tal vez, de traslado al hospital de segundo nivel por el hecho de la distancia

NV: ¿El paciente tiene mucha distancia para llegar a el hospital?

I: Sí. Por qué este tiempo se va 50 minutos para poder llegar al hospital de segundo nivel.

NV: ¿Como se va en ese caso?

I: Ambulancia

NV: ¿Cuantas ambulancias tiene esta unidad?

**I:** Ahorita tiene 3. Y un vehículo que es adonde los especialistas se trasladan a 2x de la micro red.

NV: ¿Las ambulancias funcionan bien? ¿Hay veces que hay problemas con las ambulancias o las carreteras o que se caen las piedras a la carretera?

I: Con las ambulancias, ahora no. Y con las carreteras sí, pero no es frecuente.
NV: Así que las ambulancias pueden llegar

I: Si. Llegan.

NV: Con respeto a las diferencias culturales, usted ha notado diferencia en el uso de idioma técnico. ¿Usted nota que lo entiende o hay dificultad?

I: Si. Muchas veces cuando no me comprenden la información, yo le notificó a la [promotora de salud, y la promotora va a su casa y le explica con su idioma. Frecuentemente las promotoras de salud por lo general son de la misma comunidad así que hablan bien y se entienden. no con palabras que no van a comprender.

NV: ¿No es el mismo idioma?

I: Si, el mismo idioma, solamente diferentes palabras.

NV: Y se comunican mejor porque son del mismo sitio

I: Sí.

NV: ¿Pero hay situaciones aun que en las unidades saludables no entienden mejor con la promotora?

I: Por lo general no. Es muy raro. No es frecuente

NV: La pregunta es porque en algunos sitios de repente hay choque de culturas. ¿Entonces, durante la comunicación entre persona y paciente hay algún caso que usted ha notado que se pierde la traducción? O que le explica algo y el paciente dice si, si, sí, pero usted se ha dado cuenta que el paciente no ha entendido nada.

I: No, no no.

## Seccion 6: cambios a lo largo del tiempo

## NV: ¿Ha notado cambios en los últimos 10 años?

## I: Por supuesto que sí, se han aumentado el número d unidades de salud y es un avance que ahora los especialistas se trasladen a las comunidades.

## NV: Esto sería todo entonces, muchas gracias

## Staff interview 4: Regional Director of Health

Entrevista con: Director Regional de salud

Fecha: 13/06/18
Lugar de la entrevista: Hotel San Salvador
Código del informante: SMMJ06
Genero del informante: M
Nombre del entrevistador: MJ

- Bueno, lo primero de todo agradecerle accede a esta entrevista. Lo primero que nos gustaría preguntarle es cuál es su función en el primer nivel de atención.
- Bueno, mi cargo en el ministerio de salud, es el de **director regional,** es decir ejerzo la función de la ministra en el oriente del país, que comprende **1 millón y medio de habitantes, 4 departamentos (Usulután, Morazán, San Miguel y La unión), corresponde a casi un tercio geográficamente hablando, del país y que cuenta con 292 establecimientos de salud apertura dos. Tenemos, al igual que en la mayoría del país, 60% intervenciones con ECOs y el 40% no tenemos intervención** con ECOs, así que el departamento oriental del país no es porque yo sea de allá, pero del Lempa para allá somos diferentes, históricamente, social, económica y políticamente somos diferentes al resto del país, y nos comportamos de manera diferente, así las áreas también son muy diversas, tenemos playas, montañas, terremotos, tenemos islas donde tenemos servicios comunitarios, somos la única zona del país donde tenemos **ECOs en islas**., somos el único, y esa diversidad nos hace ser un poquito etnocéntricos (risas) y hacemos la función gerencial de la zona oriental del país.
- Entonces, ¿usted formaría parte del SIBASI?
- No, yo estoy arriba del sibasi, yo conduzco los 4 sibasis.
- ¿Cuáles son las principales barreras que se encuentra usted en la coordinación o frente sobre todo al abordaje de las enfermedades crónicas, a la hora de la coordinación con las microrredes y teniendo en cuenta el gran peso que tienen estas enfermedades ahora mismo?
- Bueno, quizás es una pregunta que por mucho tiempo no hemos hecho, y en sí es una pregunta en sí, bastante estructural. Nosotros tenemos ahora 115 equipos especializados, los equipos especializados como ustedes saben tiene, aparte de bagaje de médico, enfermera, farmacia, polivalente y laboratorio, y otros para el tema de enfermedades crónicas tiene asignados internistas, ginecólogos, pediatra, para esto, pero que pasa, nosotros tenemos las plazas para asignar estos recursos, lo que pasa es que los recursos **no se quieren ir a trabajar lejos**, tenemos cerca de 7 plazas de especialistas libres para cualquier especialista de estas ramas, pero no se quieren ir, porque? Porque el modelo desde el punto de vista educativo **desde la formación no se le forma a los estudiantes sobre la importancia de la comunitaria,** de hacer llegar, como nosotros decimos “Acercar la salud a la población” y que llegue un médico internista o un MGI como le dicen en Cuba, o un médico de familia a una comunidad alejada y que sepa la importancia que esto tiene que en sí es la esencia de nosotros como profesionales de la salud llevarla “Hasta los más pobres de los pobres”. Lastimosamente esta conciencia, dentro de la formación, tanto básica como especializada, no le dan la suficiente importancia. Posiblemente las buenas universidades como la Nacional, mete la atención comunitaria como una materia por 5 años, algunas otras las ven por un año o dos años, se ve epidemiologia y todo pero poco práctica, ya luego cuando van a las unidades formadoras que sacan su Carrera y sacan su especialidad que se tardan 3 o 4 años en formar a un especialista no le inculcan el verdadero valor que tiene el hacer trabajo comunitario, y el salario que le ofrecemos a un médico en la ciudad es el mismo que le ofrecemos en el campo, con las mismas prestaciones, misma estabilidad 8 horas, de lunes a Viernes, sin turnos, que otros hacen guardias como les llaman ustedes, de fines de semana y así, aquí no, es más se les estimula a muchos de ellos llevándoles a congresos o dándoles un plus como por ejemplo cursos de ultrasonografía que son pagados por el Estado, pero a **las nuevas generaciones no les gusta ir al terreno.** Entonces, no tenemos Muchos médicos internistas y muchos de ellos están renunciando porque con esta apertura de establecimientos más cercanos, así como las grandes empresas se come a las chiquitas, los grandes prestadores jalan de los médicos a las ciudades. Por ejemplo, hoy en San Miguel se va a construir el hospital regional del Seguro social, es un mega obra de años y van a necesitar muchos especialistas y ahí, aunque ganen menos en el Seguro prefieren estar ahí cerca en la urbe que estar en el equipo comunitario. Yo admiro mucho, y **me quito el sombrero por los compañeros que han permanecido y les gusta ese trabajo,** que están el todo por el todo, no les importa que un día vayan a ver a un cantón 5 – 10 personas, adultos mayores, diabéticos o pacientes crónicos en general, que le van a dar un buen seguimiento un buen control en las unidades y que les gusta su trabajo, algunos renuncian porque llevan 5 o 7 años y quieren estar más cerca. Por eso, es una de las grandes dificultades que tenemos la falta de personal que quiere adherirse a este esfuerzo que desde el gobierno central se está haciendo para brindar de Equipos Comunitarios Especializados. En plan de retos como les comentaba, nosotros tenemos las plazas disponibles, las 7, si alguien llama se le dice, sí, empieza a partir de mañana, pero nadie quiere, es más tenemos unidades formadoras en San Miguel, en el hospital San Juan de Dios de ginecólogos pediatras internistas. Pero no quieren ir al trabajo comunitario. **Posiblemente si se les creara más conciencia social más entrega o fueran más humanitarios, posiblemente sí ampliaríamos más nuestra cobertura. Pero lastimosamente la gran mayoría no se quiere ir, Nada** menos este año se presentaron a concurso 4 ginecólogos, y cuando se les dijo que eran en zonas Rurales (parquin, Corinto.) renunciaron, frente a eso no se les puede obligar, mucho queremos quererlo, pero no se puede. Como les comento el salario es el mismo que en la ciudad de lunes a viernes y más estable, pero como les comento, algunos prefieren irse a la práctica privada y pueden llegar a un nivel adquisitivo adecuado, pero no ayudando humanitariamente a quien más lo necesita. Ese es una de las barreras.

La otra es, que va digamos concatenado con esto, es el **problema de los medicamentos**. Nuestro cuadro básico de medicamentos tiene una clasificación entonces a ciertos medicamentos de prescripción por especialista que en las unidades intermedias no pueden utilizar, por ejemplo carecillo o medicamentos cardiacos o de segunda o tercera generación de antihipertensivos irbesrsartan no puede ser prescrito más que por un médico especialista, entonces ya luego puede ir haciéndosela repetidamente, pero el primero que se lo prescriba tiene que ser un especialista, también para que dese el punto de vista legal se confirme que esa persona sí lo amerita. Los medicamentos sí los tenemos, pero tienen que ser prescritos por un especialista que, como les digo, no suele estar en la zona. Un médico general con muchos años de experiencia tiene la misma capacidad, pero por lineamiento tiene que ser un especialista el que indique a menos por primera vez el medicamento. A veces con las insulinas también se dan gratuitamente los servicios, pero tiene que ser un especialista el que los prescriba y a veces, hay que decirlo, **a veces el complot de la industria farmacéutica nos deja sin abastecimiento, no es culpa del gobierno**, porque el Gobierno tiene el presupuesto para desembolsarlo con ellas, o comprárselo, pero ellas a veces dicen “no podemos, no tenemos la materia prima, no hace falta”, entonces generan desabastecimiento en esos medicamentos esenciales. Por ejemplo, tenemos pacientes hipertensos que la enalapril no les funciona o les da tos o lo que sea, entonces tenemos que cambiarlo por otro losartan o irbersartán, pero posiblemente la industria farmacéutica dice que no tienen, aunque tengamos el dinero desde el gobierno dicen que no tienen. O cuando se hacen las licitaciones públicas, como les menciono es un complot, y dicen no tenemos, pero cuantas se han ido del país, solo la Pfizer, y el resto se han quedado. Y tenemos farmacéuticas nacionales que les pueden elaborar, pero lastimosamente se meten en el mismo juego de estar en contra de la reforma y eso hace que nos veamos mal, desabastecidos, como que no estamos hacienda bien las cosas, ¡de ahí la gente de derechas es donde se agarra y aferra para decir que en los establecimientos no hay medicamentos y tal vez sea uno! Del cuadro de 55 medicamentos básicos solo uno Podemos estar desabastecidos, pero ahí es donde la gente de derechas dice están desabastecidos. Y en alguna ocasión hace como 4 años, parece inédito increíble, el Seguro social no tenía aspirina para los cardiacos, un medicamento que tú vas a la esquina y lo encuentras, pero las empresas ninguna tenía aspirinas para poder venderle al Seguro social por las cuestiones legales por la ley *LAPCA* tenemos que hacer un tipo de formulario, presentarles y quien gana muchas veces nadie concursa porque no va a ser siempre para nosotros, como les menciono también la barrera política económica, a nosotros nos limita el presupuesto, queremos que se nos dé más anualmente pero siempre hay recortes presupuestarios, por eso cualquiera puede ser una empresa diría que estamos en banca rota. Porque nosotros desde el ministerio cerca del 70% es en salario, y el 20% para medicamentos, que es mínimo si tú lo compruebas, entonces anualmente el mismo gobierno no Podemos aumentar el presupuesto porque se nos resta, no podemos caer en una ilegalidad con los trabajadores reduciendo el salario porque ahí sí que se nos cae el mundo encima, pero para infraestructuras por ejemplo, no tenemos capacidad de poder construir un edificio, un ECO, porque no tenemos presupuesto para eso, tenemos para reparar, tapar hoyitos, pero nuevos, difícilmente, y por eso lo que más se sufre es en la parte de medicamentos que no se da presupuesto, nosotros querríamos tener más medicamentos dentro del cuadro básico para que estén al alcance de la población, pero como le comento por los presupuestos no puede ser, y es que en algunos casos es por cuestiones burocráticas digamos, por digamos acá en el Bajo Lempa por ejemplo, con todos los enfermos renales que tenemos, allá estamos llevando lo que se llama **“Unidad de Gestión Clínica renal”** para asegurarles que tengan su tratamiento en sus casos, pero que pasa que en muchos casos esos pacientes requieren eritropoyetina y nosotros desde el primer nivel no Podemos dispensarlo porque no tenemos códigos para ello, tendrían que crearnos ese Código para poder asegurarse la dispensación a los pacientes renales, eso sí es una barrera bastante superable, pero por ejemplo nosotros en el primer nivel tenemos un presupuesto, y los hospitales tienen otro. La eritropoyetina recombinante humana está dentro de su presupuesto y des sus posibilidades económicas, nosotros no tendríamos la capacidad económica para comprar la eritropoyetina tampoco al menos que nos pasaran parte de su presupuesto para poder dispensarla desde el primer nivel., pero nosotros poder comprar este medicamento no Podemos. Y algunas cosas que se han conseguido es por ejemplo en los ECOS tenemos **electrocardiogramas**, ustedes supongo que habrá visto, tenemos e**quipos de ultra** para las embarazadas. EN medicamentos estamos peor, pero en la parte de **exámenes de diagnósticos**, en estos años hemos conseguidos Buenos objetivos en cuanto a reactivos para enfermedades crónicas, por ejemplo, la hemoglobina glicosilada que es un reactivo muy útil para el diagnóstico del paciente diabético más fiable que la glicemia central. Lo que sí que nos ha faltado es que son equipos para el laboratorio regional centralizado que al ser el oriente tan grande queda lejos cerca de 3 horas del laboratorio regional, el laboratorio regional analiza la muestra, pero el problema es el transporte si tenemos un circuito de toma de muestras, pero es insuficiente. Entonces como les digo hay que asegurarse que una muestra no se extravíe ni coagula, es difícil, sí que se intenta hacérselo a todas las personas que lo ameritan, pero el problema es cómo hacerlo llegar al laboratorio regional. Sí nos han dotado de motos, pero quien las maneja esas motos, no tenemos presupuesto, aunque tuviéramos para gasolina no hay para todo. Algunos han creado sus redes de traslado en motos, pero otros lo transportan en vehículos entonces no se llega a todos sitios, es una dificultad. También hace dos años como le comenté se creó la Unidad de gestión clínica renal que se está haciendo un tamizaje de pacientes con ciertos criterios, si son hipertensos, diabéticos, embarazadas, niños con ictus de repetición y por supuesto lo pacientes renales entran, se ha hecho uso de todo lo que nos ha dado para hacer tamizajes, eso ha sido un gran apoyo para nosotros como región que es una de las más afectadas por la enfermedad crónica. Algo que sí conseguimos fue el tema de los insumos, También con los enfermos renales se han hecho un convenio regional del cual los pacientes se están haciendo DPSA (diálisis peritoneal ambulatoria), el hospital ha hecho que la empresa les lleve a los pacientes el material necesario hasta su casa, esto nos ayuda mucho a que la sobrevida haya aumentado, la mortalidad siempre ha sido la primera en el país, pero Bueno estamos viendo aun resultados a corto plazo, en el oriente es donde más se ha diagnosticado, ahora se detectan con estadio 3b y 4 antes se morían no más por gracia de Dios, ahora se les explica se les ofrece tratamiento como la diálisis, unos dicen que sí otros que no, se la posibilidad de la diálisis peritoneal qué materiales necesitan, todo. Y el otro tema que hemos intentado trabajar un poco es el de **los pacientes convulsivos** I que hemos intentado alejarlos un poco de los psiquiátricos y tenemos neurólogos, pero pasa lo mismo no quieren ir a trabajar al interior del país, muchos de esos medicamentos son controlados, el uso que algunos médicos le dieron o que los medios le dieron, algunos tienen miedo de recetarlo pero lo estamos concentrando a través de esas clínicas, tenemos recetas especiales que queda todo registrado y está llegando hasta el primer nivel que es donde se necesita, lo que necesitamos es más profesionales que atiendan este patología. Otro déficit que tenemos es en **la atención de la tercera edad,** además que no tenemos geriatría en el país, casi nadie lo estudia aunque sea fuera, en todo el oriente solo tenemos uno en Usuluán y trabaja solo 4 horas al días así que se pueden imaginar que no pueden ver a todos los adultos mayores de la región, y es que sería importante porque no es lo mismo un paciente hipertenso de 40 años que de 70 el manejo es distinto, digamos que más integral, no tan sintomáticos, no es solo me duele la espalda tomate esto sino una atención más especializada al adulto mayor pero eso lastimosamente no hay acá no tenemos formación de este tipo de especialistas en este país. Y poquísimos los especialistas y si se abriera más formación debería dares más presupuesto para que llegue al primer nivel y a hospitales regionales. Si se hace un esfuerzo en el primer nivel donde se crean clubes de enfermedades no transmisibles (de diabéticos, hipertensos, adultos mayor), se dan estilos de vida, dinámicas, consejos, como grupos de autoayuda, pero hasta ahí, tenemos psicólogas que También apoyan esos procesos, muchas enfermeras que han tenido su parte de estudios en atención específica a estos clubes, y psicología como le digo aunque a veces tampoco quieren ir pero a veces la necesidad les hace salir de la urbe e ir al campo entonces siento que hemos tenido mucho apoyo. En el oriente por ejemplo tenemos un **TAC que antes no teníamos, un cateterismo, en Usulután se habilitó una unidad de hemodiálisis que antes había solo en San Miguel donde a su vez se le ha dado más equipo y personal, contamos con un nefrólogo en el primer nivel, el único del país** que está ahí en el Bajo Lempa, muy Bueno También, lastimosamente le estamos explotando más de lo que deberíamos (risas) pero es un apoyo, se necesitarían más de estas personas. Se han reforzado También los hospitales con materiales, incluso en el primer nivel tenemos glucómetros para urgencias en el tamizaje de embarazadas, también se hacen tamizaje neonatal para temas de tiroidismo, así que poco a poco yo creo que en estos 9 años de reforma a las enfermedades crónicas se le ha apostado mucho más que antes, quien no quiera ver eso es que está ciego.

- ¿Nos podría contra un poquito como es la coordinación con los 4 sibasis a nivel regional?
- Bueno, como tenemos un equipo de la región tenemos responsables de programa de cada uno de ellos, y ocasionalmente tenemos cada quien alguien, bueno de pendiendo de la parte que le corresponda tenemos cada quien alguien que nos acompaña, por ejemplo, yo tengo a alguien que es el trabajo que yo ejercía antes que es de la parte técnica. Cada programa tiene indicadores talleres convocatorias reuniones y se está intentando que éstos se hagan de la mejor manera posible, coordinamos con los hospitales, en las reuniones de RIIS departamentales, las microrredes. Los hospitales no siempre nos asisten a estas reuniones, en la **RIIS regional** que el jueves tenemos, **los 9 hospitales de la región, 4 coordinadores de SIBASI, asisten**. Y así quien va teniendo sus reuniones de RIIS departamental en la cual asisten sus propios hospitales y muchos temas se tratan, **se hace énfasis en lo materno infantil, pero también se ven temas relacionados con las crónicas** para darle más movilidad, aparte de ellos se mencionan las evaluación mensuales o bimensuales o trimestrales de indicadores de cada uno de ellos hipertensos, diabéticos y tenemos una evaluación anual que llamamos ”**Indicadores de RIIS”** dentro de estos indicadores están los de los crónicos, ahí analizamos primero hacemos una evaluación por cada departamento y se analiza la tasa de morbilidad por hipertensión, tasa mortalidad por diabetes, la razón de mortalidad por insuf renal, tasa IR, tiempo medio de hospitalización por ingreso de complicación de diabetes, varios indicadores que hospitalarios pero que también se relacionan con nosotros, grado de detección de este tipo de enfermedades, nivel de seguimiento…entonces cada departamento hace su análisis de estos indicadores. Lo mucho que tenemos es de crónica tienen su propio análisis, por ejemplo, se analiza en qué lugar está aumentando el diagnostico por enfermedad crónica, nada menos tenemos que en 6 meses hemos detectado que la incidencia de IRC ya no es Usulutan sino la Unión, atraves de estos indicadores hemos visto que hemos detectado por tamizaje. Está aumentando más y es porque también se está diagnosticando más, de ese departamento seguramente haya un municipio que hay mayor concentración, entonces esos indicadores que se nos dan a nivel central para que los analicemos y hagamos verificaciones vemos qué municipios puede ser que tenga... puede ser que solo tenga dos, pero son solo 2000 personas, pero la incidencia es mayor que en San Miguel por ejemplo que puede haber un caso y son cerca de medio millón de habitantes. Entonces nos lleva la detalle, luego lo llevamos a la macro y nos da la imagen de como región cómo estamos para tratar de hacer acciones, y lo hacemos comparativamente enero diciembre 2016 frente enero diciembre 2017, o ahora en agosto tenemos enero junio 2018 y hay un umbral que no puede aumentar más del 5-15-20% del año anterior y eso nos orienta a nosotros para ver cómo podemos hacer si estamos aumentando, entonces tenemos que ver como paramos, hacemos meseta y empezamos a descender. No perdemos de vista el **punto de vista educativo**, en esos indicadores RIIS están **cuantas personas se han formado, cuantos asisten y a veces también medimos cuantos Ecos tienen esta serie de clubes y cuantos no para así incentivar a que los apertura**, porque eso es lo que nos pasa muchas veces, de qué sirve que haya ECOs si no tienen un club como estos para darle atención a estas enfermedades y aunque lo tengan no esté activo, de nada sirve que firmen un papel que diga que existe si luego no se hacen cantidades. También está la parte comunitaria, los **comités locales de salud** **que es la innovación que se tiene de organización territorial las cuales ellos mismo también pueden solicitar o a iniciativa del promotor que se den charlas de enfermedades crónicas** o digan miren aquí tenemos muchos diabéticos, ayúdennos que es algo que desde la RIIS departamental podemos hacer. O la coordinación para pacientes que necesitan movilización específica, tenemos coordinación entre primer nivel y hospitales para darle seguimiento a los pacientes, de hecho, pacientes dados de alta en el hospital tienen que darle seguimiento el primer nivel, tenemos deficiencia en ese sentido porque algunos creen que mandarle con referencia al paciente al hospital es que ahí quedo y no le dan siguiente. Y por otro lado, también hay muchas veces que **los hospitales detectan a un paciente crónico y no nos lo informan a nosotros, muchos no visibilizan lo que es la reforma de salud, pudiéndole dar seguimiento en vez de quitarse la carga de tantos pacientes no lo refieren porque creen que no están capacitados**, dicen uy este paciente cardiaco es muy complicado, este esto otro…y viene la gente de escasos recursos que casualmente llegaron ahí o porque la familia les llevó o que se yo, pero luego el seguimiento, difícilmente pueden llegar a San Miguel por el transporte, no pueden entonces, quien tendríamos que dar seguimiento somos nosotros pero no confían en esos, se bloquean y ahí. A veces la misma comunidad de los médicos absoluticen al paciente creen que les pertenece, aunque este estable o solo con dieta no les dan de alta, y porque, porque les conviene, tienen una consulta rápida y fácil y llenan sus números. Cuando posiblemente como primer nivel podemos darle ese seguimiento consejería régimen alimenticio, pero ellos no quieren eso. Y desde el punto de vista nutricional, también tenemos profesionales en la región que nos apoyan en ese sentido con las dietas de enfermos crónicos. Lo que sí y hay que decirlo también no se siente la vinculación entre el médico especialista y las otras partes, o sea, yo soy el médico y yo lo es todo, te puedo dar tu dieta tus consejos y sabemos que no, que la nutricionista sabe más que nosotros en esa área, la psicóloga también en su área, el fisioterapista sabe los cuidados de un pie diabético, pero muchos médicos incluso nuestros hay que 300 pacientes diabéticos, 200 hipertenso, pero cuantos los ve la nutricionista, cuantos los ve la psicóloga y más los renales que se deprimen porque cree que ya les lleva la muerte, no hay esa derivación ni de especialistas de hospitales ni en el primer nivel teniendo a la mano otras personas que les pueden ayudar, no se les cita, **esa es otra de las dificultades a superarles falta a los profesionales sentirse dueños de la persona.**
- ¿Con respecto a las coordinaciones que comenta, cuantas reuniones se suele tener con los sibasis o con otros coordinadores de las regiones?
- Entre regiones sí que no hay reunión, tenemos una reunión mensual, bueno, va por niveles:

- La primera semana del mes: reuniones de **microrredes**, las pequeñas.

- La segunda semana: las **intermunicipales**, entre esos municipios la Microred podríamos decir entre esos municipios.

- La tercera semana: **RIIS departamentales**, cada cabeza de Microred del departamento, con el coordinador de Sibasi y los hospitales de la zona. Por ejemplo en San Miguel es mañana es la RIIS departamental de a la cual tienen que acudir las tres cabezas de Microred, los 3 hospitales más la coordinadora del sibasi, y cada cual dice cómo están las cosas que es lo que pasa, por ejemplo para coordinaciones con el primer nivel, por ejemplo dicen miren tenemos muchos diabéticos que necesitamos que se les haga angiografía y no tenemos como, aquí está la directora del sitial, cuando van a la especialista cuando le puedan ver con Doppler, ese tipo de coordinaciones se pueden hacer ahí.

Estas reuniones son **mensuales** por lineamiento.

- Y la **RIIS regionales** que son bimensuales, el próximo jueves tenemos la riis regional, la siguiente es en agosto, donde vemos los temas que no pudieron superarse en la otra, materno infantil, epidemiologia, hogares de espera materna, si hay algún tema relacionado con crónicas lo metemos también. Nosotros invitamos a todos los prestadores de salud sean públicos o privados del sistema de salud y los del foro nacional de salud tiene su participación en esta parte y ahí discutimos todo lo que nos compete en temas de salud de la región y como le decimos temas que no se han resuelto en los otros niveles, y que haya una injerencia, más bien que haga una injerencia yo como gerente de la región para poder solventarlo y si no puedo solventarlo yo lo llevo a una RIIS nacional, por ejemplo que necesitamos resonancia magnética porque tenemos muchos pacientes con ictus y el TAC no es lo más indicado. Mi deber es llevarlo a la mesa y que en el momento alguien diga mire, tenemos una resonancia por ahí que queremos donar a El salvador, mire en oriente necesitamos uno allá., esas son las coordinaciones que tenemos. Y también existe una coordinación informal, fíjese que ahora me llamó una doctora indicándome que no tiene acitenomifen si hay en otro sitio le préstamos y así con el ibersantan, son informales, pero desde el punto de vista gerencial tenemos que hacerlo, también con los medicamentos.
- ¿Entonces no hay una reunión entre regiones?
- Si tenemos una mensual con la Dra. XXX, coordinadora nacional del primer nivel de atención. Nos reunimos y se llevan puntos y se hacen acuerdos bilaterales, algo que no haya podido solucionar en la RIIS regional, por ejemplo, que ocupamos dos ambulancias se trata allí, ella me dice verificaremos o podemos hacerlo de esta manera y eso. Nos reunimos dos veces, una con los coordinadores regionales y otra que la llamamos la ampliada que también asisten los coordinadores de sibasi, enfermeras también, y así algo que a lo mejor se me había quedado a mí en el tintero ellos lo traen.
- ¿Y en la ampliada también participa el FNS?
- A ese nivel no, ya es gerencial. El foro nada más participa en el sibasi, microrredes, departamental y regional. En la parte nacional ya no depende de mi persona convocar, sino que ya depende de la coordinadora nacional, Dr. Robles Tica. Para convocar a estos colaboradores de la salud que es importante.
- Como ves, nosotras hemos tenido la suerte de poder conocer Bajo Lempa y Morazán, como usted comentaba el contexto puede ser muy diferente dentro incluso de la misma región, las diferencias sociopolíticas incluso el tema de violencia social que es un tema importante en este país. son algo importante pueden ser muy diferentes entre regiones, EN el caso de las enfermedades crónicas, con la existencia como nos contaba de clubes, ¿lugares para hacer actividad física, como ves que está avanzando eso en términos de adaptar más al contexto por esas propias diferencias dentro de la región?
- Pues fíjese que los profesionales van adaptándose al contexto así cada quien va hasta cierto punto innovando, algo que me falto de decir de deficiencia que no tenemos, es que por ejemplo los fisioterapista que pueden ayudar a rehabilitar un infarto cerebral, o de un pie diabético, no se tienen los insumos para ello, entonces tienen que hacer lo que llamamos RBC (**rehabilitación basada en la comunidad**) tienen UE ver como utilizan por ejemplo una fruta para dar masajes o la masa de tortillas en vez de una pelota que te cuesta 10 dólares, o un pasa manos con palos, y eso tienen que adaptarlo ellos en las comunidades y es lo que se ha visto, porque si son instrumentos bastantes caros, los Tensa por ejemplo, las bandas frías, la mayoría creo que no cuentan con eso y otro tipo de equipamiento que son necesarios tanto para tener en el establecimiento como para transportarlos, no tenemos esa serie de cosas entonces así como lo mencionas, han tenido que adaptarse, en alguno sitios que ya llevan 8 o 9 años en la comunidad ya saben que pueden hacer y que no, incluso con **el tema de violencia social** esta feo decirlo pero ha hecho que nos adaptemos o acomodemos o acordemos trabajar en conjunto con este tipo de personas, algunos condicionantes que se ponen y tenemos que acordar por el bien estar de la población, hasta el punto de tener que acceder, no es un pacto es solo para beneficiar a la población, si ellos nos dicen que quieren que les atiendan los primeros y a cambio nos dejan pasar a ciertas zonas a fumigar, hemos tenido incluso que gente de los mismos de ellos se ponen las bombas y nos ayudan a fumigar la comunidad, y hemos tenido casos de amenazas de muerte que han tenido que salir de la comunidad y al final los que pierden son ellos no nosotros, es la población misma donde sus familias viven sus madres tías hijos. Así que como tú lo mencionas también en la parte de la costa, que tienen que acostumbrarse a vivir a la isla y no viajar todos los días porque no da para eso, y ver cómo mejorar la vida de las personas en su medio de vida, estilos de vida saludables, tanto en las comunidades como en las islas o en Parquin por ejemplo, que estilos de vida pueden mejorarles según l territorio, y eso son los médicos y demás profesionales que se han mantenido en la zona han visto de qué manera que metodología que equipo pueden adaptar ellos para la zona. Por ejemplo recuerdo el Dr. Calero el nefrólogo que nos comentaba que estaba viendo casos de una hipocalemia pero especifica de aquí, en tondices es interesante ver como se adapta el diagnóstico y el tratamiento al contexto por ejemplo en las dietas recomendadas con lo que haya en la zona, por ejemplo recomendar una tortilla en vez de dos, hacer el queso menos salados, en fin esas innovaciones que son experiencias exitosas no documentadas que estaría bien hacerlo para poder socializarlas y compartirlas para mejorar entre todos la salud a nivel nacional, por ejemplo la unidad de gestión clínica renal del oriente que y ase esta casi nacionalizando y al revés, por lo que sería importante tenerlo encuentra. Yo si siento que los trabajadores de salud son los que más nos respetan el color el uniforme, hay una actitud de aceptabilidad en general, son pocas las ocasiones en las que si se ponen un poco reacios a que entremos que se hagan acciones en sus casas para mejorar la situación, pero nos hemos ido adaptando con el tiempo.
- En ese sentido, la participación del FNS, aunque no participe en la RIIS nacional, como considera que es su participación en los otros niveles regional, ¿sibasi microrredes?
- Para nosotros es una fortaleza contar con la participación social es una porque ellos son en cierta manera los vigilantes que tenemos de cosas que metodológicamente no se ven o por años y décadas se habían ocultado como es el trato humanizado a la población. Quien mejor que ellos visualizan, hacen la denuncia le dan seguimiento, que muchas veces nosotros no podemos por trabajo, también hay ciertos compadrazgos de los mismos trabajadores que tenemos equipos que no denuncian porque también tienen miedo que los pateen. Por eso el FNS es una ayuda importante, ellos tienen los buzones de sugerencia, anualmente hacemos la rendición de cuentas, ahí todos los años se les da su espacio que hagan su presentación para nosotros y es una cosa muy importante porque se ve que el pueblo que le quiere apostar a esto. En las poblaciones que están más organizadas, o que el foro las organiza mejor, estas son las que más nos participan en campañas que hacemos como de vacunación, vacunación canina felina humana, campañas contra el dengue, cuando el foro la población está bien organizada se nota ya saben cuál es la obligación de quien (por ejemplo para evitar los zancudos ya saben que hay que dejar tapados los contenedores de agua y que eso es responsabilidad para la propia población), nosotros colaboramos una vez pero saben que es una responsabilidad compartida. El foro que está muy empoderado en las comunidades y en los comités de salud concientizan a la población de que la salud es de todos, no es solo responsabilidad del ministerio, que la salud no es solo que te vengan a curar que debe ser más preventivo que curativo, y es ahí donde la gente del foro está ayudando mucho.
- Por un lado, el eje de participación como comentábamos, por otro el de la intersectorial. Por ejemplo, estuvimos hablando con una persona en una unidad de salud especializada que es la encargada del tema de saneamiento, y nos comentaba que por ejemplo en la isla de Méndez no tienen agua potable porque el sistema de potabilización del agua está fuera del sistema y por eso no tienen acceso a agua potable e incluso muchas personas reportan lo contaminada que esta el agua, entonces en ese sentido, que tipo de ligación con otros sectores o áreas de gobierno tenéis contacto para poder un abordaje más integral a este tipo de problemas, porque a salud le suele pasar que abarca todo pero también se responsabiliza demasiado a salud cuando quizás compete a otras áreas como es la potabilización del agua. Porque también nos comentaba el coordinador de sibasi la intersectorial que hay, pero más a nivel de terreno, ¿entonces desde su posición como coordinador regional como se coordina con otras áreas, como está el tema de la intersectorialidad?
- Pues en este tema de la intersectorialidad creo que estamos aún un poquito mi…con deuda, porque es cierto que se menciona entre los ejes de la reforma, pero nos cuesta mucho. Quizás desde el punto de vista del minsal si intentamos hacer los acercamientos, pero más que todo las coordinaciones con las instituciones en ese sentido hay gente que como usted menciona de ANDA por ejemplo que llevan el agua, pero que tanto nos hará caso a nosotros que un director regional de salud le dé una orden ellos están más centralizados a veces y dicen no, si la orden tiene que venir de San Salvador, de mi director general. Y así toras cosas como arreglar caminos para que salga mejor la ambulancia o limitar una vereda, muchas veces son alcaldías u obras públicas quienes tienen que hacer eso. Y parte la participación de la población, porque ese usted me dice aortita esto de medes yo no lo había escuchado y posiblemente el promotor del eco no lo vio como tal, no lo llevaron a la Microred, no llego a la regional y a mí nunca me llegó y entonces no puedo hacer nada, si no existen denuncias y reclamaciones entonces sí, sino no nos Podemos dar por enterados. Así que si necesario el trabajo intersectorial pero no lo hay mucho. Con educación es con el que más que hacemos un buen dúo.

-          Cuando comenta el escalonado que es muy Bueno pero si uno de los peldaños no denuncia no termina de llegar a otros niveles,  en ese sentido nosotras sí que hemos visto que por ejemplo las unidades especializadas suelen funcionar muy bien pero que en las unidades de salud básicas e intermedias niveles más bajos sí que vemos que quizás depende mucho mucho de los profesionales, como quieran hacer el trabajo, en el momento en que no hay compromiso o se ha relajado porque lleva muchos años trabajando allí como que no transciende el problema o la dificultad.

-          SI tenemos las mismas deficiencias en las unidades de formación, que llegan a hacer el servicio social en el último año de Carrera y pasan a ser el director d ella unidad porque no tenemos gente allí trabajando a largo plazo, sea como sea, tiene mejorar, y muchos lo hacen porque es obligatorio para terminar su Carrera y punto, se ven que están ganando dinero siendo el director y punto, pero no más que eso, yo diría que el 25% de los médicos directores que llegan a esas unidades llegan con esa mentalidad de Bueno, ya termino en un año la carrera y voy para el hospital, pero llegar con ganas de hacer cambios en la comunidad, por un año no, que no tienen tiempo que no les da la Cabeza, me da hueva, ósea eso no funciona bien.

-          Bueno, ya para terminar, qué éxitos resaltarías un poco de la reforma que has visto tú desde tu posición que pueden ser fortalecidos, ahora que estamos en época de elecciones y así, qué éxitos consideras tú que deberían ser más visibilizados.

- ¿Qué éxitos? Es que todos, para mi todos, los nuevos ejes. Cuando llegan los del servicio social yo les doy el primer tema que es la reforma, es el tema de inducción al año social. Yo les comienzo a explicar ahí que cuando en la vida una embarazada tuviera la oportunidad de que una especialista la Viera en su casa, en su cantón, jamás nunca nadie se podía imaginar que un gobierno pusiera en marcha una reforma de salud como esta, incluso, que se le haga una ultrasonografía en la unidad de salud cuando antes viajaba al hospital y el especialista También le veía cada 3-4 meses en el hospital departamental. Sierpe les pongo el ejemplo porque me gusta, hay una comunidad en Corinto que se llama Hondable, la gente antes se tarda 2 horas de Hondable hasta la carretera de Corinto, entonces antes para que les Viera el especialista tenían que ir hasta Gotera que se demora como 4 horas, hoy llega hasta sus casas, alguien que piense que eso es maligna, más maligno es él. O que a su hijo le ve un pediatra le ve un especialista o le toman un examen del Corazón cuando antes se tardaban dos meses para citarle en el hospital y que se los mire un especialista yo creo que quien no ve eso, este mall de la Cabeza. Aquello que llegue la gente a su comunidad que saquen embarazadas que ese organice que le atiendan con calidad en el hospital que para a su hijo en el hospital, eso antes no se veía, que las embarazadas de alto riesgo tengan un lugar de espera maternal para estar bien resguardadas cuando antes parían en los cantones y se les morían ellas o sus niños y no se vea eso como un beneficio... Que los medicamentos son efectivos, tenemos control de calidad y se le da gratuitamente a la población y que no vean eso que antes se pagaban 50 dólares por un parto normal y 100 por una cesárea, y que ella gente no vea eso...están mal. Que no vean que en su cantón tiene una clínica cuando antes caminaban antes. Yo antes cuando trabajaba en la sopita llegue a conceptualizar el concepto de pobreza en la atención, yo seguía pacientes diabéticos e hipertensos más que todo, y me recuerdo que le decía, mira señora, ¿hace dos meses tenía cita conmigo y no vino origen no? No tenía dinero doctor, cuando yo empecé a conceptualizar el no tengo dinero que era: 1 dólar para el transporte, 2 dólares para comida, otro dólar que le dejaba a los hijos y al marido para la comida, otro para la consulta y otro para las medicinas son 6 dólares en total, hoy puede ser que los jóvenes se lo gasten en una recarga en el celular, pero para ellos eso son millonadas. Y ahora que es todo gratuito solo necesita un dólar para el pasaje, y alguien que no vea eso. Lo que sí que diría es que no se hace falta rellenar la reforma, crear más Ecos, ese 40% sin intervenciones, crear UCCP, el complementar la reforma. Porque de ahí la implantación se está mejorarnos cada vez más, y esa es la calidad que se tiene, por ejemplo en mi pueblo necesitamos 10 Ecos, sin embargo solo témenos 3 unidades, dos en cantones y uno en el pueblo, entonces que pasa, que se satura, entonces I hubiera más ecos yo creo que mi pueblo sería más saludable, una mejor calidad de vida, no porque no se teja a las personas trabajando sino porque la cantidad es grande y a veces tienen que esperar hasta 2 y 3 horas en ser vistos. Con los medicamentos que tenemos y que los especialistas lleguen a las casas, Ya con la parte de investigaciones que tenemos serian mejores más verídicas, podríamos saber de qué en realidad nos estamos enfermándolos salvadoreños con nuestros datos y no lo que dice el jornal o medicine, sino que el pipil jornal publicación y eso es parte de los ecos para saber todo eso, como la implementación de las tabletas que tienen ahora los promotores y eso es muy valioso para poder tener actualizados los datos pro ejemplos de cuantas embarazadas hay o tener al día cuantos niños, cuantos Muertos, cuando antes solo en papel lo teníamos, por eso eje por eje, son grandes, y lo hemos montado aún falta pero es la parte que nosotros no podemos manipular que es el dinero, no está en nuestras manos poder usar ese dinero para hacer ECOs por ejemplo es del gobierno, si el nuevo gobierno impulsa el 40% que nos faltan estaría bien. En Perquin que ustedes estuvieron, en el hogar de espera materna el 80% de las mujeres que se alojan allí, son hondureñas, que quiere decir esto? Que tenemos mejor sistema sanitario que Honduras o que Nicaragua, en la zona de La Unión hay muchas mujeres que paren a sus niños y lo asientan en El Salvador porque van a tener atención gratuita, yo creo que en muchas zonas de occidente debe pasar lo mismo las zonas fronterizas y es que aquí es gratuito y no discriminamos por nacionalidad ni nada, no somos xenófobos, aquí les emos como hermanos salvadores, pero enférmate tú en Honduras no nos ven como hermanos salvadoreños, aunque saben que aquí se lo damos gratuitamente, hay que ir a un seguro privado a consultar, todas esas cosas, muchas veces a la gente se le olvida es cortoplacista ahora creen que es un deber y una obligación del estado los servicios de salud cuando antes nunca era así, pero no luchan para defenderlo muchas y tampoco quieren apoyar para que esto avance, da un poco de pesar porque es para beneficio para otras poblaciones que no han podido acceder antes y les podíamos apostar para que esto avance pero sin el incremento de Ecos o UCFF no podemos hacer más, hacemos lo que humanamente podemos con lo que poco que tenemos. Como alguien dijo, **“Administradores de la pobreza” y eso somos, con lo poco que tenemos hacemos milagros.** Pero sí, todo oeste es un gran avance, en el Oriente el hospital regional no podemos envidiarle nada, mucho a San Salvador, porque de los subespecialistas, solo dos no tenemos que son el endocrinólogo pediátrico y el neurocirujano pediátrico eso si no tenemos, de ahí todo lo demás se tiene en oriente, pediatras, neonato logos, oftalmólogos, urólogos, primatólogos, endocrinólogos de adultos eh..que más te puedo decir, todo esto, tal vez la deficiencia con pediatría pero vamos oncólogos, nemato oncólogos, lo único que sí, al igual que se va a modernizar el hospital Rosales, tener mejores áreas para atender eso aquí., pero sí, son cosas que solo con dinero se pueden hacer y un poco que los compañeros le den más calidad de atención a la población y en eso hay que ser francos, hay malos trabajadores, llegan tarde, se van cuando les da la gana, así que sí eso es una de las dificultades que hay que mejorar con la reforma. Lo de abastecimiento de medicamentos ahora ya siempre anda arriba del 80%, los tiempos de esperan en los hospitales se han reducido drásticamente, en algunas llegas hoy y casi ese mismo día te está viendo el subespecialista. Así que ya nos queda nada más la calidad de la atención, que eso ya no te puedo obligar yo, sino que es tu misma quien lo tiene que implementar, que potenciar y…que el foro tiene que vigilar (risas).

Quizás vosotras como europeas cómo nos ven a nosotros como nos ven a nosotros que estamos avanzando en la reforma de salud.

## Staff interview 5: Health educator

Entrevista con: Educadora

Fecha: 13/06/18
Lugar de la entrevista: Unidad Comunitaria de Salud Familiar La Palma
Código del informante: SFML006
Genero del informante: F
Nombre del entrevistador: ML

**Sección 1**: Perfil del participante y función en la prestación del servicio del primer nivel de atención

**I: Me puedes explicar un poco sobre ti, ¿cuáles son tus antecedentes tu capacitación, como ha sido tu trabajo en la unidad de salud?**

Educadora La Palma. Aquí en la Unidad de Salud, ya tengo 8 años de estar trabajando, porque entre en el 2011 con la reforma. La educación para la salud antes en el primer nivel de atención, no era tan visible, sino que era implícita, ósea se daban charlas, se hacían reuniones con grupos, se tenía un objetivo, pero no se tenía un programa. Entonces ahora promoción de la salud, con la reforma si se ha ido dando más auge, al principio nosotras como educadoras para la salud, al menos en mi área, y creo que en los demás también, tuvieron cierta resistencia el personal de salud ante un educador, porque como era algo como que alguien venía a hacer lo que nosotros ya estábamos haciendo, entonces venía específicamente a eso, entonces a lo largo de los años uno se ha ido abriendo brechas, o abriendo caminos digámosle así, en una forma positiva, porque hoy en día un educador para la salud es fundamental para un ECO especializado.

Mis capacitaciones en enfermedades no transmisibles, el ministerio nos ha capacitado mucho, al inicio por allá como el 2012, nos dieron un curso completo, que era específicamente sobre diabetes, tanto tipo I como de tipo II, y fue con coordinación con ASADI (asociación de diabéticos), es una asociación privada pero también trabajan mucho con el sistema de salud, entonces ellos son los que están trabajando y los que nos capacitaron. Después que se creó la unidad de enfermedades no transmisibles en el ministerio de salud, hace como dos años que ya empezó, con más auge, y ya con eso, era como más visibles las enfermedades crónicas. Siempre se tenía dentro de las unidades un trabajo permanente, más que todo con consejerías, entonces mi trabajo también incluye tres tipos de población:

- la primaria que es persona a persona, que serían las consejerías, ya un sesión específicamente para grupos específicos, así como el que tenemos ahora en formación que es acerca de las enfermedades no transmisibles, está dirigido para todas aquellas personas que tienen algún riesgo de tener una complicación o de tener una enfermedad no transmisible, como aquellas personas con obesidad, sedentarismo o fumadores, o alcohólicos, que ya sabemos que es uno de los fundamentos de enfermedades crónicas.
- Ya la población secundaria sería la familia de la persona, la familia que influye en las decisiones de las personas, entonces en un diabético quién le da de comer o si el mismo se prepara la comida, o con quien vive, si tiene apoyo familiar.
- La población terciaria es la comunidad. Son grupos de personas más grande y también líderes y lideresas que pueden influir en la toma de decisiones de una persona.

Entonces, estas son las tres poblaciones que nosotros nos dirigimos, como educadores. Igual con promotores de salud, nos dirigimos casi de la mano, porque para un educador es bien difícil hacer una convocatoria si alguien no me conoce como líder, entonces ya nosotros con 8 años trabajando, ya todo el mundo nos reconoce, pero los promotores de salud son los encargados de las convocatorias, porque ellos saben a qué población, ellos saben hacía quien va dirigido, entonces, todo eso también nosotros tenemos en cuenta.

**I: Entonces en tu trabajo eres parte del ECO comunitario y también trabajas en esta unidad**

A las comunidades salgo al menos una vez al mes con cada uno de los equipos ECOS, aquí tenemos a cargo 9 ECOS; aquí en la palma son 4 más el especializado entonces 5, en San Ignacio son 3 y dos en cítala. Entonces se trata de una vez al mes ir a cada ECO, y luego piden apoyo de algunas actividades ya sean grandes, pequeñas o específicas, entonces de tratamos de tener la manera de tener influencia en casi todos los ECOS. Hay otras veces que pueden ser coordinaciones aquí en la palma. No necesariamente tiene que ser aquí en la unidad sino de que estamos también con protección civil, en las comisiones, con la comisión de prevención de violencia, entonces como le digo estamos en todos los programas del ministerio, teniendo inherencia en casi todos.

**I: ¿Dónde realizas tus actividades?**

Aquí en la unidad de salud, yo hago las actividades 1 a vez a la semana y el resto de actividades las hago en campo, entonces nosotros trabajamos a base de programación, entonces el último día cada mes, nosotros nos reunimos y ya tenemos que tener nuestra programación hecha para ver a donde vamos a ir y que vamos a ir a hacer.

**I: ¿Las actividades las realizas en coordinación con algún profesional?**

Las actividades algunas las hago yo sola, y otras se programan con algún otro profesional específico, por ejemplo con una promotora en específico, no con las 4 promotoras, porque cada una tiene una zona, entonces ahí en esa zona es donde vamos a hacer esta actividad, y también como educadoras de manera independiente se hacen coordinaciones con las unidad de la mujer de la alcaldía, de la unidad ambiental de la alcaldía también, con ONG como ayuda en acción que está trabajando aquí en la zona, o plan internacional que está trabajando aquí también. Como educadora, como tal si se hacen coordinaciones siempre en nombre del Ministerio de Salud, lo que se pretende es que el Ministerio de Salud tenga inherencia en todos los ámbitos, y de ahí representación de la Dra. en algún centro.

Yo soy la única educadora de la Microred.

**Sección 2**: Comprender las trayectorias de los pacientes en el primer nivel de atención

**I: Me podría describir su participación con pacientes con enfermedad crónica y el resto de poblaciones con las que trabaja.**

Con la población primaria que sería el paciente con una enfermedad crónica, tenemos una forma de atención: primero lo ve el médico y según el médico así se manda para alguna consejería, nosotros intentamos abarcar los estilos de vida saludables, entonces a través de la consejería, nosotros educamos al paciente. También depende de qué tipo de enfermedad se tenga, porque a veces es la diabetes que es solo una, o también se tiene diabetes e hipertensión, u otros problemas verdad, porque entonces lo que yo pretendo que el médico me indique. Por lo tanto, esa es la cadena de atención primero el paciente llega al médico, y generalmente son los pacientes del Dr. Internista, y es el, el que me refiere las personas necesita una educación puntual, o alguna asesoría de algún caso que ellas lo necesiten, entonces así es como se trabaja en lo primario. En lo secundario, soy yo la que le hace una recomendación al paciente para que venga con su familia, en algunos casos se puede trabajar en otros no, es bastante difícil porque a veces el paciente viene solo, o si vienen con alguien más, como que no prestan tanta atención, como si ellos fueran el paciente, y a veces son los mismos pacientes los que no prestan atención, entonces solo le dicen a uno, “si, si lo voy a hacer”, pero al final los vemos el siguiente mes y siguen subiendo de peso, o su creatinina esta más arriba, entonces son cosas que nosotros debemos de ir superando con ellos, porque así como personal de salud, bueno al menos yo, siempre intento de ser recurrente siempre en lo mismo, entonces cuando vemos que la persona ya lo ha superado, allí es donde hablamos de otra cosa, de otros problemas, en verdad se forman compromisos también en la misma consejería. Hay muchas barreras que nosotros intentamos de superarlas y algunas veces no se superan porque la misma población hace la barrera, entonces eso es bien difícil, pero con la familia uno le orienta, para que en el próximo control venga con la familia para explicarle a la familia. Y ya la población terciaria es cuando ya se llama a los grupos de personas en generales, ahora se trabajan con los grupos que ahora están justo formándose de autoayuda de enfermedades no transmisibles enfermedades no transmisibles y para todos los enfermes crónicos.

Ya con la población terciaria ya hay otros grupos que se convocan a través de los promotores de salud, o en los acercamientos comunitarios, en cada uno de estos acercamientos, uno de información, charlas educativas o sesiones a la gente que participe en el acercamiento. El acercamiento es donde la gente llega a su control, ya sea con pediatría, con médico de familia, con cualquier otro. Entonces ahí es donde la gente se concentra y ahí es donde uno aprovecha que es donde la gente se ha concentrado, y ahí se llega a toda la comunidad. O en las asambleas comunitarias también, los promotores de salud tienen como meta hacer dos asambleas/anuales en la comunidad, entonces son 16 promotores solo en la palma y son 33 promotores en toda la Microred más los dos supervisores específicos, entonces ahí se trata de participar en las asambleas comunitarias y ahí es donde se desarrollan temas de prevención. En estas asambleas participa el doctor encargado del ECO, a veces yo, las promotoras.

Son las promotoras de la salud quienes dan el diagnóstico de la comunidad, como están, las enfermedades más conocidas, más consultadas y todo eso, la situación epidemiológica por parte del doctor y también lo nuevo que viene, las campañas de vacunación, la importancia de los controles que ya serían por enfermería, entonces si hay participación de todos los perfiles de salud.

Para las campañas se les informa o bien en los acompañamientos se les invita o bien se les da un papel recordatorio, y a través de las promotoras se les ha dado un número de personas a participar y así es como hacemos la campaña.

**I: Principales desafíos que enfrenta al cumplir su función**

Primero la cultura, la cultura asistencialista, y curativa que se tiene de parte de la gente que muchas veces una sola charla, una intervención educativa, una sesión donde no lo van una medicina no lo ven como algo fundamental o importante. Entonces eso nos lleva a nosotros a un mayor esfuerzo a acercarnos a la gente, pero no para dejar una huella en ellos, sino que ya hasta que las personas están enfermas, ya ellos se acercan mas, ya ponen mas atención, les ponen mas interés a la prevención de las complicaciones, no sería como algo preventivo, sino que ya sería como algo mas paliativo, curativo.

Entonces otro desafío es la resistencia todavía del personal de salud, verdad, a considerar la educación como carga laboral, entonces, con todo lo que ya se pide dentro del Ministerio, una intervención educativa les hace como, no a todos, solo algunos como mayor barrera, porque si hay algunos que son como muy este, que se prestan para eso, pero si hay otros que no, que no toman la iniciativa de invitarme a mi para desarrollar algún tema, no si no que esa es otra barrera que tenemos el mismo personal de la resistencia al cambio también. Muchas veces aquí se ve como polarizado por política, entonces piensan que un programa es por una política, viene otro tipo de gobierno, viene otra política, entonces ya toda la gente que ha estado ahí, y mas que todo, no el personal nuevo, sino el que lleva mas tiempo, hay como una resistencia a cambio, pero ya se esta superando, cuando se ve que realmente los resultados si que son buenos.

**I: Que estrategias para superar barreras?**

Para la cultura asistencialista una de las estrategias es, que yo he buscado, es ponerles metas, digamos incluso al mismo personal de salud, ponerle una meta de este, de dar una charla todos los meses, antes era bien difícil, ya solo con la reunión una sola charla se entregaba, y el resto no las hicieron, o dicen que las hicieron, pero luego no las hicieron, entonces, eso ya se esta superando, ya con las metas que se les ponen, los planes educativos han fortalecido también esto, hay por ejemplo un plan educativo de promoción de la salud en donde se colocan ahí todas las actividades que posiblemente halla durante todo el mes entonces ya eso a uno, a casi todos tenemos una meta que cumplir, entonces ya con eso como que se cumple mas, otra forma es capacitando también en educaciones continuas con ellos de que una capacitación, que es una charla, que estrategias educativas se pueden utilizar, entonces la capacitación constante hacia ellos es muy importante, y otra estrategia es llevarlos, es yo ir a hacer una actividad y ellos que se vengan conmigo, y que también tengan una participación, que no solo vayan de oyente si no que participen; por ejemplo, con los promotores de salud, si vamos a hablar de prevención de las diarreas, yo doy el tema y ellos que den la demostración del lavado de manos, entonces ya como que nos dividimos el trabajo educativo y ya ellos también van a practicar.

En general hay que elevar el interés en las cosas preventivas, no solo como que una educadora no se quede como “ah ya no les voy a decir las cosas porque luego no me hacen caso”, digo no, sino que hay que seguir utilizando técnicas educativas, como darles un panfleto que puedan leer, entonces ese tipo de cosas también se han realizado, pero si mucha gente se ha quedado en el camino como quien dice, porque las citas son muy largas, o porque la gente vive demasiado lejos, verdad, y muchas veces también porque no se llega a todos los ECOS correspondientes, el doctor especialista, solo llega a todos menos uno que no va de toda la microred, y hay mucha gente que no llega a su control, uno por el transporte, otro porque pueda ser de que ellos tienen otra cita, digamos que la tienen en el tercer nivel de atención cuando justo tienen el control, entonces no asisten. Entonces se han intentado buscar estrategias, porque por ejemplo el Doctor especialista tiene un tiempo muy limitado por paciente, entonces ya la educación no se puede dar extendida, entonces eso ya pasa para donde mi, pero si en donde si tenemos mas dificultades, en donde es mas importante que se haga mas recurrente esa educación, es aquí, por que aquí viene el flujo de pacientes de todos lados y mis actividades a veces no solo son especificas de consejería sino que también tengo que andar en todos lados con otras actividades también entonces quizás esa seria otra barreras pues, tiempo profesional y personal muy corto, porque el educador no va a cambiar el comportamiento de una población de casi 15000 personas que es lo que tiene la microred, asi que es bien complejo.

**Sección 3:** Trayectorias de cuidado de personas con enfermedad crónica

**I: Me podria describir cual es el camino que recorre un paciente en el abordaje de su enfermedad por el primer nivel**

Primero se detecta a través de una consulta general, se detecta un paciente que esta pre-diabetico, pre-hipertenso, o ya con alguna enfermedad avanzada. Entonces pasa consulta en cualquier unidad y el medico general le hace la entrevista, previa a la consulta con el especialista, entonces, ya si el encuentra alguna cosa, también por todos los factores de riesgo que el paciente menciona, se le hace unos exámenes, de todo lo que hay aquí, se mandan los exámenes, se hace los exámenes y depende de los resultados se hace una interconsulta con el especialista, entonces el medico consultante general hace la consulta con el especialista, y el paciente según se valore se puede quedar con el especialista par que le lleve su control, sobretodo los pacientes que se encuentran descompensadas, o por cualquier otra cosa, porque tienen predisposición a cualquier otra enfermedad, entonces todo eso se tiene que controlar a través del especialista, y ya no vuelve al general, si el especialista decide que se queda con el paciente; por tanto se queda como paciente recurrente. También esta la *receta repetitiva*, que es otro programa que nosotros tenemos, que ya el paciente viene a recoger su medicamento cada mes y a los tres meses tienen que venir a pasar su control (ver su presión…). Por tanto, cada tres meses están en control con el especialista. Esa en cierto modo es otra barrera, porque con la receta repetitiva pueden venir cualquier persona a retirarles el medicamento con la boleta, y no viene el paciente, solo para la consulta de su control. Entonces si viniera siempre el paciente ahí tendría yo mas rol, porque se podría educar al paciente de alguna forma, o explicarle como se va a cuidar los pies a un diabético, por tanto, tocar cada uno un tema. Entonces, siento que pasa mucho tiempo sin educación, pero también en donde se aprovecha es en el tiempo que se hacen los grupos de auto ayuda o los grupos de diabéticos o hipertensos, o de enfermedades no transmisible, ahora se incluyen a todos en los grupos, cuando se tienen dislipidemias, colesterol, triglicéridos altos, obesidad, entonces ahora se incluyen a mas pacientes. Después de la consulta con los especialistas, el los retorna, si ya es por control de su presión, de su glucosa, entonces ya pasa al medico general, ya no regresa con el especialista si el considera que no tiene ningún otro factor de riesgo, pero si esta descompensado, si ve que es un paciente de riesgo o si ve que viene desde muy lejos, también si pasa con los especialistas.

Ya en los acercamientos comunitarios, aquí es mas reducido e número de pacientes por el transporte, de la salida de aquí a la llega de allá, las horas de consulta, entonces le ponen menos pacientes que los que ve allá. Pero en eso el paciente también puede entrar en contacto con el especialista y con el medico general.

**I: ¿Que pasos tu tiene para llegar al paciente?**


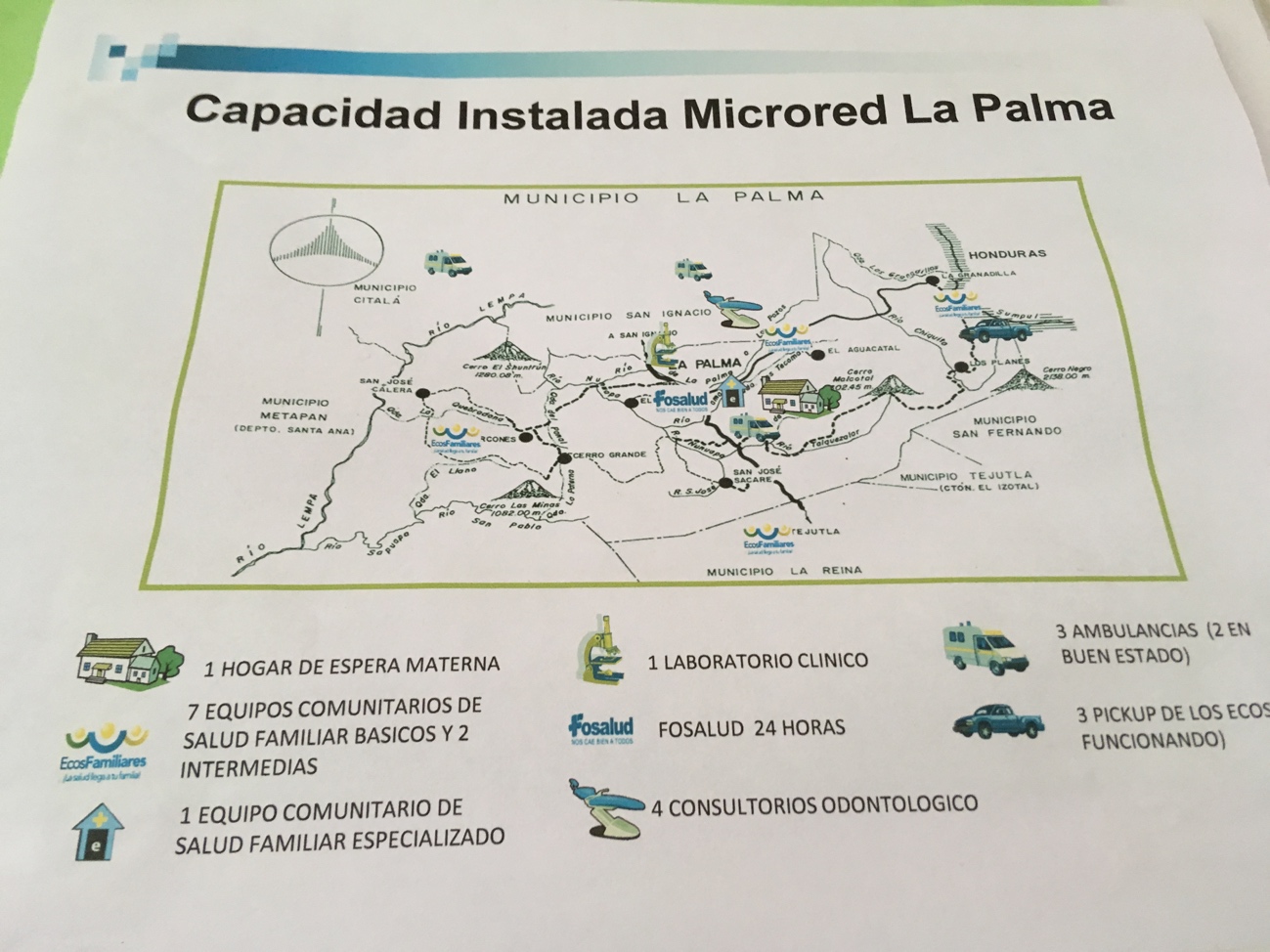


Además de aquí en la consulta a través de la derivación del especialista, también se hacen las visitas domiciliarias, con el promotor de salud, entonces a veces con los mismos promotores se coordina la visita, a este tipo de pacientes, porque el promotor de salud tiene que hacer una visita de riesgo, de riesgo familiar. Entonces digamos por ejemplo los promotores tienen visita de riesgo 4 al mes, entonces se decide, un paciente de enfermedad crónica se tiene que visitar por lo menos una vez al año. Si está bien, digamos si no ha tenido que haber sido referido a ningún lado, si el mantiene su control y todo bien, entonces a ese paciente lo tienen que visitar tanto el promotor como el medico una vez al año, es como *dispensarización.*La dispensarización se tienen dibujados las zonas de cada una de las promotoras, en donde se puntea, aquellas personas que son riesgo familiar 1, 2 o 3, porque se tiene una ficha familiar. El 1, es el aparentemente sano, que no pasa consulta, que no tiene ningún otro riesgo, un adulto digamos contemporáneo.

Por ejemplo la promotora aquí tiene 4 zonas (A, B, C, D): la zona D es la zona más lejana que está en las montañas, allí en moribundo, el aguacatal, entonces no se tiene a todas las familiar, pero las zonas de aquí urbana, cada familia tiene una ficha, y la familia esta punteada con numero familiar, entonces esa familia esta como segmentada en cada uno de los grupos familiares, según qué riesgo tiene, una mujer embarazada es riesgo número 3, con junto con las enfermedades crónicas que tienen que visitarse primero según lineamiento. Cuando ya salen de embarazadas pasan a riesgo 2, entonces siempre sigue en riesgo, pero digamos no es ni riesgo alto ni riesgo bajo. Ya el niño, por ejemplo, ya es otro tipo de riesgo, y de ahí se va punteando. Y ya cuando la promotora tiene que salir a campo, ya ahí dice, voy a ir a este lado, a visitar a la embarazada, entonces van y les hacen un familiograma, entonces se van punteando como está la familia, si todavía esta como empezaron, y ahí tienen una Tablet donde meten todos los datos y esa Tablet se va directamente al sistema y es ahí que la familia queda bien identificada. Esto es algo como muy nuevo y muy bueno que se está realizando.

Entonces los promotores de salud si van alguna visita de riesgo, sobre todo esto se hace casi que, en las zonas rurales, entonces los promotores le dicen “venga licenciada, quiero que me acompañe a donde un diabético”, entonces es riesgo 3, entonces ellos lo marcan y vamos a la casa de ellos, y vamos donde ellos, se les da consejería en la casa y también se ve como es el entorno de la persona. Yo voy a la visita domiciliaria cuando me lo pide el promotor de salud y se programa con ellos, exclusivamente con promotores, primero porque ellos conocen la zona y la gente, y ya la gente tiene como más confianza cuando está el promotor que no cuando este uno solo. Así es como tengo el primer contacto con ese paciente. Ya después del segundo contacto lo podemos ver a través de las distintas actividades educativas que hacemos. También a través de los maestros es una buena forma de entrar en contacto con los adolescentes o con niños, quizás ahí se tiene menos al promotor porque ya se tiene como una coordinación ya MINSAL con educación, entonces ya es más fácil y uno llega, y ya le dan el espacio, pero en algunos lados tiene uno que coordinar previo con el mismo profesor, y de ahí el mismo profesor de puede llamar a uno “mire que necesito que nos venga a dar una charla de salud sexual y reproductiva, o del uso del condón” , entonces ahí es donde ya nosotros hacemos el tiempo dentro de nuestra programación y vamos.

I: Que tipo de actividades que realiza con los pacientes en relación a la enfermedad crónica

- Cuando hacemos la consejería, hacemos un plan alimenticio, más que todo se le escribe, y también se le ayuda a mantener el orden de sus medicamentos, se le hace un plan de medicamentos, que según la prescripción que le haya hecho el doctor así es como nosotros hacemos el cuadrito y porque mucha gente dice “es que yo no me acuerdo de la hora de la pastilla”, y a lo mejor otra familia que si tenga dentro de casa, entonces le hago un cuadrito donde dice la medicina y la hora, se le específica a qué hora. Entonces ellos ya se llevan el medicamento con cómo se lo va a tomar. Porque si uno le dice, “esta se toma una cada doce horas” al paciente se le va a olvidar, porque no sé cuántas horas son en la tarde, y a veces le dicen en la farmacia, cuando se le despachan, le dicen se va a tomar una cada 8 horas, entonces ya el paciente como que, por lo menos las personas con así crónica, la mayoría son adulta mayor, pero también hay adultos joven, adulto masculino, y ahí es donde se les hace el plan de medicamentos y el plan de la alimentación. Bueno yo he hecho un material educativo que se le enseña lo del *plato saludable*, y ahí se le ha agregado lo de los minutos que hay que caminar, o hay que hacer algún tipo de ejercicio, y también hay material educativo donde pone las cosas que no tiene que consumir, más que todo de alimentación, de cómo que le refuerza más lo que uno ya le ha dicho, que es la enfermedad, cuáles son las causas, cuáles son las consecuencias, entonces ahí se le describe el tríptico. Entonces son 4 cosas: plan de medicamentos, plan de alimentación, plato saludable y explicación de la enfermedad; que se utilizan con el paciente.
- Ya cuando uno llega a la comunidad puede desarrollar charlas educativas que se utilizan a veces algunas laminas que se llaman *tarjetas ara*, que son como una estrategia para que la gente de la comunidad, que no sabe leer, porque claro si yo le doy un papel a alguien que no sabe leer, va a ser como ilógico. A veces también se le indica; se le da la charla, se le da el material, y se le dice que, si no puede leer, que lo lean en casa, alguien que si puede leer que le pueda explicar lo que dice ahí, a parte de lo que ya se le ha explicado. Otra cosa que hago es ir a las radios locales, donde ahí se desarrolla un tema, acerca de enfermedades no transmisibles y de todas las otras cosas enfermedades, entonces según la programación o según la priorización. A parte, hago murales educativos que están en casi todas las paredes del centro, también sesiones educativas con grupos organizados, embarazadas, adolescentes, adulto mayor que ya tenemos los grupos específicos. Los ECOS también tendrían que estar actualizándose de esto, pero es verdad también que, por el tiempo, y otras circunstancias, quizás lo hacen no mensual, si no que una vez cada dos meses, entonces es menos cuando yo no participio directamente en el ECOS, sino que solamente cuando llego al acercamiento. Otra técnica es los grupos de autoayuda, que eso se trata de hablar de las enfermedades en sí, de cómo han superado, o de cómo se sienten, y ahí se autodesarrollo. Entonces nosotros damos un pequeño tema antes de comenzar, y después se hace el conversatorio acerca de esa enfermedad.

**I: ¿Me podrías poner algún ejemplo de algún paciente de como has llevado a cabo la actividad?**

Los apoyos son sobre todo en hacerles demostraciones de como nosotros podemos mejorar nuestra salud, no soy una nutricionista, pero si tengo conocimientos en cómo ayudarles en su plan alimenticio, entonces ya empezamos a hacer una lista de intercambios, digamos si yo quiero comer pan francés o pan dulce, que puedo intercambiar, que puedo comer en vez de eso, entonces la gente como que va tomándose bien la consejería. Además se les revisa los pies y se les explica como debe ser el zapato, y se le explica que por lo menos una vez a la semana tienen que verle los pies, no el mismo, sino algún problema, porque uno con la negación puede ser que nosotros no veamos la realidad, ya uno les explica cómo hacerlo o cuando llega alguna hija o algún pariente que es ahí el contacto con la población secundaria, se les explica que hay que verles, a ver si tiene hongos, entonces de esa manera uno trata de ayudar, se le da todo el material educativo y también se les invita a los grupos, a las sesiones educativas que se están realizando.

Ejemplo de un paciente

Tengo un paciente que uno siempre lo recuerda bien problemático digamos así, en el caso que tienen negación entonces, era una persona que aunque viniera al control seguía con los mismos hábitos, no tomaba agua, trabajaba debajo del sol, entonces él cuando vino ya con una enfermedad renal, porque el sí que era renal, ya como que se asustó, entonces ya sí que cuando le dijeron que tenía un daño renal nivel tres, ya si eso era bastante impactante, ya ahí entonces el señor mejoro mucho con la educación, y era alguien que era bastante resistente al cambio, decía “yo miré como va creer que voy a dejar de comer arroz, y me voy a comer solo una tortilla”, ósea que él creía que eso no iba a poder hacer, y quizás este sea uno de los casos exitosos porque no empeoro, sino que se mantuvo, quizás obviamente no se mejoró. Igual hay otras personas que también han logrado bajar de peso, y manteniéndose con una dieta estricta. Hay historias que yo puedo contar, y otras que me las han contado mis compañeros, por ejemplo, la nutricionista me contó que había mujeres que no podían dar de mamar, entonces que el bebé lloraba, qué me quería meter la pacha, o cosas así; entonces ya todo eso se ha ido superando bastante.

**I: Teniendo en cuenta el contexto de violencia y las causas prematuras de la enfermedad, ¿cómo es tu acercamiento a esta población?**

Para acercarnos a la población joven se hacen intervenciones directamente con grupos de adolescentes, en las escuelas o en los centros escolares o a través de la radio también, que mucha gente joven escucha la radio, y ahí se les pasan cuñas radiales donde se les da información, se promociona la salud específicamente, de ahí se acerca también a través de los televisores que se les ponen videos para que la gente vea, y de igual manera a la gente que llega a las unidades de salud ahí también se aprovecha, en las asambleas comunitarias también, en las reuniones de padres y madres de familia de los niños que llegan a las escuelas, entonces son oportunidades que uno aprovecha, aunque sean 15 minutos que me den en el espacio suficiente, y a veces a los profesores como ya saben, le pedimos que nos concedan 15 minutos y a veces yo me extiendo media hora, incluso a veces me lleva hasta una hora, pero el tranquilo porque sabe, que no es que estemos hablando de otras cosas que no le vaya a servir a la población. Pero incluso de esa manera no es suficiente, como todo, pero algo se llega a la población que no vienen a consultar temprano. También nos acercamos en las movilizaciones sociales que se tienen de parte de otras unidades, como la alcaldía, los centros escolares como ya dicho que también hacen mucho, cooperativas también que también hay muchas cooperativas de café, artesanías. Es en algunas reuniones que yo me doy cuenta y entonces aprovecho para promocionar todos los temas. Por tanto, si con alguien del equipo ya hay alguna con programación entonces yo me sumo y si soy yo la que tengo la iniciativa entonces yo propongo.

**I: ¿Como identificas tú las necesidades de salud?**

Primero son por los lineamientos que hace el ministerio de salud, entonces sabemos que no nos podemos olvidar de ningún lineamiento porque sabemos que todos los programas están siendo monitoreados, por tanto, intervención tenemos que tener en algún tema. Entonces, yo voy a ver el programa y si veo que no hay ninguna intervención entonces tomo yo la iniciativa y propongo alguna intervención, Y si no, también por las situaciones epidemiológicas, como por ejemplo ahora que estamos al alza de las diarreas, entonces ese es el tema que se trata con todos los grupos, por ejemplo, con los adolescentes también se habla de diarreas, aunque tengamos otro tema programado, entonces se prioriza, según priorización también de temas.

**I: ¿Qué cambios con la Reforma en las ENTs?**

Ha cambiado mucho desde la reforma para acá, porque ha sido en uno de los puntos donde nos han capacitado bastante, y más aún con la creación de la dirección Nacional de enfermedades no transmisibles, entonces ha ayudado en muchas cosas. Desde que, hace unos tres o cuatro años para acá ya, están todas las personas casi hablando de lo mismo de las enfermedades no transmisibles, y de igual manera casi las barreras que poníamos desde el principio, una resistencia al cambio en este tipo de actividades, porque antes, y esto si se ha sido un cambio muy evidente, antes sólo teníamos el grupo de adultos mayores el mismo que el grupo de no transmisibles, de hipertensos de diabéticos. Entonces si teníamos adultos mayores que tenían alguna enfermera transmisible pero también sabemos que hay de otras edades, que están padeciendo de las enfermedades muy jóvenes. Entonces todo eso si ha cambiado bastante, se hace la distinción entre adulto mayor, se incluyen los temas eso sí, pero no deja de ser un grupo específico.

Otro cambio desde la reforma, han sido los lineamientos, lo del cáncer, algo muy bueno que se han creado materiales educativos, para ese tipo de enfermedades. Se han creado fichas también para pegar, para hacer murales, entonces todo eso también ha mejorado bastante, la gente ya habla sobre la prevención de enfermedades no transmisibles.

También, se han incluido los cafetines saludables en las escuelas. Los cafetines saludables son unas tiendas donde se prohibieron cierto tipo de alimentos, hay una lista de alimentos prohibidos para ayudar a la prevención de las enfermedades no transmisibles, entonces por ejemplo churros, golosinas, gaseosas, ya no se ven en las escuelas, hora lo que se está promoviendo son alimentos saludables por decirlo de alguna manera, y eso es a lo que se le llama cafetines saludables, frutas, verduras ósea comidas buenas de verdad, nada de comida chatarra, nada de papas, hamburguesas, no tostadas fritas, entonces todo eso entra dentro de todos nosotros. Entonces nosotros tenemos una ficha técnica, en dónde saneamiento ambiental puede ir a las escuelas, e ir a pasa su ficha técnica, estas fichas se mandan al ministerio de salud de la región, y lo unen con educación y ahí ven los resultados de la ficha. Entonces, si todavía se están vendiendo chocolatinas, entonces ahí se puede chequear qué tipo de alimentos se venden. El problema es que, si no se venden dentro, pero se venden fuera, ahí seguimos con el problema, porque la gente de fuera ya ahí no está tan educada, pero por lo menos durante la jornada educativa, al menos durante la mañana y a la tarde, los alimentos ya son más sanos. También hay un programa presidencial, qué es que le dan alimentación a todos los niños, este a toda la escuela digamos, entonces aquí por ejemplo tenemos algunas mamás que, han hecho como comisiones para hacer los alimentos, como darles como un refrigerio, durante la jornada porque, y hay muchos niños que llegan sin desayunar, entonces los alimentos ya son más nutritivos, por ejemplo pupusas pero no pupusas fritas sino que por ejemplo de chiquilín, de mora, de ayote, entonces son cosas que es un nutritivo, aparte del queso y también arroz con leche, o sólo leche, o leche con Cornflakes, todo eso, entonces son alimentos que han ayudado bastante. Hicieron una investigación de sobrepeso y obesidad en niños escolares entonces, se vio que los niños estaban el sobrepeso, y desde ahí se agarró para cambiar todos esos lineamientos para ayudar a bajar las enfermedades no transmisibles en toda la población.

**I: ¿Me podrías explicar que problemas ves tú que tienen los pacientes para la abordar su enfermedad?**

Primero, no tiene la cultura de prevención, la gente cree que no está enferma, entonces se quedan como en la etapa de negación, y muchas veces se les deja solo el trabajo de las pastillas para que trate de hacerlo todo, entonces no hacen dieta, no hacen ejercicio, poner barreras ellos solos, y también ahí es donde vamos, al entorno, los precios muchas veces de las cosas sanas son más caras que las cosas sanas, por ejemplo uno a va a comprar una ensalada, y bueno en primer lugar, aquí nadie te va a vender una ensalada, y si te la venden, siempre es lo mismo lechuga, tomate y poco más, entonces no hay ofertas. Otra cosa es que, por ejemplo, una botella de aceite de Palma cuesta 1.65- 1.70, y es como más de medio litro de aceite, y un bote de aceite de oliva, que son sólo 200 ML, vale como 10 $, entonces el precio ya es demasiado caro. No hay una oferta y demanda de productos que no sean nutritivos y de igual manera, casi que toda la gente cocina todo frito, aquí que casi casi casi todo es frito, frijoles fritos, huevos fritos, las tortillas a veces también las hacen fritas, entonces la cultura del aceite es lo que a nosotros nos dificulta, también incluso la manteca que es aún más dañina, Y es mucho más barata del aceite, y la gente compra por lo accesible, en lo económico que es para ellos. También, a la familia no hace una cultura de prevención para la salud, Sino que como él es el enfermo, él es el que tiene que cambiar, entonces no hay un cambio comportamental familiar, que es de apoyo a la persona enferma, sino que es de “ah como vos es el comes mucho eres vos el que te enfermas”, Como echarle la culpa a alguien, entonces no tenemos eso lastimosamente. Por ejemplo en mi caso, yo lo tengo igualmente, en mi familia, como casi todos los hermanos de mi papá, son diabéticos, bueno es más, ¡todos! son diabéticos, y ya tengo una tía que murió por hacer cetoacidosis diabética, entonces ya los antecedentes en mi familia de diabetes es bien marcado, y también como que ellos también hacen una resistencia, yo como profesionales salud uno sabe que no puede trabajar con su familia, porque a veces no les creen verdad porque necesitan que alguien diferente se lo diga. Y con la comunidad también pasa lo mismo es bien difícil, también ponen barreras que no puedo, que no tengo tiempo, que es lo más fácil hacer. Incluso aquí con los profesionales de salud también le dice uno “oiga y porque no se trae usted su comida” y dicen “y no qué me tengo que levantar más temprano”, entonces es una realidad que existe, y definitivamente que nosotros tenemos que hacer por cambiar nuestra forma de ver cómo son nuestros estilos de vida para también dar a conocer a las demás personas que sí que se puede. Por ejemplo yo siempre he padecido de obesidad y sobrepeso, tenía mi IMC muy alto; entonces desde entonces, yo ya he ido bajando mi peso y eso lo ven las personas, yo lo he hecho a título personal, y les digo que sí se puede, se pueden dejar de tomar refrescos, se pueden dejar de tomar gaseosas, se pueden dejar de comer cosas fritas, Y no necesariamente se tienen que morir de hambre, entonces, yo le explico a la gente con propiedad. Incluso también sirve para motivar a la otra, por ejemplo, mi directora era como yo, que pesaba mucho, y ahora ya las dos hemos bajado. Entonces, muchas veces tenemos que hacer nosotros mismos el cambio para que la gente vea que sí se puede. Y, aún con la gente que sí que puede plantar cositas en su casa, eso nos ayuda a nosotros a conocer el entorno en que puede ayudar a la gente, también si le apoyan a la gente, porque realmente hay mucha gente que, por ejemplo pasan más de dos horas mirando una novela, entonces no hacen ejercicios, porque nadie lo vigila, O alguien quien cree que no lo necesita, entonces el cambio comportamental no se ha hecho totalmente.

Con la actividad física, lo primero, es que el paciente no quiere, porque no le veo el problema si yo no hago ejercicio, segundo, otra barrera que puede existir es que le da pena, que la gente le vea haciendo ejercicio, le da pena que lo vean caminando, o que lo vean corriendo. Otra cosa es, que la gente le hacen bullying, es increíble, pero le hacen bullying, una persona que por ejemplo quiere cambiar sus hábitos de alimentación entonces por ejemplo le dicen” no nos vamos a quedar a cenar porque tú no comes de nada entonces para eso nos hubiéramos quedado en la casa “. Otra barrera, que encuentra la gente, es que aquí no hay espacios, bueno en la zona urbana quizás sí, pero la zona rural no, no hay un espacio donde uno pueda llegar y decir voy a correr a la cancha, O por ejemplo el deporte rey, el fútbol, que es un deporte que sólo lo practican los adolescentes no los adultos mayores, que les puede servir mucho para su ritmo cardíaco bueno todo eso, pero no lo practica, porque eso es de jóvenes. También otra cosa es que te dicen que no tienen tiempo, que tiene que hacer muchas cosas en la casa, y no les queda tiempo para hacerlo; y ahí es donde uno hace hincapié, y le dice “entonces si usted se llega morir quien va hacer todo lo que usted hace en la casa”, entonces ellos te dicen “y si el día tiene 24 horas y 8 me las paso durmiendo que tiempo me queda para hacer todo eso”, entonces poco a poco tiene que ir uno concienciando a la gente de que sí que puede. Les ayudo a ver dónde pueden sacar, aunque sea media hora, se les dan como opciones, por ejemplo, si va a dejar a los niños a la escuela pueden ir caminando en vez de en moto taxi, son 10 minutos que les va a añadir a su esfuerzo físico, entonces uno le va ayudando a ver los espacios donde sí se puede. Otros se cierran totalmente porque te pueden decir estoy mal de las rodillas, o estoy enfermo de los pies, me duele la espalda, me duele la cadera, me duelen las manos, entonces nosotros tenemos una estrategia que se llama *pasaporte saludable*, que es específicamente de las enfermedades no transmisibles, y es una tarjeta que se apunta lo que nosotros llamamos *ejercicio en medicina*, donde se les pasa una prescripción de ejercicios, no de medicamentos. El doctor especialista le va a dar una prescripción de ejercicios, y luego la enfermera le va a explicar cómo tiene que hacer esos ejercicios, y le va llenar el pasaporte, es un librito donde la va a enseñar imágenes de cómo debe hacer los ejercicios si le duelen las rodillas, si le duelen los tobillos, no puede hacer sentado, no puede hacer parado, o viendo tele, digamos la media hora que está viendo tele esa media hora la puede aprovechar para hacer ejercicios. Entonces, eso es lo que se le puede enseñar a la gente, todos los pacientes con enfermedad crónica tienen ese pasaporte, y se les inscribe a un grupo focal y es el grupo que tenemos nosotros para hacer ejercicio medicina, y también se les puede inscribir en el grupo de autoayuda. Este pasaporte se les da a todos los pacientes con enfermedad crónica, pero tiene que haber sido prescrito por el doctor especialista, qué ha identificado que el paciente está descompensado de su azúcar, de su presión, todo eso. También, estamos haciendo coordinaciones con la alcaldía con la unidad de deporte, por qué hay y hay tres chicos haciendo su obra social en la alcaldía, y los tres son de licenciatura en educación física, entonces en cada uno de los ciclos de vida, ellos pueden poner un ejercicio, entonces, ese ejercicio medicina está coordinado con el INDES (Instituto de los deportes de El Salvador), y ellos vienen como delegados para dar ejercicio a la gente que dicen que le duelen los pies, que se cansan demasiado, entonces ellos les ponen ejercicios que sí que pueden hacer en el grupo y que lo puedan hacer en la casa. Entonces, es bien bonito realmente, y es interesante ver como un adulto mayor puedo hacer ejercicio en su casa y no puede decir que “no esto no lo puedo hacer”, no implica ni gasto, ni gasto en ropa de gimnasio, porque eso es lo que no les gusta, porque las mujeres suelen andar habitualmente en falda, entonces ellos, lo que no les gusta es ponerse ropa de ejercicio, o zapatos tenis, porque hay muchas adultas que no se han puesto ya un zapato ejercicio, entonces se les dice “aunque sea en sandalias, puede hacerlo”. Entonces se trata de buscar incentivar a la gente a qué haga ejercicio, entonces se les pone todas las posibilidades para que no puedan decir que no pueden hacerlo.

**Sección 4**: Estrategias en el manejo de pacientes crónico

**I: ¿Que desafíos has encontrado a la hora de la comunicación con el paciente?**

Tal vez en adultos mayores con la cuestión de que ya no miran, porque a lo mejor ya les falla la vista, cosas así. Otra es por el tiempo, hay mucha gente que trabaja entonces ya no les da tiempo de quedarse al grupo, o quedarse a una consejería, o porque son las mujeres que cuidan a niños o a sus propios hijos, entonces eso es lo que les dificulta mucho. Pero, un desafío así de comunicación quizás no hay muchos, y es actitudinal, de parte de la persona hacia la actitud que se tenga con la información o la educación de las enfermedades crónicas.

**I: ¿Cuándo ves tú que un paciente es más receptivo?**

Las mujeres son más receptivas que los hombres, pero los hombres cuando ya se les detecta una enfermedad crónica también cambia su actitud positiva hacia la prevención de complicaciones, solo eso lastimosamente.

Los adolescentes en el tema de no transmisibles quizás no tienen tanto interés, no tienen interés en diabetes o hipertensión, pero sí que tienen mucho interés en el cáncer. Nosotros por ejemplo vamos con un grupo de adolescentes y les preguntamos qué tema les gustaría saber, y siempre es cáncer de mama o cáncer de cérvix, al principio me quedaba “¿Por qué?”, pero si me di cuenta que los adolescentes y el cáncer sí que es una forma de ver las no transmisibles como algo que, si les preocupa, porque son unas enfermedades que por su estilo de vida sí que podían desarrollarse más rápido. También, cuando alguien se muere de una enfermedad no transmisibles en la colonia, o en la comunidad, ya sí que se preocupan. Por ejemplo, cuando alguien se muere de diabetes, todos dicen y qué es eso de la diabetes y como la puedo prevenir, o si alguien muere de cáncer de mama todos empiezan hacerse el examen de mamá, consultar, hacerse la citología. Entonces, lastimosamente, es una educación con mucho miedo y muy directa.

**I ¿Que estrategias de comunicación recomendarías?**

Recomendaría aumentar más el tiempo en una consejería específica a una persona, O poner el requisito de venir con alguien de la familia que la cuida, también eso sería muy bonito, porque alguien que lo cuido también poder escuchar las educaciones que se les da al paciente, y también otra estrategia, es que no solo el paciente partícipe en estos grupos de autoayuda, sino que también la familia, entonces todas aquellas que influyen en las personas o en la enfermedad.

Otra recomendación sería buscar espacios más amplios en las radios locales, o las radios nacionales, y no solo quedarnos en una radio, porque si sólo sale en la radio Nacional, la radio nacional casi nadie la escucha, es más aquí ni se agarra la radio Nacional, sino que se agarran todas las demás, entonces sería buscar por parte del ministerio de salud unos espacios en las radios comerciales, e invertir más en la comunicación: en spots publicitarios, en cuñas radiales, en videos, sí que se hace pero se necesita más. También, más material educativo que sea más explícito para la gente, porque hay veces que el material de lo educativo tiene demasiada letra, cosas muy técnicas, o que no les llame la atención, entonces sí que hay cosas en comunicación para la salud que les puede llamar más la atención, y que lo vea como algo muy importante, y que lo guarde, y que les sirva, no que solo lo lea y luego lo bota.

También más intervenciones educativas en los centros escolares, sabemos que con los cafetines saludables se está haciendo mucho, pero también el profesor tiene que dar ejemplo, y muchos de los mismos profesores son los que se han molestado, y son formas de como influir.

**I: ¿Que apoyos personales, profesionales, y comunidad tienes tu para abordar al enfermo crónico?**

Aquí dentro de los profesionales de salud, nos apoyamos, es un trabajo en equipo. Has ido todo un proceso donde ya se han brindado más apoyo, también se han dado más materiales educativos. Las radios locales también nos dan espacios donde nosotros podemos llegar para dar las formaciones educativas, porque por ser nacionales, no tenemos un fondo de las unidades de salud para hacer estas intervenciones, entonces tenemos que utilizar estos espacios que nos han ayudado mucho. Aquí hay tres radios y en dos nos dejan participar sin cobrarnos.

Otra barrera que identifico, es el poco personal educativo en salud, porque no podemos abordar a varias personas, o grupos de la comunidad, simultáneo. Porque no puedo estar allí o acá, no me puedo partir. Entonces, quizás una debilidad sí que sería eso, falta de personal de educación para la salud en todas las microrredes, porque, así como yo, pasa lo mismo con mis compañeras, somos solo cinco educadoras en todo el departamento de Chalatenango, y somos 33 municipios, y en todo el municipio hay de 5-7 ECOS, entonces hay compañeras que no dan a vasto. Y también, el transporte, el transporte hacia los ECOS, yo muchas veces utilizo mi propio carro, pero eso no es la idea, la idea es que el ministerio nos aporte las herramientas necesarias, entonces el transporte sería como una debilidad que tenemos como ECO especializado.

Una de las cosas con la que yo me siento apoyada, es una buena coordinación con las instituciones de todo el municipio, porque tienen buena apertura hacia el ministerio de salud, y, hacemos coordinaciones con alcaldía, con juzgados, con todas las escuelas, entonces hacemos una buena coordinación para que se desarrollen actividades, como las que he mencionado, porque vamos en muchos temas, pero las enfermedades no transmisibles es lo que nosotros estamos ahorita dando.

Con las otras educadoras fuimos compañeras de estudio, con algunas vaya, porque con otras, y ese es otro problema, no son educadoras, sino que son trabajadora social, licenciaturas en trabajo social, que ellos tienen algo más gerencial, no tanto como choque con la comunidad, sino que algo más gerencial, pero en el camino uno se va moldeando, y viendo que se tiene que hacer, pero todas esas barreras se sobrepasan. Nosotras hacemos, las cinco educadoras, evaluaciones, y también con el SIBASI, la cabeza de aquí, hacemos evaluaciones cada 3 meses, que nos lo marca el ministerio, y también otras reuniones son algunas capacitaciones que vamos todas juntas, ahí nos vemos, y casi todas nos conocemos, y ya sabemos cómo somos cada una, y ahí para ser educador es mucha iniciativa, y, siempre ser muy propositivo, porque yo muchas veces las aliento a ellas, las ayuda a pensar en cosas, por ejemplo si yo hago un mural yo les mando las imágenes, para que ellas lo hagan casi igual que el mío, entonces son cosas pequeñas que uno les ayuda a los demás, entonces a veces incluso los del SIBASI, me llaman para hacer alguna actividad con ellos, pero eso sí que yo siento que depende de cada uno y su personalidad de cada quien. Quizás hay en otros lugares donde al educador le cueste más, pero eso depende de las personalidades, eso ya es actitudinal.

Otro apoyo que encuentro es con las ONG por ejemplo con ayuda en acción, ha estado bastante, y en los años atrás hemos estado con el tema nutrición, entonces como la nutricionista de acá y yo, hemos andado con grupos de mujeres, más que todo porque ayuda en acción uno de sus pilares es el género, entonces en la formación de lideresas, y todo eso, entonces ahí es donde nosotros nos hemos incluido en la salud. Entonces, hemos hecho como una red que todos cumplimos con cada una de las metas entonces por ejemplo nosotros cumplimos con la parte nutricional y ahí se cumplen sus metas, y entonces nosotros acercamos más a la comunidad a ellos, entonces, todo eso nos sirve. También, se está acercando la ONG Provida, que vamos a empezar a trabajar con el tema del agua y saneamiento, entonces es como que al ministerio de salud lo agarran como apoyo para introducirse en la comunidad, entonces ya con diagnóstico, con gente que nosotros ya hemos digitalizado toda la gente que vive en un territorio. Para nosotros el apoyo de ellos es más que todo económico, no es que nos den el dinero sino que es como con ayuda, capacitaciones, actividades conjuntas, actividades de movilización social qué mucha gente se da cuenta de lo que estamos haciendo, ayuda en acción también con capacitación a líderes y lideresas de nuestra comunidad, desarrollar habilidades, hubo una capacitación a promotores de salud que les enseñaban a cómo cocinar con alimentos que ayudan al hierro en las mujeres embarazadas por ejemplo, entonces era desarrollar habilidades con el mismo personal de salud, ellos nos apoyan para eso. Hemos tenido muy buen apoyo por parte de ellos, y a veces donaciones, donaciones de escritorios, gaceteros, de basculas para pesar, basculas de niño, o papelería, entonces todo eso también se hace en coordinación.

**Sección 5**: Experiencias con componentes de intervención en torno a la estrategia de fortalecimiento de la capacidad instalada de las Redes Integrales e Integradas de Salud (RIIS), para mejorar su respuesta, acceso y calidad de la atención a las personas, familias y comunidad, reduciendo así́ las complicaciones y mortalidad de forma prematura causadas por las ENT.

A mí una de las cosas que más me ha fortalecido es las capacitaciones de no transmisibles que se me han dado. También se ha fortalecido mucho la cadena que hay que seguir con el paciente, los tipos de abordaje que también le podemos dar. Bueno, también se ha fortalecido la organización de este programa, porque antes se veía como un paciente más, no se tenía una base de datos, no se tenía un paciente específico para decir ah sí estés diabético, éste no, o cuantos, tener el número, entonces eso ha ayudado mucho en el organigrama, digamos, en la organización de todo el programa. También, capacitaciones que nos dan por ejemplo en tipos de cáncer, de las adicciones, ósea todo eso entra de eso, para ejemplo también donación de sangre que también entra dentro de las no transmisibles. Todo eso sí que ha fortalecido mucho el ministerio, y de igual manera, este con los médicos de familia, y eso ha cambiado mucho el abordaje entonces ya no es una relación médico-paciente sino que entran más temas de consejería, porque ya el médico le dice “mire tiene que bajar de peso o si no se puede llegar a complicar”, entonces todo eso hace que la población vaya asimilando más el cambio, y dándole espacios para consejerías a un médico ya es como que algo muy bueno.

**Para el paciente en riesgo:** esta estrategia ha mejorado en el control del paciente en riesgo, igual el paciente se marchaba sí mayor información, volvía casi igual o peor, entonces eso ha cambiado, y también incluida la familia en todo esto, también. Como digo, ha sido un proceso, no es fácil que la familia se incluya entre las consejerías, olas intervenciones educativas que se hagan, pero si ha sido como en algunos casos muy bueno, incluir a alguien que no es el paciente, es un paso como mucho más, y de igual manera llevar las comunicaciones a la comunidad, incluir estos temas dentro de las posibilidades de trabajo en grupo.

**Insumos y medicamentos**: quizás en insumos de medicamentos si se ha mejorado algo, tal vez en los glucómetros también, antes no existían, cada médico tenía que tener el suyo, pero ahora si los da el ministerio. Los médicos saben más de todas las pruebas del laboratorio. En cuanto al desabastecimiento eso va por temporadas, como por ejemplo si el ministerio no ha hecho la compra, o la burocracia que hay para que llegue el medicamento a lo local. Entonces, es como un periodo que no va a ver el medicamento pero no es así que pase en todo el año, solo por periodos de uno o dos meses, entonces sabemos que en esos meses el paciente tiene que comprar sus medicamentos, entonces ya que alguien compre sus medicamentos es algo que como ya le toca el bolsillo y no les gusta; entonces sí que sé que el sistema de salud de otros países incluyen todo eso, que es el gasto que tiene que hacer uno por su salud, pero aquí como estamos acostumbrados a que todo se nos dé gratis, verdad, mucha gente se queja cuando no hay medicamentos. Pero no solo es eso, hay mucha gente que sí que es muy consciente de ello, y dice que sí que las puede comprar mientas no halla en el ministerio. Las pruebas de laboratorio también han ayudado mucho a la detección y la prevención de complicaciones, y de igual manera, los equipos que se han comprado, que se han asumido, quizás no solo por este programa, pero que ayudan básicamente a tratar este tipo de problemas. También la RIIS, la intercomunicación con hospitales, con las unidades de primer nivel y segundo nivel, entonces todo eso ayuda bastante.

**I: ¿Y en relación al personal?**

En cuanto al personal, quizás no haga falta más personal sino que más capacidades para el propio personal que ya está trabajando, entonces capacitaciones para enfermería, para el médico que antes no se metía, porque antes sólo se veía como curativo curativo curativo curativo, recibir a los pacientes y los pasaba, recibía y los pasaba, ahora no ya tiene como un rol como dentro de la atención al paciente, en donde ya con eso el ejercicio en medicina, ya ellas, las doctoras prescriben el ejercicio hacia esas personas. Es algo positivo. Tal vez no ha aumentado el personal, porque somos los mismos desde hace 8 años, desde los que estuvieron desde el 2010, porque en Julio del 2010 fue la reforma, ya cuando todos se fueron a sus ECOS, pero ya en el 2011 los equipos especializados ya empezaron a funcionar como tal, todo lo que se tiene hasta hoy, muy bueno.

Han aumentado no solo las actividades con enfermedad crónicas para todos los roles profesionales, sino que también el profesional apunta a este cambio.

**I: En caso de actividades de rehabilitación, discapacidad o paliativo**:

Pues en verdad con fisioterapia se está haciendo un poco, se trabaja, pero, así como rehabilitación específica, como un programa tal, no se hace. Por ejemplo, que hacer cuando un paciente se queda ciego por diabetes, o si tiene pie diabético, bueno eso sí que se hace, las curaciones, a las recomendaciones que se le dan; pero realmente un programa como tal para rehabilitar eso si no se tiene, pero las atenciones que se requieren eso si se tiene, por ejemplo, un paciente que no tiene pierna se le lleva un control, igual en otras situaciones, por ejemplo hay pacientes especiales que se les necesita ir a ver a casa porque no se puede mover cosas así, entonces a través de los promotores de salud es allí donde nosotros vamos a entrar.

**I: Que desafíos encuentras a la hora de aplicar protocolos de la RISS:**

Quizás el desafío también por parte de ellos, del paciente, es el tiempo que ellos invierten en la prevención de complicaciones, porque el programa literalmente se refiere a alguien que ya está enfermo, entonces tal vez en eso, y de igual manera, lo que estábamos hablando, el apoyo familiar, qué muchas veces eso es lo que dificulta que ellos apliquen las recomendaciones que se les da, la cultura, Como el miedo a la muerte, es inmediato sólo que le digan que tiene cáncer la gente ya se piensa que se va a morir.

**I: ¿Que Recomendaciones propones?**

Que haya más personal en educación. También pediría más esfuerzos en priorización porque a veces vemos que hay programas que se priorizan más que otros, entonces sabemos que ninguno está sobre otro, y también sería como no igual con un monitoreo constante, sino que se apoye más con material didáctico, material educativo, porque eso sabemos que es el pan de cada día, y nosotros hacemos las coordinaciones para conseguir papel fume, tijeras, todo lo que utiliza.

También dar incentivos a la población cuando participen en estos grupos, cuando ya un paciente a esta en el grupo de autoayuda, por ejemplo, entonces ya un incentivo como una botella de agua, o quizás un par de zapatos, se me ocurre cosas así, entonces son incentivos para los grupos que de verdad funcionen. Todo eso nosotros lo podemos gestionar porque realmente dentro de las unidades, pero no solo las enfermedades crónicas son las que necesitan gestión, sino que todos los programas. Todo eso es bien complicado.

También, políticas públicas locales para que se puedan desarrollar ejercicio, para que la gente aprovecha los espacios que tienen libres, fines de semana, después del trabajo, porque aquí casi toda la gente trabaja de ocho a cuatro, bueno nosotros de 7:30 a 15:30, pero realmente nosotros no salimos nunca a la hora, entonces tenemos que buscar espacios que verdaderamente nos beneficia nosotros, y eso intentamos. Por ejemplo, ahora que se están dando ejercicios de aeróbicos a las seis de la tarde, en la casa comunal, siempre en coordinación con la alcaldía de la palma y nosotros, entonces con eso de las enfermedades no transmisibles se está haciendo todo eso, pero necesitamos difusión, que la gente lo conozca, que lo sepa, entonces comunicación de todo eso.

El tema de enfermedades no transmisibles es grande, complejo, porque mucha gente se basa en lo que ya hacíamos, verdad de chiquitos, o desde los abuelos, entonces, quizás apuntarles más en lo cultural, en masa, la comunicación en masa, porque nosotros lo hacemos aquí pero local, pero se necesita en masa porque si no trabajamos en masa, a mucha gente no le llega la educación a donde esta, verdad, entonces si nosotros tuviéramos así una educación más grande, y más comprometida, a bueno, esa es otra recomendación para el ministerio, mayor compromiso.

Es muy bueno que ustedes vengan acá para que conozcan un poco más de la realidad

## Staff interview 6: Sanitary inspector

Entrevista con: Coordinador Gestión medioambiental-saneamiento

Fecha: 13/06/18
Lugar de la entrevista: Unidad Comunitaria de Salud Familiar Jiquilisco
Código del informante: SFML0005
Genero del informante: F
Nombre del entrevistador: ML

**Sección 1**: Perfil del participante y función en la prestación del servicio del primer nivel de atención

**I: Historial de cuándo empezó a trabajar como inspectora de saneamiento y porqué empezó a trabajar en esta área**

Buenos días, mi nombre es xxxx y soy inspectora de saneamiento desde el año 2011, pero llevo hasta el momento 22 años laborando porque anteriormente trabajaba como promotora de salud en esta misma zona. Aquí me inicie trabajando como promotora y simplemente cuando hubo la reforma de salud me cambiaron la plaza, porque se vio la necesidad de que tenía que haber un inspector en la zona.

Mientras fui promotora de salud, atendía solamente dos caseríos del cantón zamorano. En la zona solamente había tres promotores que cubríamos desde aquí de la zona de Zamorano hasta la isla de Méndez. Luego con la reforma ya cubrieron toda la zona con promotores de salud y fue ahí cuando me cambiaron la plaza.

El trabajo es bien diferente porque cuando se trabaja como promotor de salud la atención es directamente con la persona en la visita domiciliar, los acercamientos y el programa de maternidad infantil, y lo que es el niño y la mujer. Es poco lo que se ve desde el ambiente, la zona ambiental se ve, pero es mínimo. Ya en mi trabajo actualmente como promotor de saneamiento ya no es con la persona directamente, ya mi trabajo es, específicamente, la vigilancia del agua de consumo, los alimentos, basura, electrificación… es decir el ambiente alrededor de las casas, y se hace una programación anual, trabajamos en base a metas. Tengo programado hacer la vigilancia del agua todos los exámenes que el Ministerio de Salud actualiza y todo lo que ellos mandan, yo simplemente lo traslado. En cuanto a la vigilancia de los alimentos se vigilan desde los chalets escolares hasta los comedores o restaurantes que existan en la zona. Si hay denuncias de personas por cualquier cosa, sea contaminación ambiental, también se hace seguimiento hasta encontrar una solución. A veces se encuentra uno con problemas que tiene que buscar y coordinar con otra institución que estén dentro como el Ministerio de Medioambiente, el Ministerio de Agricultura y Ganadería, y si el problema se sale de la salud le compete a otro. Se hace la coordinación. Y el SIBASI que esta siempre para apoyar, si está el SIBASI y luego la región. Mas que todo un inspector de saneamiento ve el área urbana de un municipio; en el área rural solo vemos dos en municipios en el área de Usulután, uno que está en Puerto Parado, y mi persona que esta la zona de Bajo Lempa

**I: APROXIMADAMENTE QUE TERRITORIO CUBRE USTED EN BAJO LEMPA**

El área que cubro es desde aquí en el Zamorano hasta la isla de Méndez, veo Coliguay, pantano canoa, isla de Méndez y Zamorano acá en la intermedia.

**I: CÓMO SE REALIZA EL ANÁLISIS DE AGUA**

La vigilancia del agua va desde investigar si el agua trae cloro, y la lectura del cloro se hace a diario. Se buscan unos puntos específicos, a mediación de donde pase el sistema o al final de donde cubre el sistema. Aquí en el sistema que tenemos el último caserío que cubre es el caserío de las mesitas del Cantón la Canoa. Más abajo está también el Babilonia, pero casi están en la misma entonces uno toma una lectura al final, se hace otra intermedia, para ver si el cloro alcanza a llegar a la última vivienda, y eso se registra a diario. Las muestras de agua van desde el físico-químico, traza de metales, bacteriológicas… unas van a San Salvador, al laboratorio Central, y para las demás hacen las lecturas aquí en San Miguel, pero de las bacteriológicas tengo programadas veinticuatro al año, llevo dos mensuales. De las físico-químicas solo tengo una en el año que lo tengo programado para agosto de este año. De traza de metales solamente tengo una para enero y gracias a dios salió dentro de norma. Es una vigilancia constante al sistema de agua. Este sistema de agua funciona desde el año 2010 y desde que se fundó se ha venido haciendo un seguimiento, y si hay algunas cuestiones como también se le hace vigilancia al tanque donde está el pozo (si el tanque está limpio, si cumple los requisitos, si la persona que está a cargo tiene sus exámenes correspondientes…). Además, hay una programación de reuniones con la junta que lo administra, se les presenta a ellos si alguna muestra salió fuera de norma, si hay algún problema en el tanque que hay que solucionarlo, ahí es parte de la misma vigilancia.

**I: hay muchas zonas que se quedan desprovistas de esta agua potable**

Nuestro sistema cubre alrededor de 1000-1050 viviendas que si tenemos descubierto es bien mínimo, pero la vivienda que no tiene agua potable directamente del sistema, puede ir al trayéndola directamente del vecino, de modo que consume algo del sistema porque el agua subterránea está altamente contaminada y no es apta para el consumo humano. De todas formas, tenemos Isla de Méndez que no tiene agua apta para el consumo humano, ANDA que es el ente Rector del Salvador del agua, perforó un pozo para poder darle agua a Isla de Méndez, pero hay un problema con el agua siempre que se hacen muestras salen fuera de norma porque llevan heces fecales, sale alguna turbidez del agua, un olor, entonces la persona lo rechaza. La mayoría se abastecen de agua de pozo perforado, aguas contaminadas, de las cuales se han cogido muestras y se ha demostrado que está altamente contaminado.

Además, se realizó un estudio por parte del Instituto de la Salud del Ministerio, con esto de la enfermedad renal donde vinieron unos médicos cubanos, se investigó el agua del río Lempa, del río que cruza por Nueva Esperanza, las aguas de la bahía, de los estanques camaroneros, los pozos de las parcelas y de las viviendas… luego se les entregó unos documentos que en la mayoría de esos lugares que se muestrearon tienen metales pesados, tienen agrotóxicos, que no son aptos para el consumo humano. El río Lempa tiene menos porque es agua que corre, pero no así el agua subterránea de los pozos perforados o de los pozos artesanales, que están altamente contaminados y no es apto para el consumo humano.

El agua del sistema sigue unos requisitos que tenemos actualmente administrado por la comunidad, porque el pozo no está dentro de esta zona, el pozo está yendo por San Marcos y desde allá nos abastecen acá. Se les han hecho todos los análisis y nunca ha salido los metales pesados.... Más tarde se hizo otro estudio que nos programaron a nosotros tomar muestras de plaguicidas y buscarlas en el agua. A nivel del país, no solo fuimos nosotros, y salió el agua con *paraquat* y como no se prohibió la venta, se sigue aplicando al suelo, nunca desaparece, y por muy profunda que el agua esté siempre le llega y está ahí presente. Como salud, decirle a la población “mire el agua tiene paraquat”, pero cuál es la solución que tenemos. Hacerlo desaparecer del agua es algo difícil, el Ministerio no encontró una forma. Los parámetros pueden cambiar, quizás en cantidad puede tener más un año según la cantidad de tóxicos que apliques al suelo. Recordemos que no solo se aplica en esta zona, la zona de filtración del recurso hídrico (la cuenca) está en la parte norte, y esa no está dentro del sistema Se ha tratado de que cerca del sistema donde estamos nosotros no haya cultivos de ningún tipo, para evitar que se aplique herbicidas o algo tóxico, y en un futuro forestarlo, para que esté protegido. De momento esté sistema no tiene viviendas alrededor y tratamos de que esté al menos protegido de agroquímicos. No hemos tenido otro problema. Para este año no me programaron plaguicidas, porque con los dos estudios que se hicieron, se vieron que hay el mismo paraquat, de los otros no apareció; porque les hacen cinco análisis, y siempre el paraquat está ahí.

Al ver esto, el Ministerio de Salud decide coordinar con la Junta de Agua para ver que los sistemas se mejoran, verificar si hay fugas, corregirlas de inmediato, se les exigen que haya un plan de seguridad del agua, ya que por exigencia deben de tenerlo ellos, y ver donde está los puntos críticos, en qué puntos el agua se puede contaminar. Aquí también sufrimos inundaciones cuando el río se rebalsa y se produce un arrastre de materia fecal, y va al subsuelo. Si hay una fuga de agua, y el agua se contamina, nos damos cuenta de inmediato porque mensualmente se están enviando las muestras, y por el momento, gracias a dios, las muestras no están saliendo contaminadas, y podemos tener una seguridad en el agua que estamos tomando

**I: tal como comentas, aún coexiste un gran número de zonas que están fuera del sistema, y que por tanto están consumiendo de ese agua. ¿quién se encarga de realizar los controles del consumo de esa agua?**

Aparte de ANDA, estamos nosotros como Ministerio de Salud haciendo correcciones y sugerencias que hay que hacer para mejorar este sistema, pero no es fácil tratar con ANDA a nivel nacional puesto que ellos se dirigen por un director que los dirige. No es lo mismo tratar con una junta administradora que tan solo administra un sistema, que tratar con un ente Rector a nivel nacional. A ellos se les ha exigido en el sistema de Méndez un plan de seguridad. Han hecho algunas correcciones, por ejemplo, lavar y desinfectar el tanque, mantenerlo tapado, hacer la vigilancia de la lectura de cloro, pero si no da cloro en esas viviendas es por la misma turbidez del agua, no hay cloro residual. Entonces ellos han venido haciendo unas correcciones, pero ellos no pueden realmente quitar la turbidez del agua, ni la contaminación del paraquat.

Además, en la zona de Isla de Méndez, está muy cerca del mar, por lo que conseguir agua dulce para poder abastecer es más difícil. Pero aun así encontraron ese pozo donde el agua cumple en algunos parámetros, pero en otros no, no obstante, siempre se le hacen las recomendaciones al personal de salud.

**I: ¿QUÉ agua consume la gente de esa zona entonces?**

El agua que consumen es el agua de un pozo perforado. Se le llama una puntera ya que colocan un tubo a 10-12 metros de profundidad, pero a esa profundidad el agua siempre sale contaminada con bacterias *escherichia cola*, heces fecales, porque hay un arrastre por el sistema de filtración. Se les ha dicho que a través de clorar el agua o filtrarla (en esto también participa la Cruz Roja, donde hizo una donación de filtros caseros para que las personas pudieran purificar su agua y consumirla a través de un filtro), pero un filtro también requiere su mantenimiento, las personas dejan que se deteriore, y terminan tomando el agua cruda desde un pozo.

Hasta el momento, no hay brotes tan seguidos de enfermedades hídricas porque como que el cuerpo se adapta y se acostumbra a la contaminación y no tiene consecuencias en la persona, aunque parasitismo siempre hay.

**I: ¿las personas que consumen ese saben que lo que beben está contaminado?**

Ellos saben que está contaminado, pero alegan que no han tenido otra opción y que en todo en el tiempo de su vida han tomado esa agua y nunca han tenido síntomas. Y al ver, si relacionamos este problema del agua con la insuficiencia renal, no observamos que el agua sea el culpable, ya que si comparamos Isla de Méndez con los casos que tienen en comparación con el Zamorano, hay una gran diferencia, ya que en la Isla de Méndez, existen otros problemas de salud, las personas padecen más de ácido úrico por el marisco que consumen, pero no el nivel de insuficiencia renal como tenemos nosotros porque nosotros hemos tenido más muertos que ellos que no tienen ninguna persona dializándose, si tienen estadios, pero como en otras zonas como la zona alta, pero si la comparamos con nosotros, tenemos más muertos; aquí en ciudad romero, luego seguido por Nueva Esperanza, también en nuevo amanecer, pero donde se concentran más es aquí en ciudad romero. Nosotros el primer caso que tuvimos por el 97’-98’, una señora que falleció, y hay empezaron a aparecer los casos, pero el Ministerio no tenía como un programa así del estudio de la enfermedad, entonces moría la persona y se decía que era muerte súbita, luego se vino viendo que la enfermedad renal, entonces se hizo un escándalo, luego el fondo de emergencias vio la necesidad de investigar a la población, y se hizo más que todo con adultos, a empezar a investigar, a hacer un tamizaje, a través de un laboratorio privado y empezaron a ver que aparecieron más y más personas porque uno ya estaba en estadios para diálisis, y otros que morían para dializarse, y hemos tenido bastantes fallecidos acá, y yo siempre me pregunto porque el resto no tienen tantos casos, entonces no es el agua, que está más contaminada y han vivido toda la vida allá.

También vemos las culturas o las formas de alimentarse, de qué trabaja esa población en las islas y qué hace la población nuestra. Allá trabajan más en la pesca, mientras que aquí se trabaja más en la agricultura. Allá consumen más marisco mientras que la población de acá es una comunidad que vino repatriada de Panamá y con culturas diferentes, consumen más comida enlatada, hay más consumo de arroz, harina, están más expuestos al estrés térmico. Si vemos la parte de las islas, hay una disparidad en la alimentación porque no es la misma cultura. Se investigó con el estudio que se hizo, donde vinieron los médicos cubanos, se hizo un estudio del cabello, la alimentación, los resultados que dio el agua, hicieron biopsias, pero finalmente no tenemos un resultado de qué es lo que está ocasionando el problema o el daño renal. Cuando hablamos del cloro con la población, nos dicen que no quieren que cloremos el agua porque el cloro daña los riñones, pero eso es un decir porque tampoco es el cloro. El cloro es un residual que quedó después del efecto, y hay una norma salvadoreña que nos rige la cantidad de cloro que debe de tener lo permitido y lo no permitido, entonces no va por ahí. No obstante, se sigue trabajando y se descubriendo más pacientes, y vemos que es a nivel nacional, pero con la mayor población en la zona costera, pero no en los caseríos que están entre la bahía y el mar. Cuando nos reunimos y nos dicen acerca de la referencia del paciente renal en la Isla de Méndez, solo son algunos, más bien pocos, pero nosotros hemos tenido dializándose. Allí tienes pacientes con estadios que se mantienen, pero acá por ejemplo en la canoa hemos tenido un niño de los 12 años dializándose, y ahora tiene 20 con hemodiálisis y se mantiene, pero aquí también hemos tenido uno de 24 con hemodiálisis que murió, y han investigado desde el embarazo, se le hizo un estudio bien completo, pero no se ha podido hallar la causa. Este estudio lo hizo el Doctor Orantes, que está a nivel central, con apoyo de médicos de la OPS, cubanos, después de que tenía un listado de pacientes de la zona norte y la zona sur del Fondo de Emergencia, que trabajaba la parte de tierra blanca, ya que tiene una buena cantidad de pacientes renales, unos que ya fallecieron, otros que están en proceso, otros están en diálisis, y otros que van ahí con estadio avanzado. Por eso es bien contradictorio

**I: ¿qué perfil de población reside aquí y allá?**

El perfil es parecido. En la tierra del norte, en Tierra Blanca, hay agricultura y ganadería, y aquí es igual.

No obstante, la zona de Tierra Blanca está más alta, y nosotros estamos en la parte más baja, y los pacientes con enfermedad renal casi andan en las mismas proporciones. Si comparamos el agua, ellos no han tomado agua tan contaminada como la que tomamos nosotros, han tomado más agua apta para el consumo humano, porque han tenido sistemas de ANDA, desde antes, por eso me da la idea de que no es una cuestión del agua. Luego les echan a los plaguicidas, ahí es donde me pregunto en la zona alta del departamento de Copán, Santiago de María, viven mis papas, y mi papa tiene 77años, e igual en ganadería no ha trabajado, pero en agricultura toda su vida, utilizando agroquímicos, no con la protección que ahora se recomienda, pero tampoco él ha caído en problemas renales. ¿Dónde está la causa entonces? ¿Serán los medicamentos, la alimentación o son otras enfermedades crónicas que llegan a la persona? como la diabetes o la hipertensión, pero también hay personas que no han caído, que no son hipertensas ni tampoco diabéticas y tienen el problema renal. Hay otras que dicen que la persona es alcohólica o fuman, pero nosotros tenemos personas que han sido alcohólicas toda su vida y todavía están ahí, tal vez ya tienen un daño, pero hemos tenido gente joven que no han tomado nunca y han fallecido por el daño renal.

**I: Entonces cuando se tratan de causas “no tradicionales” encontramos el problema en el agua, por otro lado, está la comparativa de la isla de méndez y la de acá, donde allá el agua está altamente contaminada y es consumida por personas sin enfermedad renal como el que hay acá. además, está el problema de la temperatura, donde encontramos más casos en las zonas más elevadas. finalmente, no ha habido protección en ningún lado.**

Puede ser, nosotros pensamos que no puede ser algo genético, porque no se viene heredando un problema renal, cuando estudiaron a la persona embarazada y al niño, y no dio resultado, el niño nace sano. Según va creciendo puede desarrollar este problema, porque también se hizo un estudio en un muchacho en Sisiguayo que vimos que tenía niveles altos de creatinina en otros exámenes, y luego le pusieron en tratamiento, pero el muchacho se fue a EEUU y ahí está trabajando, por ahora no sé qué habrá pasado con él. Luego hemos tenido casos donde algunos pocos de Sisiguayo que están en la salina, pero nunca se compara con el número de nosotros que estamos acá en el Zamorano y Tierra Blanca.

Una vez, como se corrió la noticia de que en Bajo Lempa tenían el problema más grande insuficiencia renal, me llamaron a mí en San Miguel y me preguntan: ¿cuántos casos tenéis con DPCA? En ese momento yo les dije: tengo dos, y me responde: hay un caserío de San Miguel que tiene treinta y cinco dializándose en casa, unos que los cubre el seguro social y otros que los cubre salud pública, y ¿Por qué tantos?, si estamos ya hablando de zona norte de San Miguel, en Moncagua, y dice el compañero: estoy colapsado por los desechos que tengo de los pacientes que se dializan porque de treinta y cinco han bajado a veintiocho porque los otros han fallecido. Esa gran cantidad si los comparamos con el Bajo Lempa, si tenemos a pacientes en diferentes estadios, pero se mantienen, nunca hemos tenido esa cantidad que Moncagua tiene, y si tenemos pacientes que se niegan a la diálisis, y ha habido momento que parece que van a fallecer, y luego se recuperan y vienen con Creatinina que la bajo a 10, luego a 7 y de ahí se mantienen. Y de los que tenemos, dos tienen DPCA y uno con hemodiálisis, se mantienen. Mi pregunta ahora es porqué en los diferentes lugares del país se da el problema en la misma población y en las mismas edades [entre 19 y 55-60 años]. También vemos el dato entre género masculino y femenino, siendo la población femenina aquella con un menor daño renal, siempre el dato más alto se tiene en el hombre.

**I: En mujeres tampoco tenéis casos por causas que no sean tradicionales, es decir, la mayoría de los casos vienen por una diabetes o por casos de una hipertensión**

Algunas hay. Sí notamos en la consulta cuando da el nefrólogo, dos o tres mujeres que están en control, pero la mayoría son hombres. Estas mujeres son las esposas que trasladan, que van a dejar alimentos a la parcela, que están siempre en contacto con el trabajo del esposo o han trabajado con ellos, pero no podemos decir que esta es la causa, ya sea agroquímicos, recursos hídricos… porque no encontramos el patrón. Siempre se ha visto que en la mujer es menos, donde fallece el hombre y la mujer está sana. Tenemos varios en Ciudad Romero, donde que yo recuerde, acá han muerto dos señoras en sesenta y sesenta y dos años, y los señores ahí están. Por ejemplo, tenemos un hombre paciente que no ve ya casi, y que no tiene daño renal, y trabajo en negocios, vivió parte de su vida en Panamá, y luego acá, y acá falleció la esposa de enfermedad renal, pero no él no ha sido paciente renal.

Mucha gente durante la guerra se fue a panamá, y de ahí que paso se repatriaron acá, pero no volvieron a la Unión, que era donde nacieron, sino que quisieron venir a la costa, porque allá no tenían terrenos para trabajar en la agricultura. Ellos querían trabajar en ganadería y agricultura, y aquí se daba la oportunidad, al ser la zona donde más se trabaja la agricultura y la ganadería en el país. La población quiso venir para acá.

Digo yo, en la zona norte, donde es más fresco el clima, no tiene ese problema que tenemos nosotros, en los municipios de San Agustín, San Francisco, El Triunfo. En el puerto de El Triunfo, dice el nefrólogo que no tenemos el problema que tenemos acá porque allá el problema es por consumo de marisco, ya que puede subirse el potasio, a veces el sodio, ácido úrico… Pero allá es por consumo de marisco.

Yo le pregunto a mis compañeros de San Agustín, San Francisco… solo uno tiene dializándose. Y siempre me enfocan en Ciudad Romero, que es el que más casos ha tenido. No sé si es porque la población es más grande o es porque está más concentrado. Me queda esa pregunta de porque aquí hay más. Se nos ha muerto gente joven, mujeres que, como le digo, solo recuerdo dos, y la mayoría hombres, entre veinte a sesenta años.

**I: Cuál considera usted la causa/s más determinante?**

A veces pienso que en el hombre hay otros factores, se desvela más, el consumo de tabaco, la deshidratación es más frecuente en él, porque es él quien realiza el trabajo de campo, también hay alcoholismo en la mayoría, el tipo de alcohol que consumen, es decir está más expuesto.

**I: ¿Estas mismas características que describes también se darían en san miguel?**

San Miguel tiene un lugar, aparte de Moncagua, yendo a Morazán, que también le llegaron a hacer un estudio de acá porque presentaba bastantes pacientes, aunque el regional de San Miguel tiene allí el centro de Diálisis y Hemodiálisis, y decidieron hacer el estudio ahí, no supe si investigaron la causa del problema renal en ese lugar, porque también en San Luis Talpa varios muertos, y sigue habiendo insuficiencia renal. Decían que porque estaba la contaminación de una fábrica de baterías Récord y la quitaron y bueno. Será que le encontraron la causa que eso era, ¿será que el problema se detuvo o siguen apareciendo más? Pero en diferentes puntos del país se dan como brotes de enfermedad renal.

Santiago de María que es un lugar fresco, presentó un caso de una joven de veintiún años, y dicen que el problema es a nivel nacional, pero no es en la cantidad que tenemos nosotros. Es cuando yo digo que es por la deshidratación que el cuerpo sufre. Porque cuando el niño está dormido, ya sea por la noche y en el día, el niño se está deshidratando, porque cuando se despierte y se levante, el niño está bien sudado, estando dormido, y desde ese momento el niño está perdiendo electrolitos de su cuerpo, que no los recupera con el agua que uno le da. Esto de niño, luego está la actividad física. El joven hace deporte, pero no se hidrata, y en las actividades de deporte pierde muchos líquidos porque siempre está expuesto a sudoración.

**I: cuando se dan estos en los pacientes, ¿preguntáis acerca de la cantidad de agua que beben?**

Es una pregunta que siempre se le hace a la persona, ¿qué cantidad de agua consume?, y es ahí donde nos encontramos con eso de que no se hidrata. Además de eso, culpaban la coca cola, pero científicamente no se ha descubierto que la coca cola genere un daño renal.

También tuvimos un caso de un hombre que murió a los treinta y tres años, consumía el litro más grande de coca cola con comida chatarra, y agua no llevaba en su trabajo. Cuando cayó, cayó de un solo.

**I: Si en esta zona de ciudad romero, vosotros habéis visto que una persona que consuma poco agua en comparación con otra persona que consuma más agua, ¿los dos sufren de enfermedad renal?**

Se ha visto casos de que personas que se cuidan, que se hidratan, no presentan así tan luego la enfermedad, o sea lo mantiene cuando ya le dijeron que su problema renal va en un avance, empiezan y le prestan atención al médico y comienzan a tomarse los medicamentos que se le dio y a hidratarse, no solo con agua, sino con sueros, y se ve que la persona mejoró. Pero no así, hemos tenido dos hombres que al mismo tiempo lo descubrieron que tenían presión alta, no diabetes, y en ese momento le dijo el médico: va a comenzar a tomar este medicamento como un nefroprotector, y dijo uno de ellos: “yo no me voy a tomar ese medicamento, yo no me siento mal, yo me siento bien”. El otro sí comenzó tal como el médico se lo indicó. El que no se tomó el medicamento y se dejó guiar por lo que el sentía, ahora está en hemodiálisis, después de que estuvo en diálisis, y está totalmente deteriorado. La otra persona, hasta la vez, es hipertenso, pero no tiene un daño renal.

**I: Por lo que estás contando, la contaminación de paraquat está presente. lo que pasa que aquellas personas que beben agua, consiguen de alguna manera desechar ese agrotóxico en gran medida porque se hidratan y no consiguen avanzar, además si lo juntan con un nefro protector. está claro que aquellos que no cambian su hábitat de agua, son los que acaban llegando a la diálisis o un daño renal crónico**

Si ellos escuchan la consejería del médico y cambian su forma de alimentarse, son personas que viven con un daño que se mantiene, que no llegan a la diálisis, y es por eso que vieron la necesidad de que fueran tratados por la psicóloga y por la nutricionista, porque la alimentación es básica, y también la orientación de la psicóloga, más que todas las personas que se niegan a la diálisis, que para convencerlos hemos tenido que traer a la persona que se está dializando en ese momento para que puedan ver una orientación, para que le explique que lo que va a hacer no es sentirse de que ya se va a morir, sino que puede seguir viviendo, pero si se puede evitar llegar a la diálisis, eso es lo que se pretende en salud, que esta persona no llegue a la diálisis.

Yo llegué a esta zona en 1994, yo vengo de ser originaria de la zona norte. Al llegar acá, es un clima diferente, y yo digo si yo también consumí la misma agua, y un tiempo trabajé en la agricultura en esta misma zona, pero claro protegiéndome, y no muy expuesta a los químicos, y siempre cada año yo me hago el tamizaje, y se lo hago también a mi hijo casa año, mi hijo nació aquí, tiene 23 años y no presenta ningún daño, tiene parámetros normales, y yo igual.

Siempre nosotros tenemos el cuidado, y la orientación a la familia, el consumo de agua. Cuando estamos muy expuestos al estrés térmico, decidimos consumir suero, y eso es lo que le hacemos ver nosotros a la población, a las jugadoras yo les digo, en vez de traer a la cancha una soda, una botella de agua, debemos tener un suero preparado o un agua de coco, que eso nos va hidratar y nos va a reponer lo que se pierde en la cancha de fútbol a la hora de jugar, porque sí se suda y las temperaturas a las tres de la tarde son fuertes. Pero llegar a concienciar a esa población no es fácil. Por ejemplo, mi hijo es consciente de que si yo le llevo suero él se los prepara y se lo toma, porque viene de una jornada de campo. Él estudia agroecología, y sabe que él se tiene que hidratar, si se deshidrata él tiene que reponerse de lo que ha perdido. Pero eso es como educar a la familia. Concienciar a esta población joven, yo siento que estamos hablando con personas adultas, cambiar a un adulto es bien difícil, pero si estamos entrando desde las escuelas, desde la niñez, y esa es la parte educativa que tiene que llevar el personal de campo, los promotores de salud en las charlas en las escuelas, pero en el plan no se dice que se trabaje con la población estudiantil, y esa es la población que pretendemos un día cambiar, porque al adulto no le podemos cambiar porque lo que nos dice es “toda la vida he trabajado en el campo, ahorita me vienen a decir que ese trabajo a mí me daña, o que tome esto, que tome lo otro”, asique cambiarle de idea no es tan fácil.

**I: Ahora hablaremos del tema de la juventud. pero antes de nada en ciudad de méndez, el agua está contaminada y también se está en contacto con el paraquat. ¿porqué crees que no se dan tantos casos ahí?**

Esta población se la pasan jóvenes y hombres adultos metidos en el mar y en la bahía por la actividad de pesca, y los que no tienen otra actividad, pero no es agricultura, sino en la ganadería, pero son pocos, la mayoría son trabajos de pesca. Y son jornadas que entran siempre de noche, de día es poca la población que está en la actividad de la pesca. Ellos entran de noche, y de noche el clima es diferente. Puede ser ese el motivo por el cual no entran en ese estado de deshidratación. Y en el día lo pasa uno en su casa o preparando el material.

**I: Volviendo al tema de la prevención. ¿qué barreras encuentra usted de su trabajo para realizar esta tarea de prevención?**

Como mi trabajo no es directamente con la persona, sino con el medioambiente, pero siempre se hacen coordinaciones, la barrera más grande es la credibilidad de la gente a la enfermedad actual por la que está pasando. No toda la gente es consciente de que la prevención es lo más importante antes de llegar a la enfermedad, siempre han pensado de que salud es curativo, buscar el medio para curarse, pero nunca nos buscan para prevenir, y nosotros el trabajo de campo es más preventivo, enseñar a la persona a no llegar, si es posible, evitar que tenga contacto con el hospital, con la unidad de salud, si se puede prevenir en casa. Pero hay una barrera de las culturas, el problema es cultural. Cuando la persona es adulta y nos dice: toda mi vida he vivido como estoy, he pasado una guerra, tome agua de un riachuelo que pasó ahí un agua contaminada, y aquí estoy, no me pasó nada. Cuando nosotros vemos eso como que sentimos que no nos deja pasar más allá para poder alcanzar un cambio de conducta en la persona. Vemos que nos quedamos cortos y no podemos avanzar, porque el adulto no le da la importancia, a que el joven participe en grupos de autoayuda, que el joven participe en actividades recreativas, que piense que para él la salud es importante, que es su salud y que la debe de cuidar, y eso es lo que nosotros no hemos podido lograr, que la persona esté consciente, y nos cuesta entrar en ese círculo que tiene la persona, como que se cierra a escucharlo. Simplemente como es un proceso, plasmamos otra fecha de visita y vamos de forma continua porque siempre hay una programación para llegar a esas viviendas. No es solo seguimiento a la persona enferma, también está la población sana y es ahí donde a uno le interesa llegar para que esta persona sana no llegue al problema renal, y es un trabajo en equipo: la enfermería, el médico, el promotor, saneamiento ambiental… porque cada uno tiene su área que mover. Se trabaja en equipo porque el resultado es mejor. Ahí uno se sienta junta todas las partes que vio.

Tuvimos un caso ayer de un jovencito (de menos de 18 años), el doctor lo comentó, colapsó en la cooperativa, se desmayó trabajando allá. Lo trajeron, lo vio el nefrólogo. Lo primero que le preguntó fue ¿tomas agua?, poco, le respondió, ¿qué cantidad le preguntó?, no te sé decir, dijo. ¿Cuántas horas trabajas? ¿Estudias?, horario de mañana y de tarde, no estudio, responde el joven. Trabajó todo el día, se le bajó el potasio. El doctor dijo, él está totalmente deshidratado. Le colocaron suero en ese momento.

Como le digo, mi parte no va hacia la persona, le hicieron los análisis, le mandaron un test de riñones, ahí desconozco ya como salió, porque eso ya es parte del médico, pero aun lo tienen el control, y ya no va a la cooperativa, o sea, sería recaer, si él lo tiene control es porque ya encontró un daño a temprana edad, pero desde cuándo se vienen poniendo estas temperaturas, y es de familia porque la mama, el papa… todos van a la cooperativa. Y no es que sea la cooperativa, porque puede ser hoy en la cooperativa, pero mañana va a ser en el terreno de ellos, en la parcela de ellos, y es la misma deshidratación, mal alimentados.

Aquí hay otro factor que yo lo analizo, no sé si el nefrólogo lo habrá analizado, aquí la alimentación en la persona es un horario que no. En el desayuno solo toman café y un pedacito de pan. Te estoy hablando de horario entre seis y media – siete. Viene a comer algo ya sólido tipo nueve – diez de la mañana. Y si esta persona anda en el campo trabajando, solo con un café y un pedacito de pan, el cuerpo no recibió nada para esa exposición donde va a trabajar. Cuando come a las nueve de la mañana, qué tipos de alimentos come. Él está comiendo más harina porque es arroz, esta población come mucho arroz, cultura de Panamá. Más arroz, o sea, las harinas prevalecen en la población. El almuerzo lo está comiendo tipo una. Tipo nueve – diez come una porción de arroz grande, también puede ser un huevo, pero casi siempre es atún de la lata o una sardina, ellos lo que más consumen son enlatados. Un atún y una sardina en el arroz. Rara vez, sí tienen pescado, pero más para la cena, pero es la persona que tiene acceso a un pescado, pero los demás puede ser frijol y el arroz, o puede ser un atún o una sardina.

Pero para mí, lo esencial es el desayuno que ellos lo hacen mal. Yo les digo, debe de tomar un desayuno normal, una o dos tortillas, lo que usted se vaya a comer, y a lo que comes agrégale frijoles, huevos o productos lácteos. Así usted come balanceado y se va al campo. Con eso usted aguanta hasta las diez/once y una merienda o algo, un refrigero y a las doce/doce y media usted almuerza. Pero esa parte de la alimentación usted es bien mala, y es una costumbre de ellos, y me dicen ellos fíjese que es que a mí no me da hambre en la mañana. Pero es cultural.

Mi hijo cuando sale, él se va a las cinco y media y mi hijo desayuna a esa hora, yo le digo cómase algo, se toma una avena con leche o lo que sea, pero pan no, ni pan francés ni pan dulce, mejor cómase una tortilla porque es cultural de nosotros y se va desayunado. Pero a mí no me parece problema. Y ya está en un camino de adaptación, pero no se va a ir a trabajar sin desayunar. Esa parte de alimentación acá, a mí me parece un desequilibrio que ellos tienen.

**I: Y en los comedores que usted visita, ¿cree que hay algún riesgo con la alimentación?**

No, en los comedores acá se exhibe comida de la cultura que nosotros manejamos, hay comidas variadas: carnes, marisco… de lo que la persona desee consumir. Pero como en estos lugares también se debe hacer vigilancia a la comida que está implementada en los centros escolares. Qué se le está dando al niño en la escuela. Pero como es la costumbre de acá que el niño no desayune en la mañana, no llega desayunado. En la escuela le están dando leche, que es bien nutritivo para el niño, cereales, el arroz que lo cocinan, el frijol y a esa parte de la alimentación nosotros hacemos vigilancia. ¿Cuál es el menú que tienen? Ah este día se le va a hacer esto y esto a los niños, para mañana cambia el menú, se le va a dar otra cosa. La escuela que puede y tiene apoyo con otros fondos, le está dando bastante variado. El programa de gobierno le cubre una parte y ellos ponen otra parte o hacen un huerto escolar. No hay la costumbre acá en la comunidad del consumo de frutas y verduras. No existe o no hay acceso o no tienen la costumbre. Se podría cultivar, pero como no les gusta no lo hacen, prefieren lo más rápido, una comida enlatada y ya.

Hay un libro que creo la Cruz Roja de alimentación, “hojas verdes”, que se consume, ellos no la conocen. Este libro se les entregó a algunas personas de la comunidad, por ejemplo, una señora que estaba ayer en la reunión lo tiene en Nueva Esperanza. Pero Nueva Esperanza tiene otra cultura. Ellos se repatriaron desde Nicaragua, tienen otra forma de vivir, ellos comen diferente, quizás mejor, desayuno a su hora y variado de comida, comen frutas y verduras, también las cultivan, comen productos lácteos, pero aquí tenemos familias que la leche toda la venden y compran otra cosa. Aunque en Nueva Esperanza cayeron últimamente en el problema de la insuficiencia, ellos se mantuvieron, tuvimos un tiempo que ellos se mantenían, el trabajo de la cooperativa les afecto, porque de los dos que fallecieron uno de ellos trabajaba de lleno en la cooperativa, el otro era un paciente que tenía un retraso, obesidad, otras enfermedades coronarias que él tenía, entonces eso le afecto, y también falleció rapidito. Uno sobrevivió, por lo que puede ser la forma en la que se cuida, tiene oras prácticas de vida, la familia lo apoyo, dejo de trabajar, ya se dedico no mas a cuidarse. El otro que traía el sustento a la casa, tenia que ver como hacía, es diferentes cosas que pasan en la familia, y uno puede ver que pasa entre las dos.

**I: ¿Cuáles son los puntos de prevención que considera que se están haciendo? ¿dónde son? ¿dónde faltan para hacerlos?**

En la parte preventiva, es bien importante descubrir las enfermedades crónicas que llevan a un daño renal a una persona, como es la obesidad, la hipertensión y la diabetes, la hidratación y la exposición al estrés térmico que es permanente, pero en la parte preventiva es bien fundamental el trabajo educativo, el seguimiento a las familias, las visitas domiciliarias para corroborar si aquella persona que le dejó el medicamento el nefrólogo se lo está tomando o lo abandonó. Si lo abandonó porqué lo abandono, ¿será porque el vecino le dijo que no lo tomara?,¿está tomando un remedio natural? llegar a la investigación de lo que está pasando.

La otra parte bien importante es trabajar con la población joven, con los niños en los centros escolares, con aquellas personas que no estudian, con personas que tampoco saben leer.

**I: ¿Cómo se puede llegar a esas personas que por ejemplo no estudian?**

La población que no estudia, esa persona uno la puede encontrar a través de la visitar domiciliar. Lo que no se hace es el trabajo de búsqueda de esa población y cómo concentrarla. Porque no es lo mismo tratar con personas que saben y leer donde uno les explica un proceso, a sentarse con una familia que no sabe leer, y hacerle entender o que trate de entender la prevención que uno lleva o la que uno lo quiere someter, pero se puede lograr si uno lo hace a través de una visita domiciliaria, en las áreas de juego recreativo. Siempre uno se da cuenta ahí qué joven va a la escuela, que joven no va, si no estudia qué hace, ¿trabaja?, ¿en qué trabaja? ¿cuántas horas trabaja?

Siento que hay que hacer un estudio en esa población, porque también a veces tenemos jóvenes que nunca nos han visitado. Personas que no saben leer y nunca han visitado el establecimiento de salud y no tenemos información de ellos. Porque cuando vamos a visitarles no están, están en el área de trabajo, o andan visitando a alguien, por alguna razón no tenemos datos de esa persona.

Hay formas de llegar a la parte educativa, pero se requiere de una planificación de trabajo por el equipo, para poder organizarse y buscar esa población. Porque estamos acá, pero estamos esperando a la población que viene, y con esa tratamos, pero afuera hay una población que no viene.

En el Ministerio hay laboratorios reactivos en cantidades que no podemos desplazarnos a la parte fuera y decir “Ah vamos a hacer una campaña y decir que queremos examinar a la población de diez años en adelante con este o ese examen”, es que no tenemos el recurso. No así el fondo lo hacía, porque con una donación, ya sea la Cruz Roja o de alguien más, de Bélgica o de alguien que tienen allí, consiguen una donación, buscan un laboratorio privado, llega el laboratorio, y dicen va a valer, qué, 5 dólares el combo de exámenes. Hacen el llamado. Pero hay personas que nunca han ido. En la visita domiciliar le pregunto a un joven “¿has hecho algún examen renal?”, y responde “no, nunca me lo he hecho, tengo miedo a hacérmelo”. Ahí es donde hay que trabajar con la población

**I: En el caso de los promotores de salud, ¿una de sus tareas no es llegar a estas familias?**

Es que al promotor de salud le han dado unos programas específicos, y le hacen una programación de los programas materno – infantil estrictamente, pero en la enfermedad renal, hasta hoy, en estas reuniones, ha surgido que participe el específico, pero no se ha venido trabajando ni en los seguimientos al paciente que ya está con DPCA ni a la población que no se ha estudiado. Se requiere que se haga un enfoque hacia esa población, por medio del trabajo comunitario, para que se pueda llegar a esa población que nunca se ha llegado, que nunca se ha muestreado, que no tenemos información de ellos. Así como yo se lo hago a mi hijo, porque sé que se lo tengo que hacer una vez al año, pero conozco un montón de jóvenes que no tenemos datos de ellos. Que cuando volvamos a tener va a ser porque el consultó porque se siente mal, entonces nos va a dar datos, que por fuerza va a tener que llegar a un medicamento. Mientras que si lo hiciéramos, pero como los recursos no nos alcanza, si lo hiciéramos, pudiéramos verificar cómo está ese joven, y también nos sirviera para comparar información, porque este y este que tienen la misma edad y este tiene la enfermedad y este no, entonces ¿a que se dedica? ¿que trabajos hace este muchacho?

**I: En el tema de población, vemos la cuestión de la violencia social que ha hecho que mucha gente emigrase de toda esta región**

Los problemas sociales han venido a generar un problema en la población juvenil porque la sensibilidad de llegar a esas familias, a esas personas no es fácil.

Tenemos la Isla de Méndez, que es el caserío céntrico, pero tiene una población acá que es San Juan del Gozo, que esta población pasó un buen tiempo que no hubo lugar de consulta. Su lugar de consulta era Méndez, pero no podía cruzar para acá por la violencia, ni podía cruzar para acá donde nosotros, la misma violencia. Teníamos que sacar a las mujeres embarazadas directamente al hospital, o sea, fueron casos bien difíciles, y todavía se sigue dando.

Para poder llegar a esa población joven, es montar ahí en ese caserío, que pasa de doscientas viviendas más lo otro que está allá pegado al mar, que no hay acceso a llegar (por la problemática de la violencia), un estudio y llamar a una cantidad de la población, y hacer un estudio, examinarles para ver cómo están los resultados.

Hoy en día se puede llegar ahí pero antes no. Yo siempre dije si el Fondo de Emergencia trabajaba, pero nosotros desde el Ministerio no podemos porque no podemos hacer nosotros el tamizaje a la población, ya que el laboratorio no da abasto con los reactivos. Como es a nivel nacional que se abastecen los reactivos, no se puede decir darnos una cantidad para un tamizaje, eso solo se dio cuando se llevó a cabo aquel estudio, y pusieron todo un equipo, pero sino no damos abasto para llevar a cabo ese tamizaje.

Cada laboratorio tiene sus metas para trabajar, y tiene la cantidad de reactivos que le asignan, no se como de factible es de que ellos asuman esa responsabilidad. Al menos si se montará un programa y hubiera un fondo extra para esos reactivos, y un personal dedicado a eso, ver hasta que población se llega. A raíz del estudio que se hizo, donde se descubrieron a estos pacientes, se quedaron en control y lo asumía el ministerio. Antes les daba seguimiento el fondo, pero nos lo pasaba al Ministerio, pero el Ministerio no tenía un programa en sí. Luego sí se realizó, y el Ministerio comenzó a darle seguimiento, tanto en la parte curativa como en la parte preventiva, pero nos quedamos cortos todavía. Aun falta mucho por hacer.

**I: Ves que hay un problema muy importante con la parte de diagnóstico y la identificación de casos. ¿qué hay del seguimiento del paciente crónico? ¿en los casos de que se dé un episodio agudo, qué problemas crees que tiene el paciente?**

El que más se ha visto es la negación del paciente en aceptar su daño renal, siente que se le desmorona todo. Algunos a los que se les viene el doctor, que ya están en estadio cinco en adelante, esa persona es más renuente en aceptar el tratamiento, que es la diálisis. Aunque el médico trate de decirle que cuanto más temprano sea la diálisis mejor va a ser para su cuerpo, el paciente se niega y no lo logra aceptar y se niega, se niega.

El otro problema que se daba, hoy ya no lo es tanto, era el tipo de catéter rígido, que no lo aceptaba el paciente, porque el catéter blando es más caro. Aun así, se logró que San Miguel colocará catéter blando. El seguimiento con el paciente es a que no vaya a agarrar una infección, que haga correctamente las cosas, siempre hay una vigilancia de enfermería…para que este paciente logre hacerse correctamente la diálisis en su casa. Eso si logramos que lo acepte. Pero tenemos la influencia de la familia. Esa es otra cosa con la que nos hemos encontrado. Cuando las familias no ceden o no aceptan que el paciente debe de pasar de un medicamento a la diálisis peritoneal. Es otra cosa con la que lidiar con esa familia. Porque hay personas que depende de lo que diga el familiar. Porque con la cantidad de pacientes que el doctor ve, él no tiene ese espacio de estar dándole una consejería, sí se la da al paciente en su poco tiempo, pero no para el familiar, eso se trata ya en otras disciplinas: enfermería, trabajadora social, la educadora lo sienta y lo explica…

La mayoría sí lo hemos logrado, pero tenemos renuencia del familiar, y es como un tropiezo en el proceso que se lleva.

**I: ¿cuál es la forma de acceder al manejo del paciente en esa situación?**

Se hace a través de la visita domiciliar, porque es el momento donde se tiene más tiempo para escuchar, no dentro de las instalaciones de la unidad de salud, porque al ser una visita integral que hacemos multidisciplinar, y específicamente tiene que llegar la persona que va a llevar a ese caso y lo hace ahí en la forma más cercana que pueda, y un día pueda entender que lo que se está haciendo es para el bien familiar, no tratando de coartar su sistema o su forma de vivir en la persona. Se ha logrado en la mayoría. La familia es un factor muy importante para el paciente renal, porque luego se vuelve dependiente, ya no depende de lo que el hacía antes, que la actividad económica ahora cambió, hay que trabajar esa parte, que va a asumir una responsabilidad distinta, que el ya dio lo que podía dar, ya que con su problema renal no va a poder dedicar las horas que dedicaba a su trabajo. Esa parte es bastante delicada y hay que saberla tratar. Pero al final se logra.

**I: ¿en los acercamientos comunitarios?**

En los acercamientos que se hacen, se va con unos objetivos, bien de buscar a la persona que falta, de tamizar, y en esa parte se logra descubrir otros factores que están influyendo, se lograr abordar la familia, y si está la persona ahí con el daño renal, se le involucra para que vea que la familia le está brindando el apoyo y que no está solo. El problema es cuando se sienten solos, no hay seguimiento de salud y la familia también lo abandona. Pero en esta parte no, en el acercamiento se logran varios objetivos, ver si otra persona dentro del grupo familiar está presentando problemas o se ha quedado y no se ha hecho ningún examen y se trata. A veces es más fácil convencer al familiar, al otro por ahí cercano. En un momento se lleva a la educadora, a la nutricionista para que vea cómo está la alimentación de él. No solamente se mide acá la parte nutritiva, sino que hay que ir a la vivienda y ver que es lo que está consumiendo, si está haciendo lo que le recomendó y si porque por el problema económico no puede. No es lo mismo que yo le diga a la persona usted va a comer de estos grupos alimenticios, se va a controlar en la mañana, la persona solo se queda callada y escucha, pero diciéndose para sus adentros, si supiera que solo tengo tal cosa, y eso se mejora en los acercamientos porque realmente uno ve cuál es su forma de alimentarse, y le da consejos según lo que uno ve que el paciente tiene alrededor y lo orienta, porque es la parte que se educa de cuales son los grupos alimenticios y con cosas que estén a su alcance de preparar un alimento bueno para el. Y se van cambiando los hábitos.

**I: ¿considera que a nivel comunitario existe algún otro soporte de manera que se pueda incidir aún más en los cambios hacia estos hábitos más saludables?**

Apoyos de grupos comunitarios no hay, si se pudiera tener el apoyo de las iglesias evangélicas o de las iglesias católicas o de otros grupos que están organizados, pero la población no está consciente de que tienen que incidir en esos grupos de la problemática del daño renal, solo lo hacen instituciones que nos apoyan, dependiendo del apoyo que salud solicite.

**I: ¿Los cambios antes y después de la reforma en cuanto al manejo del paciente renal?**

Hay una diferencia que se puede notar antes de la reforma como el personal que era menor, no existía lo del acercamiento, siempre se esperaba que la persona buscara los servicios de salud, no contábamos con un laboratorio para ver los problemas inmediatos y ahí dar una respuesta. El único hospital de referencia era el Hospital de Jiquilisco y no lográbamos que la persona se trasladara porque tampoco teníamos transporte.

Cuando yo entré acá solo había una ambulancia que recorría la zona, toda la zona baja de Bajo Lempa. Solo había un médico para tres unidades de salud, que llegaba un día por semana, y el personal solo eran tres auxiliares de enfermería, y no se podía llegar más allá de los programas que veíamos.

Cuando vino la reforma se vio la necesidad de llevar la salud a la casa porque como esa población que nunca nos visita y es la población masculina. Hay hombres que aun con la reforma no nos visitan, pero se va a buscar y llega un momento que los encontramos, pero antes de la reforma no, esa población no nos visitaba o la población de mujeres que no padecían ninguna enfermedad o no se sentían mal tampoco nos visitaba. Las que tenían un poquito de posibilidades económicas se iban al área privada. Solo cuando su problema iba más allá del alcance económico nos buscaban en el área de salud pública. Pero luego con la reforma cambió, se aumentó el recurso humano, se dotó ambulancias y de unidades de transporte.

Antes se llamaban brigadas médicas. Se iban a los caseríos, pero no se hacía ningún registro médico, solo se anotaba que medicamentos le dieron, pero hoy con la reforma se lleva el expediente de las personas a citar, y va ahí el historial, y en base al seguimiento que se le está dando, hay más control digamos, más control de la población, porque se logró llegar hasta la vivienda.

Con la reforma se habrán quedado algunas viviendas que se visitan más tardío, porque son adultos, porque no hay niños, porque no hay una embarazada, pero siempre hay una fecha que se le dedica para visitarlo, ya sea porque es adulto mayor, porque es una persona con una enfermedad crónica, porque hay un niño, pero siempre esa vivienda está en la programación del equipo comunitario, donde se desplaza el médico, el enfermero y el promotor. Si alguno de la familia no está, porque no siempre se encuentran todos, como los jóvenes, que, o están trabajando o están en la cancha. Se les deja una notita que se presenta una unidad de salud para hacerles, algunos vienen algunos no vienen. Y dicen “Ah, pero mira a mí no me visitaron”. Y luego porque al siguiente día es otra programación hacia otra área. Pero las cosas mejoraron totalmente de antes de la reforma a después de la reforma.

Ahora la persona no puede decir “es que fui y no me atendieron”. Si vino y se fue es porque no quiere. Porque aquí hay un control que se lleva. A veces la persona no nos entiende cuando le decimos que hay una emergencia y el médico tiene que, para la consulta, y a ellos no les gusta esperar. Pero eso no es culpa del personal, es porque tenemos un solo recurso en el laboratorio. Cuando tenemos un médico no más, y a ese uno le toca salir al campo entonces no tenemos médico acá. Pero se ha ido tratando de educar a la población que hay días que el médico tiene que andar en el campo, para visitar a esas familias que no se presentan acá. Ya sea por seguimiento o porque van a ir a buscar población nueva que no tiene ningún expediente habilitado que le van a hacer uno, que de qué padece, no le van a citar. Ya la gente fue entendiendo poco a poco, que tres días hay consulta, y dos días el médico va a andar fuera. Aquí siempre hay porque tenemos al director y tenemos al otro, pero no así en las otras: Isla de Méndez, Canoa… solo hay un médico. Cuando a él le toca salir al campo, en la unidad no hay consulta, pero si hay una emergencia si él se desplaza.

En los días de consulta, el médico espera que la población que tiene citada pues le llegue, porque trabajamos citando al paciente para poderlo tratar en la unidad de salud, y si citó a uno y no le llegó, manda al promotor para que investigue porque no llegó, si le paso algo o no quiso llegar. Si vuelve a haber renuencia, se vuelve a desplazar el médico y lo vuelve a visitar.

**I: ¿qué población está fuera?**

Los jóvenes más que todo, y hombres que se dedican a al campo por la mañana.

El médico está llegando tipo ocho y media – nueve de la mañana a la vivienda, a esa hora no está el hombre, siempre encuentra a los niños y a la mujer. Pero la población joven o anda estudiando o está trabajado.

**I: aparte de la falta de recursos, ¿ese médico que pudiera cambiar el horario de visitas al horario de tarde, considera que esa situación mejoraría?**

Claro, en esa parte mejoraría, pero estamos regidos por un horario de trabajo de siete y media a tres y media.

El médico de las dos a tres y media tiene que hacer trabajo para de administración para registrar a esos pacientes y meter la información en su censo, para dar información al siguiente día, o que la secretaria meta al sistema el trabajo que se hizo. Por eso es que por la tarde no hay visitas acá. Por eso que se queda ese grupo familiar fuera, no se puede ver.

**I: ¿Cuáles son sus recomendaciones como acciones prioritarias a realizar?**

A mí me parece que fuera bueno hacer el estudio o los tamizajes a la población que se han quedado en comunidades que nunca se han chequeado, poder llegar a ellos, y tener un dato de la población, desde el más pequeño a la últimas, por familias, cómo está esta familiar, cómo está el joven, cómo salió el niño.

Si un día tuviéramos esa información, tendríamos un diagnóstico. No sé si un día se pudiera lograr porque esto es un proceso, nunca se sabe que va a pasar mañana. Hacer el trabajo más que todo con una población joven, población estudiantil, para lograr de que esta persona cuando sea grande va a lograr y sea consciente de cómo evitar llegar a un problema renal.

Se necesita que ese programa esté anexo a una disciplina, ya sea a la salud comunitaria o a enfermería, quién lo va a realizar, con qué recursos contamos, pero me parece bien importante de que, si tuviéramos un dato por familias, chequearlas, hacerles un tamizaje, educar a la población de la rehidratación, de cómo prevenir una enfermedad renal, concientizarlos. El clima no lo van cambiar, sino que va a empeorar, y eso la población estudiantil lo sabe, que por el cambio climático que tenemos vamos en peor. Lo que hay que hacer es aprender a vivir con ese problema. La forma de aprender es que conozca cómo prevenir, como hidratarse, y no llegar hasta el problema de donde está. Que, si el papa llegó, que el hijo no vaya a llegar, que se concientice a través de la educación que se le dé. En la población estudiantil que tenemos se está trabajando en temas como lo que más nos está afectando a el país como es la violencia, se está trabajando en eso.

Entonces son esos programas que ha visto el Ministerio como de urgencia de tratar, la violencia juvenil, pero no este programa en sí.

Puede ser que un día lo retomen. Digan, miren no se va a trabajar en violencia, en enfermedades de transmisión sexual, se va a trabajar en la prevención de la enfermedad renal.

**I: desde tu posición para el entorno, ¿qué crees que se debería de mejorar?**

Para mejorar quizás sería involucrar algunas de las instituciones que no nos brinda apoyo, las Directivas, las ADESCO, nos hemos quedado solo en salud.

I: ¿QUÉ ES ADESCO?

Es la unidad rectora de una comunidad, la autoridad máxima digamos, la Junta Directiva, que está avalada por la alcaldía municipal, que tiene unos estatutos.

Y las instituciones que se involucraran más en apoyar al sistema de salud, porque sentimos como que nos hemos quedado solos.

Nosotros tenemos todos los programas. Tenemos darle y buscarle solución, sino la comunidad se queda, no más. En un futuro para que las cosas mejoren, aunque tuviéramos el apoyo comunitario y de las instituciones presentes en la zona, dependiendo de donde estamos. La comunidad está organizada, pero no para apoyar o decir tenemos el Ministerio de salud, pero esto es lo que nosotros vamos a aportar. Que se yo, organizamos la comunidad tardía y ustedes vienen y hacen su actividad. Pero no contamos con ese apoyo en la comunidad. Si eso se llegara a dar, funcionaría mejor y mejorarían muchas cosas. Porque también lo jóvenes fueron aprendiendo de ahí, de apoyar en la parte de salud en los temas que el Ministerio hace llegar a la comunidad. En la reunión de ACUDESBAL que es donde se reúnen todas las organizaciones de la zona vamos a hablar de eso, ahí están asociados, por ejemplo, un grupo de hombres ganaderos, una organización de mujeres, y a través de esos grupos se llevan distintas actividades, de paseos, módulos de gallina comedora, de cerdos, siembra. Es una organización muy fuerte que, si pudiera trabajar con salud, se reuniría a mucha gente donde el medico se podría desplazar y dar la ponencia sobre la prevención y que se yo, también tamizar a una gran cantidad de hombres. También, hay muchas mujeres que se las puede reunir.

**I: ¿QUé otros lugares organizativos hay en estas comunidades aparte de acudesbal?**

Lo otro son las cooperativas de la pesca, ahí están organizadas cuatro cooperativas de pesca, son camaroneras, tienen estanques de camarón.

**I: ayer se hablaba de que hubiera una ley que obligará a las cooperativas a utilizar material de protección o incluso el uso de sueros en los agricultores. ¿existe alguna organización que trate de manera más específica estos aspecto?**

No, de momento no.

En ACUDESBAL trabajan el género, con grupos de jóvenes y trabajan sobre los derechos.

Con los jóvenes se reúnen en el Zamora, y a veces en el Acudesbal. ¿Qué temas imparten? Lo desconozco hasta ahí porque no les he visto el plan, que temas tienen incluido…Pero sí tienen un trabajo con jóvenes aparte de las actividades deportivas. También tienen a cargo el grupo de becados, tienen un población con beca estudiando en La Nacional y en diferentes Universidades de El Salvador.

**I: ¿qué nexo existe con el sector privado?**

La enfermedad renal para algunos doctores se convirtió como en una mina de oro, pero aun cuando inicio, había una clínica en San Miguel privada, ahora esta hasta en Usulután. Luego se crearon unidades especializadas diálisis, y cuando ocurrió eso, los médicos de los hospitales los mandaban allí porque eran suyas, y cobraban 160 dólares e iban dos veces por semana, las personas quedan absolutamente sin nada, la persona falleció y quedaba sin nada la familia, eso hizo que mucha gente no entrara en el tratamiento sino que prefería morir, muchas personas, y aun hoy se sigue oyendo esto, que para hacerse diálisis mejor morir, porque todo ese proceso es costoso. La parte privada se lucro y se sigue lucrando de la enfermedad renal, y se crearon comités de médicos, asociaciones que pensaban que el paciente en lo privado se iba a curar, pero nunca le explico que el paciente no se va a curar si no se hace diálisis, entonces les hacen hemodiálisis en lo privado. Si que se ve que los mismos medico de san Miguel digan que no hay cupo para hacer la diálisis y se los lleven a sus propias clínicas, solo si fallece alguien le da el cupo, entonces en toda esa espera se nos murieron a nosotros varios pacientes, porque no alcanzaron el cupo en el hospital o no alcanzaron económicamente para pagar la clínica privada.

Ya de lo del tema de curanderos, quizás para el bien de nosotros, falleció la mujer que había en Santiago de maría que atendía a bastante población de nosotros. Y la gente no atendía sus citas aquí por ir allí, y le daban medicinas naturales, de tratamiento que lo único que hacía era que avanzara la enfermedad renal, se empezó a trabajar y trabajar, pero luego lo sobrellevo y se empezó a preguntar a los pacientes para que consultasen con nosotros lo que se estaban tomando, porque por ejemplo una señora no lo llevo a un curandero de allí de san francisco, porque la consulta era 400$, pero que no era caro, pero luego no mejoraban el paciente.

El sistema privado da una respuesta inmediata porque no les lleva mucho tiempo, esa persona por ejemplo le mandó a Jiquilisco, de ahí a San Francisco, que no la hallaron ninguna solución, y me dijo que estaba en lo mismo, que le hablo a una doctora que estaba muy molesta, que no la daban ninguna información, y entonces la señora se sentía como. que no la había hecho nada, entonces la señora quiere una solución inmediata. También se tiene la cultura de cuando se paga, le van a dar mejores medicamentos, y son los mismos.

## Staff interview 7: Medical student completing year of social service

Entrevista con: Estudiante de medicina

Fecha: 13/06/18
Lugar de la entrevista: Unidad Comunitaria de Salud Familiar Jiquilisco
Código del informante: SMMJ007
Genero del informante: M
Nombre del entrevistador: MJ

ENTREVISTA SMMJ002 US SAN IGNACIO

**Sección 1**: Perfil del participante y función en la prestación del servicio del primer nivel de atención

• Perfil breve del participante (antecedentes, capacitación, historial de participación en la unidad de salud o ECOS)

- Quisiera preguntarle su edad y su formación y un cómo ha terminado trabajando acá en la unidad de salud de San Ignacio.

Soy estudiante de medicina tengo 27 años actualmente estoy realizando mi servicio social aquí en la unidad de salud básica de San Ignacio, hasta el momento estoy muy contento aquí. Aquí los pacientes que más consultan en primer lugar por infecciones respiratorias agudas y también tenemos un gran número de pacientes con enfermedades crónicas sobre todo hipertensión arterial y diabetes, insuficiencia renal es más raro, aunque en este año diagnostiqué a dos en grado 3 que tuve que mande referencia al médico especialista de La palma. Los dos pacientes eran hipertensos, uno era agricultor y la otra era una mujer diabética con mal control con glucemias hasta de 600, en el caso del agricultor su profesión pudo afectar más que todo por el uso de pesticidas y al estar expuestos al sol la poca hidratación y todo eso le lleva a desarrollar la enfermedad además del hecho de ser hipertenso. Empecé en este lugar desde enero 2018, yo lo elegí tras realizar el examen después del periodo de internado el último año de Carrera, dependiendo de la nota podemos elegir un lugar u otro del país y a las unidades de salud que abarca cada región. Yo soy de San Salvador del barrio de Mejicanos. Hay demasiada competición a la hora de coger la región porque no solo somos de la Universidad nacional, sino que también todas las privadas, de la UES solo éramos 290, de otras universidades que son como 5 universidades en total, añadiendo también los médicos que vienen de cuba que para homologar su título tienen que hacer el servicio social acá y entran también en la misma rifa. Se va a lanzar la convocatoria la próxima semana para acceder a las especialidades, en mi caso voy a aplicar a medicina interna en el seguro social. En lo personal a mí el primer nivel de atención me parece bastante Bueno es un ambiente tranquilo donde uno puede influir bastante en la vida de las personas, sobre todo en las zonas rurales uno que es médico le hacen bastante caso y siguen bastante las indicaciones que uno le da y si es in ambiente donde se puede hacer mucho por la vida de las personas. Me gusta mucho pero no creo que me quedara aquí el resto de mi carrera, buscaría más que todo el ambiente hospitalario.

• Su función actual en el primer nivel de atención

Yo principalmente mi función es la consulta, yo soy el primero que los atiende. EL otro médico que está aquí conmigo que es el coordinador se dedica más que todo a labores administrativas por lo que es raro que este aquí y suelo estar yo solo. Hay horarios en los que se anotan los pacientes que vienen, a las 7:30 se anotan los pacientes que están y luego a las 10:30 los que han venido después así que aquí se les va atendiendo según llegan. EN in día normal llego a las 7:30 y usualmente ya hay pacientes esperando para pasar consulta, en in día normal vemos como 20-25 pacientes en total. El principal motivo de consulta son las infecciones respiratorias, hoy hasta ahora he visto 9 y de ellos 5 tenían infección respiratoria. Veo a todas las edades. Tenemos ciertos controles preventivos el más importante es el de menores de 5 años que vienen a chequeo, en menores de un año cada 2 meses, de in año a 2 año son cada 3 meses el control de 2 a 5 años se les ve cada 6 meses. Igual hay in programa para adultos mayores de 65 años para distribuirles la medicación para enfermedades crónicas y hacerles el chequeo. Y el otro más importante es el de embarazadas para hacer el control de la mujer y el niño en gestación. De los 20-25 pacientes al día como unos 7 pacientes tendrían enfermedad crónica, la mayoría ya están diagnosticados y traen el tratamiento, la mayoría bien a por el medicamento y comprobar que todo esté bien, yo intento que cada 3 meses tengan examines, sobre todo en pacientes diabéticos, dependiendo de lo que yo identifique en los examines les cambio el tratamiento, Bueno yo puedo proponerles un cambio pero siempre tengo que mandarle la referencia al internista/familiar de la Palma para ver si está de acuerdo y luego me manda la contra referencia con las indicaciones, él es quien tiene la última palabra porque es el especialista.

**Sección 2**: Comprender las trayectorias de los pacientes en el primer nivel de atención

• ¿Podría **describirme su participación con pacientes con enfermedad crónica**?

Un paciente hipertenso o diabética que son los que más se presentan aquí vienen, se anota para la consulta, las enfermeras les pesan tallan, les miden la temperatura y la tensión. Cuando llegan a mi consulta yo les vuelvo a tomar la tensión porque a veces llegan andando cansados y hay que volver a tomarla como a los 10 minutos y la verdad es que suelo variar. Normalmente vienen con su tratamiento habitual, pero si hay alguno que tiene la tensión alterada o ha salido algo mal en los examines ahí es cuando les mando al médico especialista de La Palma. Yo luego si he identificado algún factor de riesgo o que algo no va bien les vuelvo a citar en in mes para hacerle un nuevo chequeo.

• **¿Cuáles son los principales desafíos que enfrenta al cumplir su función?**

**El principal desafío es que el paciente cumpla con el medicamento o con las recomendaciones**, sobre todo cuando son recomendaciones sobre los estilos de vida, sobre la dieta principalmente, porque es bien raro que la gente esté dispuesta a hacer cambios, aquí existe mucha prevalencia de sobrepeso y obesidad, pero es raro que la gente cumpla la dieta. La gente aquí tiene una alimentación y se les dificulta mucho para cambiar.

**• ¿Y qué haces para que cambien?**

Yo les insistió mucho en que si no cambian sus estilos de vida o que bajen in poco de peso la enfermedad les va a progresar hasta un momento que no pueda ser controlado por medicamentos. La economía es la principal razón para no cambiar la dieta, buscan lo más económico para la familia y aquí más que todo las pupusas, toda la gente aquí las come porque son baratas y accesibles, la mayoría de gente come cosas fritas que también perjudican a las enfermedades de la población.

Es raro que yo salga a la comunidad pero alguna vez voy a ver a pacientes que no pueden movilizarse a la unidad de salud y algo que se ha cambiado ahora es que ya no las puede recoger la familia del paciente, por ejemplo en el caso de una paciente con 60 años y artritis que no puede venir antes se lo daba a la hija pero ahora ya no se puede y ahora se ha organizado que vayamos a sus casas, peor es raro yo desde que estoy acá solo he salido unas 3 veces a vista ese tipo de paciente más inmovilizados.

**Sección 3:** Trayectorias de cuidado de personas con enfermedad crónica

• ¿Podría describirme los pasos que sigue un paciente crónico desde que entra en la unidad de salud?

- En el caso de pacientes con enfermedades crónicas, **primero vienen les anotan en el archivo que es donde están todos los expedientes, luego pasan a la enfermera les toma las medidas antropométricas, tensión y temperatura y luego ya pasan a la consulta les receto el medicamento, luego pasan a la farmacia y luego si vienen a por control también se le pasa a odontología, no se cada cuanto les evalúa la odontóloga** ¿Pero se hace a todos los pacientes o solo para enfermedades crónicas? No se hace a todos los pacientes, es parte de los programas para cada tipo de pacientes, menores de 5 años, embrazadas, todos llevan sus controles y de ahí se van, si es necesario se les da referencia para la unidad especializada de la Palma o si es una emergencia se hace la referencia al hospital que nos corresponde que es de la Nueva Concepción aquí en Chalatenango.

• ¿Podría describirme los pasos que usted sigue para llegar al paciente crónico? Los enfermos con enfermedad crónica llegan a mi más que yo salir a por ellos, pero en personas no diagnosticadas pero que sí que tengan algún factor de riesgo. ¿Podrías poner algún ejemplo concreto? Por ejemplo, personas con sobrepeso u obesidad o que tenga antecedentes familiares les hago exámenes una vez al año para detectar tempranamente esas enfermedades. ¿Cómo localizan a las familias? Por la misma consulta, cuando bien aquí ,los expedientes clínicos están organizados por familias, en cada expediente cada familia tiene in número a donde pertenece la casa el barrio pertenecen aquí en ciudad de San Ignacio y sobre todo lo detectamos cuando vienen a la consulta, además las promotoras de salud tienen unas fichas con distintos números, **grupos de dispensarizacion** se les llama aquí, el **grupo 1 son personas sanas , grupo 2 con algún factor de riesgo (como sobre peso obesidad o antecedentes familiares), el grupo 3 son pacientes ya con alguna enfermedad crónica y el grupo 4 son los que tienen ya alguna discapacidad debida a su enfermedad** por ejemplo aquel paciente que desarrolla ceguera por su diabetes. Esto es a nivel individual, pero luego a cada familia también se le clasifica en grupos de despenalización que son los mismos, pero ya a nivel familiar y ahí es donde vamos identificando las familias que tienen riesgo y ahí vamos viendo por cada paciente para identificar si llega a presentar alguna enfermedad de estas. ¿Y con los pacientes que identificas riesgo cuando le dices que quieres monitorizarles, cuál es su respuesta? Si ellos sí que bien a hacerles los examines que se les indica, pero hablando de los estilos de vida eso es más difícil influir. ¿Pero cómo sientes que los pacientes responden cuando les dices esto, ponen atención o cómo actual? Sí, ellos ponen atención porque se preocupan verdad, sobre todo si han visto a sus familiares (abuelos o papas) y los han visto sufrir por estas enfermedades ahí sí que ponen atención porque se preocupan.

• ¿Puedes poner algún ejemplo concreto acerca de tu experiencia brindando apoyo para pacientes crónicos?

Sí, por ejemplo, tuve una paciente de 60 años que vino a consultar porque se sentía marea, mal, débil, vino diciendo que a ella nunca le habían dicho que tenía una enfermedad, pero una vez que se encontró mal en el Salvador, unos médicos de estados unidos que venían en una campaña sanitaria le dijeron que era diabética y le pusieron insulina pero que solo tuvo lo que le dieron allí. Ahora venía a mi consulta con unos examines que le había recomendado la promotora de salud y que se los había hecho en in laboratorio particular y venía con 600 de glucemia, colesterol de 300...todos los examines estaban mal y nunca había recibido tratamiento. Eso que le pasó de que se desmayó había sido hace 2 años y había pasado mucho tiempo sin tratamiento así que le expliqué que era diabética, necesita tomarse el tratamiento todos los días, le voy a mandar a que le vea el medico familiar en la palma, se puso a llorar, porque si me hubieran dicho antes yo hubiera cumplido el tratamiento sobre todo porque se hubiera podido detector mucho antes.

• ¿Qué tan factible es brindar un mejor soporte a los pacientes que lo necesitan?

Es bastante factible que se pueda mejorar, pero la población tiene que poner de su parte y eso es lo que más cuesta. Por ejemplo, uno les deja cita para in día y a veces no vienen y se presentan a la siguiente semana, entonces para mejorar tenemos que ponernos a trabajar todos, principalmente las promotoras que identifiquen quienes son los pacientes que se les dificulte o se les complique llegar y que les estén siguiendo y explicándoles porque deben venir a la consulta y les den educación sobre su enfermedad. Se está creando un grupo de adultos mayor que se les está dando charlas de todo sobre enfermedades crónicas, hasta la fecha solo se ha hecho una, pero fue bastante la colaboración de la población para esta actividad así es que yo pienso que se deberían hacer más este tipo de actividades.

- ¿Los pacientes que dices que no acuden a la consulta, porqué razones crees que es?

Principalmente porque se les dificulta venir, porque no solo vienen de la ciudad de san Ignacio, sino que también están como adjuntas la unidad de salud de Las Pilas y el Carmen, estas unidades son los que le dicen ECOS y el medico que está en estas comunidades

Hace labores de dar consulta y labores administrativas también, entonces hay momentos el medico de esa unidad no está presente porque está en alguna reunión o algo así y vienen a consultar aquí. Por eso la población que se ve aquí es grande y a veces tienen que movilizarse mucho y eso les cuesta bastante.

• ¿Puede pensar en algún problema que presentan más frecuentemente los pacientes que realizan su seguimiento con usted?

El que tengan **problemas para movilizarse** es de los principales, otro problema que se ve bastante es el **desabastecimiento de medicamentos** que pasa frecuentemente, aquí normalmente tenemos lo básico, pero algunos que le han puesto el especialista o son más especiales esos sí que tienen que comprarlos.

- ¿Cuándo sí están los medicamentos se les dan gratis?

-Si, cuando los tenemos acá se los damos gratuitamente.

• ¿Ha notado alguna diferencia en la forma en que los pacientes y el personal interactúan entre sí desde que comenzó este abordaje con la reforma?

En cierto caso si he notado un cambio principalmente por lo que les decía que ahora ya no se puede despachar medicamentos a familiares de pacientes que no pueden venir, eso tiene su lado bueno y su lado malo, el lado malo es que hay pacientes a los que se les dificulta el transportarse a la unidad de salud. Pero el lado bueno es que se tiene más control de los pacientes, antes se les daban los medicamentos por 2-3 meses y pasaban ese tiempo sin venir a chequearse ahora tienen que venir mes a mes obligados. Se está queriendo implementar in Nuevo programa de **receta repetitiva** en la cual se pretende que el paciente no tenga que ir a consulta, pero si tenga que venir a la unidad de salud donde la enfermera le toma la tensión y todo y si está todo bien se les despacha el medicamento y no tiene que pasar consulta. Esto más que todo para agilizar porque hay gente que viene solo a por medicina y que tenía que pasar consulta, pero está bien que tengan que venir a la unidad de salud para al menos chequearse con la enfermera.

¿Y pacientes que te hayan comentado si han visto cambios a la hora de recoger la medicación si antes pagaban o no? Principalmente he recibido quejas por lo mismo, porque les cuesta mucho movilizarse hasta acá y como no se les da la receta para varios meses tienen que venir y eso les molesta, pero es todo por el bien de los pacientes, pero algunos no les gusta.

- Hablando de quejas, hemos visto el buzón de sugerencias, ¿crees que se utiliza?

- Poco, yo no lo he visto utilizar, pero si se lo he sugerido y les he informado que está el buzón de sugerencias.

- ¿Qué les dices cuando vienen con esas quejas?

- Se les explica que no es porque queremos sino porque hay ciertos lineamientos que hay que seguir y se explica cómo está cambian el Sistema de salud.

- ¿Y si tienen quejas como hacen? Pueden llegar directamente al ministerio de salud.

- ¿Pero estos buzones luego se llevan a la oficina del derecho a la salud no? ¿Sabes si existe aquí en el hospital?

- Si si existe, en cada hospital hay uno.

- ¿En tu formación universitaria consideras que se te ha formado suficiente en enfermedades crónicas?

- La verdad es que sí, en mi caso hablo de la universidad nacional (UES), nosotros rotamos en el principal hospital del país y de ahí vemos muchos pacientes con en crónicas y la que más abunda es la ERC, diría que casi un 60% de los pacientes de urgencias vienen por complicaciones de ERC.

- ¿Y dentro de tú formaciones pasas por el primer nivel de atención? Sí, bueno hay varias rotaciones, existe una materia que se llama salud comunitaria en la cual a partir de tercer año nos hacen hacer visitar comunitarias a una comunidad donde vamos a hacer labores de prevención de tercero a sexto curso siempre en la misma comunidad, nos dividen por parejas y vamos siempre a las mismas familias a hacer talleres de prevención. El séptimo año hacemos 5 rotaciones, todas en el hospital (cardiología, cirugía, pediatría, ginecología) y una de salud pública que dependiendo del área geográfica de donde vive cada interno se le adjudica una unidad de salud y ahí es donde vamos a trabajar por un mes.

**Sección 4**: Estrategias en el manejo de pacientes crónico

• ¿Qué tipo de problemas o desafíos surgen en el manejo del paciente crónico además de los ya comentados anteriormente?

La adherencia al tratamiento que ya había comentado por falta de conocimiento de los medicamentos que están tomando. La mayoría de pacientes crónicos no saben lo que toman.

Hay personas que viven en zonas muy remotas y se les dificulta venir sin transporte público o lo tienen lejos.

Hay algunos que se les dificulta seguir las recomendaciones sobre todo de la dieta, normalmente es porque la familia no les apoya porque el tener una alimentación concreta es un gasto extra y ellos ya tienen sus menús establecidos a los que sus economías les alcanza y el tener que tener una dieta especial lo ven como algo tedioso, como un gasto extra.

Eso y el poco conocimiento que tienen las personas sobre las enfermedades, uno les dice, mire usted es diabético o hipertenso, por eso en grupos del adulto mayor lo que se intenta es explicarles cosas básicas sobre su enfermedad, que es y que complicaciones pueden tener.

• ¿Cuáles son algunas estrategias que ha utilizado o que ha visto que han usado otras personas, para garantizar que el paciente demuestre una comprensión clara de la importancia del cuidado de su enfermedad?

Como muchos no saben lo que toman yo lo que hago es hacerles una lista con todos los medicamentos que toman para que lo lleven también al hospital cuando lo necesiten o la unidad especializada.

Yo lo que hago es dar conserjería, una explicación sencilla sobre el porqué de la enfermedad y los factores que pueden hacer que progrese y como evitarlos, como prevenir que siga avanzando.

• ¿Y cuándo das estas explicaciones supongo que habrá pacientes más y menos receptivos a esa información, porque crees que hay esas diferencias?

Por el nivel educativo de cada paciente, algunos no han recibido ningún tipo de educación. La mayoría son poco receptivos porque no entienden, hay que explicarles detenidamente y es bien difícil que lo entiendan. Y hay otros que estudiaron por ejemplo los estudios básicos y son más receptivos a la información de tratamiento y de todo.

Hay otro factor que influye bastante por lo menos en mi caso porque me ven que no soy médico todavía por estar en el año social, no me tienen tanta confianza como al coordinador de la unidad, mi estrategia es brindarles todo el apoyo siempre que vienen y cuando vuelven ya confían más en mí, pero la primera vez siempre hay cierta desconfianza porque no estoy graduado todavía.

¿Se te ocurre algún caso en concreto? El de la señora diabética que les comentaba, que al verse tan mal ha sido muy receptiva a todas las recomendaciones que le he dado.

• ¿Qué recomiendan como formas de comunicarse con persona con enfermedad crónica para ayudar a mejorar el manejo de su enfermedad?

Hay que ser honesto con los pacientes y explicarles lo más que se pueda sobre sus enfermedades porque la mayoría desconoce mucho sobre su enfermedad sobre el tratamiento así que hay que tener paciencia y explicarles lo más que se pueda hacerles exámenes de laboratorio para que su enfermedad no vaya progresando. ¿Los pacientes que bien ya los conoces? La mayoría sí, ya los he visto varias veces y eso influye porque tiene más confianza y hacen más caso a las recomendaciones.

• Ahora nos gustaría preguntarte sobre tus apoyos como personal sanitario cuando te encuentras con algún desafío.

Como parte de la rifa también depende quienes reciben un salario, este año han sido solo 260 plazas remuneradas para todos los que nos presentamos así UE hay muchos compañeros que no están recibiendo un salario, depende de la nota y para mí eso influye porque hay algunos que hacen mí mismo trabajo sin remuneración y eso puede influir en las gansas con las que un profesional llega a su trabajo.

De apoyo tengo ciertos amigos en cada área de especialidad que hice durante la carrera e internado, si tengo alguna dificultad les llamo para pedirles su opinión. Aquí en la unidad el personal de salud me apoya también mucho y también puedo acudir al coordinador que me resuelve las dudas con todo gusto.

Aquí a nivel comunitario hay médicos privados con los que también tengo cierta comunicación y podría acudir a ellos. El principal apoyo para mi es mi padre que también es médico y le pregunto cuando tengo dudas. Acá en la comunidad las directivas de las comunidades que saben cómo moverse en la comunidad, comités de salud les llaman, cuando se quiere hacer una charla o una actividad por ejemplo la campaña de citología de la semana pasada la organizó el comité de salud y la difundió en la comunidad y vinieron más de 100 mujeres.

**Sección 5**: Experiencias con componentes de intervención en torno a la estrategia de fortalecimiento de la capacidad instalada de las Redes Integrales e Integradas de Salud (RIIS), para mejorar su respuesta, acceso y calidad de la atención a las personas, familias y comunidad, reduciendo así́ las complicaciones y mortalidad de forma prematura causadas por las ENT.

¿Os dieron formación específica sobre los lineamientos sobre enfermedades crónicas en la formación?

- En las rotaciones hospitalarias no, pero en la de salud pública si nos enseñaron algo y nos entraron en el examen para elegir el lugar de servicio social.

¿Consideras que pueden mejorar el manejo del paciente crónicos?

- Si porque dan instrucciones sobre cada cuanto debes hacer exámenes a un paciente crónico, con que medicamentos empezar, que seguimiento dar, ¿son una guía estructurada para el seguimiento del paciente crónico?

- ¿Consideras que se están implementando en el primer nivel de atención? ¿Sí en lo que yo he visto sí que se están siguiendo estos lineamientos?

- ¿Y para la búsqueda activa de un potencial paciente crónico como hacen para la detención temprana?

Identificar los factores de riesgo en la consulta, pero previamente los promotores identifican a los pacientes en riesgo y les dan charlas y consultorías sobre estilos de vida, aquí es un trabajo en equipo, si ellas detectan riesgo les dan referencia para venir a consulta y yo ya me encargo de hacer todo lo necesario para localizar ese riesgo, riesgo en términos biológicos, pero también sociales (de violencia etc.).

- ¿Qué te parece lo más desafiante a la hora de implementar estos lineamientos?

- Lograr la máxima cobertura porque los recursos en la comunidad son bien limitados y a veces es difícil dar todo a todos los pacientes porque a veces no tenemos todos los medicamentos o recursos que necesitan. Otra cosa muy problemática es que el hospital de la zona está bien lejos y el transporte se les dificulta y aquí solo disponemos de una ambulancia que a veces es compartida con las otras unidades de salud. La mayoría cuando se les dice que tiene hacerse algún examen especifico en el hospital lo ven como un gran problema porque no tiene recursos para movilizarse y el transporte público es bien tedioso. Otro desafío es que la gente entienda un poco más sobre estas enfermedades y así al ser conocedores de la enfermedad y como se desarrolla puedan hacer cambios y cambiar aquellos factores de riesgo modificables, pero para eso tienen que conocer algo y saber porque se les está dando tratamiento y que pasa sino lo toman, para mi es el mayor desafío, por muchas charlas y conserjerías es difícil que lo entiendan.

¿El momento político actual lo consideras un desafío?

-Sí porque se ha dado dinero a otras cosas últimamente más que a salud y por eso hay desabastecimiento o por ejemplo mis compañeros que no reciben remuneración.

- ¿Crees que pueda mejorar?

- Lo veo muy difícil porque los nuevos gobiernos siempre quieren cambiar las cosas y no creo que mejore.

- ¿Crees que se debe mantener la reforma?

-Si porque ha traído muchas cosas nuevas pero cada gobierno nuevo quiere cambiarlo todo y eso puede afectar a como se atiende ahorita desde el primer nivel de atención.

## Staff interview 8: General practicones

Entrevista con: Médico general

Fecha: 13/06/18
Lugar de la entrevista: Unidad Comunitaria de Salud Familiar Ciudad Romero, Bajo Lempa.
Código del informante: SMMJ008
Genero del informante: M
Nombre del entrevistador: MJ

**Sección 1**: Perfil del participante y función en la prestación del servicio del primer nivel de atención

- Me gustaría preguntarle sobre su perfil, sus antecedentes, donde se formó cómo terminó trabajando en esta unidad de salud, un poco todo eso.

- Tengo 32 años de edad, estudié medicina en Venezuela, me gradué en 2015 empecé a trabajar en 2017 y soy de acá del Bajo Lempa, específicamente de la Canoa. Bueno, en realidad me crie en Morazán, pero cuando decidí empezar la carrera de medicina, para muchos jóvenes de acá del El Salvador, era como un sueño, más que todo por la falta de recursos económicos, pero en mi país, en las diferentes etapas que tuvo, se pudieron conseguir ciertas becas tanto para ir a Cuba como a Venezuela. En mi caso, me dieron la beca para Venezuela. El objetivo de todo esto era volver y poder atender a nuestra gente que, para aquel entonces, en los años 2007, era impensable que hubiera médicos dentro de las unidades de salud públicas y los médicos tampoco querían porque era más que todo montaña. Ahora ya tenemos hasta una calle, aunque sea de arena, pero bueno está mejor, también tenemos un servicio de agua no privatizado todavía gracias a la lucha de la gente de acá de Bajo Lempa y ahora ya contamos con la red de servicios de salud públicos en todo Bajo Lempa los distintos cantones de Zamorano, Ciudad Romero, San Marcos Lempa, La Canoa, Isla de Méndez también cuenta con una US que pertenece a la Microred de Jiquilisco que vienen a dar servicios a toda la población. Y también se cuenta con ECOS básicos que lo forman un médico, una enfermera, una promotora, polivalente y motorista donde se tiene acceso a una ambulancia, una secretaria y una ordenanza, eso es lo que forman los ECOS, que están dentro de las unidades de salud.

- ¿Cuándo volviste de Venezuela, tuviste que hacer el servicio social acá?

Sí, hice mi servicio social en 2017 por el que no me pagaban, pero lo hice acá mismo en la Unidad, me dieron opción para elegir y yo elegí acá, por una razón, porque aquí estaba el medico nefrólogo del que podía aprender mucho de él, y además somos amigos, me trasladé con él al segundo nivel para conocer más de la enfermedad renal y conocer los servicios de hemodiálisis y diálisis peritoneal.

-Ahora me gustaría preguntarle un poco sobre su función actual en el primer nivel de atención.

Bueno mi función a partir de enero de este año pasé a ser el coordinador de la unidad comunitaria Nuevo Amanecer que está dentro de acá de la especializada. Anteriormente hacía la función de medico consultante de la US Monseñor Romero, pero ahora soy el coordinador así que ahora tengo la función de dirigir a todos los profesionales sanitarios que trabajan en la US Nuevo Amanecer, que incluye enfermeras, promotores, farmacia y también el seguimiento oportuno de los pacientes en las diferentes etapas de la vida (edad pediátrica, adolescentes, adultos, adultos mayores, embarazadas, que éstas últimas son como decimos el pan de cada día pues hay que estar muy pendientes de evitar la mortalidad materno infantil), las promotoras son en esta parte de las embarazadas las más importantes pues son ellas las que andan día a día verificando donde esta una embarazada con alto riesgo, para inscripción de embarazos, niños de alto riesgo también, ellas verifican y tienen muy encuentra cuando es de alto riesgo para su seguimiento, ellas tienen su programación en cada comunidad. También hacemos la dispensarización de los pacientes en las distintas etapas de la vida que ese es un trabajo muy importante porque así sabemos qué personas son del grupo 1, del 2, del 3 o del 4 y en base a eso hacemos la programación de las salidas a terreno. Hacemos salidas a terreno dos veces por semana, allí hacemos visitas domiciliarias, inscripciones, controles infantiles de adolescentes, adultos y adultos mayores, vamos directamente a su hogar, martes y jueves. El resto de días de la semana hacemos consulta acá donde hacemos de todo, desde niños a adultos mayores, pasando por embarazadas y emergencias. Eso sería un poco lo que hacemos a parte de la parte estadística de los informes que hacemos para el Sibasi. Acá al ser una US especializada a veces tiene mucha demanda de pacientes, los que nos llegan de las diferentes unidades algunas referencias o los que vienen espontáneamente de otros lugares. En el caso de San Marcos Lempa nos llegan pacientes que creo que se debería mejorar allá la atención porque diariamente atendemos de 8 a 10 pacientes de allá y nos manifiestan que a veces pasa cerrado y por eso vienen aquí, y bueno esos días que salimos a terreno vamos a las distintas unidades y no hay consulta acá, pero cuando voy a terreno intento sacar todos los pacientes cuando regreso porque acá me están esperando, es por eso que el trabajo es bien fuerte.

-Y cuando vas a la casa de los pacientes, ¿con quién vas?

- Con la enfermera, la promotora porque ella es la que más conoce la casa de los pacientes y tiene localizadas las casas a ir a visitar ese día. La enfermera se encarga de tomar la presión tallarles y medirles, yo les hago ola historia clínica y la promotora tiene un papel muy importante visualiza los alrededores, como está el hogar, si hay hacinamiento si la yerba está muy alto que facilita la propagación de zancudos y roedores, cómo está el pozo si lo tienen si está tapado o no, si hacen uso adecuado del agua de ese pozo para lavar o también cocinan y beben de él, les orientamos sobre el uso adecuado del agua, también la promotora hace el rol de inspección haciendo la albanización y si hay algún criadero de zancudos también se le hace su control, y si encontramos algún paciente febril o con sospecha de dengue se hace control de los focos, en ese caso nos vamos todos juntos en campaña a hacer albanización en el foco para matar el huevecito de zancudo.

- Muchas gracias, ya me he hecho un poco la idea de tus funciones principales, ¿cuál es tu horario laboral?

Trabajo de 7:30 a 15:30 de lunes a viernes.

**Sección 2**: Comprender las trayectorias de los pacientes en el primer nivel de atención

-Muchas gracias, ahora me gustaría preguntarle sobre **su participación con pacientes con enfermedad crónica**

A los pacientes crónicos hacemos varias etapas, primero el diagnostico, que lo hacemos a través de una buena historia clínica, los paraclínicos que nos van a demostrar que estos pacientes tienen una enfermedad ya crónica. Para poner un ejemplo un diabético, que tenemos muchos acá algunos lo han desarrollado a temprana edad, nosotros aquí para poder hacer el abordaje de estos pacientes como a veces vienen ya cuando presentan síntomas les hacemos la historia clínica y le pedimos los exámenes que suelen salir con glucosas de 300-400 a veces también hacemos después la glucosa postprandial o la hemoglobina glicosilada que también tenemos acceso a ello para poder corroborar el diagnostico. Una vez corroborado el diagnostico le mandamos con la nutricionista quien le pone el plan de consumo de alimentos, también si vemos algún daño renal por la enfermedad le damos la referencia al nefrólogo. También le mandamos a la educadora social que contamos con ella aquí, entonces lo abordamos por varios aspectos, la parte psicológica también la abordamos porque hay algunos pacientes que son un poco rebeldes así que les mandamos con ella para que haga énfasis ahí. Los hipertensos pues son otra rama de pacientes, en ellos hay que ver algunos factores de riesgo que tienen como obesidad, consumo inadecuado de ciertos alimentos, consumo de sustancias toxicas como alcohol o cigarrillo que también es muy frecuente su consumo acá a temprana edad y también el afán que está ahora de la enfermedad renal crónica que a nivel nacional se ve que ha disminuido la mortalidad por ERC, pero ha aumentado el número de pacientes que la padece.

- ¿Y por qué ha aumentado la ERC?

- Porque ahora con todo lo de la reforma de salud y el esfuerzo que se ha hecho para una detección precoz de la enfermedad, han aumentado el número de diagnósticos, por eso es que, aumentado los pacientes, pero eso significa que estamos trabajando, antes se morían sin saber de qué.

- **¿Cuáles son los principales desafíos que enfrenta al abordar estos pacientes crónicos?**

-Uno de los desafíos es que muchos pacientes en realidad no aceptan que tienen la enfermedad porque no sienten ningún síntomas porque la ERC no da ningún síntoma hasta los estadios 3b o más, o empiezan a sentir calambres en las piernas porque les baja el potasio y se detectan por esa razón, porque trabajan muchas horas bajo el sol sin hidratarse y llegan con una espasticidad muy fuerte y vienen de urgencias, se les hace exámenes y se ve que tienen los iones alterados y la creatinina alta y es ahí cuando se les diagnostican.

- ¿Además de la exposición al sol y la deshidratación, consideras que hay más factores que influyen en el desarrollo de la ERC acá?

Se han dicho que hay muchos, pero los que tenemos claros son pocos, el que tenemos claro es la exposición a los agrotóxicos. Nuestra población es bien indisciplinada, sobre todo los más jóvenes que no se protegen y a veces hacen hasta competiciones a ver quién hace más bombadas, porque les pagan un dólar por bombeada, y eso lo hacen a veces sin camisa para no chorrearla sin ser consciente de que ese tóxico se mete en el cuerpo, a veces llegan incluso con intoxicaciones agudas por órganos fosforados. Quizás eso es una de las cosas más fuertes que tenemos, se les está dando la prevención y promoción de cómo deben usarse estas mochilas para bombear cómo usar los trajes, pero dicen, esto es muy incómodo así que no lo uso y como el daño es a largo plazo a veces no son conscientes, aunque a veces se refleja a corto plazo y vienen con espasmos musculares, a veces incluso remueven con la mano el veneno y al ir a comer no se lavan las manos a comer. Así que hay una indisciplina por parte del agricultor, pero a su vez hay muchas cooperativas que no les dan las recomendaciones ni les dan las cosas básicas que necesitan para poder protegerse, porque en las cooperativas sufren muchas veces de caída de sodio y potasio y eso se podría evitar dándoles suero cada hora incluso haciéndolo obligatorio, porque si no cada vez que suceda esto va a ver una lesión en el riñón que finalmente se convierte en ERC.

- **¿Qué estrategias usa para responder a estos desafíos en los pacientes crónicos?**

- Nosotros utilizamos varias estrategias, por ejemplo, lo que son las charlas educativas en las que a veces vamos hasta la casa con la psicóloga la educadora, la nutricionista, la promotora, la enfermera y yo para hacerles conscientes de todas las complicaciones que pueda haber sino se trata bien. Hacemos mucho énfasis en la alimentación y ahí la nutricionista le explica que con lo que tiene en casa puede hacer un balance, lo abordamos en conjunto con todo el comité interdisciplinario. Esto es una de las estrategias, en el caso de la ERC que ya está en estadios finales 4 o 5 estamos visitándoles cada 15 días en su hogar si todavía no ha aceptado a hacerse la diálisis peritoneal, conocen a algunos que han fallecido, pero no por la diálisis sino por las complicaciones y equiparan la diálisis a la muerte y les explicamos que eso no es siempre sí. Al final explicándoles de buena manera con paciencia, a veces incluso llevamos a pacientes en diálisis al hogar de pacientes nuevos para que le demuestre que lo que él piensa es erróneo y demostrando con cada testimonio de cada uno de ellos a la final cede porque se da cuenta que lo que estamos haciendo es para prolongarle más la vida.

**Sección 3:** Trayectorias de cuidado de personas con enfermedad crónica

• ¿Podría describirme los pasos que sigue un paciente crónico desde que entra en la unidad de salud hasta que sale?

**Primeramente,** cuando llega un paciente acá a la US con una enfermedad crónica, lo primero hacemos la **anotación**, en la anotación hacemos el **traje**, si viene mal de salud es el que pasa primero a la consulta, luego cuando ya pasa a **enfermería** le toma los signos vitales le pesa y talla, luego nos lo pasa a la consulta, aquí les hacemos un interrogatorio minucioso, si viene descompensamos indagamos en los factores que puedan estar descompensando la enfermedad crónica. Muchos nos comentan que tienen muchos problemas en su hogar o no tienen para tener una alimentación adecuada, o no están tomando la dosis correcta de medicamentos, si está teniendo algún problema renal como médico general les mandamos con referencia al nefrólogo. Aunque también puede haber **pacientes descompensados con hipertensión o diabetes que también le mandamos al nefrólogo para que le controle porque él también es de medicina interna, le modifica el tratamiento y le compensamos. Si él tampoco consigue compensarlo ahí si se le manda al hospital de Jiquilisco a medicina interna, si en el hospital no le resuelven les mandan ya a otros especialistas en el hospital San Juan de Dios en San Miguel**. Pero si es un **paciente bien compensado en mi consulta yo le hago la receta y va a la farmacia para sacar el medicamento y sacar nueva cita si es necesario.**

- ¿Podría describirme los pasos que usted sigue para llegar al paciente crónico?

La estrategia de nosotros, repito que las promotoras son los ojos de la unidad de todo lo que acontece en la comunidad, ellas son las primeras que nos comentan que hay una persona que puede padecer una enfermedad crónica, al saber eso, programamos una visita para ir al hogar de ese paciente. Le interrogamos le pedimos exámenes y le damos cita para que venga un día a la extracción y otro para ver los resultados con el médico, si los exámenes salen alterados y se confirma que es un paciente crónico ya se le da la cita siguiente para ir haciendo seguimiento y si era de grupo 2 en la dispensarizacion se le pasa al grupo 3 si lo amerita.

- ¿Siempre se les da cita a los pacientes crónicos con el resto de profesionales sanitarios (psicóloga, educadora, etc.) o solo si lo amerita?

-En la primera consulta siempre se les da la referencia para las otras compañeras del equipo y luego se les sigue según ellas lo consideren.

- ¿Cuéntame acerca de tu experiencia brindando apoyo para pacientes crónicos? No sé si me lo quiere explicar a través de algún caso concreto o cómo prefiera.

Bueno, ha sido bastante positivo desde que estoy acá, tenemos pacientes que en realidad son bien receptivos y en esos casos a pesar de que a veces no cumplieron a base de insistir e insistir han notado que ha mejorado su vida y para uno como médico eso es muy satisfactorio ver que el paciente se siente un poquito mejor. De los pacientes que tenemos casi un 80% ya saben mucho de su enfermedad, saben que medicamentos toman cuando tomarlo y todo, y luego el otro 20% pues está en aprendizaje, no podemos decir que es el 100% que están bien controlados. Pero por ejemplo cuando los ves ya en la comunidad en un parque en la cancha de fútbol haciendo una vida normal es muy satisfactorio al verle que en algún momento estaba en su casa en una hamaca prostrado y ver que sale es muy satisfactorio. Con el Dr. C. tenemos muchas anécdotas, por ejemplo, teníamos un paciente que estaba muy muy malito con una ERC muy avanzada y psicológicamente destruido, verle ahora que sale con su familia y todo y se hace la diálisis peritoneal ambulatoria eso nos anima a seguir trabajando.

- ¿Puede pensar en algún problema que presentan más frecuentemente los pacientes que realizan su seguimiento con usted?

- Quizás uno de los problemas es que a veces no tenemos algunos de los medicamentos que nos ayudarían a controlar mejor sus enfermedades o determinados exámenes que nos serían muy útil tenerlos acá y que ellos no pueden pagárselo fuera como los electrolitos que son muy frecuentes las descompensaciones de los mismos por la propia ERC y no se pueden hacer en la US hay que mandarles al hospital o a privado y eso es un gran limitante para nuestros pacientes por lo que estaría muy bien tenerlo acá.

• ¿Ha notado alguna diferencia en la forma en que los pacientes y el personal interactúan entre sí desde que comenzó este abordaje con la reforma, aunque usted lleve poquitos años trabajando acá?

- Bueno, desde mi punto de vista sí he visto que se ha mejorado, no al 100% porque nuestra población nunca fue educada de esa forma para tener contacto directo con el médico, incluso algunos se molestan de que lleguemos tanto a su casa y así, porque no están acostumbrados a tener ese servicio sanitario, pero es uno de los retos. Pero si se ha mejorado mucho el abastecimiento de medicamentos, consultas y exámenes gratuitos y eso les desahoga a ellos económicamente y eso sí que se nota que ha mejorado mucho aunque la población no sea muy consciente, le falta un poco la educación en la parte de la salud, no tenemos todo eso que decimos de qué bueno que tenemos la salud en nuestras manos y vamos a colaborar en todo lo que nos digan para que un día la población sea consciente de todo ello para que todos esos factores que desencadenan la enfermedad pudieran evitarse porque ese sería uno de los retos de la reforma. No es tan fácil que se diga porque a veces las condiciones higiénico dietéticas de las familias no son las adecuadas, pero como eso lo traen desde que eran niños no es tan fácil de cambiarlo de la noche a la mañana, pero ahí estamos, intentando inculcar lo bueno.

- ¿Cuáles son algunas estrategias que ha utilizado o que ha visto que han usado otras personas, para garantizar que el paciente demuestre una comprensión clara de la importancia del cuidado de su enfermedad ya sea en el hogar o en la consulta?

- Bueno una de las estrategias son las charlas educativas con medios audiovisuales, porque a veces les hablan y se lo imaginan, pero si les enseñan las imágenes se les queda mejor.

Además primeramente yo les doy la confianza, que soy un amigo de ellos, que quiero ayudarlos, que me ayuden hacer mi labor adecuadamente y ellos también pongan de su parte y trazarnos plazos por decirle en un mes yo quiero que usted mejore esta parte y hacemos el trato, a su vez les llamamos en grupos de familias para que se comprometan, pero de una manera dinámica pero hablando bidireccionalmente no poniendo como eslabones no, hacemos como un bostezo para que se comprometan, entonces yo les digo bueno en tantos días voy a pasar a ver si ha hecho a lo que se ha comprometido pero siempre dándoles la confianza, porque si les hablamos así muy rustico de medico a paciente vienen un poco restringidos o a la defensiva así que no hay que ponerse al a defensiva con ellos porque no se va a lograr nada.

-Comentaba anteriormente que hay pacientes más y menos receptivos, ¿podría comentarme porque cree que eso es así?

-Los pacientes menos receptivos son aquellos que nunca han podido asistir a la escuela, son analfabetos, están con los conocimientos que sus padres que también nunca estudiaron se mantienen, y cuando se les intenta cambiar la idea les cuesta. Por ejemplo, con la dieta, te dicen bueno, si mis padres no hicieron nada de dieta, ellos lo que comían eran tortillas frijoles sal tomaban café cuando les daba la gana y ahí murieron con 90 años y yo, que apenitas tengo 60 ya me quieren restringir, por eso con ellos cuesta un poquito más. Pero siempre uno tiene que sacar alguna estrategia para poder abordar a esas personas.

• ¿En ese sentido que recomienda como formas de comunicarse con esas personas con enfermedad crónica que comentaba anteriormente para ayudar a mejorar el manejo de su enfermedad?

-Primeramente, quizás acercarse a otras personas que sean más de confianza de ellos y que esa otra persona, como le escucha más orientar a esa persona y que ésta le oriente a él de manera más adecuada, después nosotros evaluamos como ha ido la cuestión y en realidad sí que vemos que funciona. Otra manera de comunicarse por ejemplo con los jóvenes era en la cancha de futbol, yo voy a la cancha y cuando terminamos el partido aprovecho a reunirles y les explico, miren esa enfermedad que padecen alguno de los compañeros, yo como amigo que estoy aquí con ustedes, pero también como médico, es animo a que vayan a la unidad a hacerse los exámenes, les digo que no tienen que pasar por la consulta 1ue les hago la boleta directamente y algunos han hecho caso la verdad.

- Ahora nos gustaría preguntarte sobre tus apoyos como personal sanitario cuando te encuentras con algún desafío tanto a nivel personal como profesional.

- Bueno cuando hay algún inconveniente o alguna cosa que nos falta yo se la pido al coordinador de la unidad de salud y él lama a otro nivel para ver si nos pueden ayudar en eso. Muchas veces el respaldo es inmediato, pero en otras ocasiones hay que esperarse un ratito, pero en general hay buena receptividad porque tenemos buena relación con los otros niveles. Y como las unidades especializadas son como un modelo a nivel nacional pues en general existe bastante receptividad. También con el médico nefrólogo/internista, con cualquier duda que yo tengo le llamo inmediatamente. Y por supuesto mi familia es un gran apoyo el más importante porque si en la casa estamos estresados no vamos a poder trabajar bien. Y en la comunidad con los líderes, los consejos comunales, los jóvenes que están organizados, aunque comparado con años anteriores hay muchos menos jóvenes por el problema de las pandillas. No es que yo colabore activamente en ninguna porque no me da el tiempo, pero si organizamos algo desde la US informamos a los líderes comunitarios y siempre nos colaboran.

**Sección 5**: Experiencias con componentes de intervención en torno a la estrategia de fortalecimiento de la capacidad instalada de las Redes Integrales e Integradas de Salud (RIIS), para mejorar su respuesta, acceso y calidad de la atención a las personas, familias y comunidad, reduciendo así́ las complicaciones y mortalidad de forma prematura causadas por las ENT.

- ¿Nos puede describir si existe algún cambio en los últimos 10 años en cuanto al manejo del paciente crónico?
- Sí ha habido bastantes cambios, la población muchas veces no los percibe con la misma magnitud porque siempre quiere más y más pero sí a nivel nacional sí ha habido hecho muchos cambios a nivel de salud en los últimos años. Ahora, que se quieren hacer muchas cosas en el caso de construcción de unidades de salud u otro tipo y eso siempre es bueno para reforzar el primer nivel de atención (el participante recibe llamada del coordinador de la unidad de salud para acudir a una convocatoria urgente así que decidimos acortar las ultimas preguntas de la entrevista).
- ¿Cómo consideras que ha sido útil la estrategia para el abordaje integral de las ENT propuesta desde el Ministerio de salud?
- Bueno, ha sido una de las estrategias claves quizás porque unas de las que elevan la tasa de mortalidad en nuestro país y en otros son las enfermedades crónicas cuando no se da un seguimiento y un control adecuado vamos a tener muchas muertes pero como la estrategia está encaminada a la prevención y promoción el abordaje en esta parte es muy importante y juega un gran papel en la estrategia vamos a evitar muchas muertes enfatizando la mejora de la calidad de vida de los enfermos crónicos y evitar que otros se enfermen, así que yo considero que está muy bien establecida porque vamos a la parte comunitaria que es donde están todos los factores que van a desencadenar todos los factores que desarrollan estas enfermedades, entonces sí, muy buena estrategia esta.
- ¿Y qué mecanismos se han definido para asegurar la disponibilidad de insumos, de medicación de equipo para el diagnóstico temprano de las ENT?
- Uno de los mecanismos ha sido la gestión con el Sivas la coordinación con los distintos niveles, la gestión es muy importante porque si no gestionamos parece que todo está bien y no siempre es así, estamos trabajando estamos viendo respuesta y son hay respuesta hay que hacerle gestión, pero a veces las gestiones son rápidas y si no hay que esperar y seguir gestionando. También ha habido donaciones de España por ejemplo que nos han abastecido de medicamentos y han ayudado mucho a la población.

- ¿Qué te pareció más difícil a la hora de implementar los documentos regulatorios para la atención de personas con ENT priorizadas?

- Uno de los desafíos es la falta de recursos humanos porque uno solo tiene muchas ideas, pero uno solo no puede abarcarlo solo y si lo abarca pues lo hace mal. Anteriormente en esta unidad por ejemplo había 4 médicos, un coordinador y 3 para dar consulta pero ahora, bueno ahora se ha incorporado otro médico hace 2 meses pero anteriormente a mi solito me tocaba pasar consulta, lo hacía pero era muy agotador y cuando es así las cosas no se hacen efectivamente y es por eso que hemos pedido al coordinador y él también lo ha pedido que nos manden más recursos humanos porque esta unidad es la de referencia y vienen pacientes de distintos sitios y cuando yo salgo a terreno por ejemplo, antes de venir este otro compañero, la unidad quedaba sin médicos porque el coordinador suele estar fuera en reuniones y temas de gestión. Entonces es por eso que para poder cumplir eficazmente todos los lineamientos entorno a ENTs yo sugeriría que pusieran más recursos humanos.

- ¿Tiene alguna pregunta para nosotras? Yo creo que por el momento ya está, muchas gracias.

## Staff interview 9: Family doctor

Entrevista con: Medica de Familia y comunitaria

Fecha: 13/06/18
Lugar de la entrevista: Unidad Comunitaria de Salud Familiar Perquin
Código del informante: SFMJ009
Genero del informante: F
Nombre del entrevistador: MJ

**Sección 1**: Perfil del participante y función en la prestación del servicio del primer nivel de atención

• Bueno, muchas gracias por acceder a la entrevista doctora, lo primero que me gustaría preguntarle es sobre su perfil, antecedentes, su edad, dónde se formó, cuánto tiempo lleva trabajando en la unidad de salud especializada, todo esto.

- Yo tengo 40 años soy formada en la Universidad de El Salvador mi pregrado fue en la Universidad de El Salvador y mi postgrado en el hospital José Antonio Saldaña en neumología y medicina familiar en los planes de Renderos en San Salvador. Antes de ser especialista en medicina familiar trabajé un año en el segundo nivel de atención como médico general en el hospital de residentes le llamamos aquí de Gotera, otros dos años en el hospital de Ciudad Barrios y 5 años en el hospital de Nueva Guadalupe viendo todas las áreas. Y desde 2012 aquí en el equipo especializado de Perquín. Tengo una hija, y me gusta la medicina familiar. La especialidad la hice en el hospital Saldaña en San Salvador. La especialidad es de 3 años. No sé muy bien cuando empezó la medicina de familia, tal vez más de 10 años, pero fíjese que pocos entran cada año, en el hospital que yo me formé entran 5-6 al año, siempre en el hospital Saldaña, antes el hospital seguro social Coatepeque en San Salvador había, pero ya la cerraron. Ahorita solo están formadoras de médicos de familia en el hospital Saldaña y el del seguro social, pero en el departamento de Santa Ana.

• ¿Y cuál es su función actual en el primer nivel de atención?

- Fíjese que nosotros aquí con la reforma de salud que ya hay especialistas la modalidad es de médicos internistas, pero también han aceptado médicos de familia, así que por lo general nosotros vemos solo adultos porque como también hay pediatra yo me dedico a los adultos. Realizo la medicina de familia con adultos. En realidad, en el primer nivel somos todos médicos de familia porque los internistas se quedan en el hospital de tercer nivel, pero lo que pasa es que en el país aún no tienen una visión clara de lo que somos los médico de familia aunque más sin embargo, somos la puerta de entrada al sistema sanitario, como en muchos otros países desarrollados, pero los que no nos conocen creen que somos médicos generales, pero cuando pasan la consulta con nosotros allí si se sabe la diferencia.

**Sección 2**: Comprender las trayectorias de los pacientes en el primer nivel de atención

• Cuénteme un poquito como es su día a día acá.

-Nosotros como un día normal los lunes y viernes trabajamos en la unidad de salud, pero martes miércoles y jueves visitamos las unidades de salud básicas intermedias de la región que son 13 establecimientos, hacemos una visita al mes a cada US.

Nosotros empezamos todos los días a las 7:30, si nos quedamos acá ya sabemos que tenemos nuestros pacientes agendados o alguna emergencia que venga. Pero si salimos a terreno nos desplazamos hasta la unidad de salud que toque, enfermos crónicos para medicina familiar, niños para el pediatra…y trabajamos pues hasta mediodía, luego hacemos un receso para comer y a la una seguimos hasta las 15:30hs, 8 horas laborales. Somos un **ECO especializado y nos desplazamos a las unidades básicas e intermedias, una vez al mes a cada una.**

- ¿Y cuando llegan a las unidades de salud, el paciente ya tiene cita previa o cómo?
- Sí, son pacientes que ya han sido valorados por el médico general y les da la cita con nosotros, aunque también si ese día les llega algo complicado que no pueden manejar también lo vemos ese día. Además de eso también les damos charlas, atención primaria ¿verdad? Hay un cronograma de que temas se van a dar, enfermedades crónicas, otro día de diarrea, vacunación, violencia si es el mes contra la violencia de género, temas específicos que se dan en los establecimientos antes de la consulta que ya están organizados. También la educadora se reúne con los diferentes clubes, también eso está programado, de adolescentes, adultos mayores, embarazadas, como el equipo está conformado por distintos profesionales pues cada uno ya sabe, aunque a las charlas vamos todos a todas y duran como 15-20mins, siempre damos esas charlas porque es parte de la atención primaria que se brinda, de la atención integral. Se trata de hacer lo más completo que se pueda para ayudarle a la gente a la vamos todas, menos ginecóloga que no tenemos ahora, pero vamos todas, la enfermera, fisioterapista, psicóloga, educadora, psicóloga y medica de familia, todas vamos.
- **Ahora sí me gustaría que me describiera un poquito más sobre su participación con pacientes con enfermedad crónica**
- Nosotros cuando evaluamos al paciente o cuando el médico general evalúa al paciente ya tenemos normado que al menos una vez al año tenemos que valorar al paciente con enfermedad crónica. Por ejemplo, si un paciente diabético que esta con insulina un medicamento delicado y está inestable, al tener yo la primera consulta con él, me quedo con él, lo evalúo, lo estabilizo, se le da educación de diabetes, en insulinoterapia, cuidados de los pies, bueno, ya sabes, y cuando está estable se retorna de nuevo al médico general. En un paciente hipertenso, lo mismo, una vez que se estabiliza, pues ya lo retorno. Pero también hay pacientes complicados que conllevan cuidados específicos de pie diabético o grados 3b o 4 de enfermedad crónica, pues ya esos los sigo yo siempre. El paciente se va citado, llega entrega su tarjeta, se le toman los signos y ya lo veo yo en la consulta.
- ¿Pero a los pacientes crónicos que ya se cita usted los ve en sus comunidades o en la unidad de salud especializada de Perquin?
- A los pacientes crónicos d otras unidades de salud los veo allá, pero por ejemplo pacientes cardiópatas o fabricadores, que requieren de seguimiento electrocardiográfico, les hago el chequeo si ameritan control medicamentos con digoxina o amiodarona eso se lo hago aquí en la consulta de la unidad de salud especializada de Perquín, pero si solo es electro de control nuevamente retorna al médico general.
- ¿Y por ejemplo un paciente diabético, bien controlado en su unidad de salud (básica o especializada), en algún comento también le ves tú?
- Siempre, una vez al año chequeo a todos los pacientes crónicos. Solo que yo tengo un problema, siempre luego se quieren quedar conmigo, ya no se quieren retornar más con el médico general de su unidad, pero bueno eso es siempre igual la educación de la gente, ¿verdad? Que ellos comprendan que eso da un chance para que se pueda ver también a otras personas en la consulta.
- ¿Y en el caso de la enfermedad renal crónica un estadio 4, lo sigue usted?
- Si bueno, ahí se le hace la derivación para el nefrólogo, pero eso es en el tercer nivel en San Miguel, pero no nos desprendemos nunca del paciente porque estamos pendientes de sus vacunas, que si está tomando el medicamento, que si está siguiendo las indicaciones del nefrólogo, igual nosotros les chequeamos aquí. Igual un paciente que está ten terapia sustitutiva, o se la hacen en San Miguel, o les dan la DPSA y el seguimiento siempre se lo damos aquí.
- ¿Y un paciente nefrópata, por ejemplo, en estadio IV, cuanto tiempo pasa desde que usted le hace la derivación hasta que le ven en el hospital?
- Bueno, como usted sabe, nosotros los médicos de familia tenemos un hasta aquí, cuando ya no se le puede dar el medicamento por ejemplo o es una enfermedad de difícil control, si es una emergencia se le manda al hospital de Gotera y ahí ya le ven de un solo. Peor si hay que mandarle a un subespecialista, un endocrinólogo, internista, nefrólogo del hospital, ahí depende…es otra política que tienen en el hospital. En el hospital por ejemplo si es un paciente renal, ahí tienen toda un área para ello, ahí le valora un internista y si él considera que para él amerita que siga siendo evaluado ahí, ahí queda la evaluación del paciente. Pero sí no, nos manda una contra referencia. **Están tardando como unos 4-6 meses en dar la cita con el nefrólogo y los demás especialistas del hospital desde que yo les hago la referencia desde el primer nivel**.
- ¿Y esa otra contrarreferencia desde el hospital le llega fácilmente? Porque nos explicaron en el SIbasi que les llega a ellos primero.
- Por lo general la contrarefererrencia viene con el paciente e o el promotor lo busca en la comunidad se revisa la hojita y les dice pues vaya el lunes o cuando sea, por lo general 48 hs tras darles el alta tienen que venir. Igual que nosotros cuando vienen a la unidad de salud especializada, les damos la contrarreferencia al médico general y les decimos que se la entreguen lo antes posible.
- ¿Y qué tal función en la realidad?
- (Risas) Sí, porque esto que te contaba es en teoría, lo ideal, lo bonito, **pero la realidad es que la contrarreferencia desde el hospital por lo general no llega**. De 20, llegan uno o dos. Esa es la realidad.
- ¿Cuántos pacientes suele ser usted de media al día?
- **El límite de ver son 24- 25 al día** entonces algunos días se ven 20, 22 va dependiendo así, a veces 24 o hasta 26 va dependiendo, pero depende del establecimiento de salud, porque luego hay algunos establecimientos de salud que son bien pequeños e igual ven a 10-12 pacientes al día. Pero acá 20-22 pacientes al día estamos viendo.
- ¿Y cuánto tiempo tienen por paciente?
- Como eso ya está normado, son **15 minutos por paciente,** 4 pacientes a la hora, pero vaya si por ejemplo un paciente diabético que le tengo que introducir en insulinoterapia por primera vez, hasta una hora me tardo con ellos, no es un tiempo pues que uno pueda decir ya pasaron los 15 minutos le voy a decir, ¡vete! No **si necesita más tiempo se le da el tiempo.**
- De esos 20-22 pacientes diarios doctora, ¿cuáles de ellos son enfermos crónicos?
- La mayoría, fíjese que yo solo casi pacientes crónicos veo, adultos y adultos mayores, nefrópatas, artritis, diabéticos, epilépticos, hipertensos, de todo.
- **¿Cuáles son los principales desafíos que enfrenta al cumplir su función?**
- **Que el paciente tenga sus medicamentos, porque en muchas ocasiones hay desabastecimiento**, o **la poca adherencia** al medicamento por parte del paciente, porque hay algunos que le dicen a una, ay si si, y luego llegan al siguiente mes igual el azúcar o más alta e igual la presión, entonces esa sintonía es el desafío a veces.
- **¿Qué estrategias usa para responder a estos desafíos?**
- Con la falta de medicamentos es bien difícil porque nosotros solo podemos reportar que no hay y en alguna ocasión si es mucha mucha la necesidad que tiene el paciente, porque hay gente que para venir a qui caminan 4- 6 horas para venir hasta aquí, entonces nosotros vamos buscando por los establecimientos, porque los conocemos a todos y decimos mire en tal lugar hay medicinas y se las van a despachar. Y luego para lo otro lo de siempre, la educación, la empatía con el paciente yo si la tengo gracias a Dios, pero para lograr el objetivo de que se adhiera a los tratamientos, con educación educación para la salud, esa es la clave, explicarles a los pacientes sobre sus tratamientos su enfermedad.
- ¿Cuándo reportan desabastecimiento a quien lo reportan?
- Al coordinador de la unidad de salud especializada, por lo general siempre buscan. En las farmacias cada cierto tiempo tienen que pedir medicamentos, pero no siempre llegan cuando se necesitan porque lleva su tiempo y ahí es donde se demora.

**Sección 3:** Trayectorias de cuidado de personas con enfermedad crónica

• Ahora me gustaría preguntarte sobre los pasos que sigue un paciente crónico desde que entra en la unidad de salud?

Bueno, viene el paciente, si nunca ha estado ante al establecimiento de salud pasa al **área de archivo** para que se abra sus expedientes de ahí les ve la **enfermera** le mira sus signos vitales, lo prepara, le mide y le pesa, presión, temperatura y los llevan al área de espera.

En mi caso como mis pacientes como son **citados** vienen en horario y van viniendo escalonados y funciona igual, les ve la **enfermera**, toma signos vitales y ya pasan directamente a mi consulta les evalúo veo si tenía exámenes, les doy su consejería, sus medicamentos y si el paciente nunca ha sido visto con la nutricionista o educadora social, se intenta que les vean ese mismo día de una sola vez, porque algunos pacientes vienen de lugares muy lejanos, entonces nosotros, el paciente viene por la doctora y ya después decirles que vengan otro día para que la ve la fisioterapista o lo que sea siempre es difícil que vengan por eso es que se aprovecha siempre esa primera consulta ya empieza a trabajar desde entonces, desde ese momento Si es diagnóstico de enfermedad crónica por primera vez, ya ese mismo día también recibe **atención psicológica**, por lo mismo, porque la mayoría de las veces os pacientes lo primero es la negación verdad igual al saber que van a tomar medicamentos de por día,, así que como no es fácil es por eso que se brinda la atención, así que **por lo general son 4 consultas que recibe ese día**, después va a **farmacia** a recoger los medicamentos, luego pasa con **la enfermera** para ver la cita de la próxima consulta, si va tener que hacerse exámenes, chequea las vacunas y posteriormente el paciente **se va**. A veces, como se intenta hacer lo más integral posible, son varias las especialidades verdad. la gente a veces dice que echan acá el día, pero la verdad es que es muy provechoso porque antes no teníamos eso, antes se conseguía la cita con el especialista en un año, y si ese día conseguía el paciente tener dinero para el transporte que mala suerte el especialista estaba incapacitado ese día no le veían y se regresaba sin ser atendido. Si han sido muchos los obstáculos superados gracias a esta reforma integral en salud y yo siento que eso le beneficia a la gente.

- Y por lo general, estos otros especialistas (nutricionista, ¿etc.) también les vuelven a ver?
- Sí, les dejan chequeos, y por lo general hacen que coincida con la fecha que tienen la consulta de la enfermedad, la educadora, nutricionista y el psicólogo. Nosotros aquí en esta zona es donde se dio el conflicto armado y tenemos muchos veteranos de guerra y todo el estrés que les pudo haber acarreado, por varios años que han pasado, todavía existen secuelas psicológicas, en general a toda la ve es bien raro que haya alguno que no, y en ese caso es requisito que le vea.
- ¿Y al médico general normalmente van sin cita?
- Sí con ellos es por orden de llegada. En ese caso si esta con una emergencia le atendemos el mismo día o nos manda la referencia.

• ¿Podría describirme los pasos que usted sigue para llegar al paciente crónico?

Como nosotros ahorita en los ecos se está trabajando con el diagnostico de salud aquí todas ases viviendas se les pasa fichas familiares y de esta manera se dan cuenta cuantos hipertensos cuantos diabéticos, porque también hacen acercamientos o visitas a las comunidades si ellos allá detectan algún problema ya deciden si mandarlo de urgencia o esperar a que lleguemos Alos establecimientos de salud. Justo en la zona de parquin aun no lo tenemos. Los primeros son los promotores que lo hacen, igual nosotros si tenemos un paciente complicado de difícil control se lo dejamos anotado en su informe y el promotor lo ve para hacer la visita a domicilio y verificar cómo se está haciendo. ¿Es un trabajo coordinado verdad?

- ¿Por qué usted también acude a las casas?
- Sí acudimos a las casas. Por ejemplo, algún paciente encamado o que no se puede movilizar fácilmente también se va a visitar a las casas no es lo general, pero en esos casos sí se hace, también en el caso de enfermos terminales, pacientes artríticos que ya no pueden deambular. También se van a ver a sus casas.
- ¿Porque en el caso de cuidados paliativos, también lo llevan ustedes?
- Sí, nosotros no tanto los médicos generales. Pero igual para un cáncer medidas de sostén higiénico dietéticos se explican a las familias, pero así un protocolo de paliativos no hay, pero ya se está trabajando en ello.
- ¿Cuéntame acerca de tu experiencia brindando apoyo para pacientes crónicos? Por favor, denme un ejemplo reciente y cómo se realizó el seguimiento del paciente, ¿cuáles fueron los problemas concretos abordados?
- Si siempre, por ejemplo, aquí y en todos los establecimientos si hay pacientes por ejemplo 3b ya se por cronograma que le tengo que ver cada 3 meses, o un paciente diabético también tengo estipulado cada cuanto tengo que verle, igual un paciente hipertenso que ha tenido que ir al hospital por una crisis hipertensiva, yo cuando vuelve ya le veo. Y es lo que le decía la paciente, paciente citado es fiel, esa noma falla, salvo que se le haya muerto algún familiar o no haya habido transporte o algo, pero en general son bien fieles.

• ¿Qué tan factible es brindar un mejor soporte a los pacientes que lo necesitan?

- Bueno, yo siento que una vez uno se adapta al sistema, pero tanto para nosotros como para ellos, por ejemplo, vienen enfermos que me dice: “Mira llevo así desde el sábado, pero como sabía que tenía cita con usted ya me esperé” pero eso ya es la educación sabe que lo más prudente es que venga a pedir ayuda ese fin de semana, pero sí, ósea, **la continuidad del tratamiento** es bien fieles. Vienen siempre vienen.

• ¿Puede pensar en algún problema que presentan más frecuentemente los pacientes que realizan su seguimiento con usted? ¿Notó alguna diferencia entre estos y sus otros pacientes?

- Las **etnopracticas**, porque en muchas ocasiones, este que la vecina le dijo que está tomando unas cascaditas amargas de té, algunos están tomando algún tipo de medicina alternativa o natural y a veces si interfiere en el tratamiento. Eso sí que es bien común aquí, que los pacientes, buscan otras alternativas, entonces y este no son todos, pero esos son los que se complican, por ejemplo, los pies diabéticos en muchas ocasiones le es tan difícil desplazarse a asan miguel por una complicación, que igual se curan en casa con cascarita de algún palito. Y eso sí que nos complica a veces. Acá no es muy frecuente porque los pacientes que lo hacen suelen dejar el tratamiento y se complican.
- ¿Ha notado alguna diferencia en la forma en que los pacientes y el personal interactúan entre sí desde que comenzó este abordaje con la reforma?
- Sí, bastante, la empatía que se crea es como que el paciente ya tiene su médico especialista o su médico de cabecera, a veces me traen las medicinas para que yo las vea, fíjese que me las mandaron. Aunque el médico general en las comunidades también tiene continuidad de la asistencia por supuesto.

**Sección 4**: Estrategias en el manejo de pacientes crónico

• ¿Qué tipo de problemas o desafíos surgen en el manejo del paciente crónico? ¿Cómo manejas estas situaciones?

- Fíjese que la mayoría de pacientes que yo tengo, son adultos mayores, entonces yo lo que les pido, es que vengan siempre con un familiar, que sea la persona responsable la que le de comer le cuida, el que pasa con ellos. Porque si vienen solitos a veces te dicen que sí entendieron y cuando llegan a la casa ya no se acuerdan. Por eso yo siento que para mí es un pilar muy importante la familia que le ayude y cuide y esté involucrado en su enfermedad.
- ¿Y suele ser así o hay también soledad en ancianos?
- Pues no suele sin muchos, por ejemplo, hoy de todos los que vi en la consulta, solo uno vino solo, no es mucho pero sí también se da a veces que viene solito el paciente claro.
- ¿Y cuando viene solito?
- Ahí echo mano de la unidad de salud de donde viene, para que sean ellos los que estén pendientes de que está tomando bien el tratamiento, cumpliendo bien las indicaciones, todo. Normalmente a los que vienen solos me los cito bien prontito la próxima consulta para ver si están haciendo lo que se debe o no. Por ejemplo, los hipertensos ver cómo van a haciendo y así.
- ¿Y cómo hace para que le entiendan bien la información distintas tipas de pacientes?
- Les explico con los medicamentos con sus horarios e indico cómo los va a tomar, también les pido que me repitan que es lo que les acabo de decir para comprobar que han entendido bien. Igual con las insulinas, les doy la jeringa, por ejemplo, les digo, ¿por la mañana son 20 UI las que se va a poner, hasta dónde carga la jeringa? Entonces el paciente se fija o donde se tiene que pinchar…es bien difícil pero siempre hay alternativas porque por ejemplo se les pregunta si el promotor pasa todos los días, pero como siempre siempre hay una vecina que sabe inyectar y vive en frente, siempre buscamos la alternativa para así antes que ellos reúnan el valor de poder inyectarse ellos buscamos la manera para que le puedan poner el tratamiento como se debe.
- ¿Puede proporcionar algunos ejemplos de casos en los que sintió que un paciente era más receptivo a la información que le estaba proporcionando? ¿Menos receptivo? ¿Por qué crees que es esto?
- Siempre hay pacientes que a veces se les olvida, por ejemplo, un enfermo renal que le tengo que cambiar reclasificar, le cito con los exámenes, se me perdió la hojita de laboratorio, o que usted no me dio la boleta del examen, siempre hay una excusa, o su hija se los quedó o que se han olvidado las medicinas.
- ¿Por qué crees que es?
- El nivel educativo siempre influye y contra eso no podemos luchar nosotros porque no podemos mandar a estudiar a un hombre de 80 años, eso ahí va a estar, lo que procuro es tomarme el tiempo para explicarles, igual con la alimentación, ósea, yo les explico que, con la sal, el azúcar los carbohidratos, todo comparado con determinados alimentos que conocen, pero luego el refuerzo lo hace la nutricionista. Ajan, yo me tomo mi tiempo, pero también son todos sus refuerzos, por ejemplo, la educadora también les pregunta que les dijo la doctora y como tienen el expediente ellas saben lo que tiene y comprueba si lo ha entendido o no, así que son 3 refuerzos los que recibe.
- ¿Cómo realizan los refuerzos los especialistas de la unidad?
- Pues depende, por un lado pacientes citados con ella decide cada cuanto verle o yo por ejemplo si le veo que es un paciente renuente, él o su familia o que no les vuelvo a mandar para refuerzo, pero por ejemplo la nutricionista sin son pacientes que sigue ella les va citando y va valorando estado nutricional, o por ejemplo igual con la psicóloga si tienen la necesidad, por ejemplo pacientes que vienen a hablar de los problemas que tienen en la casa, ahí se le refuerza con el psicólogo. Fíjese que por lo general intentamos atender al paciente en la especialidad que necesita ese mismo día, pero por si lo que sea no está se les cita lo antes posible en ese sentido sí que somos bien asequibles.

• ¿Qué recomiendan como formas de comunicarse con persona con enfermedad crónica para ayudar a mejorar el manejo de su enfermedad?

- Fíjese que aquí aparte de que se les da la atención y todo se dan charlas, se forman clubes, para que la gente esté informada, del diabético del hipertenso del adulto mayor, rehabilitación, hay medios de comunicación, radios y canales de televisión, ellos por lo general vienen a pedir algún tema que se brinde y eso puedo ayudar a gente que está en la casa y pueda hacer consciencia de la enfermedad y acudan a la unidad de salud. Yo sí he participado en alguna ocasión en la radio comunitaria, pero sí siempre se busca la estrategia.
- ¿Los clubes cómo funcionan?
- Pues como ya se tiene a los enfermos crónicos, ¿verdad? Les dicen tal día va a ver club y ya ellos vienen, dependiendo de la enfermedad que tenga ahí se citan, también suele estar la educadora social, si va a dar una charla la nutricionista o fisioterapia, si funciona, por lo general una vez al mes, igual en los demás establecimientos de salud.

• Ahora me gustaría preguntarte sobre tus apoyos como personal sanitario cuando te encuentras con algún desafío.

- Bueno aquí esto para cuidar al usuario interno que somos nosotros, nada (susurrando), ahorita está en plática que vamos a formar clubes para jugar futbol o softball en las canchas de acá, pero hasta ahorita hemos formado los equipos, pero aún no se comenzó nada. Pero bueno, cuando nos desplazamos porque no solo se habla es malo, en las salidas de campo, yo si ahí noto que nos reforzamos porque contamos con vehículo propio, antes no, de las unidades de salud nos venían a traer entonces si ese vehículo en el transcurso de la mañana había una emergencia o algo y se tenía que ir a Gotera, entonces nosotros nos esperábamos, hasta que llegara de regreso a veces qué 4 de la tarde y nosotros estábamos ahí donde estamos cerca allá del fin del mundo. Entonces si siento que ha sido una ayuda.
- ¿Por qué ese transporte propio para el ECO especializado se movilice a las unidades de salud intermedias y básicas, desde cuando lo tienen?,
- Ya hace como unos tres años y yo eso siento que no tenemos en ese sentido problema, ha sido una fortaleza cimiento, saber que te vas y vas a poder regresar a tu hora, pero de ahí de auto cuido, más bien difícil, ya uno lo hace personalmente, los fines de semana con la familia tomarse un espacio para disfrutar con ellos con los amigos, pero eso ya es bien personal no sale de aquí.
- ¿Y en qué tipo de vehículo van?
- En un pick-up, vamos todos, nuestras caderas ya no lo resisten, pero ahí vamos, y cuando llueve todos en la cabina hasta 10, y aquí llueve todos los días en invierno y llueve.
- ¿Y con el equipo, aunque no tengan un tiempo estipulado de auto cuido, considera que lo hay?
- Si, siempre, en las comidas. Salimos al cafetín a comer a ese de afuera, yo ando cargando mi comida, pero bueno ahí vamos, en la unidad de salud no hay un espacio físico para comer todos, pero siempre se busca. Aunque por lo general yo cargo la comida y suelo comer sola en la consulta, como por lo general soy de las ultimas en terminar la consulta ya todos han comido.
- ¿Y existen espacios comunes en la unidad?
- Esta es la sala para reuniones y eso, pero no para comer, esta sala de reuniones es el único espacio común de ahí no tenemos nada más.

**Sección 5**: Experiencias con componentes de intervención en torno a la estrategia de fortalecimiento de la capacidad instalada de las Redes Integrales e Integradas de Salud (RIIS), para mejorar su respuesta, acceso y calidad de la atención a las personas, familias y comunidad, reduciendo así́ las complicaciones y mortalidad de forma prematura causadas por las ENT.

- Cuénteme su experiencia en cómo se han fortalecido sus competencias para la promoción de la salud y el abordaje integral de las ENT ¿Qué es lo que le resulta más y menos útil de esta estrategia?

-Pues fíjese que ha beneficiado porque todos hablamos el mismo idioma, uno ya sabe cuándo un médico general tiene que referir, o yo cuando les tengo que hacer referencia a otro niel de atención, es mejor sí. Pero fíjese que el martes pasado me llamaron para validar, formamos un equipo para validar el plan nacional de enfermedades crónicas, el ministerio está trabajando en ello y no es por nada, pero está quedando muy bonito. Así que siento que si se tiene ese apoyo, que todos hablamos el mismo idioma, tener protocolos de las distintas enfermedades para que uno no pueda decir a no lo manejo mal, porque ya sabemos que es lo que tenemos que hacer con los pacientes. Los protocolos solo crean a nivel central realiza el Instituto Nacional de la salud, la dra. Dubon forma parte de todo ese equipo, ellos hacen los protocolos de el mismo país, esta semana se reúnen con nosotros, la siguiente con cardiólogos y todos los subespecialista, todos los validamos y podemos quitar cosas que no nos parezcan, está muy bien. Yo ya he evaluado de pediatría, de enfermedad renal y ahora de enfermedades no transmisibles.

- ¿Cuentan con protocolos sobre enfermedades crónicas para el primer nivel de atención?

- Si como Ecos tenemos protocolos tanto de prevención como tratamiento, dese antes de los lineamientos de 2017 pero se han ido evaluando, los ECOS están desde hace 7 u 8 años, desde entonces, los han validado 2 veces, ahora ya van por la tercera validación.

Pero también existen debilidades porque a veces los médicos generales con un paciente diabético si yo no estoy les dan insulina o a hipertensos les dan ARA II si yo no estoy porque no estoy aquí todo el tiempo. Pero ya en pacientes con amlodipino o digoxina, esos i ya tengo que validarlos yo, porque así en el país no está esa costumbre de que uno va a respetar la…le voy a poner un e ejemplo, en un establecimiento de salud se acabó el enalapril, y les dieron atenolol, siempre con ese pensamientos de darles todo lo que haya pero en esas unidades habría que revisar los criterios para darles ese medicamento porque en algunas ocasiones terminan bloqueados, así que en ese sentido si creo que estamos fallando, también **tenemos las debilidades** que tenemos, no todo es de color de rosa, tenemos debilidades.

- ¿Sabes si los visitadores médicos de industria farmacéutica pueden visitar los establecimientos de salud?
- No eso está prohibido, porque como nosotros supuestamente el acceso a la salud es gratis y es por eso que tenemos que prescribir de nuestro cuadro básico, y podemos ser sancionados sino, hasta ahora no se ha sancionado a nadie, pero eso de que vengan no, ya no. Hace 10 años si venían, ahora ya no, en ese sentido **con los medicamentos siento que es una debilidad**, porque cuando se acaban los colegas generales les dan lo que hay aunque no esté indicado para ese paciente, eso sí siempre se sabe dar, o ahora por ejemplo con el uso de glibenclamida frente a metformina, algunos todavía les ponen la glibenclamida de primera elección y cuando yo les veo les pongo en la caja ”No glibenclamida” para que entiendan, o con las dosis de medicamentos. Lo malo de aquí es que alguno como que se encajona o enfrasca y el no actualizarse nos hace no ver que tenemos más opciones, o a veces educándoles, fíjese que yo con la enalapril les explico lo de la tos, que si les molesta mucho yo se la cambio pero que le protege los riñones etc. y es educarles, es negociar con el paciente, no voy a darle otro medicamento solo porque el que necesita no le haya.

- Y con el tema de actualizarse, ¿se les brindan a ustedes desde el ministerio programas de formación o acceso a base de datos?

- Por lo general los que ese está actualizando son los coordinadores, por ejemplo, actualización de tuberculosis o cosas así, lo lógico es que se baje la información, y eso no se suele hacer, no solemos tener reuniones conjuntas, es bien raro, pero como le digo siempre hay el ministerio da esas cosas, pero no todos vamos.

- Hay alguna organización de medios familiares?

- Acá no, pero en San Salvador sí. Bueno hay una a nivel nacional sí, no me acuerdo del nombre del doctor que lo lleva, pero si hay.

• ¿Me puede describir si existe algún cambio en los últimos 10 años en cuanto al manejo del paciente crónico?

- La reforma de la salud, es lo más importante, el acceso a las diferentes disciplinas en el primer nivel de atención que antes no se contaba con eso, incluso en los hospitales de segundo nivel hay mucha más tecnología que antes no había, antes si se tenían que hacer un TAC o Resonancia tenían que ir a un sitio privado y ahora se les hace allí, no es que se lo vayan a hacer al día siguiente que a veces hay que esperar pero se van haciendo las cosas.

-Y ahora que se refiere a la tecnología, ¿con que material se cuenta en las unidades de salud?

- Todos tenemos aparatos de ultras, solo que por el momento se utilizan para embarazados, yo tengo mi aparato de electrocardiograma. Antes eso no existía. Para la ultra se utiliza más que todo para los embarazos, no nos dan formación específica en ello al menos que tengas un curso pagada tú. Para mí lo más difícil fue acercar los equipos especializados al primer nivel de atención, incluso el trato con la gente también ha cambiado, también tienen más acceso a la información, y eso puede ser también un arma de doble filo, peor yo pienso que educando a la gente, dándoles la información.

• ¿Qué te pareció más desafiante a la hora de implementar los documentos regulatorios para la atención de personas con ENT priorizadas? ¿Menos?

- Pues la verdad es que no lo que yo si siento en lo personal, es el acceso, porque yo vivo en san miguel y vengo todos los días y eso sí que desgasta el transporte, pero si a uno le gusta su trabajo pues compensa. Y bueno pues que no se supla a los colegas que ese enferma o embarazos, o las vacaciones nunca los suplen. Somos los que no tienen voz. Bueno precisamente para eso estamos aquí (risas)
- ¿Crees que la reforma de salud se va a mantener?
- Pues debería mantenerse, porque el beneficio es para la gente y en la medida que se mejore el analfabetismo que se radiquen o controlen las enfermedades por qué. ahora estaba escuchando a una enfermera dando una charla y es cierto, “es más barato prevenir que curar “entonces en la medida en que los que dirijan la nación estén conscientes de ellos vamos a seguir haciendo nuestro trabajo, en l atrinchera que nos toque ahí vamos a estar haciéndola ojala que sí se mantenga porque salir del subdesarrollo, aún falta no, pero en la medida en que la gente se empodere de su salud, ahí vamos a salir del subdesarrollo en el país en el que estamos.

Yo me forme con una maestra cubana la doctora Delis pestorejo con ella usábamos el EMGI de medicina general de Cuba, que Cuba es muy bueno para diagnóstico y todo, pero para tratamientos, que hacíamos ver los artículos de España, y de ahí es que se quedaba un poco obsoleto el de cuba, a mí el enfoque de la atención primaria de espada o de Canadá que el de Cuba.

## Staff interview 10: Nurse

Entrevista con: Enfermera

Fecha: 13/06/18
Lugar de la entrevista: Unidad Comunitaria de Salud Familiar La Palma
Código del informante: SFML008
Genero del informante: F
Nombre del entrevistador: ML

**Sección 1**: Perfil del participante y función en la prestación del servicio del primer nivel de atención

• Perfil breve del participante (antecedentes, capacitación, historial de participación en la unidad de salud o ECOS)

- Me gustaría preguntarle ¿qué edad tiene, su formación, desde cuándo es que trabaja acá en la unidad?

- Yo hace 22 años, a los 20 años de la edad había recibido mi técnico en enfermería en la unidad de salud, yo me gradué e hice mi servicio social acá y luego apliqué para una plaza y aquí estoy desde hace 22 años, ahorita tengo 43 años. Las experiencias han sido muy bonitas, uy diferente cada año porque cada vez van hacienda reformas para abordar a los pacientes a veces con más recursos y a veces con menos. EN la nueva reforma la verdad es que ha sido muy bonito porque estamos brindado mejor atención al paciente, ahora contamos con especializada, yo estoy con los especialistas ahora pudiendo dar atención pediátrica fisioterapia ginecología. Que hace más de 6 años no contábamos con eso, y como acá las distancias son largas, ya no tienen que desplazarse al hospital y las personas han sido beneficiadas con esto. Las enfermedades crónicas están siendo tratadas por medicina interna, el medico de medicina familiar pacientes con diabetes, tensión alta, insuficiencia renal, troilismo que contamos con todos los medicamentos aquí, el médico le hace la **receta repetitiva** para que puedan recogerlos cada mes y él le cita cada 3 meses en consulta dependiendo de si el paciente se encuentra estable, sino se le ve cada mes o 1cada 5 días según lo amerite.

El servicio social yo lo hice en un cantón acá en la montaña en un dispensario que había sido donado por Luxemburgo, fuimos a in concurso y ahí me dieron la plaza. Si había 5 plazas ahí nos evalúan y me dieron la plaza, trabaje un año allá arriba y luego ya me destinaron acá a la unidad de salud. Antes era mucho más pequeño, muchos menos médicos y enfermeras y todas las personas de los cantones se desplazaban aquí a la consulta y controles, en ese tiempo sí que tenían que pagar, había un patronato que decía cuanto costaba cada cosa por ejemplo inyección cuesta tanto etc. y teníamos in talonaria para cada cosa, era una donación simbólica la cuota voluntaria. Se dejó de pagar hace 10 años.

• Su función actual en el primer nivel de atención

Nos cambian las asignaciones cada 2 meses, tenemos horario ampliado de 7 am a 7 pm y luego se lo pasamos a Fosalud. Somos 3 enfermeras que vamos rotando horario, dos de 7 a 3pm como yo ahora y la otra de 11am a 7pm para asegurarnos que se cubra todo el horario. Y no dejar en ningún momento descubierta la unidad de salud. Yo entro a las 7 si nos deja algún paciente fosalud me encargo para enviarlo al hospital o lo que haga falta. La unidad de salud abre a las 7:30 am y muchas veces ya tienes a gente sentadita ahí desde mucho antes, más que todo los señores que no duermen bien. Nosotras recibimos las tarjetas del paciente con un número que se le ha elaborado donde se indica a qué especialista va a consulta. Nosotras somos una enfermera por cada 3-4 especialistas así que nosotras llegamos y recibimos las tarjetas, nosotras les organizamos según los números que le ha dado el vigilante al llegar, así que van siendo atendidos en especializada según orden de llegada siempre y cuando no necesite ser adelantado por su enfermedad y entra en urgencias y se acelera su procedimiento para que sea atendido cuanto antes. Los pacientes de especializada ya han sido visto previamente y traen consulta agendadas y vienen de otras unidades básicas o intermedias de otros lugares con una **referencia retorno.** Por ejemplo, el médico general de San Ignacio o Las Pilas le hace una referencia al médico familiar de acá porque tiene una hipertensión descompensada, el médico especialista le ve acá y decide el tratamiento y se lo envía de Nuevo al médico general y le dice cuando le vuelve a ver si lo amerita sino el médico general le sigue el tratamiento si está estable.

Aquí también trabajamos con el ECO urbano consulta general, si por ejemplo llega in paciente por primera vez, el medico que entra a las 7 también le evalúa en el traje para ver qué tan grave está, se prepara signos vitales y el medico prescribe lo que haga falta, en caso de urgencias y consulta de medicina general. Se les clasifica por colores, rojo Amarillo y verde según su gravedad según indica la reforma para atender antes a quien más lo necesite. Si hay un paciente que necesita curas o lo que haga falta yo me movilizo donde haga falta incluso al hospital si es necesario en la ambulancia y la otra enfermera se queda en la unidad de salud. Si no tenemos ambulancia pedimos ayuda a la alcaldía para que nos ayude.

En el primer contacto hacemos la charla informativa en la sala de espera y explicamos qué enfermera va a recibir a qué pacientes según el médico especialista que tengamos adjudicado ese día, esa charla la damos todos los días donde también les explicamos que primero les vamos a tomar las medidas y signos vital luego le verá el medico luego a farmacia y luego a archivo para recoger la próxima cita. EN el horario de 3 a 7pm solo hay un médico y una enfermera, ahí nos volvemos multiusos porque no tenemos archive ni farmacia así que nosotras misma les buscamos el expediente y les dispensamos la receta (porque la farmacia queda cerrada, pero nos dejan in stock para poder dispensar a los pacientes en ese horario ampliado hasta que llegue Fosalud). Si llega una emergencia a esa hora si se tiene que ir la enfermera se queda el medico solo en la unidad de salud y hace lo que puede.

**Sección 2**: Comprender las trayectorias de los pacientes en el primer nivel de atención

- ¿Podría d**escribirme su participación con pacientes con enfermedad crónica**?

- Mi abordaje es, primero velar que tenga bien los signos vital tensión peso y talla. Si tuviera por ejemplo la tensión alta vemos porqué, quizás porque no tomó el medicamente, porque ha sido recientemente diagnosticado, etc. así que lo primero concientizamos para entender su enfermedad. Nosotras como enfermeras tenemos un libro que nos dice qué hacer por ejemplo con un paciente hipertenso darle educación para mejorar sus estilos de vida de comida, ejercicio, les decimos de lo importante que es esa persona para ella misma, para su familia y para la comunidad, le concientizamos para que esté estable con sus medicamentos y estilos de vida saludables. Esos protocolos que seguimos nos los da una organización de enfermería **ANES** (Asociación Nacional de Enfermería) que nos supervisan y son los que nos avalan el carnet, el sello, la cuota anual que tenemos que pagar y si hacemos algo mal nos pueden quitar del trabajo. A nosotras nos paga directamente el ministerio de salud. ANES también nos protege en ciertas circunstancias, son como nuestros abogados, nuestro colegio de enfermería, nos facilitan el **protocolo** que debemos utilizarlo en nuestra práctica clínica.

Cada enfermera tiene 3 especialistas cada 2 meses rotamos y yo me voy a la emergencia por un mes y luego volvemos a cambiar y nos cambiamos también de médicos especialistas que nos corresponden. La enfermera de emergencia se encarga de dejar todo bien preparado esterilizar, comprobar material y recibir a los pacientes urgentes.

• **¿Cuáles son los principales desafíos que enfrenta al cumplir su función?**

Como barrera para mí es no poder darle una atención completa al paciente por falta de tener el tratamiento complete porque así no puede tener su recuperación inmediatamente. Lastimosamente aquí se tiene la cultura de “si no me lo dan no me lo tomo” por más educación que se dé de explicar que es necesario tomar los medicamentos sino los damos no se lo toman, unos porque no quieren y otros porque no pueden pagarlo.

Otro inconveniente sería que a veces no contamos con ambulancias o no están en las condiciones adecuadas para poder trasladar los pacientes.

La encargada de farmacia dispensa los medicamentos, a veces ocurre porque las cantidades han aumentado al triple, las poblaciones aumentan, más aquí porque al ser una Microred la gente se cree que es in hospital, las personas acá le llaman “el hospitalito”. Además, viene mucha gente de Honduras porque acá la asistencia es gratuita y a nadie se le dice que no y se le da al que viene y al momento en que lo necesita.

• **¿Qué estrategias usa para responder a estos desafíos?**

Cuando no contamos con los medicamentos la directora gestiona al ser informada por la farmacia y hace gestiones para intentar dar la medicación. Y como enfermera me la rebusco también hay gente que da los medicamentos de personas que han fallecido por ejemplo y también con gente que sabemos que tienen dinero y nos ayudan. Por ejemplo, necesitamos parches para curas de diabéticos y sabemos que hay gente que tiene familiar en Estados Unidos y les decimos amablemente si nos pueden ayudar a comprarlos, entonces nosotras como personas también nos rebuscamos para poder ayudar a los pacientes. Es una cuestión actitudinal de cada enfermera.

Si es un paciente que cuesta concientizar intentamos contactar con los familiares para explicarles, porque hay veces que son pacientes muy madres y necesitamos del apoyo de la familia, lo hacemos a través del promotor de salud que le hace la visita domiciliaria dependiendo de la zona que le toque. Le decimos al promotor que visite a la familia y les concientice para un mejor seguimiento del paciente. Nosotras como enfermeras de especializada no salimos a domicilios, lo hacen las enfermeras de los ECOS urbanos y coordinamos con la zona de la que sea el paciente, nosotras informamos y ellas se trasladan a las casas (ECOs urbanos y ECOs rurales de los cantones). Ellos hacen las visitas directas, con que cuentan o no cuentan, si necesita referencia al hospital se busca estrategia de buscarle transporte al paciente para que vaya con coordinación de promotor y alcaldía, son personas de escasos recursos, todo eso se hace por in paciente, estrategias se buscan hasta agotar.

**Sección 3:** Trayectorias de cuidado de personas con enfermedad crónica

• ¿Podría describirme los pasos que sigue un paciente crónico desde que entra en la unidad de salud?

Primero el vigilante que le da su numerito, se queda en sala de espera para que enfermería le reciba su tarjeta y le busca su expediente (que en paciente con consulta agendada los dejamos sacados el día anterior), cuando los tengo llamo a los tres primeros, los mido los tallo, les tomo la tensión y pasan a cada médico especialista asignado. EL medico está 15-20 mis con cada paciente, mientras están con el medico yo voy pasando otros 3, los preparo y ya los llevo a la sala de espera de cada especialista que hay otras banquitas con tele. Al salir de donde el medico pasan a farmacia si les dejaron algo de medicamentos sino pasan directamente a archive, después de archive se les da su siguiente cita y dependiendo de cuando le vuelva a ver el medico así le dan cita en laboratorio, después de todo esto ya se van a casa. En archivo la secretaria genera su cuadro y le da la siguiente cita por el Sistema informatizado.

• ¿Podría describirme los pasos que usted sigue para llegar al paciente crónico?

AL ser enfermera de especialista estoy en la unidad de salud y salgo a buscarles a la sala de espera, pero hay veces que por ejemplo tengo una cura y veo que no sana y le puedo dar directamente para examines porque sospecho que es diabético, en ese caso yo detecto directamente, o también con la tensión alta que no está diagnosticado que quías viene por ejemplo a odontología pero yo detecto que la primera toma es alta y le cito para volver a tomársela, así hemos diagnosticado muchos pacientes, luego le pasamos al médico que le pide examines y se confirma el diagnostico. Medimos la presión arterial a todos los pacientes que llegan a la unidad de salud, les tomamos tema. Peso talla y presión arterial y pulsímetro si veo que viene muy fatigado que cambien indica frecuencia cardiaca y presión. Ahí también detectamos incluso emergencias de una persona con una presión muy alta, por ejemplo.

• ¿Cuéntame acerca de tu experiencia brindando apoyo para pacientes crónicos? Por favor, denme un ejemplo reciente y cómo se realizó el seguimiento del paciente, ¿cuáles fueron los problemas concretos abordados?

Ahorita estamos trabajando con **Ejercicio es medicina** que si llevamos la dieta, ejercicio y los medicamentos podemos vivir una Buena vida. Todos nos ponemos excusas, pero les concientizamos que tan solo caminar alrededor de la casa ya puede mejorar. Si por ejemplo son pacientes que no pueden correr les explicamos que al menos caminar es muy Bueno.

Ahora estamos recién empezando vamos a hacer in primer grupo para el 18 de junio les estamos dando papelitos para ver si se acercan, al igual que tenemos grupo de adultos mayor, embarazadas, adolescents, tenemos de todo. También estamos haciendo el grupo de enfermedades crónicas donde explicaremos sobre cada enfermedad, porque es importante tomar los medicamentos y llevar una vida saludable, va a empezar en in par de días.

EL programa ejercicio es medicina primero nos formaron como personal de salud nos evaluaron nos evaluaron nuestra condición física ,nos pesaron, nos pusieron metas a los que tenían que bajar, con nuestros examines y nos pusieron unos ejercicios a realizar y la semana siguiente vimos que la verdad nos funcionó y vimos como la capacidad de nosotros fue mejorando, fuimos el 25% del grupo de la unidad de salud, acudimos los que pudimos en ese momento pero la idea es formarnos todos. Lo organizaba andes (Instituto nacional del Deporte) con la educadora social y forman también a el medico familiar. Se pretende que nosotros como persona l de salud sepamos para poder implementar mejor y mejorar la salud del paciente con orientación. A veces les mandamos directamente al nutricionista para que mejoren la dieta.

A veces para concientizar buscamos alguna consulta que esté libre en ese momento para asegurar la privacidad del paciente.

• ¿Qué tan factible es brindar un mejor soporte a los pacientes que lo necesitan?

Gracias a la coordinación que tenemos como equipo puedo encontrar por ejemplo una consulta especifica que esté libre o nos programamos por ejemplo los días que los especialistas salen a las comunidades. Nosotras organizamos nuestra propia agenda para ver por ejemplo la evolución de in paciente o dar concientización. Si por ejemplo tiene un paciente que tiene que verle el especialista, pero no tiene cupo le busco el espacio y el tiempo para que le pueda ver. La nueva reforma está tratando que todos los pacientes tengan la mejor atención en el menor tiempo posible, por ejemplo, una persona con una enfermedad crónica que requiere ser visto por el medico también por el psicólogo y fisioterapia cuadramos la agenda para que le vean todos los especialistas el mismo día, especialmente los que vienen de lejos.

• ¿Puede pensar en algún problema que presentan más frecuentemente los pacientes que realizan su seguimiento con usted? ¿Notó alguna diferencia entre estos y sus otros pacientes?

Primero que no haya el tratamiento para dárselo. Otro que el paciente no colabore, o que le paciente no venga a consulta porque venga de lejos, o que se le olvide los medicamentos o la propia enfermedad que tengan, esos pacientes después se acercan o les mandamos a decir que vengan. Los pacientes que no vienen o se le olvida el medicamento es más que todo por falta de cultura, la educación de nosotros, la cultura no la queremos cambiar, nuestra negatividad no la queremos cambiar. Pero como decimos esos pacientes son contados, la mayoría sí que cumple, sería solo como un 10% que no cumple, la mayoría vienen con el doctor especialista bien a su hora, con su nevera para la medicación, bien cumplidores.

• ¿Ha notado alguna diferencia en la forma en que los pacientes y el personal interactúan entre sí desde que comenzó este abordaje con la reforma?

El abordaje ha mejorado mucho no solo porque ha aumentado mucho el personal sanitario, sino además que tengan la atención más cerca, antes tenían que ir a Nueva o a Chalate, el hecho de que se le haya acercado la atención ha mejorado mucho el control de las enfermedades, están tomando conciencia de que ciertas enfermedades si no las tratan va a empeorar o puede perder un miembro, así que sí, ha mejorado mucho el abordaje.

**Sección 4**: Estrategias en el manejo de pacientes crónico

• ¿Qué tipo de problemas o desafíos surgen en el manejo del paciente crónico? ¿Cómo manejas estas situaciones?

Visitar al paciente, mandarlo a llamar y llevarlo de cerca con el promotor de salud. Hay muchos pacientes que nos tienen mucha confianza, yo por ejemplo tengo dos teléfonos, uno específico para los pacientes para poder darle por ejemplo una cita por teléfono o para ayudarles con lo que sea, por ejemplo, cuando pierden la cita les aviso por WhatsApp y les ofrezco alternativas como pasarle más rápido para que no se desesperen en la sala de espera esperando, le explico que le darán la medicación etc. Esta estrategia de dar el numero a los pacientes lo hacemos bastantes compañeras, la gente nos llama no pide cita no piden que pueden hacer si tienen por ejemplo la presión alta, etc.…también lo contesto fuera de mi horario laboral y les correspondo la llamada. (Enseña el móvil con muchos WhatsApp de distintos pacientes de distintas edades). Son estrategias que nosotras buscamos ahora que la tecnología esta mejor, por ejemplo, a pacientes que viven lejos les confirmo si llegó su medicación para que sino no tenga que bajar desde lejos. Y la verdad es que la gente lo agradece mucho, a veces vienen a abrazarnos...y esa es la satisfacción que nosotros llevamos, aquí a veces no tenemos horario de salida, aunque supuestamente sea a las 3pm, pero si a esa hora sale algo nos quedamos tranquilamente, sabemos que es nuestro trabajo y estamos adaptadas.

• ¿Cuáles son algunas estrategias que ha utilizado o que ha visto que han usado otras personas, para garantizar que el paciente demuestre una comprensión clara de la importancia del cuidado de su enfermedad?

Se les busca la manera más sencilla de explicarles, a veces se les explica dos o tres veces y les volvemos a preguntar para asegurarnos que entendió, o les damos un papelito para que se lo dé a la familia y lo lean con todas las explicaciones. Yo la verdad es que no me arrepiento de la carrera que elegí, ayudar a alguien que realmente lo necesite, aunque a veces halla mucho papeleo, es bien bonito, no me arrepiento de mi profesión.

• ¿Puede proporcionar algunos ejemplos de casos en los que sintió que un paciente era más receptivo a la información que le estaba proporcionando? ¿Menos receptivo? ¿Por qué crees que es esto?

Yo creo que eso depende d ellos caracteres de cada uno, a veces ha balas con alguien y hace como que no te está escuchando, no le gusta que nadie le diga lo que tiene que hacer, y eso no va por nivel educativo sino por la personalidad de cada uno, que no se abren a recibir información. También es por problemas psicológicos, anda tal vez con muchos problemas de familia de pareja de delincuencia, todo eso es una cadena, de religión a veces algunas religiones dicen que no tome eso u otro. Hay religión que incluso impiden que se pongan vacunas a niños, planificación familiar, transfusiones de sangre así que influye, yo creo que la gente lo toma como in pretexto para no hacer cambios de vida.

• ¿Qué recomiendan como formas de comunicarse con persona con enfermedad crónica para ayudar a mejorar el manejo de su enfermedad?

Hacemos casos excepcionales, por ejemplo, tuve un paciente diabético con muchas complicaciones que estuve lleno a su casa a atender y darle apoyo psicológico y decirle que yo lo iba a ayudar que confiara, una atención personalizada... a veces la gente necesita que más que una enfermera sea una amiga para que esa persona pueda aceptar sus tratamientos.

• Ahora nos gustaría preguntarte sobre tus apoyos como personal sanitario cuando te encuentras con algún desafío.

(Risas) mi familia creo yo, cuando les digo que voy a llegar tarde, cuando tengo que salir in fin de semana para curar a in paciente por ejemplo la diabetes que le comentaba. Y la clínica también porque me facilita las gasas y material de cura, yo informe y me lo dejan llevar. Así que de apoyos son mi familia y la clínica. A veces también hay personas que ayudan al contarle de otras personas que no tiene recursos y apoyan económicamente. No colaboro en otros sitios en la comunidad. El medico también me apoya mucho por ejemplo si estoy en casa de in paciente que me han avisado por la noche y me traslado para allá, yo llamo al médico le explico y me dice lo que puedo hacer. A veces he salido a la noche a la unidad de salud para recoger material e ir a atender al paciente con los insumos que me facilita la clínica. A veces también algún vecino que me ha facilitado el transporte para ir a las casas de los pacientes. Dentro de la familia, mi esposo cuando está porque también trabaja fuera, a veces se viene conmigo a la noche e incluso con mi hijo que dice que quiere ser médico, tiene 10 años y me ayuda a echar la agüita cuando hago curas a pacientes. Hay mucha gente que viene y te dice que está muy agradecida, le visita a la casa y le dan su pastelito para agradecer, una flor, cosas muy sencillas pero que motivan mucho, uno no le puede decir que no porque ellos lo hacen de corazón. Aunque nosotros no podemos recibirles cosas, no nos dejan porque dice que puede ser por interés y que le va a tratar distinto, eso está puesto desde hace mucho, pero es que a veces de pacientes terminan haciéndose amigos.

**Sección 5**: Experiencias con componentes de intervención en torno a la estrategia de fortalecimiento de la capacidad instalada de las Redes Integrales e Integradas de Salud (RIIS), para mejorar su respuesta, acceso y calidad de la atención a las personas, familias y comunidad, reduciendo así́ las complicaciones y mortalidad de forma prematura causadas por las ENT.

• ¿Nos puede describir si existe algún cambio en los últimos 10 años en cuanto al manejo del paciente crónico?

Todo ha ido cambiando, ahora al paciente crónico se hace in mayor chequeo y de examines. Por ejemplo, también de medicamentos algunos que antes solo se daban en el hospital y ahora ya los damos aquí. También ha mejorado la frecuencia de consultas al médico dependiendo de las necesidades del paciente. EN los examines se ha mejorado el perfil de examines (colesterol todo), su ECG dos veces al año, es visto por el especialista, y antes no contábamos con eso. También contamos con el hemoglutest que se toma en el momento, y si viene que se encuentra mal se puede hacer el examen en el momento. El tiempo de espera de in paciente se ha ido reduciendo según las necesidades del paciente, todo eso ha mejorado menormente, si tiene in problema cardiaca antes esperaban meses ahora se puede detector en el momento, se puede hacer creatinina, etc. Los cambios han sido enormes, contamos con más equipos de personal, laboratorio, electrocardiogramas, todo...

Es lo que ha dicho el ministerio que demos una atención integral que se le pueda dar apoyo psicológico si lo necesita, fisioterapia, etc. darle todas las atenciones posibles en el menor tiempo posible, darle una atención complete no solo dedicarse a su tensión, eso es una atención integral.

• Cuénteme su experiencia en cómo se han fortalecido, si considera que así ha sido, sus competencias para la promoción de la salud y el abordaje integral de las ENT

Si porque por ejemplo ahora hay muchos jóvenes que presentan alguna enfermedad crónica y ahora se le toma la tensión y se le ve en la consulta específica del adolescente se le ve dos veces al año se ve el peso, la tensión, se van valorando muchos mecanismos para poder detectarlo tempranamente. Hoy una persona con muchas infecciones de orina o que orina mucho se le pide examines y se puede detector cosas. Hay controles del niño sano, del adolescente en el control del embarazo por ejemplo ahí se detectan muchas cosas, también en pacientes con antecedentes familiar se piden examines a temprana edad buscando esos patrones familiares.

• Cuénteme su experiencia sobre los mecanismos que han definido para fortalecer la disponibilidad de medicamentos para el tratamiento de las ENT.

ANES no se encarga de eso, se dedica a dar los protocolos para que atienda bien con calidad y calidez con la mejor ética posible como enfermera, como in padre para que sea lo más ejemplar posible. Cuando hay problemas de medicación se comenta a la directora para que gestione con otras unidades de salud con hospitales de in director a otro y así va escalando, gestionándose, haciéndose transferencias por ejemplo entre unidades de salud, se informa de si se tiene medicación de más y se pasa a otro si se sabe que va a sobrar en almacén, lo controlan cada 3 meses.

• Me puede describir las acciones de prevención, atención, rehabilitación de las discapacidades relacionadas con las ENT y cuidados paliativos que se están desarrollando desde su unidad.

Les hacemos conscientes de que no tienen cura, pero si con una vida saludable y con la medicación al día podemos vivir mejor que un paciente sin enfermedad, que este tipo de enfermedades no son para que nos deprimamos porque podemos vivir incluso más que personas que no las tienen, eso le hacemos conciencia al paciente, solo es saber llevar la enfermedad aceptarla y saber cómo llevarla, les hacemos conciencia para poder llevar esa carga.

• ¿Qué te pareció más desafiante a la hora de implementar los documentos regulatorios para la atención de personas con ENT priorizadas? ¿Menos?

La cantidad de gente que viene cada vez es más y el personal no es suficiente, si hubiera más personal seria más fácil el trato en nuestras instituciones, de todo tipo de personal sanitario., de farmacia, de enfermería de médicos, de todos...Con respecto al papeleo lo hacemos incluso en casa todas las cosas que piden. Yo todo lo que hago lo tengo que dejar plasmado en un libro, yo doy la concientización hablando rápido, pero lo tengo que apuntar todo en el expediente y eso genera más trabajo rellenamos hasta 3 hojas, el medico tiene que rellenar cuantas recetas...y eso conlleva mucho tiempo que a veces no tenemos, así que las computadoras en cada consulta podrían facilitar bastante. En vez de estar sacando cada expediente podría ser un programa como el que está utilizando el seguro para que no tenga que esperar tanto en archive para que salga su expediente que tenemos solo 3 secretarias. De tantos pacientes que yo le hablé solo tenemos una encargada de farmacia no tenemos a alguien especifico y ahora es la señora de ordenanza que llevamos pidiendo de años que nos pongan una encargada de farmacia. SI archivo se detiene, enfermería se detiene si farmacia se detiene, si el paciente sale disgustado no vuelve... todo es una cadena, y todo debe ir en común para que la gente salga satisfecha y vuelva, todo es en común, como personal de salud nos interesa una población saludable pero para eso queremos que salga satisfecha y que confié porque si no es más fácil sacar unos dólares para unas pastillas que venir a perder toda una mañana aquí.

## Staff interview 11: Pharmacist

Entrevista con: Farmaceutica

Fecha: 13/06/18
Lugar de la entrevista: Unidad Comunitaria de Salud Familiar La Palma
Código del informante: SFML009
Genero del informante: F
Nombre del entrevistador: ML

Farmacéutica citala. Unidad intermedia

**Sección 1**: Perfil del participante y función en la prestación del servicio del primer nivel de atención

Perfil breve del participante (antecedentes, capacitación, historial de participación en la unidad de salud o ECOS)

Mujer de 29 años

**E: ¿Cuál su función aquí en la unidad de salud? ¿Y su relación con el paciente?**

f: Mi función soy farmacéutica y estoy encargada de los medicamentos y los insumos, pues la atención con el paciente es bien cercana con respecto al medicamento, es hasta cierto punto íntimo. Eso, el despacho, explicarle como hacer a una hora, incluso a veces hasta escribírselo, eso y ayudarle, a veces andan con familiares y entonces tengo que explicarles con los familiares y al paciente, porque a veces a los crónicos se les olvida un poco, y es medicamento que es delicado hasta cierto punto.

**E: ¿Cuánto tiempo lleva en esta unidad trabajando?**

f: 14 años

**E: ¿Tu residencia es por aquí cerca?**

f: aquí mismo, mi contacto con la comunidad es bien cercana.

**E: ¿Cómo es tu día a día aquí en la unidad de salud?**

f: Las actividades que realizamos a diario por ejemplo es la tabulada de los medicamentos. Es decir que cada uno de los medicamentos se despacha diariamente, esa es un, ingresar en cada departamento, ver archivos, odontología, entonces es despachar a cada uno, y cada uno tiene un carde o tarjeta de inventario, porque todo se mete manual, todavía no se computa nada, entonces hay tarjetitas de cada uno de los medicamentos, de cada uno de los insumos y cada uno de los medicamentos entonces cuando yo despacho alguna cosa, tengo que ingresarlo, y cuando me despacho yo: porque aquí farmacia y almacén están totalmente divididas, tengo que tener allí cantidad y aquí cantidad, entonces las cantidades tienen que estar rellena en las tablas, entonces cuando yo hago egreso de allí para acá, tengo que hacer el ingreso acá. Quiero ver que más, el ingreso de los pacientes, limpieza, orden.

**E: ¿Y usted tiene que hacer los pedidos de lo que le falta?**

f: No, viene de la central. Nosotros tenemos un tabulador diario, y ahí están todos los medicamentos, y luego al final hacemos el consolidado, eso lo pasamos a otro formato, y aquí se van metiendo todos los datos de los medicamentos, y aquí en este formato esta la parte de farmacia y la parte de almacén, aquí esta lo que yo consumí en el almacén y lo que me quedo, también en el almacén o que me llego de la central, lo que transferí, porque tengo a cargo un ECOS, y aquí es lo que me queda. Esto lo mandamos mensualmente a la región, ellos lo editan y allí es donde ellos están monitoreando las cantidades que tenemos, ya sea en almacén, ya sea en farmacia y según eso ellos nos abastecen los medicamentos. Este proceso lo hago todos los meses.

**E: ¿Tiene algún mecanismo de que si la falta algún medicamento, o se sabe que se va a necesitar más para poder hacer el pedido?**
f: si, en caso de emergencia, pero si manejamos un consumo promedio mensual, que son digamos, la suma de mayo, abril y marzo, los tres consolidados, del consumo por el 1,5 entre 3, entonces ese es mi consumo promedio mensual, eso lo manejamos nosotros y lo maneja el ECOS también, pero como nosotros venimos ya entregando esto mensualmente, entonces ellos nos van monitoreando. Mire por ejemplo con el suero que ahorita tenemos la época de diarreas, ellos me van monitoreando que cantidades tengo de sales de rehidratación oral, y según eso, y que ellos ven que yo tengo poquito y según el promedio mensual, ellos me abastecen, entonces o ellos me monitorean o yo les hago la solicitud.

**Sección 2**: Comprender las trayectorias de los pacientes en el primer nivel de atención

**E: ¿Cuáles son los principales desafíos que enfrenta al cumplir su función?**

f: la educación del paciente, porque a veces estamos limitados, verdad, no saben leer, no saben escribir, entonces cuando yo les digo se van a tomar una pastilla cada 12 horas y ellos me dicen cada dos, y digo no cada doce, entonces me tengo que tomar dos juntas, y entonces siento que eso es un desafío para mí el que el paciente me entienda lo que yo le digo, día por día.

**E: ¿Qué has ido haciendo para superar esas barreras?**

f: Pues verdaderamente para superar es paciencia, que cuando me salen que no lo entienden, pues para mí para explicarlo a veces tengo que bajarme al nivel de la persona, o hablarles con las mismas palabras de que ellos me entiendan, no palabras técnicas de que no me entiendan, utilizar las palabras que ellos utilizan para poder expresarme mejor o bueno son tantas cosas, pero eso si es difícil a veces, y tener paciencia porque a veces, no entienden, porque a veces he tenido pacientes que son diabéticos y que se toman 4 glibenclamidas por ejemplo, y me vino una señora de que se tomaba las cuatro de un solo juntas, entonces, es difícil. A veces uno se siente mal porque dice en que falle, o, para mi forma de ver, yo les explico bien a los pacientes, pero a veces se ve difícil la forma de percepción de ellos.

**Sección 3:** Trayectorias de cuidado de personas con enfermedad crónica

**E: ¿Podría describirme los pasos que sigue un paciente crónico desde que entra en la unidad de salud hasta que llegan a ti?**

f: Bueno entonces, mire cuando lleguen en crisis, ya sea diabético o hipertenso, se pasan directos a emergencia, lo tienen en el evaluación, el medico lo está evaluando, y dependiendo de cómo vea eso, o lo evalúa o lo pasa directo a la consulta, para darle sus medicamentos, o digamos que si ya trae el azúcar súper alta, ya creo que lo va a tener en seguimiento, entonces dependiendo de cómo evoluciona o lo refiere o le deja el medicamento o igual se lo cambia para la insulina, pero solo los crónicos. Un crónico que viene por su dispensarización de medicamento, viene a archivo, se anotan, después la enfermera los llama a pesarlos, a tomarlos su presión arterial y su temperatura, y en lo que ella está tomando sus signos vitales, archivo está sacando su expediente, después, la enfermera los llama para poner todos los datos y así los ordena con el médico. Después de pasar su consulta con el médico, pasan al mío, al despacho del medicamento.

**E: ¿Cada paciente entonces pasa por farmacia si o sí?**

f: si

**E: ¿Entonces aquí como unidad intermedia, que servicios existen?**

f: aquí hay, médico general, enfermería, promotores de salud, también está el odontólogo, está el inspector de saneamiento.

**E: ¿Qué pasos lleva usted para acercarse al paciente crónico?**

f: El paciente llega aquí. A veces si acompaño al odontólogo a escuela saludable. Antes se hacían brigadas, y hoy ya no se hacen, pero a veces cuando van a escuelas a dar controles o escuelas saludables, si yo los acompaño, a la dispensarización de los medicamentos. De lo contrario, aquí en la unidad, casi siempre ellos vienen hacia mí, yo permanezco aquí anclada.

**E: Alguna otra causa por la que usted sale a la comunidad.**

f: mí, no.

**E: ¿Qué otro problema puede presentar el paciente crónico, por ejemplo, a la hora de tomar su medicación?**

f: A veces no escuchan, a veces, es eso, como los mandan solos, a veces no vienen acompañados, a veces eso si se hace engañoso, que vienen solitos, más que todo las personas de tercera edad, vienen solas, y a veces no me escuchan, a veces si yo salgo a despacharlos fuera, cuando son ancianitos y andan con su bastoncito, yo les digo pásame la receta, y entonces yo me voy a la farmacia, agarro los medicamentos, los meto en una bolsita y de ahí yo vuelvo con ellos y me voy a sentar y les explico, a veces así me toca, y no me pesa la verdad, eso quizás.

**E: ¿Con respecto al abastecimiento ve usted algún problema con respeto al paciente crónico?**

f: a veces sí, pero así es importante nuestros informes, así se hacen compras.

**E: ¿Me puede dar un ejemplo de algún problema que haya encontrado el paciente?**

f: Entra un paciente a coger el suero, esta borracha, este borracho pues (haciendo alusión a un paciente que entra), así que así es difícil. Uno esta vulnerable hasta cierto punto, porque por ejemplo, ayer vino una señora crónica, ahorita hemos entrado en un lineamiento, antes era la receta repetitiva, ósea le daban una receta para tres meses, y se le daba la medicación y se quedaba el registro, ahora ya no se puede, ha entrado un lineamiento que hay que hacer un montón de papeleo, al mes va a venir el paciente a retirar su medicamento, y va a tener que pasar por chequeos de su presión, tiene que pasar consulta y luego recoger su medicación, entonces tiene que ser un paciente estable, hasta cierto punto también de físico, y bueno vino una señora, y el doctor le había dejado la receta repetitiva y el doctor nos la envió, entonces yo me quede viendo la receta que era del mes de mayo y ella no había pasado en mayo y se la habían dejado para junio, y yo no la vi en consulta, entonces fui donde el doctor y me dijo así, se me olvido, y fui donde la señora y la señora todo molesta, porque la faltaban unos días para retirar, y la señora bien molesta y me dijo un par de cosas, y yo callada, entonces ahí sí, en eso es bien difícil, la intolerancia y la paciencia, si porque nosotros no le faltamos el respeto a nadie, nos merecemos entonces respeto nosotros también, todos nos merecemos respeto, pero es difícil en este entorno, no se tiene la educación suficiente, y yo tampoco me voy a poner a explicar le tanto al milímetro porque no me van a entender solo van a pensar que no se la quise dar. Hay gente que sí, que te entiende, pero hay gente que piensa raro, entonces es como venga el lunes, yo si le voy a hacer el favor de despacharle el medicamento, pero ahorita no puedo entonces la señora se fue así como que yo no quería. Porque, la verdad que a nosotros nos caen mucha revisión de cuentas, mucha supervisión, y si me ven la receta de tantos días y ven que yo la despache, eso, por un lado, y por el otro que, si ya hay receta repetitiva pues entonces a veces, uno esta vulnerable a muchas cosas.

**E: ¿Por ejemplo tu a los promotores de salud le das los medicamentos para que cuando visiten estas comunidades más alejadas se las den o como haces?**

f: No, no no, teníamos ese proyecto con el doctor que estaba, de mandar los medicamentos con los promotores, pero como con lo de la receta repetitiva ya lo quitaron, no porque sí, eso podría ser una opción.

**E: ¿Usted cree que sería una buena estrategia?**

f: Fíjese que eso sería una opción. El seguro tiene esa opción el mandarlos los medicamentos, a mí por ejemplo me hacen muchos beneficios, porque yo estoy asegurada, porque yo por ejemplo no voy a traer el medicamento al seguro yo no voy a pedir permiso solo para traer el medicamento, no lo hacía pues, entonces nos ayudan un montón, se van a arreglar las cosas allá, se hacen una recetas y ellos por correo nos mandan los medicamentos a mi o a otras personas, entonces ya nos ahorramos tiempo y dinero, entonces yo siento que aquí sería bueno también, verdad porque hay personas de tercera edad que les cuesta venir aquí, que incluso de la ciudad que estamos cerca, pero aun así tenemos a personas que les cuesta venir. Eso sería bueno. Los promotores podían hacer eso, eso no sería problema, además vendría todo especificado, pero lamentablemente eso no se puede hacer.

**E: ¿Recuerdas alguna situación de un problema difícil? ¿qué paso? ¿cómo lo resolviste?**

f: bueno el que más recuerdo, yo no tuve tampoco muchos problemas, pero fue que yo estaba recibiendo medicamentos, estaba despachando medicamentos, y estaba atendiendo a la consulta a otro, entonces el señor quería que le despachase inmediatamente sus medicamentos, entonces se puso a decirme un montón de cosas, y yo solo me puse a decirle, mire permíteme que yo no solo despacho medicamentos, hago un montón de cosas, entonces yo me fui y me fui a llamar al director, y él fue a hablar con él, y yo seguí haciendo mis cosas, y luego yo atendí al señor y el doctor lo tranquilizo bastante, pero si estaba nerviosa. Y la verdad que no solo despacho medicamentos, hago un montón de cosas, mire yo a veces en la bodega estoy desde que comienzo hasta que termino, entonces es difícil porque cuando hago eso me atraso con otras cosas, otra vez tengo pendiente pedidos o algo, tengo que egresarlos, para que este cabal, por si allí dice que tengo 100 tiene que estar 100 allá, inventarios, inventarios hay que hacer casi a diario. Y al final del mes cuando estoy haciendo esos consolidados, como aquí esta, y dice que tengo tantas y quiero ver y reviso que está bien. Y hay veces que surgen cosas por ejemplo una emergencia, viene el doctor deme tal, ya le doy luego la receta, entonces yo a veces anoto o me lo gravo, entonces cuando hago inventario y falta, entonces voy y le pido una receta, porque así debe de ser.

**E: ¿Ha notado alguna diferencia en la forma en que los pacientes y el personal interactúan entre sí desde que comenzó este abordaje con la reforma?**

f: Si fíjese estamos más abastecidos, y con respecto a crónicos estamos bastante bien, este porque, también con la entrada de los especialistas la población creo que tienen una mejor consulta pues. Porque a veces van a los especialistas de los niños, o los crónicos, psicólogo, ginecólogo entonces pienso que si si se ha mejorado en este aspecto.

**E: ¿en qué crees que no se ha mejorado, que se deberían poner más esfuerzos?**

f: en recursos, porque en cuestión humana, yo siento que es mucho, mucha presión porque como hay tantas cosas que piden por hacer, que a veces un recurso no alcanza a hacer todo, por ejemplo, a promotores son tantas cosas que los saturan, entonces por hacer tanto, no hacen bien ninguna, porque son un montón de cosas. Por eso también pensamos en la receta repetitiva porque tiene que hacer tantas cosas y cargarlas con eso. Yo no tenía problema como decía el doctor, yo si hay varios pacientes del día yo me voy a despacharlos, no voy a dejarlos irse a su casa, yo tengo ahí la ambulancia o el pick-up, yo no tengo problemas, pero pues sí, ahora lamentablemente no se puede hacer más. Solo podemos que evaluar, evaluar a aquellas personas que han puesto para hacer tal programa y priorizar, porque no se está acercando salud a la gente.

**Sección 4: Estrategias en el manejo de pacientes crónico**

**E: Que estrategias usted ve que tenga para superar algunas barreras que comenta, otra serie de problema que puedan tener para abordar su enfermedad**

f: Fíjese que tienen sus clubs, hay de tercera edad, de embarazada, ahí también está el de crónicos, y ahí a veces yo me incorporo, para darles charlas, hay veces siempre hay dudas, eso sería bueno, pero ahí tendría que entrar ya la alfabetización, verdad, de ahí ya como que nos hace un poquito difícil, pero si sería bueno.

**E: ¿Y en cuanto a lo que me decías del lenguaje para que los pacientes que no tienen un alto grado de alfabetización en salud, que otros problemas puedes ver entorno a la comunicación que un paciente crónico pueda tener?**

f: A veces la distancia porque tenemos los cantones verdad, que a veces están bien retirados, a veces las personas no vienen por su medicamento por la distancia, o porque su hijo no la puede traer.

**E: ¿Puede proporcionar algunos ejemplos de casos en los que sintió que un paciente era más receptivo a la información que le estaba proporcionando?**

f: cuando le hablo con palabras que me entienda, que no hablo con palabras técnicas, sino que le hablo, que me bajo al nivel de cada paciente y así me adapto.

**E: Ahora nos gustaría preguntarte sobre tus apoyos como personal sanitario cuando te encuentras con algún desafío [apoyo personal, apoyos profesionales y apoyos en la comunidad].**

f: me ayuda nada más en el despacho de medicamentos, en el despacho con la papelería.

**E: ¿Y en cuanto a lo profesional, como es el equipo?**

f: Si, es un apoyo, nos ayudamos mucho, con respecto a papelería es porque quizás es un poco delicado, porque como son números, es un numerito lo que hace la diferencia, pero por lo demás todo bien. La verdad que somos un equipo y nos tenemos que ayudar.

**E: ¿Y qué tipo de capacitación recibes para reciclarte en la profesión?**

f: Pues de parte del ministerio la verdad que no mucho, es poco la verdad, la capacitación es poco, cuando nos dan es como una retroalimentación, no es algo nuevo. En todo en caso en otros recursos, pero aquí en farmacia es casi lo mismo, no hay nuevos lineamientos, y cuando los sacan nos lo dicen, pues como una vez al año o así.

**E: ¿Y usted tiene contacto con personas de su gremio?**

f: si claro, tenemos reuniones mensuales con la encargada de las pilas, y de aquí del ECO del Carmen, al cargo mío, puede decirse. Entonces nos llamamos mes a mes como estamos con cuestión de consumo promedio mensual, medicamentos próximos a vencer, desabastecimiento otras cosas, entonces estamos las tres porque solo las tres nos tenemos, y hacemos red, con San Ignacio nos reunimos porque, toda la Microred como que no le ven mucho provecho, pero nosotras si, si nos hemos mejorado.

**E: ¿Como considera que ha mejorado con la Microred?**

f: Como Microred no nos reunimos, nos reunimos como municipio, porque pertenecen a San Ignacio, las pilas y el Carmen, pero como Microred no nos hemos reunido porque no han querido, porque no lo ven como provecho, es como para ellos perder un día, pero para nosotros no, porque así avanzamos, tenemos que estar para hacer el consumo promedio las tres, el Carmen, las pilas y yo, de que todo esté bien, porque eso si nos evalúen de que todo esté bien, y en cambio la Palma, los ECOS que tiene a cargo al parecer no hacen sus consumos promedios pero a nosotras si nos evalúan, nos hacen los consumos promedios, nos hacen graficas de cómo estamos, y por ejemplo El Carmen no puede tener más que yo, entonces por eso nos reunimos una vez al mes, y por eso yo propuse que nos reuniéramos toda la Microred, pero no apoyaron la verdad, entonces nos reunimos como municipio, pero si serviría para las transferencias, porque sí que ha medicamentos próximos a vencer que tal vez la Palma no lo mueve pero las pilas si lo va a mover en dos meses y ella no lo mueve en un mes y ya después se vence, entonces para eso sí, yo le veo eso

A nosotras no nos convocan en la Microred, yo si iba, estuve yendo porque participaba en un comité de farmacoterapia, entonces a nivel de chalate, de aquí de la Microred, yo era la responsable, y nos reuníamos a nivel de chalate, pero ese comité lo deshicieron o hicieron un nuevo, entonces ya no nos reunimos, y esa reunión si servía porque yo traía información, información de chalate, pero ya no, no nos convocan. Yo creo que el comité ya lo hacen a nivel de región, ya no es a nivel de chalate, entonces yo ya no asisto, pero en ese entonces, yo asistía a la Microred, ahora ya no llaman a nadie para que lleguen. Allí en Chalatenango hay una responsable que nosotras la transmitimos la información y ella se lo transmite a los demás. Hay un grupo de WhatsApp y es quien nos transmite la información.

**E: ¿Os habéis intentado organizar vosotras para hacer alguna reunión a nivel municipal?**

f: no, no nos han dado chance para organizarnos, quizás por tema de distancia porque nos dicen de reunirnos en Santa Tecla, que está lejos.

**E: ¿Estas en contacto con alguna farmacia de la zona?**

f: Si, yo tengo farmacia, entonces tengo mi farmacia, pero igual, yo ayudo al paciente, cuando ellos necesitan algo, quizás porque acá se ha terminado, o porque el doctor le manda otra cosa, pues entonces yo lo hago igual, le apunto los miligramos, como tiene que tomárselo, yo se lo doy, yo no les refiero a ninguna farmacia. También hay veces que los tratamientos no son correctos. Por ejemplo, hace un tiempo vino un policía que le habían dejado metronidazol y no había, pero era para parásitos, y el metronidazol no se ocupa solo para parásitos, entonces es de amplio espectro, entonces le dijeron que no, que el metronidazol no era para eso, y que el metronidazol era 4 de un solo, el caso es que el tratamiento que le dieron no le iba a favorecer en nada, y ya le explique al paciente, entonces ya se fue tranquilo, y a la vez algo molesto pues, entonces ahí depende de la persona, de los conocimientos que tengan.

**E: ¿Cuándo tu detectas algún error en la medicación como lo gestionas?**

f: Yo hablo con el doctor porque necesito que me haga una nueva receta, yo la puedo manchar en un momento dado para arreglarla a veces, pero a veces me la tiene que hacer de nuevo. También me pasa con los médicos privados, porque aquí viene pacientes. Por ejemplo, vino una paciente que le habían dado amoxicilina/ac clavulánico, pero se lo habían mandado en frasco, y el laboratorio que nos abastece a nosotros el frasco con otra cantidad por ml, por lo que lo que no iba a darle suficiente, y la paciente quería un frasco, entonces en esos casos o le ponen en la receta el fabricante o le ponen la cantidad, y ahí yo le explico a la gente que si trae esa cantidad tiene que tomarse tanto. Nosotros no podemos hacer un pedido de medicamentos que no tenemos, porque nosotros solo manejamos lo que nos mandan. Antes sí que se hacía es, nosotros preparábamos las órdenes para los medicamentos que necesitamos, ahora mismo ellos abastecen lo que necesitamos nosotros, si hay un medicamento nuevo ellos nos empiezan a mandar cierta cantidad para que nosotros empecemos a trabajarlo, y cuando ya tenemos un consumo promedio mensual ya nos empiezan a mandar la cantidad. Pero a medicamentos nuevo nosotros no lo manejamos porque no tenemos acceso a las opciones que ellos tienen.

**E: ¿Has realizado alguna capacitación al personal que trabaja aquí para que se haga un buen uso de medicamentos?**

f: Si cuando nos reunimos en la sala situacional, ahí lo hablamos todo, y cuando llega mi turno, sí que les hablo de cómo se han gestionado las recetas, que tienen que escribirle las cantidades, dejar la receta completa, los mg, cuantas tienen que tomar, si son capsulas o que son. Y si no está bien, yo la regreso para que lo corrijan. Estas reuniones son una vez al mes. Por ejemplo, ahora con diarrea hay que hablar que estrategia se sigue.

**Sección 5**: Experiencias con componentes de intervención en torno a la estrategia de fortalecimiento de la capacidad instalada de las Redes Integrales e Integradas de Salud (RIIS), para mejorar su respuesta, acceso y calidad de la atención a las personas, familias y comunidad, reduciendo así́ las complicaciones y mortalidad de forma prematura causadas por las ENT.

**E: ¿Nos puede describir si existe algún cambio en los últimos 10 años en cuanto al manejo del paciente crónico?**

f: La verdad que bastante, en general, se dan charlas en general, pero se ha incrementado la toma de medicamentos.

**E: ¿Y eso porque crees que es?**

f: por los malos hábitos en general, también hay más problemas de presión, sobrepeso.

**E: Cuénteme su experiencia sobre los mecanismos que han definido para fortalecer la disponibilidad de insumos/equipo para el diagnóstico temprano de las ENT.**

f: Aquí no hay laboratorio, pero si se toman los exámenes y si hay tiras, hay tiras rápidas para la prueba de orina y también las enfermeras toman exámenes de sangre y se mandan a la palma. Algunos insumos se llevan a nivel de farmacia y ahorita estamos desabastecidos, a veces también no hay reactivos para ciertos exámenes, depende de la demanda.

**E: Cuénteme su experiencia sobre los mecanismos que han definido para fortalecer la disponibilidad de medicamentos para el tratamiento de las ENT.**

f: Con los hipertensos, es bien difícil, porque ahí se la juegan los médicos, hay veces que en caso de desabastecimiento hay que cambiarlo por otro, y ver que pueda servir al paciente porque la gente a veces es de escasos recursos, y no pueden estar sin medicamentos, porque otros son bien caros.

**E: ¿Y tú les asesoras por otro que se pudiera sustituir?**

f: no, no ahí es solo el médico. Aquí la que más demanda tenemos es el irbersartan y es el que más desabastecido esta.

**E: ¿Cómo resuelves tu cuando hay un problema de desabastecimiento?**

f: Vaya mire, no sé si con esto respondo, pero hay algunos medicamentos que son especializados, que esos medicamentos el internista los evalúa a los pacientes y se los deja, esos medicamentos no me los manda la región directamente a mí, sino que se los mandan a la palma, y la palma me abastece a mí por consumo si hay existencias, y a veces si falla, porque como ellos tiene que abastecernos a nosotros, a todos los ECOS de ella y a todos mis ECOS, necesita más también.

**E: ¿Qué haces cuando falta algún medicamento?**

f: Hay personas que, si lo pueden comprar, que dicen si si yo lo puedo comprar, pero hay personas que no pueden, y no pueden no tomarlos, y claro la región nos llama a nosotros a ver como esta. Y hay gente que lo compra, lo adelanta y luego se le informa de que día hay aquí y se le compensa lo anterior. Pero si hay que informar cuando hay desabastecimiento porque ellos no tienen la culpa.

**E: ¿Y en esos casos que crees tú que se podría hacer para aquellos pacientes que vienen y no hay medicamentos y no pueden comprarlos?**

f: Pues en esos casos lo que se puede hacer es informar y hacer una llamada rápida en un momento dado a la región, para ver cuándo van a mandar, para así tener informada a mi población, porque hay gente de escasos recursos, entonces informarles a todos, al doctor, entonces como le repito, hay que evaluarlos y ver que otra opción hay de medicamentos para poder dar.

Vaya y recomendaciones no sé, porque serian más a nivel de la región, por ejemplo, hay veces que compran insumos, u otros medicamentos que no nos son como prioritarios, gastan mucho en papelería, que podrían ser recursos que si son necesarios.

**E: ¿Has visto que algún paciente se le haya complicado su situación por desabastecimiento?**

f: No por eso que estamos en comunicación, las farmacéuticas de las pilas, la palma, el Carmen, incluso en otras regiones a lo mejor ellos estas super abastecidos, y se pueden hacer transferencias, y como nos tiene bien monitoreados, se hacen transferencias, entonces para quitarle un poquito a uno y darle al otro.

**E: ¿Que recomendaciones darías tu para mejorar el manejo del paciente crónico?**

f: Deberían darle más prioridad en medicamentos, porque hay un alza ahorita de personas crónica, darles prioridad en todo el sentido, en cuestión de medicamentos, en cuestión de ayuda, con respecto a la distancia.

Es importante la organización, hay veces que esta unidad básica esta menos desabastecido que una intermedia y especializado, entonces fuera buena la propuesta de coordinarnos en la región, pero, si no se lo toman a bien esa propuesta pues no se puede hacer, yo lo veo provechoso porque en vez que el medicamento se desplace a otros lugares, tal vez nuestra población la pudiese aprovechar, y nosotras ya lo estamos haciendo. Entonces una reunión entre todas ayudaría. Y lo veo no solo para cuestión del medicamento, sino también cualquier otra duda que le surja al encargado de farmacia, ahí entre todos lo podemos resolver, u otra de nosotras puede saber ya la respuesta, entonces ahí nos podemos fortalecer, o para cualquier sospecha de algo, un efecto adverso que se pueda repetir en otra persona. yo por ejemplo cuando tengo dudas estoy en constante comunicación con otras farmacéuticas, aquí por teléfono, sobre todo de la pilas, citala, el Carmen y la palma, ya mas allá de la Palma son bien cerrados, por eso una reunión mensual de todos los encargados de farmacia siento que estaría bien, pero no todos lo ven de ese punto de vista, porque hay muchas dudas, de porque unos lo hacen de una forma y otros de otra, o quizás yo puede que esté haciendo algo mal, ahí aclaramos dudas.

Bueno muchas gracias. No sé si tiene alguna duda para nosotras.

El informe de devolución se requiere.

## Staff interview 12: Laboratory technician

Entrevista con: Laboratorio

Fecha: 13/06/18
Lugar de la entrevista: Unidad Comunitaria de Salud Familiar Ciudad Romero
Código del informante: SMMJ010
Genero del informante: M
Nombre del entrevistador: MJ

**Sección 1**: Perfil del participante y función en la prestación del servicio del primer nivel de atención

- Perfil breve del participante
- Me gustaría preguntarle sobre su función acá en la unidad de salud y dónde se formó.
- Yo soy el encargado de laboratorio clínico donde se realizan los exámenes a todos los pacientes que acuden a esta unidad. Yo primero estaba de la unidad de Carolina en San Miguel y luego perdí el traslado acá porque soy de por acá cerca. Yo hice mi licenciatura en la UES en laboratorio.
- Muchas gracias, ahora me gustaría preguntarle sobre su función actual en el primer nivel de atención
- Yo llego acá a las 7:30 aunque intento estar en un poquito antes para empezar a tomar las muestras a los pacientes. Normalmente veo como 14-15 pacientes, los médicos en las boletas le ponen el diagnóstico y todo.
- ¿Cuántos de esos pacientes que ves a diarios crees que son enfermos crónicos?
- Pues de los 14-15 que veo diariamente como unos 3-4 serán crónicos, diabéticos, renales o hipertensos.

**Sección 2**: Comprender las trayectorias de los pacientes en el primer nivel de atención

- ¿Cuáles son los principales desafíos que enfrenta al cumplir su función o que cosas mejorarías?

- Pues aquí no podemos hacer la prueba de electrolitos a pacientes renales y eso sería muy importante que la tuviéramos acá y no tener que mandarla al hospital como tenemos que hacer ahorita, yo centrifugo la muestra y sigo la cadena de frío, un motorista viene a recogerla para llevarla al hospital. De ahí esperamos los resultados que suelen tardar como una hora y media, ese mismo día recibimos los resultados, aunque lo más recomendable sería tener aquí el equipo de tanto paciente renal que hay. Otra cosa que nos gustaría tener es un equipo automatizado o semi automatizado de química que deberíamos contar con ella en la especializada con tanto paciente renal que tenemos, yo pensé que tendría, pero la realidad al llegar fue otra, lo he intentado gestionar con mi jefe regional pero no he recibido respuesta por ahora.
- ¿Y en un examen, que no requiera ser llevada la muestra al hospital, sino que se haga acá, cuanto tiempo tarda en recibir los resultados?
- Depende, si el medico los pide urgentes se le dan ese mismo día sino incluso al siguiente día que vienen a pasar la consulta con el médico especialista ya tiene los resultados de los exámenes.
- ¿Y solo la US especializada tiene laboratorio?
- Sí solo la especializada, el resto de unidades de salud intermedias o básicas también se realizan los exámenes acá.
- ¿Y viajan los pacientes hasta acá o solo las muestras?
- No, en el caso de los ECOS, cada día tienen un día asignado para toma de muestra que se la toma la enfermera en la unidad, la trae un polivalente y yo recibo acá la muestra.
- ¿Y cuánto tarda desde que se saca la muestra hasta que la recibe usted acá en la especializada?
- Depende de la distancia, porque por ejemplo la isla de Méndez está a 45 minutos, pero los demás ECOs están más cercanos. Ese mismo día las analizo y luego a los dos días vienen a recoger los resultados. Es una modalidad que yo he adaptado por que antes se entregaban a los 3 días, pero yo lo intento hacer cuanto antes porque a veces puede ser importante y no hay que demorarlo mucho tiempo. El polivalente recoge los resultados y los lleva a las distintas unidades de salud.

**Sección 3:** Trayectorias de cuidado de personas con enfermedad crónica **y Sección 4**: Estrategias en el manejo de pacientes crónico

- ¿Podría describirme los pasos que sigue un paciente crónico desde que entra en la unidad de salud/establecimiento?

- Bueno depende, si es un paciente renal le pregunto cómo ha venido a la unidad (caminando, en bicicleta, etc.) porque eso puede influir en los resultados de los exámenes así que les dejo descansar un ratito antes de tomarle la muestra. Cuando vienen ese día soy la primera persona que ven porque ya vienen directamente a tomarse los exámenes, normalmente tardan como 10-15mins en ser atendidos, de ahí después de tomarle la muestra ya se van porque tienen la cita con el médico especialista a los 2-3 días para asegurarme que ese día tiene los resultados. Así suele ser, el médico especialista le da cita supongamos para dentro de 3 meses y ese mismo día pide la cita conmigo para hacerse los exámenes un poquito antes de la próxima consulta para ir con todos los resultados.

- ¿Podría describirme los pasos que usted sigue para llegar al paciente crónico que por ejemplo esté inmovilizado y no pueda acceder a la unidad de salud?

Pues hasta la fecha no he tenido yo ningún caso así, pero supongo que en esos casos va la enfermera a pincharle y trae la muestra, pero como le digo no he tenido ningún caso de ese tipo.

- ¿Cuánto tiempo llevas trabajando acá?

- Llevo ya dos años.

- ¿Y cómo haces para que los pacientes se enteren bien de los exámenes que se van a hacer y todo?

- Yo les abordo, les explico bien que tienen que hacer, si tienen que venir en ayuno, las muestras de orina les explico todo bien. Ellos me entienden porque no les hablo en técnico sino de una manera que me pueda comprender bien porque a veces cuesta con personas de acá para que lo entiendan todo bien.

- ¿Y las indicaciones se las das en papel también?

- Sí también se lo entrego en papel con todas las indicaciones y dibujos para que lo entiendan bien cómo tomar la muestra de orina y todo eso.

- ¿Y tú te sientes apoyado por el resto de profesionales sanitarios del centro?

- Hasta el momento sí, me llevo bien con los compañeros, cuando necesito algo me apoyo más con las enfermeras, por ejemplo, cuando tengo algún problema con una vena, más que todo con los neonatos porque a veces cuesta, he recibido formación específica para ello, pero a veces cuesta.

- ¿Puede pensar en algún problema que presentan más frecuentemente los pacientes que realizan su seguimiento con usted, por ejemplo, si vienen o no a las citas de laboratorio, y por qué crees que eso es así?

- Con los pacientes del médico especialista ellos son bien cumplidos, no suelen faltar a las citas. Con otros sí, a veces se les olvida y yo les reprogramo, pero no para meses sino para algo cercano para tener el diagnostico cuanto antes. Alguno dice que es porque están trabajando, o tienen problemas en casa o a algunos se les olvida no más.

- ¿Crees que hay alguna otra razón para que no acudan a la cita?

- Un paciente tuve que fue por el transporte me dijo, y que a veces son de escasos recursos uno les dice venga aquí, pero se da cuenta de que son de escasos recursos, en una ocasión por ejemplo me dijo uno que no tenía para el transporte y le dije tome, y ahí le día para el transporte.

- ¿Entonces usted no sale a las casas a buscar pacientes crónicos no?
- No, en ese caso se encargan las enfermeras, más que todo en el caso de pacientes renales.
- ¿Y considera que hay buena coordinación por ejemplo en el transporte de las muestras de los otros Ecos?
- Hasta el momento me han cumplido con lo que yo les pido en regla, no he tenido dificultad hasta el momento.
- ¿Y el transporte de la muestra ha sido siempre así?
- Bueno desde que yo vine sí, tenemos un cronograma tal día tal ECO envío de muestras a la US especializada ya viene en el calendario. Pero antes de que no existieran los Ecos me comentaban que tenían los pacientes que venir todos aquí, pero en ese momento había 3 técnicos de laboratorio, en este momento solo estoy yo.
- ¿Sabes si en otras US especializadas tienen solo un técnico de laboratorio o varía?
- Sí solo suele haber uno, por ejemplo, en Ciudad Barrios solo es mi colega, pero a él le mandan del servicio social, pero a mí no me mandan por eso del peligro social. Miré que con la peligrosidad desde que yo vine no he tenido problemas no he visto nada.
- ¿Crees que ha mejorado el tema del transporte de muestras o ha empeorado?
- ¿En eses aspecto es mejor porque si no yo me tendría que trasladar y quien me cubriría acá? Y antes era la propia gente que tenía que trasladarse porque no existían los ECOs, la reforma es la que vino a cambiar bastante porque no existían los ECOs.
- - ¿Para mejor o para peor?
- Para mejor por supuesto porque antes tenían que venir hasta aquí y por ejemplo desde la Isla de Méndez que está retirado algunos no tendrían para el transporte o el riesgo que podrían sufrir en el transporte.
- ¿Habéis tenido problemas de desabastecimiento de material de laboratorio?
- Hasta el momento está bien abastecido porque yo hago una programación del pedido y lo solicito a la región oriental al CEN, me comunico con mi jefe regional y me dice qué hay y que me puede mandar. No todas las US especializada tienen exactamente lo mismo porque cambian los reactivos que se requieren, hay algunas US intermedias que sí que tienen laboratorio como la que yo estaba antes que fue equipada por un proyecto de Andalucía. EL pedido lo realizo anualmente al jefe regional de laboratorio, él coordina todos los laboratorios de la región oriental que forma parte del Minsal, cada uno va pidiendo en base a las necesidades que tiene, yo voy programando en base a lo necesitado el año pasado, veo cuantos realizo cada mes y en base a eso programo. Yo realizo casi 2000 exámenes mensuales de todo tipo.
- ¿Y una vez al año le mandan todo el material o cómo?
- No lo enfocan por meses, en enero me mandan un pedido a mitad de año me mandan otro según el registro mensual de lo que hayamos necesitado qué nos falta qué no. Cada mes hacemos el control de lo gastado ese mes y si voy a necesitar más lo pido. Todos los colorantes por ejemplo los mando pedir al laboratorio Maxblock a centrales de San Salvador que pertenece al Minsal.
- ¿Los resultados de los exámenes quien se los entrega?
- Yo los entrego a archivo y los meten en su expediente y cuando vienen a consulta ya se los entrega el médico, pero en realidad yo no sé si esos exámenes que les hago vienen a recogerlos o no.
- ¿Y los crónicos cada cuanto se suelen hacer análisis?
- Cada 2 meses o 3 dependiendo de cómo estén. Como también se están realizando tamizajes renales en cooperativas pues depende.
- ¿En el caso de los tamizajes en las cooperativas de agricultores ellos vienen acá o cómo?
- Esos se focalizan más que todo en los ECOS en pacientes hipertensos o diabéticos, pero de cooperativas nunca he tenido porque cuando toman las muestras en terreno me los mandan directamente o si se hace una campaña de tamizaje desde la US se pasa con el altavoz y ellos vienen a tomarse la muestra acá, a mí no me importa si se me pasa del límite de pacientes siempre se los saco, pero es así que se está manejando.
- ¿Y por ejemplo para Papanicolau (citologías para la detección precoz de ca de cérvix) también se analiza aquí o cómo?
- No eso toman las muestras las enfermeras y lo mandan a analizar a nivel central al laboratorio MaxBlock que le comentaba en San Salvador.
- ¿Y siempre ha estado este laboratorio central?
- En lo que yo sé sí, antes de la reforma.
- ¿Sabe cómo era todo esto antes de la reforma?
- Para serle sincero no, como yo me estaba formando todavía en la universidad no sé cómo funcionaba antes.
- ¿Y consideras que hay alguna otra prueba que se debería hacer acá porque consideres que debería ser importante para el seguimiento de un paciente crónico desde el primer nivel de atención?
- Sobre todo, la Hemoglobina glicosilada para los diabéticos que se hacen en el hospital de Jiquilisco o se manda al laboratorio de San Miguel. Además, las pruebas de electrolitos para enfermos renales que pasa lo mismo, se tienen que mandar al hospital. Lo del ácido úrico ya sí que se hace acá desde que se fundó, colesterol triglicéridos y todo se hace aquí. Pero, como le decía, si contáramos con un equipo automatizado se podría hacer HDL, LDL, nitrógeno ureico (BUN), filtrado glomerular, eso nos sería muy útil poder hacerlo acá claro para que no tuvieran que ir hasta el hospital. La de electrolitos es una máquina aparte, la de HB glicosilada no se cual equipo requiere porque no he estado nunca en el laboratorio del hospital. Esa máquina automatizada es bastante cara, supongo que por eso no nos la ponen y no está en ninguna US especializada, solo en el hospital que maneja la semiautomatizada, yo manejo una que es menos que la semiautomatizada.
- ¿Y durante la noche si viene alguien urgente no se pueden hacer analíticas no?
- No en ese caso cómo yo solo estoy por las mañanas tendrían que irse al hospital.
- ¿Cuáles serían entonces sus sugerencias para mejorar el equipo de laboratorio en el primer nivel de atención?
- Que hubiera más personal de laboratorio, lo que le comentaba de los equipos para poder realizar todas las pruebas que necesita un paciente crónico para un mejor seguimiento y creo que solo eso.
